# Supplementary material for: Generative Artificial Intelligence Models in Clinical Infectious Disease Consultations: A Cross-Sectional Analysis Among Specialists and Resident Trainees
Source: Healthcare (Basel). 2025 Mar 27;13(7):744. doi: 10.3390/healthcare13070744 (PMC11988841; doi:10.3390/healthcare13070744)
Supplement: Supplementary file 1 [file healthcare-13-00744-s001.zip › healthcare-3500109-supplementary/Supplementary file S1.pdf]

Supplementary File S1

Supplementary Appendix

Index

1. Clinical scenarios……………………………………………………………… 3 – 36
2. Artificial intelligence (AI)-generated outputs…………………………………. 37 – 334

Clinical scenarios

Clinical scenario 1

<u>CLINICAL SCENARIO</u>

BASIC DEMOGRAPHICS & UNDERLYING MEDICAL CONDITIONS

F/56. No known drug allergies. Lives in a hostel. Hypertension. Crouzon's syndrome and Apert's syndrome. Mental retardation. Hearing impairment. Left parotid swelling 2011 with negative microbiological workup and inadequate sample for histology, resolved. Pulmonary tuberculosis (TB) July 2006, with bilateral pleural effusion and pleural fluid for Mycobacterium tuberculosis complex DNA polymerase chain reaction (PCR) detected. Multiple medication adjustments due to pancytopenia, tolerated isoniazid, levofloxacin, pyrazinamide. Noted pelvic abscess with computed tomography (CT)-guided aspiration and drainage March 2007, fluid acid fast bacilli (AFB) smear positive 10+ but culture negative, no TB DNA PCR. Laparotomy and drainage for pelvic abscess July 2007 but only sent peritoneal fluid for AFB smear and culture which is negative. Received anti-TB treatment till July 2008.

Systemic Lupus Erythematosus (SLE) since 2010 with Class III+V lupus nephritis presented with fever, vasculitic skin rash, pancytopenia, active serology. Renal biopsy: Combined membranous and focal proliferative lupus nephritis, class V + III-G(A). Treated with prednisolone and added azathioprine since 18 March 2011, Stopped azathioprine in 2012 due to leukopenia. Relapsed lupus nephritis March 2020 with increase in urine protein. Latest on hydroxychloroquine, mycophenolate mofetil (MMF) 500 mg BD and prednisolone 5 mg daily

CURRENT ADMISSION

Admitted for fever since 18 November 2023. No localizing symptoms except mild right chest wall pain, treated with a course of amoxicillin-clavulanate. Last menstrual period 8 years ago with no post-menopausal bleed. Bowel opening once per day with no change in bowel habit/per-rectal bleeding. No cough/hemoptysis. No constitutional symptoms.

PHYSICAL EXAMINATION FINDINGS

Swinging low grade fever. Blood pressure and pulse unsupported, on room air. Not septic looking. No palpable cervical/groin lymphadenopathy. Chest clear. Abdomen soft non-tender. Calves soft. No lower limb edema. Drip site clean. Nil active complaint from patient. Redness noted over left lateral finger, claim long-term according to patient, non-tender on movement. No rash.

INVESTIGATION RESULTS

Haemoglobin (Hb) 11.3 g/dL, White blood cell (WBC) 6.21 x10^9/L, Platelet (Plt) 188 x10^9/L, Absolute Neutrophils Count (ANC) 0.48 x10^9/L, Serum creatinine (sCr) 32 umol/L, Alkaline phosphatase (ALP) 77 U/L, Alanine aminotransferase (ALT) 16 U/L, C-reactive protein (CRP) 8.53 mg/dL. C3, C4, Procalcitonin pending, Blood culture (1 December 2023) pending

ANTIMICROBIALS & TREATMENTS

- Piperacillin-tazobactam 1 December 2023 until now

• Doxycycline 4 December 2023 until now

Clinical scenario 2

<u>CLINICAL SCENARIO</u>
BASIC DEMOGRAPHICS & UNDERLYING MEDICAL CONDITIONS
63/M, allergic to ampicillin and cloxacillin, lives with wife, non-smoker non-drinker, retired construction material industry worker, past history of carcinoma of stomach with operation done in 2017 currently in remission. Positron Emission Tomography-Computed Tomography (PET-CT) May 2023 mild diffuse increased FDG uptake (SUVmax 4.0) in the gastric mucosa, focal increased FDG uptake (SUVmax 3.0) with sclerosis and callus formation seen at left anterior fifth rib, compatible with healing fracture; otherwise unremarkable. No personal history of tuberculosis or contact.
CURRENT ADMISSION
Presented with progressive low back pain more on left side for 5 months associated with loss of weight for few kgs. No associated respiratory, bowel, urinary, or neurological symptoms. No trauma. Private MRI 22 November 2023: irregular, thick-walled, peripherally enhancing cystic or necrotic lesion within left paraspinal musculature (erector spinae, psoas major, quadratus lumborum) from L2 to L4 with bony erosion and infiltration centered at left L2-3 facet joint extending more to left L3 vertebral body, transverse process and posterior element and to lesser extent left L2 inferior facet (5.2 x 5.4 x 7.9 cm LR x AP x CC). Pending computed tomography (CT)-guided biopsy.
PHYSICAL EXAMINATION FINDINGS
Similar low back pain. bilateral lower limb proximal power four plus out of five, otherwise physical examination unremarkable
INVESTIGATION RESULTS
Sputum culture, urine routine microscopy sent today. Absolute Neutrophils Count (ANC) 6.09 x10^9/L, Absolute Lymphocyte Count (ALC) 0.47 x10^9/L, C-reactive protein (CRP) 13.08 mg/dl, Erythrocyte sedimentation rate (ESR) 107 mm/hr, Serum creatinine (sCr) 120 umol/L [Body weight 47kg, Creatinine Clearance by Cockcroft-Gault Equation (CrCl by C-G) = 37.1 ml/min], Liver function test unremarkable, Chest X-ray (CXR) not done, Lumbosacral spine X-ray: known collapse L1
ANTIMICROBIALS & TREATMENTS
• Currently not on antimicrobials

Clinical scenario 3

<u>CLINICAL SCENARIO</u>
BASIC DEMOGRAPHICS & UNDERLYING MEDICAL CONDITIONS
71/M. No known drug allergies. Smoker of 50 years (0.5 pack per day). Social drinker. Uncomplicated senile cataract; received operation. Open appendicetomy. Left frontal

brain tumour. Left frontal supratentorial tumour total excision (1 September 2023) Histology: metastatic small cell carcinoma, consistent with lung primary.
CURRENT ADMISSION
Index admission on 5 October 2023 x right sided weakness since 1 October 2023. Contrast-enhanced computed tomography (CT) brain (5 October 2023): Multiloculated rim-enhancing extra-axial collection at left frontoparietal craniotomy site, epidural collection. Left craniectomy (5 October 2023): frank pus over epidural space, dura intact. Culture showed: Propionibacterium acnes [Penicillin minimum inhibitory concentration (MIC): 0.19 ug/ml] Contrast-enhanced magnetic resonance imaging (MRI) brain (13 October 2023): Epidural collection smaller compared to earlier CT, suggestive of resolving postoperative collection. Given IV ceftriaxone 5 October 2023 to 1 December 2023 (8 weeks) and IV metronidazole 5 October 2023 to 1 December 2023 (8 weeks).
9 November 2023 Positron Emission Tomography-Computed Tomography (PET-CT): Extra-axial ellipsoid collection at craniectomy site, 24 x 11 mm. No increased metabolic activity. Hypermetabolic left lower lobe nodule (25mm x 19mm x 23mm, SUV max 6.3) is consistent with a primary lung cancer. 13 November 2023 contrast-enhanced MRI brain: Previously noted epidural collection has resolved. The adjacent cerebral edema has slightly reduced. The previously noted rim enhancing lesion at the left frontoparietal lobe is similar. There is still corresponding susceptability artefact and this could represent a haemorrhagic brain metastasis. There are no new brain lesions detected. Seizure on 21 November 2023 (while on levetiracetam); added phenytoin (dilantin). Fever and sore throat on 2 December 2023; restarted antibiotics. Then worsening drowsiness.
Transfer back to Hospital Q on 4 December 2023.
PHYSICAL EXAMINATION FINDINGS
Diffuse, generalised erythematous maculopapular skin rash. Heart sounds dual, no murmur. Air entry is equal bilaterally. Abdomen soft, non-tender

Clinical scenario 4

CLINICAL SCENARIO
BASIC DEMOGRAPHICS & UNDERLYING MEDICAL CONDITIONS
62/F, No known drug allergies. Underlying hyperlipidemia, history of closed patent ductus arteriosus (PDA) and repaired coarctation of aorta (CoA)
CURRENT ADMISSION
Admitted on 25 October 2023 for multiple medical issues including new onset heart failure with reduced ejection fracture HFrEF [Left ventricular ejection fraction (LVEF) ~30%]; coronary angiogram shows left anterior descending artery complete total occlusion, fast atrial fibrillation and metastatic adenocarcinoma of lung. Positron Emission Tomography-Computed Tomography (PET-CT) 31 October 2023 showed features suggestive of lung cancer with bilateral intrapulmonary metastases, bilateral pleural metastases, extensive nodal metastases and right adrenal metastasis.

Carcinoembryonic antigen (CEA) 26 ng/ml. Epidermal growth factor receptor (EGFR) negative. Left and right supraclavicular lymph node biopsy pathology: metastatic carcinoma, in keeping with adenocarcinoma.
Desaturation to 100% oxygen requirement in Hospital G thus transferred back for further management. Repeated scan: interval progression of right upper lobe consolidation likely due to enlarging tumor, known multiple intrapulmonary metastasis and lymphangitis carcinomatosis, new right pleural effusion, no pulmonary embolism

PHYSICAL EXAMINATION FINDINGS

Requires 100% oxygen supplementation, afebrile, blood pressure or pulse unsupported, chest bilateral crepitations, pansystolic murmur best heard at left lower sternal border, abdomen soft, drip site not inflamed looking, calves soft

INVESTIGATION RESULTS

Previous sputum culture: commensals only. Blood culture 28 October 2023 no growth, 22 November 2023 pending. Urine Legionella antigen 28 October 2023 negative; sputum acid fast bacilli (AFB) smear negative 3 times, Mycobacterium tuberculosis (TB) complex DNA polymerase chain reaction (PCR) not detected. Nasopharyngeal swabs (NPS) respiratory virus panel 22 November 2023 pending. Absolute Neutrophils Count (ANC) 15 x10^9/L, International normalised ratio (INR) 1.5, Serum creatinine (sCr) 48 umol/L, Alkaline phosphatase (ALP) 173 U/L

ANTIMICROBIALS & TREATMENTS

- Amoxicillin-clavulanate 25 to 27 October 2023;
- Piperacillin-tazobactam 27 to 31 October 2023, 20 November 2023 until now;
- Meropenem 31 October 2023 to 11 November 2023;
- Doxycycline 25 October 2023 to 10 November 2023, 22 November 2023 until now

Clinical scenario 5

<u>CLINICAL SCENARIO</u>

BASIC DEMOGRAPHICS & UNDERLYING MEDICAL CONDITIONS

F/65. Allergy to amoxicillin-clavulanate, ampicillin, thalidomide, cotrimoxazole. No G6PD deficiency. Diabetes mellitus A1c 6.6%. Ischaemic heart disease. Multiple myeloma (ISS1 IgG MM) February 2018, Bortezomib, cyclophosphamide and dexamethasone (VCD) March to July 2018. Autologous stem-cell transplantation (ASCT) 22 August 2018. Relapse August 2020, Daratumumab with lenalidomide and dexamethasone (Dara Rd) November 2020 until April 2021. Isatuximab, carfilzomib and dexamethasone (Isa Kd) January to August 2023

CURRENT ADMISSION

Admitted for neutropenic fever on 12 November 2023. Travelled to Hangzhou for Chimeric Antigen Receptor (CAR) T-Cell Therapy, receiving chemotherapy 21 to 23 October 2023, then CAR T-Cell therapy 24 to 25 October 2023. Developed fever for 6 days then fever down for 4 days. Transferred back to Hong Kong afterwards but respike of fever after. Dry cough with no sputum and watery diarrhea, otherwise no rash, joint pain or urinary symptoms. Ultrasound 14 November 2023: Diffuse increased

liver echogenicity, suggestive of parenchymal disease like fatty liver. Non dilated biliary tract. Gallstones. Focal cystic areas within gallbladder wall, query adenomyomatosis.

PHYSICAL EXAMINATION FINDINGS

Swinging fever. Blood pressure and pulse are unsupported. Not septic looking. Tired. Chest clear. Abdomen soft non-tender. Calves are soft. No lower limb edema. No palpable cervical lymphadenopathy. No rash.

INVESTIGATION RESULTS

Haemoglobin (Hb) 10.2 g/dL, White blood cell (WBC) 1.16 x10^9/L, Platelet (Plt) 38 x10^9/L, Absolute Neutrophils Count (ANC) 0.32 x10^9/L, Serum creatinine (sCr) 48 umol/L, Alkaline phosphatase (ALP) 124 U/L, Alanine aminotransferase (ALT) 20 U/L, Lactate dehydrogenase (LDH) 352 U/L, Chest X-ray (CXR) (15 November 2023) no obvious consolidation, Stool workup (15 November 2023) pending, Sputum culture (13 November 2023) commensals, Nasopharyngeal swabs (NPS) x respiratory virus panel (13 November 2023) Enterovirus/Rhinovirus RNA, real-time reverse transcription–polymerase chain reaction (RT-PCR) detected, Blood culture (12 November 2023, 16 November 2023) pending

ANTIMICROBIALS & TREATMENTS

- Meropenem 12 November 2023 until now,
- Voriconazole 14 November 2023 until now,
- Acyclovir prophylaxis, Pentamidine inhalation

Clinical scenario 6

<u>CLINICAL SCENARIO</u>

BASIC DEMOGRAPHICS & UNDERLYING MEDICAL CONDITIONS

67/M. Labeled as allergic to 'anti-diarrhoea medications'. Lives at home. Left ear deafness for many years. Hypertension. Diabetes mellitus. Hyperlipidaemia. Left central retinal artery occlusion (CRAO) on aspirin. Known bilateral hip heterotopic ossification. Streptococcus suis bacteraemia and meningitis on May 2022, complicated by ventriculitis on MRI brain and recurrent seizure. Received external ventricular drainage with vancomycin intraventricular irrigation. During the same admission, complicated by Methicillin-resistant Staphylococcus aureus (MRSA) bacteraemia (October and November 2022). Echocardiogram at the time did not reveal vegetations. Gallium scan (December 2022): No scintigraphic evidence to suggest active infection. Given prolonged courses of vancomycin, linezolid, daptomycin. Patient became bedbound. Plain CT abdomen and pelvis (December 2022) found left hip erosion.

CURRENT ADMISSION

Clinically admit 28 November 2023 x computed tomography (CT) assessment. Contrast CT Left Hip (28 November 2023): Multiloculated rim-enhancing collection up to 1cm thick at posterior left hip, inferiorly down to ischiofemoral interval and laterally to involve the gluteal muscles, worrisome of underlying septic arthritis with gluteal intramuscular abscess. No obvious osseous erosion or destruction seen. Noted elevated

White blood cell (WBC), C-reactive protein (CRP), and Erythrocyte sedimentation rate (ESR). Left hip aspiration (2 December 2023): Total nucleated cell count 4390 10^6/L (polymorphonuclear cell: 65.8%); culture pending

PHYSICAL EXAMINATION FINDINGS

All along afebrile. Denied hip pain.

Clinical scenario 7

<u>CLINICAL SCENARIO</u>

BASIC DEMOGRAPHICS & UNDERLYING MEDICAL CONDITIONS

F/53. No known drug allergies. Stage IV Mantle cell lymphoma October 2022. Hyper-cyclophosphamide, vincristine, Adriamycin, and dexamethasone plus rituximab (HyperCVAD-R) regimen December 2022 to Mar 2023. Myeloablative haploidentical peripheral blood stem cell transplantation (Haplo-PBSCT) for mantle cell lymphoma 30 August 2023 (donation from son, donor G6PD deficient; cytomegalovirus serostatus: donor sero-negative, recipient sero-positive), neutrophil engraftment on day 18. Cervical intraepithelial neoplasia. Syphilis treated with doxycycline in June to July 2023 for 4 weeks. On Cyclosporin post-transplant.

CURRENT ADMISSION

Index admission to Hospital N for right ankle abscess 3 November 2023. Incision and drainage performed twice, intra-operative findings: 3 x 3 cm abscess, wound swab culture (3 November 2023) Nocardia species, sensitivity test pending. Blood culture (6 November 2023) negative. CT thorax and brain with contrast (9 November 2023): no abnormality detected in the brain. Previously reported left upper lobe nodule, mediastinal lymph nodes are static. No new suspicious lesions or source of infection in the lungs. Spleen shows increase in size. Pending PET-CT 6 December 2023.

PHYSICAL EXAMINATION FINDINGS

Fever since yesterday. Blood pressure and pulse unsupported and on room air. Not septic looking. Chest clear. Abdomen soft non-tender. No palpable lymphadenopathy or other skin lesion detected along both lower limbs. Right ankle wound on dressing. Hickman catheter insertion site non-tender, no discharge. No signs of infective endocarditis. No urinary symptoms. Absolute neutrophil count borderline requiring Granulocyte colony-stimulating factor (G-CSF).

INVESTIGATION RESULTS

Haemoglobin (Hb) 7.4 g/dL, White blood cell (WBC) 5.64 x10^9/L, Platelet (Plt) 64 x10^9/L, Serum creatinine (sCr) 82 umol/L, Alkaline phosphatase (ALP) 58 U/L, Alanine aminotransferase (ALT) 8 U/L, Creatinine Clearance by Cockcroft-Gault Equation (CrCl by C-G) = 61.2 ml/min (Body weight 55.2 kg), Blood culture (12 November 2023) pending, CXR (12 November 2023) no obvious consolidation noted

ANTIMICROBIALS & TREATMENTS

• Imipenem-cilastatin 7 November 2023 until now
• Amikacin 7 November 2023 until now
• Pentamidine inhalation every 3-4 weeks

• G-CSF 11 November 2023
• Posaconazole stopped on 30 October 2023 previously

Clinical scenario 8

<u>CLINICAL SCENARIO</u>
BASIC DEMOGRAPHICS & UNDERLYING MEDICAL CONDITIONS
F/33, No known drug allergies. B-cell acute lymphoblastic leukemia (B-ALL) diagnosed April 2023. Previously given Hyper-cyclophosphamide, vincristine, Adriamycin, and dexamethasone (HyperCVAD) regimen plus dasatinib April to August 2023. Latest bone marrow examination 15 August 2023: Regenerating marrow post-chemotherapy with mildly left-shifted granulopoiesis and no excessive blasts.
CURRENT ADMISSION
Peripheral Blood Stem Cell Transplantation (PBSCT) 28 September 2023 (cytomegalovirus serostatus: donor sero-negative, recipient sero-positive). Cytokine Release Syndrome grade 1 on day 1 given tocilizumab. Developed fever since 10 October 2023, started on Meropenem then developed generalized morbilliform rash over bilateral thigh, then spread to chest, trunk and back, initially was only a small patch over left thigh prior post-PBSCT. Dermatology is seen, difficult to distinguish Graft-versus-host disease (GvHD) and adverse cutaneous drug reaction (ACDR) due to new drugs. Suspected drug eruption. Switched to levofloxacin afterwards. Skin biopsy done and started on steroids and Mycophenolate mofetil (MMF) for treatment of GvHD.
PHYSICAL EXAMINATION FINDINGS
Day 15 post-PBSCT. Swinging fever. Blood pressure and pulse unsupported. On room air. Not septic looking. Facial swelling. Chest clear. Abdomen soft non-tender. Calves are soft. No lower limb edema. Hickman line insertion site non-tender with no surrounding erythema. Generalized morbilliform rash over thigh, upper limb, chest, trunk and back with no active discharge noted. Neurologically well.
INVESTIGATION RESULTS
Haemoglobin (Hb) 7 g/dL, White blood cell (WBC) 1.64 x10^9/L, Platelet (Plt) 77 x10^9/L
Serum creatinine (sCr) 50 umol/L, Alkaline phosphatase (ALP) 98 U/L, Alanine aminotransferase (ALT)13 U/L, Procalcitonin <0.1 mcg/L, C-reactive protein (CRP) 0.95 mg/dl, Chest X-ray (CXR) (13 October 2023) no obvious consolidation, Skin biopsy pathology pending, Aspergillus antigen pending, Cryptococcus antigen pending, Urine culture (11 October 2023) Enterococcus faecium 1500 CFU/ml, Stool culture (11 October 2023) pending, Blood culture (11 October 2023, 13 October 2023) pending
ANTIMICROBIALS & TREATMENTS
• Cotrimoxazole 21 to 29 September 2023,
• Ciprofloxacin 21 September 2023 to 11 October 2023,
• Meropenem 29 September 2023 to 4 October 2023, 11 to 12 October 2023,
• Levofloxacin 12 October 2023 until now,

• Valaciclovir 21 to 27 September 2023,
• Posaconazole 21 September 2023 to 12 October 2023,
• Micafungin 12 October 2023 until now,
• Acyclovir 28 September 2023 until now

Clinical scenario 9

CLINICAL SCENARIO
BASIC DEMOGRAPHICS & UNDERLYING MEDICAL CONDITIONS
89/M, no known drug allergies. Old aged home resident. Underlying coronary artery disease with percutaneous coronary intervention (PCI) done, atrial fibrillation on dabigatran, gastroesophageal reflux disease (GERD), Iron deficiency anaemia not for endoscopy, history of traumatic subdural haematoma (SDH)
CURRENT ADMISSION
Admitted for scald injury by hot soup over inner thighs, ~2% total body surface area (TBSA) deep dermal injury as assessed by burn team. Plan emergency operation on coming Monday
PHYSICAL EXAMINATION FINDINGS
Wound reviewed, not infected looking
INVESTIGATION RESULTS
Absolute Neutrophils Count (ANC) 6.57 x10^9/L, C-reactive protein (CRP) 3.37 mg/dl, Serum creatinine (sCr) 83 umol/L [Body weight 54kg, Creatinine Clearance by Cockcroft-Gault Equation (CrCl by C-G) = 40.8 ml/min], Liver function test unremarkable. Multidrug-resistant organisms (MDRO) screening rectal swab positive for Multi-Drug Resistant Acinetobacter (MDRA), axilla groin Methicillin-resistant Staphylococcus aureus (MRSA) screening positive for MRSA; bilateral groin superficial wound swab 25 November 2023: Diphtheroid bacilli, MRSA, MDRA
ANTIMICROBIALS & TREATMENTS
Nil.

Clinical scenario 10

CLINICAL SCENARIO
BASIC DEMOGRAPHICS & UNDERLYING MEDICAL CONDITIONS
64/M. No known drug allergies. Ex-smoker (stopped since his 30s). Social drinker. Retired. History of caecal tumour (right hemicolectomy in private, 2019); subsequently told benign lesion. History of Covid-19 (August 2023). History of red eye (September 2023)
CURRENT ADMISSION
Initially admitted to Hospital N 11 October 2023 x chest pain, associated with elevated troponin I (managed as acute coronary syndrome). Presented with 1 day of swinging fever on 13 October 2023, then subsided. Blood culture (14 October 2023; 10:09): no growth. 1st dose of augmentin (11:10; 14 October 2023) given after obtaining blood

culture sample. Coronary angiogram (18 October 2023): Mild coronary artery stenosis. Transesophageal echocardiogram (24 October 2023): Thicken non-coronary cusp with 2 mm perforation and severe aortic regurgitation (AR). Due to transesophageal echocardiogram (TEE) findings, cardiology embarked on investigations and treatment for suspected infective endocarditis. Performed 3 sets of blood cultures between October 24 and 25 (unfortunately, IV vancomycin had already been started; which infectious disease team suggested for 4 weeks duration). Bartonella serology pending. Brucella serology negative. Mycoplasma serology negative. Coxiella serology (anti-phase I/II) negative. Developed fever between 5 - 9 November 2023, with chills and rigor, therefore added Piperacillin-tazobactam on top of vancomycin. Then noted new-onset cytopenia, as well as skin rash; so vancomycin was switched to daptomycin. Contrast-enhanced computed tomography (CT) abdomen and pelvis (15 November 2023): no significant pathology identified. Non-specific lesion at segment 4b showed no arterial or delayed enhancement, no rim enhancement. Discharged against medical advice from Hospital N on 17 November 2023. Admit Hospital Q 17 November 2023 x further management. Another episode of fever on 13 November 2023, which persists until now.

PHYSICAL EXAMINATION FINDINGS

Attended the patient at bedside with wife. Not septic looking. No more skin rash. No peripheral signs of infective endocarditis. Diastolic murmur detected, with a collapsing pulse. No personal or contact history of tuberculosis

ANTIMICROBIALS & TREATMENTS

- IV ceftriaxone November 18 till now,
- IV piperacillin-tazobactam November 6 - 17,
- IV daptomycin November 9 - 15,
- IV vancomycin October 24 - November 9,
- Antibiotics free October 22 - 23,
- PO Amoxicillin-clavulanate October 16 - 21,
- IV Amoxicillin-clavulanate October 14 - 16

Clinical scenario 11

<u>CLINICAL SCENARIO</u>

BASIC DEMOGRAPHICS & UNDERLYING MEDICAL CONDITIONS

F/76. No known drug allergies. Diabetes mellitus. Hyperlipidaemia. Depression. Stage IV squamous cell ca lung with Positron Emission Tomography-Computed Tomography (PET-CT) showing diffuse lung nodules with multiple mediastinal and intra-abdominal lymph node. Started chemotherapy in 2021, with Pembrolizumab for 35 cycles. Now on observation. Last PET-CT in May 2023 metabolic complete remission.

CURRENT ADMISSION

Admitted on 21 November 2023 for high fever previously associated with left lower limb cellulitis, seen by general practitioner with improvement with antibiotics.

However, persistent high fever, therefore admitted through accident and emergency department. Since admission the patient noted diplopia (left sixth cranial nerve palsy) with photophobia by the parent team. No personal history of tuberculosis, but the patient's husband has a history of pulmonary tuberculosis. Denied neurological symptoms.

PHYSICAL EXAMINATION FINDINGS

On approaching the patient, Glasgow coma scale fluctuates with occasional difficulty in answering questions. Gross examination showed power 5 out of 5 over all four limbs. Extraocular movement cannot be assessed as patient not completely obeyed command. Facial sensation is grossly unremarkable. No facial asymmetry. No rash seen over body

INVESTIGATION RESULTS

Urine routine analysis not suggestive of urinary tract infection. White blood cell (WBC) 7.30 x10^9/L, Absolute Neutrophils Count (ANC) 5.53 x10^9/L, Absolute Lymphocyte Count (ALC) 1.03 x10^9/L, Serum creatinine (sCr) 113 umol/L, estimated glomerular filtration rate (eGFR) 41 ml/min/1.73 m^2, Liver function test (LFT) static. Chest X-ray (CXR) no definite evidence of consolidation. Hepatitis B surface antigen (HBs Ag) negative. Blood culture on admission no growth so far. Lumbar puncture performed on 22 November 2023, with opening pressure 20 cm H2O, Cerebral spinal fluid (CSF) total cell count (TCC) 33 x10^6/L, Lymphocyte 50%, Monocyte 44%, CSF Protein 1.40 g/L, CSF Glucose 2.2 mmol/L (serum random glucose 5.4 mmol/L). BioFire FilmArray multiplex polymerase chain reaction (PCR) meningitis/encephalitis (ME) panel all not detected, including herpes simplex virus, varicella-zoster virus, enterovirus. Cryptococcal antigen negative. Not sufficient CSF for further investigation.

ANTIMICROBIALS & TREATMENTS

- Piperacillin-tazobactam 21 November 2023;
- Meropenem 22 November 2023;
- Ceftriaxone 22 November 2023 until now;
- Acyclovir 22 November 2023 until now.

Clinical scenario 12

<u>**CLINICAL SCENARIO**</u>

BASIC DEMOGRAPHICS & UNDERLYING MEDICAL CONDITIONS

M/55 Labelled allergic to penicillin presented with syncope in 2007, never tried other beta-lactam antibiotics after the episode of syncope. Poliomyelitis infection with lower limb power zero out of five, in wheelchair. Ischemic heart disease. Paraumbilical hernia. Chronic hepatitis B cirrhosis with multifocal hepatocellular carcinoma on entecavir, received Transarterial chemoembolization (TACE) in March 2023 and July 2023, decided for Drug-eluting bead transarterial chemoembolization (DEB-TACE). Incidental finding of atypical cells in right pleural effusion in September 2023, with further computed tomography (CT) showing multifocal hepatocellular carcinoma with

collapsed right lower lobe. Ultrasound (USG)-guided ascitic drainage on 12 October 2023 showed atypical cells.

CURRENT ADMISSION

Admitted since 5 November 2023 for ascites with paraumbilical hernia. CT 6 November 2023 showed umbilical hernia with a 1.6 x 1.2cm lobulated rim enhancing collection noted within. Mild adjacent inflammatory stranding and overlying skin thickening. Operation on 6 November 2023 for mesh repair of hernia, with peritoneal fluid culture no growth. Redeveloped abdominal discomfort, therefore transferred back to rule out hernia strangulation. Transferred to Hospital Q on 24 November 2023 for further management. CT 25 November 2023 showed a 8.6x3x6.8cm heterogenous hyperdense subcutaneous lesion at paraumbilical region is seen with interval enlargement. Internal gas densities, thin rim of peripheral enhancement and perifocal subcutaneous fat stranding. Operation 27 November 2023 for incision and drainage with intra-operatively noted infected haematoma, with intra-operative culture all no growth. Developed fever on 1 December 2023 then fever down. Blood culture Staphylococcus aureus, pending further susceptibility testing. Patients mainly complained of abdominal distension and occasional pain over ascitic fluid drain site. Cough.

PHYSICAL EXAMINATION FINDINGS

Vitals stable, low grade fever, general condition well. Chest with occasional crepitations. Heart sound dual no murmur. Finger clubbing, no other signs of infective endocarditis. Abdomen distended, soft, non-tender on palpation. Drip site well. No evidence of deep vein thrombosis.

INVESTIGATION RESULTS

Chest X-ray (CXR) showing right lower lobe atelectasis, no definite consolidation seen. White blood cell (WBC) 4.72 x10^9/L, Absolute Neutrophils Count (ANC) 4.02 x10^9/L, Serum creatinine (sCr) 60 umol/L, estimated glomerular filtration rate (eGFR) > 90 ml/min/1.73 m^2, Alkaline phosphatase (ALP) 126 U/L, Alanine aminotransferase (ALT) normal.

ANTIMICROBIALS & TREATMENTS

• Levofloxacin 25 November 2023 until now;
• Metronidazole 1 December 2023 until now

Clinical scenario 13

<u>**CLINICAL SCENARIO**</u>

BASIC DEMOGRAPHICS & UNDERLYING MEDICAL CONDITIONS

M/48. No known drug allergies. Schizophrenia. Obesity. Diabetes mellitus HbA1c 5.0%. Hypertension.

CURRENT ADMISSION

Admitted on 24 November 2023 for perforated peptic ulcer (PPU) with gross peritonitis presented with epigastric pain radiating to the back. Subsequently developed shock with anuric acute kidney injury (AKI). Computed tomography (CT) on 24 November

2023 showed gross pneumoperitoneum with free fluid. Operation on 25 November 2023 showed gross peritoneal contamination with turbid fluid, and 2 cm anterior first part of duodenum (D1) ulcer, with patch repair performed. Pus culture (after antibiotics) grew Candida glabrata. Blood culture no growth on admission

INVESTIGATION RESULTS

White blood cell (WBC) 6.77 x10^9/L, Absolute Neutrophils Count (ANC) 4.91 x10^9/L, Serum creatinine (sCr) 157 umol/L, estimated glomerular filtration rate (eGFR) 44 ml/min/1.73 m^2, Liver function test normal.

ANTIMICROBIALS & TREATMENTS

- Amoxicillin-clavulanate 24 November 2023;
- Piperacillin-tazobactam 24 November 2023 until 1 December 2023;
- Micafungin 24 November 2023, 26 November 2023 until now

Clinical scenario 14

<u>CLINICAL SCENARIO</u>

BASIC DEMOGRAPHICS & UNDERLYING MEDICAL CONDITIONS

72/F, no known drug allergies. Diabetes mellitus with nephropathy, end stage renal failure on continuous ambulatory peritoneal dialysis (CAPD), Hypertension, Right persistent clinically significant macular edema (CSME). Tenckhoff catheter removed June 2023 due to Escherichia coli CAPD peritonitis, recently reinserted Tenckhoff catheter on 14 November 2023

CURRENT ADMISSION

Admitted for fever, vomiting and productive cough for 3 days without diarrhea. No recent sick contact, animal contact, raw animal product intake. Peritoneal dialysis fluid (PDF) White blood cell (WBC) 1484 down to 69 with intraperitoneal antimicrobials. No headache, neck stiffness, rash, joint pain, urinary symptoms, diarrhea. Dental: no dental source of sepsis

PHYSICAL EXAMINATION FINDINGS

Glasgow coma scale (GCS) full, no neurological symptoms or signs, fever down, Blood pressure and pulse unsupported and on room air, chest clear, heart sound dual with no murmur, abdomen soft, heparin block site not inflamed looking, no rash or joint swelling

INVESTIGATION RESULTS

Blood culture 25 November 2023 Campylobacter fetus (Sensitive to amoxicillin-clavulanate, ciprofloxacin, erythromycin, imipenem), repeated Blood culture 27 November 2023 no growth; multiple PDF culture no growth. Absolute Neutrophils Count (ANC) 18.15 x10^9/L, Serum creatinine (sCr) 1377 umol/L, Liver function test unremarkable, Chest X-ray (CXR) no obvious consolidation

ANTIMICROBIALS & TREATMENTS

- Amoxicillin-clavulanate 25 to 29 November 2023;
- azithromycin 29 November 2023 until now;
- Intraperitoneal cefazolin and amikacin 25 November 2023 until now

Clinical scenario 15

<u>**CLINICAL SCENARIO**</u>
**BASIC DEMOGRAPHICS & UNDERLYING MEDICAL CONDITIONS**
76/M, No known drug allergies. Underlying unresectable carcinoma of pylorus with gastrojejunal bypass done and last chemotherapy July 2022 for palliative radiotherapy if symptomatic
**CURRENT ADMISSION**
Admitted for persistent diarrhea. Noted watery diarrhea since early October, then noted rectal mass in November 2023, biopsy showed poorly differentiated carcinoma, Positron Emission Tomography-Computed Tomography (PET-CT) 15 November 2023 showed rectal tumor, synchronous carcinoma of transverse colon, carcinoma of pylorus disease progression, right hydronephrosis and diffuse urinary bladder wall thickening to 1.4cm. Persistent diarrhea with no improvement
**PHYSICAL EXAMINATION FINDINGS**
Tired looking, stable afebrile, lower abdominal diffuse tenderness, persistent watery diarrhea four to five times per day
**INVESTIGATION RESULTS**
Blood culture 22 November 2023 Gram negative bacilli noted, culture pending. Urine routine microscopy numerous white blood cell, leukocyte esterase large, bacteria seen; stool culture pending. Absolute Neutrophils Count (ANC) 11.8 x10^9/L, Haemoglobin (Hb) 9 g/dL, Platelet (Plt) 11 x10^9/L (smear review occasional fragmented red blood cell), International normalised ratio (INR) 1.5, activated partial thromboplastin time (aPTT) 42.6 seconds, Serum creatinine (sCr) 191 umol/L [Body weight 60kg, Creatinine Clearance by Cockcroft-Gault Equation (CrCl by C-G) 24.7 ml/min], Total bilirubin 24 umol/L, Alkaline phosphatase (ALP) 164 U/L, Chest X-ray (CXR) no obvious consolidation
**ANTIMICROBIALS & TREATMENTS**
Nil.

Clinical scenario 16

<u>**CLINICAL SCENARIO**</u>
**BASIC DEMOGRAPHICS & UNDERLYING MEDICAL CONDITIONS**
38/F, No known drug allergies. Methicillin-resistant Staphylococcus aureus (MRSA) carrier, Carcinoma of gallbladder with whipple done in March 2023, CT August 2023 showed right upper quadrant recurrence and nodal recurrence at portal hepatitis with portal vein invasion and mesenteric and left para aortic lymph node
**CURRENT ADMISSION**
Initially admitted for deranged liver function test later noted breakthrough fever with urgent left percutaneous transhepatic biliary drainage (PTBD) done, then transferred to Hospital T for further management. Transferred back as noted on off fever while on

meropenem, USG 20 November 2023 noted prominent right intrahepatic ducts, then right external-internal PTBD done on 27 November 2023 with left PTBD removed

PHYSICAL EXAMINATION FINDINGS

Day 25 of Meropenem. Persistent swinging fever since 20 November 2023, tachycardiac, lowish blood pressure, on room air, not septic looking, chest clear, abdomen soft, calves soft, drip site not inflamed looking, bile stain output from right PTBD

INVESTIGATION RESULTS

Left PTBD bile culture 4 November 2023 Klebsiella pneumoniae (Sensitive to amoxicillin-clavulanate); Right PTBD bile culture 28 November 2023 pending. Blood culture 29 November 2023 pending; urine routine microscopy 28 November 2023 not suggestive of urinary tract infection. Absolute Neutrophils Count (ANC) 2.78 x10^9/L, C-reactive protein (CRP) 6.83 mg/dl. Procalcitonin (PCT) 2.6 ng/ml, Serum creatinine (sCr) 39 umol/L, Liver function test improving trend after right external-internal PTBD, Chest X-ray (CXR) no obvious consolidation

ANTIMICROBIALS & TREATMENTS

• Cefuroxime and metronidazole 2 to 6 November 2023;
• meropenem 6 November 2023 until now

Clinical scenario 17

CLINICAL SCENARIO

BASIC DEMOGRAPHICS & UNDERLYING MEDICAL CONDITIONS

M/86, No known drug allergies. Bulbous urethral stricture, Hypertension, Asthma.

CURRENT ADMISSION

Patient went to Jiangxi, China for a travel tour and was found lying at the poolside of a hot spring, unwitnessed. Diagnosed as aspiration pneumonia with suspected transient ischemic attack. Computed tomography (CT) thorax (plain) shows bilateral pneumonitis and CT brain atrophic changes. Empirically given Sulbactam + Moxifloxacin with methylprednisolone, intubated on 14 October 2023, stepped up to Imipenem + Moxifloxacin. Bronchoscopy found inflammatory changes with sputum retention.

Transferred to Hong Kong on 15 October 2023 and admitted to Hospital N. Bronchoalveolar lavage (BAL) culture (16 October 2023) Candida tropicalis; Tuberculosis, Legionella, Cytomegalovirus (CMV), Mycoplasma, Chlamydia polymerase chain reaction (PCR) not detected. Extubated 17 October 2023, weaned down to nasal cannula on 18 October 2023. CT pulmonary angiogram 25 October 2023: poor opacification of the subsegmental branch of left lower lobe lateral basal segment pulmonary artery. Patchy ground glass opacities and fibrosis/atelectasis at both lungs. Ultrasound showed no evidence of deep vein thrombosis in bilateral thighs. Noted deranged liver function since 28 October 2023.

Transferred to Hospital Q on 28 October 2023. Urgent CT performed noted emphysematous cystitis with foley in-situ, tiny distal common duct stones and sludges, fibrosis and traction bronchiectasis at lung bases. Urology seen, suggests early CT pelvis in 1 month to assess for resolution of emphysematous cystitis. ERCP performed 31 October 2023 mildly dilated common bile duct but with no common bile duct stone, intrahepatic ducts dilated, impression: recurrent pyogenic cholangitis. Persistent deranged liver function test, repeat Endoscopic Retrograde Cholangiopancreatography (ERCP) on 6 November 2023and 7 November 2023, noted swollen papilla, common bile duct cannulated with stent insertion. Blood culture (3 November 2023) grew Enterococcus faecium

PHYSICAL EXAMINATION FINDINGS

Afebrile. Blood pressure and pulse are unsupported and in room air. Not septic looking. Chest clear. Heart sound dual. Abdomen soft non-tender. Bilateral kidneys non-tender on balloting. No suprapubic tenderness. Calves are soft. No lower edema. Current drip site is clean and not infected. No peripheral signs of infective endocarditis

INVESTIGATION RESULTS

Haemoglobin (Hb) 10.8 g/dL, White blood cell (WBC) 13.91 x10^9/L, Platelet (Plt) 156 x10^9/L, Serum creatinine (sCr) 91 umol/L, Bilirubin 105 umol/L, Alkaline phosphatase (ALP) 598 U/L, Alanine aminotransferase (ALT) 114 U/L, C-reactive protein (CRP) 16.83 mg/dl, Creatinine Clearance by Cockcroft-Gault Equation (CrCl by C-G) = 43.1 ml/min. Urine culture (29 October 2023, 31 October 2023) Candida glabrata. Bile culture (7 November 2023) Enterococcus faecium. Blood culture (31 October 2023) no growth, (3 7 November 2023) Enterococcus faecium

ANTIMICROBIALS & TREATMENTS

- Piperacillin-tazobactam 16 to 21 October 2023,
- Meropenem 22 October 2023 until now,
- Vancomycin 8 November 2023 until now

Clinical scenario 18

<u>**CLINICAL SCENARIO**</u>

BASIC DEMOGRAPHICS & UNDERLYING MEDICAL CONDITIONS

F/62. No known drug allergies. Diabetes mellitus HbA1c 6.4%. Fatty liver. Right breast mass followed up by Hospital T. Carcinoma of cervix with hysterectomy > 10 years.

CURRENT ADMISSION

Admitted on 2 December 2023 for fever for 3 days associated with cough, sore throat, runny nose, and myalgia on 30 November 2023. Noted diarrhea for 1 day with five to six times watery loose stool on 2 December 2023. Vomiting of undigested food. No urinary symptoms. Travelled to Okinawa 26 November 2023 to 2 December 2023 with family members, none of them exhibited similar symptoms. Intake of raw fish in Okinawa. Developed fever on admission, with fever slowly improving with

Amoxicillin-clavulanate and Doxycycline. Patient now noted residual shortness of breath and improving diarrhea.

INVESTIGATION RESULTS

Chest X-ray (CXR) on 3 December 2023 showed pulmonary infiltrates over right lower zone (RLZ). White blood cell (WBC) 6.51 x10^9/L, Absolute Neutrophils Count (ANC) 3.12 x10^9/L, Serum creatinine (sCr) 55 umol/L, estimated glomerular filtration rate (eGFR) > 90 ml/min/1.73 m^2, Alanine aminotransferase (ALT) 53 U/L, Aspartate aminotransferase (AST) 56 U/L. Urine Legionella antigen negative. Sputum acid fast bacilli (AFB) smear negative. Urine routine not suggestive of urinary tract infection, bacterial culture no growth.

ANTIMICROBIALS & TREATMENTS

• Amoxicillin-clavulanate 3 December 2023 until now;

• Doxycycline 3 December 2023 until now.

Clinical scenario 19

<u>CLINICAL SCENARIO</u>

BASIC DEMOGRAPHICS & UNDERLYING MEDICAL CONDITIONS

78/F. No known drug allergies. Diabetes mellitus. Hepatitis B virus related hepatocellular carcinoma, on entecavir. Carcinoma of right breast, mastectomy 2011 with adjuvant Letrozole (Femara). Schizophrenia. Glaucoma. History of bleeding duodenal ulcer.

CURRENT ADMISSION

First admit 1 November 2023 x fever. Transfer back to Hospital Q 17 November 2023 for further management.

PHYSICAL EXAMINATION FINDINGS

Afebrile. Not septic looking. No chills or rigors. No active complaints. Heart sounds dual, no murmur. No peripheral signs of infective endocarditis. Abdomen soft, non-tender

INVESTIGATION RESULTS

Blood culture (1 November 2023): Escherichia coli. Ultrasound (USG, 2 November 2023): Bilobar multifocal HCC. Contrast-enhanced computed tomography (CT) abdomen and pelvis (17 November 2023): The previously noted hypodense lesion at segment III posterior aspect shows interval increase in size with ill-defined border, now measuring 2.8 x 1.5cm. A new 2.2 x 2.0cm lobulated hypodense lesion over segment II. These show no definite rim-enhancement. Overall features could represent early abscess formation. Blood culture (16 November 2023): Escherichia coli.

Clinical scenario 20

<u>CLINICAL SCENARIO</u>

BASIC DEMOGRAPHICS & UNDERLYING MEDICAL CONDITIONS

M/63, No known drug allergies. Previously a construction site worker, now cleaner. Chronic hepatitis B. Hypertension. Diabetes mellitus A1c 7.1%. Asthma. Right lung abscess in 2017 with sputum Mycobacterium tuberculosis (TB) complex DNA polymerase chain reaction (PCR) detected and acid fast bacilli (AFB) culture positive. Started HRZE (isoniazid, rifampicin, pyrazinamide, ethambutol) since October 2017, switched to HR (isoniazid, rifampicin) March 2018, then completed treatment 30 July 2018.

Latest reassessment CT: previously noted irregular right upper lobe lesion shows interval reduction in size and interval resolution of cavitation. Right middle lobe consolidation shows interval reduction. Similar residual ground glass opacity, static perilesional right upper lobe and right middle lobe nodules. Static bilateral multiple lung nodules involving bilateral upper, right middle and bilateral lower lobes with upper zone predominance are in favour of background pneumoconiosis. Bilateral enlarged, calcified paratracheal nodes remain similar and can be related to either of the above conditions.

CURRENT ADMISSION

Admitted for epistaxis with thrombocytopenia, suspected immune thrombocytopenia, no improvement with intravenous immunoglobulin (IVIG). Bone marrow examination pending. Haematology team plan for steroid treatment. Chest X-ray (CXR) shows increased right upper zone and left middle zone infiltrates, apical fibrocalcific changes compared to previous CXR. Pending high resolution computed tomography (CT) thorax 20 October 2023.

PHYSICAL EXAMINATION FINDINGS

Afebrile. Blood pressure and pulse unsupported. On room air. On transfusion. Nil active complaint. No palpable lymph nodes. Chest fine crepitations over bilateral apical area. Abdomen soft non-tender. Drip site clean. Generalized petechiae.

INVESTIGATION RESULTS

Haemoglobin (Hb) 11.7 g/dL, White blood cell (WBC) 6.67 x10^9/L, Platelet (Plt) 7 x10^9/L, Serum creatinine (sCr) 98 umol/L, Liver function test not deranged.

ANTIMICROBIALS & TREATMENTS

Nil

Clinical scenario 21

<u>CLINICAL SCENARIO</u>

BASIC DEMOGRAPHICS & UNDERLYING MEDICAL CONDITIONS

51/M, no known drug allergies. Schizophrenia on olanzapine and citalopram. Works as a cleaner, keeps a pet stingray.

CURRENT ADMISSION

Admitted for worsening wound infection. Injured by pet stingray over right hand dorsum on 9 November 2023 with cellulitic changes. Attended Hospital Q accident and emergency department same day, given amoxicillin-clavulanate. Reviewed on 13

November 2023 and added ciprofloxacin as noted no improvement in wound. Persistent wound infection noted during review on 27 November 2023 and given doxycycline alone.

PHYSICAL EXAMINATION FINDINGS

Patient in dental for tooth extraction prior to emergency operation. Vitals from clinical management system: stable, afebrile and on room air. Clinical photo seen, noted 2x4cm wound over right hand dorsum with underlying muscles and tendons seen, necrotic tissue, purulent discharges, cellulitic changes noted with erythema up to elbow

INVESTIGATION RESULTS

Deep wound swab 27 November 2023 gram stain no organism seen, White blood cell (WBC) negative, culture Corynebacterium striatum, methicillin-sensitive Staphylococcus aureus (MSSA; sensitive to levofloxacin). Absolute neutrophil count (ANC) normal, Erythrocyte sedimentation rate (ESR) 11 mm/hr, C-reactive protein (CRP) pending, liver and renal function test unremarkable, hand X-ray no obvious osteomyelitis changes

ANTIMICROBIALS & TREATMENTS

- Amoxicillin-clavulanate 9 to 19 November 2023;
- ciprofloxacin 13 to 19 November 2023;
- doxycycline 27 to 29 November 2023;
- levofloxacin 29 November 2023 until now

Clinical scenario 22

<u>CLINICAL SCENARIO</u>

BASIC DEMOGRAPHICS & UNDERLYING MEDICAL CONDITIONS

50/M. Labeled as allergic to IV contrast, gelofusin. Security guard. Ex-smoker & ex-drinker. Lives with family. History of acute pancreatitis (2022, query due to alcoholism). Case of Stage IV NK/T-cell lymphoma. Histology confirmed from abdominal wall skin lesion, Epstein-Barr encoding region (EBER) in situ hybridization (ISH) positive. Initially presented with fever, malignant biliary obstruction, and deranged liver function. Contrast-enhanced computed tomography (CT) abdomen and pelvis (July 2023) showed multiorgan involvement, including pancreas, lymph nodes, kidneys, lungs, and skin. Percutaneous transhepatic biliary drainage (PTBD) inserted. Started SIMPLE (dexamethasone, methotrexate, ifosfamide, L-asparaginase, etoposide) (50%) since August 2023.

CURRENT ADMISSION

Clinically admit 2 October 2023 x SIMPLE (II) 50% (started 12 October 2023). Positron Emission Tomography-Computed Tomography (PET-CT) scan (2 November 2023): Pancreas currently shows diffuse swelling over its head to uncinate process region with heterogeneous hypermetabolism (SUVmax 5.2) and mild peripancreatic strandings. Further complicated by abdominal pain, rising amylase and lipase. Contrast-enhanced CT abdomen and pelvis (4 November 2023): A 2.8 x 4.0 x 2.8cm (WxAPxCC) pseudoaneurysm is seen arising from gastroduodenal artery (GDA) in

pancreatic head. Contrast-enhanced CT abdomen and pelvis (5 November 2023): Enlarging pseudoaneurysm, measuring 3.9 x 5.7 x 4.8cm (WxAPxCC). 1st Coil embolisation of gastroduodenal artery (GDA) pseudoaneurysm on 5 November 2023. 2nd Embolisation of inferior pancreaticoduodenal artery branches on 7 November 2023. Contrast-enhanced CT abdomen and pelvis (11 November 2023): Interval enlargement of known pseudoaneurysm in pancreatic head, it now measures up to 6.5x7.7x4.8cm (TSxAPxCC). There is also interval increase in fat stranding around the pseudoaneurysm sac and pancreas. Increase in swelling of the pancreas with the main pancreatic duct dilated up to 1.5cm at pancreatic body (previous ~1.0cm). Common duct proximal to pancreatic head shows further mild dilatation up to 1.8cm. Bilateral central intrahepatic duct (IHD) appear more prominent. The central IHDs are dilated up to 9mm. 3rd Left gastroepiploic artery embolisation on 13 November 2023 (requiring proximal embolisation due to tortuosity, risk of non-target embolisation (predominantly affecting the stomach). Right external PTBD insertion on 16 November 2023

PHYSICAL EXAMINATION FINDINGS

Post-chemotherapy 5 weeks and 5 days. Swinging fever since 15 November 2023 until now. Clinically not septic looking. Nil active complaints. No subjective fever. No chills or rigors. Abdomen soft non-tender. Previous vascular access wounds healed, non-tender on palpation. Heart sounds dual, no murmur. Pending PET-CT scan 23 November 2023

ANTIMICROBIALS & TREATMENTS

- IV vancomycin 19 November until now,
- IV meropenem 17 November until now,
- IV amikacin 20 November until now,
- IV ticarcillin-clavulanate 10 – 17 November 2023,
- IV meropenem 24 October 2023 to 10 November 2023,
- IV micafungin 13 October 2023 to 8 November 2023

Clinical scenario 23

<u>CLINICAL SCENARIO</u>

BASIC DEMOGRAPHICS & UNDERLYING MEDICAL CONDITIONS

F/51, No known drug allergies. Born in China, previously worked in garment industry, came to Hong Kong in 2010 and worked in old aged home since. Married with one son, husband and son in China. Live in employee quarters. Intra-uterine Contraceptive Device (IUCD) insertion done 10 years ago.

CURRENT ADMISSION

Admitted for deranged liver function and fever. Travelled to Zhanjiang, China from 4 to 6 October 2023 and took cooked seafood including shrimp and crab, chicken and eggs etc. No freshwater contact, hiking or insect bite. Developed 3-4 days of diarrhea afterwards, resolved with symptomatic medications from private doctor.

Presented with dry mouth and tiredness on 18 October 2023, given symptomatic medications and 2 days of antibiotics by private. Noted diarrhea two to three times per

day with yellowish loose stool, also pale yellow urine. Attended accident and emergency department on 24 October 2023 with symptomatic medications again. Took 2 days of traditional chinese medications but was unsure about its ingredients. No other travel history according to the patient. No constitutional symptoms prior to onset of fever including weight loss.

PHYSICAL EXAMINATION FINDINGS

Swinging fever up to 39.5'C. Blood pressure and pulse are unsupported and on room air. Not septic looking. Chest clear. Abdomen faint spots of erythema over abdomen. Query palpable left upper zone mass. Query Spleen tip. No right upper quadrant tenderness. Calves are soft. No lower limb edema.

INVESTIGATION RESULTS

Haemoglobin (Hb) 11.2 g/dL, White blood cell (WBC) 8.93 x10^9/L, Platelet (Plt) 136 x10^9/L, Serum creatinine (sCr) 54 umol/L, Alkaline phosphatase (ALP) 321 U/L, Alanine aminotransferase (ALT) 357 U/L, C-reactive protein (CRP) 14.46 mg/dl, Chest X-ray (CXR) (28 October 2023) left pleural effusion. Blood culture (28 October 2023) pending. Stool culture (28 October 2023) pending. Sputum culture (28 October 2023) pending. Urine culture (28 October 2023) pending

ANTIMICROBIALS & TREATMENTS

• Amoxicillin-clavulanate 28 October 2023 until now,
• Doxycycline 29 October 2023 until now

Clinical scenario 24

CLINICAL SCENARIO

BASIC DEMOGRAPHICS & UNDERLYING MEDICAL CONDITIONS

F/77, No known drug allergies. Gastric ulcer, partial gastrectomy in private sector > 10 years ago, Lap cholecystectomy in 2011, Lymphoma treated in Hospital S with 5 cycles of chemotherapy around 2010 but details unavailable. Advanced inoperable carcinoma of pancreas on best supportive care (BSC) with chemotherapy and radiotherapy 44Gy/20fr with Xeloda in Hospital SH, completed in September 2022. Latest Positron Emission Tomography-Computed Tomography (PET-CT) 31 May 2023 (compared with PET-CT December 2022) shows known pancreatic malignancy at uncinate process similar in size with mild increase in uptake, causing pancreatic & common bile duct (CBD) dilatation; portal vein, celiac, superior mesenteric artery invasion. Malignant biliary obstruction with right Percutaneous Transhepatic Biliary Drainage (PTBD) inserted June 2023, failed internalization. Left external PTBD insertion 30 August 2023 with internalization October 2023. Not for further chemotherapy due to poor marrow function.

CURRENT ADMISSION

Admitted for right abdominal pain with fever. Computed tomography (CT) abdomen with contrast performed on 28 October 2023 showing (a) Necrotic pancreatic head carcinoma showing no significant interval enlargement, 3.4cm across, with evidence of invasion of extrahepatic biliary tree up to hepatic hilum, and posteriorly to

retroperitoneum. (b) Interval removal of right PTBD and insertion of left PTBD with internalization. Decompression of left intrahepatic ducts with right intrahepatic biliary tree obstruction, probably at hilar level. (c) New multiple bilobed tiny subcentimeter hepatic lesions, could be due to new hepatic metastasis or small abscesses. Suspected peritoneal metastasis.

PHYSICAL EXAMINATION FINDINGS

Afebrile. Blood pressure and pulse are unsupported and on room air. Not septic looking. Chest clear. Abdomen distended but soft non-tender. Previous PTBD site wound mild surrounding erythema. Left PTBD seen with bile in bedside bag. Foley bag urine clear no sediment. Drip site clean.

INVESTIGATION RESULTS

Haemoglobin (Hb) 9.5 g/dL, White blood cell (WBC) 11.21 x10^9/L, Platelet (Plt) 101 x10^9/L, Serum creatinine (sCr) 123 umol/L, Bilirubin (Bil) 46 umol/L, Alkaline phosphatase (ALP) 913 reduced to 559 U/L, Alanine aminotransferase (ALT) 39 U/L, Creatinine Clearance by Cockcroft-Gault Equation (CrCl by C-G) = 24.5 ml/min. Urine culture (27 October 2023) no growth. Bile culture (27 October 2023) pending. Blood culture (26 October 2023) pending, (27 October 2023) preliminary Enterobacter species.

ANTIMICROBIALS & TREATMENTS

• Amoxicillin-clavulanate 27 October 2023,
• Piperacillin-tazobactam 27 October 2023,
• Meropenem 27 October 2023 until now

Clinical scenario 25

<u>CLINICAL SCENARIO</u>

BASIC DEMOGRAPHICS & UNDERLYING MEDICAL CONDITIONS

51/F, No known drug allergies. Underlying hypertension, subclinical hypothyroidism, chronic sinusitis, history of nasopharyngeal carcinoma with radiotherapy done in 1995. adjustment disorder with mixed anxiety and depressed mood

CURRENT ADMISSION

Admitted for osteoradionecrosis of skull base with left parapharyngeal abscess presenting as fever, headache and nasal discharge with excision of lesion of nasopharynx, marsupialisation of nasopharyngeal abscess and turbinate reduction done on 5 October 2023. Reassessment computed tomography (CT) 24 November 2023 showed residual infective collection (4.5 cm to 3.6 cm), intraluminal hypodensity in the right proximal vertebral artery extending from the origin to C6 level. Percutaneously inserted central catheter (PICC) inserted on 14 November 2023. Consult for oral antimicrobials

PHYSICAL EXAMINATION FINDINGS

Remained stable afebrile. Body weight 38kg only.

INVESTIGATION RESULTS

Tissue specimen 5 October 2023 Gram stain Gram positive bacilli++, yeast+; white blood cell+; culture: extended spectrum beta lactamase (ESBL)-producing Klebsiella pneumoniae (resistant to cefuroxime, sensitive to levofloxacin meropenem), Enterococcus faecalis (sensitive to ampicillin), Lactobacillus species (Sensitive to penicillin G), Capnocytophaga species (sensitive to ampicillin), Bacteroides thetaiotaomicron (sensitive to meropenem resistant to moxifloxacin and metronidazole), Candida glabrata.
Left nasopharyngeal tissue 8 November 2023 Gram stain no organism seen, white blood cell negative; culture: ESBL-producing K. pneumoniae (resistant to cefuroxime, intermediate resistant to levofloxacin, sensitive to meropenem), Pseudomonas aeruginosa (sensitive to ciprofloxacin), Candida albicans. Complete blood count (CBC), C-reactive protein (CRP), liver and renal function tests unremarkable

ANTIMICROBIALS & TREATMENTS

- Amoxicillin-clavulanate 3 to 8 October 2023;
- cefotaxime and metronidazole 8 October 2023 until now

Clinical scenario 26

<u>CLINICAL SCENARIO</u>

BASIC DEMOGRAPHICS & UNDERLYING MEDICAL CONDITIONS

M/70. No known drug allergies. Smoker, Social drinker. Diabetes mellitus HbA1c 8.0%. Hypertension. Benign prostate hyperplasia. Fatty liver. Hepatitis B surface antigen (HBs Ag) positive on entecavir. Ischaemic heart disease.

CURRENT ADMISSION

Admitted on 7 November 2023 for biliary obstruction presented with derangement of liver function. Incidental finding of high CA 19.9 839 U/mL. Endoscopic Retrograde Cholangiopancreatography (ERCP) on 8 November 2023 showed dilated left intrahepatic ducts (IHDs) with stricture over left hepatic duct, with stent inserted and brush cytology showing atypical cells. Bile culture grew Escherichia coli, Lactobacillus, and Stenotrophomonas maltophilia. Post-ERCP complicated by pancreatitis. Developed fever again on 15 November 2023 with derangement of liver function. ERCP 15 November 2023: similar stricture over left hepatic duct, therefore plastic stent inserted again. Cytology pending.
Patient taken over to Hospital Q on 18 November 2023 for further management. computed tomography (CT) scan 18 November 2023: Prominent right IHDs up to 4mm in calibre. Mildly dilated left (IHD) up to 6mm in calibre. ERCP 19 November 2023: Cholangiogram: contrast readily goes into dilated and tortuous left duct. There is a segment of stricture of the right duct and dilated right anterior duct.

PHYSICAL EXAMINATION FINDINGS

Fever since 18 November 2023, Body weight 79.3 kg

INVESTIGATION RESULTS

White blood cell (WBC) 10.99 x10^9/L, Absolute Neutrophils Count (ANC) 9.82 x10^9/L, Serum creatinine (sCr) 88 umol/L, estimated glomerular filtration rate

(eGFR) 76 ml/min/1.73 m^2, rising trend bilirubin, improving Alkaline phosphatase (ALP). Blood culture on 10 November 2023, 15 November 2023, 17 November 2023, and 18 November 2023 all no growth so far. Chest X-ray (CXR) on 18 November 2023 showing bilateral pulmonary infiltrates. No positive Multi-Drug Resistant Organisms (MDROs) screening so far. Pending Positron Emission Tomography-Computed Tomography (PET-CT) appointment on 22 November 2023.

ANTIMICROBIALS & TREATMENTS

- Amoxicillin-clavulanate 7 to 10 November 2023;
- Ceftriaxone (1g IV q24h) 11 to 17 November 2023;
- Metronidazole 10 to 15 November 2023;
- Meropenem 17 November 2023 until now;
- Levofloxacin 18 November 2023 until now;
- Amikacin 18 November 2023 until now;
- Vancomycin 19 November 2023 until now

Clinical scenario 27

<u>CLINICAL SCENARIO</u>

BASIC DEMOGRAPHICS & UNDERLYING MEDICAL CONDITIONS

F/52. No known drug allergies. Right handed. Fish hawker. Adjustment disorder with previous suicidal gesture with jumping from bridge and drug overdose. Left distal radius fracture with open reduction internal fixation (ORIF) on 20 December 2022

CURRENT ADMISSION

Admitted for right hand tenosynovitis, with history of right hand stung by fish sting during work, together with fever and chills. Erythema and swelling rapidly spread from injury site to the wrist area prior to admission. Operation 4 December 2022 for incision and drainage, intra-operatively noted tenosynovitis of ring finger and little finger extensor tendons, with moderate amount of dirty fluid and synovitis. Intraoperative specimen culture no growth. Acid fast bacilli (AFB) smear negative. AFB culture pending. Histology not sent.

PHYSICAL EXAMINATION FINDINGS

Patient well with no active complaint.

INVESTIGATION RESULTS

White blood cell (WBC) 6.48 x10^9/L, Absolute Neutrophils Count (ANC) 2.96 x10^9/L, Liver and renal function test normal. C-reactive protein (CRP) 2.24 mg/dl down to 0.78 mg/dl

ANTIMICROBIALS & TREATMENTS

- Levofloxacin 4 December 2022 until now

Clinical scenario 28

<u>CLINICAL SCENARIO</u>

BASIC DEMOGRAPHICS & UNDERLYING MEDICAL CONDITIONS

71/M. No known drug allergies. Hypertension. Gout.

CURRENT ADMISSION

Admit 17 November 2023 x acute inferior ST-segment elevation myocardial infarction (STEMI) with cardiogenic shock. Coronary angiogram on 17 November 2023: left main (LM) mild disease, short. Left anterior descending (LAD) multiple 60-70% stenosis, left circumflex (LC) 80%, obtuse marginal (OM) 95% stenosis. Dominant right coronary artery (RCA) with thrombotic occlusion, Thrombolysis in myocardial infarction (TIMI) Flow 0. Primary percutaneous coronary intervention (PCI) to right coronary artery (RCA). Intra-aortic balloon pump (IABP) inserted 17 November 2023 however ongoing hypotension and brief episodes of asystole. Decided for elective intubation and mechanical circulatory support (MCS) Venoarterial Extracorporeal membrane oxygenation (VA-ECMO) established via right femoral artery (RFA) and RFV right femoral vein (15Fr/23Fr). Pump-controlled retrograde trial off (PCRTO) on 19 November 2023. ECMO decannulation 20 November 2023. Extubated on 21 November 2023, then HFNC due to thick sputum, then weaned to room air. Systemic inflammatory response syndrome (SIRS) while on ECMO, fever with chills and rigor, septic workup unremarkable. Subsequently developed low grade fever, polyarticular gout (bilateral knees), given 1 dose of prednisolone 20mg (24 November 2023). Groin drain removed on 23 November 2023. Plan for left sided coronary staged percutaneous coronary intervention

PHYSICAL EXAMINATION FINDINGS

Fever. But not septic looking. Room air. Chief complaint is polyarticular tophaceous gout, involving all four extremities, most severe over left knee and right hand joints; actually only bilateral shoulders and hips were spared

INVESTIGATION RESULTS

Blood culture no growth: 19 November 2023, 21 November 2023, 25 November 2023, and 27 November 2023

ANTIMICROBIALS & TREATMENTS

- IV vancomycin 19 to 22 November 2023, 26 November 2023 until now.
- IV meropenem 25 November 2023 until now,
- IV Piperacillin-tazobactam 17 to 25 November 2023

Clinical scenario 29

CLINICAL SCENARIO

BASIC DEMOGRAPHICS & UNDERLYING MEDICAL CONDITIONS

F/69. No known drug allergies. Old aged home resident. Carcinoma of right breast with right wide local excision (WLE) plus sentinel lymph node biopsy (SLNB) in December 2013, completion of right mastectomy in January 2014. Mixed anxiety-depressive disorder (MADD). External haemorrhoids. Carcinoma of pancreas diagnosed in March 2023 presented with abdominal pain with deranged liver function test with Positron Emission Tomography-Computed Tomography (PET-CT) showing carcinoma of pancreatic head with malignant biliary obstruction, no distant metastasis. Received

Whipple's operation on 10 May 2023, with computed tomography (CT) scan June 2023 showing pancreatic tail collection but reducing in size.

CURRENT ADMISSION

Admitted to Hospital R on 14 October 2023 for acute cholangitis, with CT showing dilated biliary tree with truncation at choledocho-jejunostomy site suspicious of stricture formation, with left percutaneous transhepatic biliary drainage (PTBD) inserted, and bile culture grew Escherichia coli. Transferred to Hospital Q on 18 October 2023, a spike of fever on 21 October 2023 then fever down, bile repeated from PTBD grew Enterococcus faecium, Citrobacter species, and Enterobacter cloacae complex CT on 26 October 2023 (performed in view of rising trend bilirubin): Suspicious of intestinal obstruction. Short segment of mural thickening at the transition zone with mild colonic dilatation associated with focal nodular peritoneal thickening and prominent mesenteric lymph nodes. Differential diagnosis include peritoneal carcinomatosis with extrinsic compression, inflammatory stricture or primary colonic neoplasm. Subsequent CT including pelvis commented dilated ascending colon with a persistent abrupt transition at the hepatic flexure, suspicious of malignant large bowel obstruction with nodal and peritoneal metastases. Operation (OT) on 27 October 2023 for hemicolectomy and gastrojejunostomy. Intra-operatively noted peritoneal metastasis at ascending colon causing close loop obstruction, right colon distended and ischemic looking. Peritoneal metastases at omentum causing obstruction to the gastrojejunostomy.

PHYSICAL EXAMINATION FINDINGS

Body weight 31.2 kg.

INVESTIGATION RESULTS

White blood cell (WBC) 15.04 x10^9/L, Absolute Neutrophils Count (ANC) 13.86 x10^9/L, Serum creatinine (sCr) 43 umol/L, estimated glomerular filtration rate (eGFR) > 90 ml/min/1.73 m^2, Improving liver function test after OT. Chest X-ray (CXR) on 26 October 2023 clear with no evidence of consolidation.

ANTIMICROBIALS & TREATMENTS

- Piperacillin-tazobactam 18 to 26 October 2023;
- Levofloxacin 26 October 2023 until now;
- Vancomycin 26 October 2023 until now

Clinical scenario 30

<u>CLINICAL SCENARIO</u>

BASIC DEMOGRAPHICS & UNDERLYING MEDICAL CONDITIONS

62/M. No known drug allergies. Anti-citrullinated protein antibodies (ACPA)-positive rheumatoid arthritis (RA) on sulfasalazine. Chronic hepatitis B carrier, on entecavir. History of myocarditis. Coronary artery disease with ischaemic cardiomyopathy with percutaneous coronary intervention (PCI) done 2014 and 2022, complicated with recurrent ventricular tachycardia (VT) episodes on cardiac resynchronization therapy

with a pacemaker and an Implantable Cardioverter-Defibrillator (ICD) and Cardiac resynchronization therapy with defibrillator (CRT-D).

CURRENT ADMISSION

Index admission to Hospital G (August 2023) for elective coronary angiogram and PCI, complicated by cardiogenic shock and on table arrest leading to multiorgan failure, requiring Extracorporeal membrane oxygenation (ECMO), renal replacement therapy (RRT), and respiratory support. Left ventricular assist device (LVAD) implantation (27 August 2023). CRT-D device change (9 November 2023).

Transfer back to Hospital Q 24 November 2023 due to fever and hypotension. Condition complicated by metabolic acidosis and respiratory distress 29 November 2023, intubated. Extubated now. Contrast-enhanced computed tomography (CT) abdomen and pelvis (29 November 2023): No sizable rim-enhancing collection over LVAD exit cable exit site. Previously noted left adductor intramuscular collection shows interval reduction in size, now measuring 2.8 x 1.8 x 2.8cm. A 3.9 x 1.1 x 2.4cm subcutaneous collection over the left sacral region with intralesional gas density, which appears to be connected to the overlying skin defect, could represent sacral sore. No sizable rim-enhancing intra-abdominal collection. Small amount of pelvic ascites. Irregular thickened gallbladder wall up to 0.6cm. Small amount of pericholecystic fluid and stranding. Gallbladder is not distended. No hyperdense gallstone. Overall features may represent early cholecystitis. Surgical assessment (29 and 30 November 2023): to continue antibiotics for suspected early acalculous uncomplicated cholecystitis

PHYSICAL EXAMINATION FINDINGS

Spikes of low grade temperature. Blood pressure unsupported. Nasal interface, high flow O2. Alert and conscious. Fully communicable. Reported chills yesterday, which had subsided. No rigors. Abdomen soft, non-tender. Grade 4 sacral sore as described by wound nurse. Seen by orthopaedics, no orthopaedic intervention

INVESTIGATION RESULTS

Bronchoalveolar lavage (BAL) (29 November 2023): pending. Sputum (27 November 2023): Elizabethkingia species, Sputum (26 November 2023): Stenotrophomonas maltophilia, Pseudomonas aeruginosa

ANTIMICROBIALS & TREATMENTS

- IV meropenem 30 November 2023 until now,
- IV metronidazole 30 November 2023 until now,
- IV vancomycin 25 November 2023 until now,
- PO fluconazole 25 November 2023 (plan until 9 December 2023),
- IV amoxicillin-clavulanate 25 to 30 November 2023,
- IV meropenem 23 to 25 November 2023,
- IV piperacillin-tazobactam 21 to 23 November 2023

Clinical scenario 31

<u>CLINICAL SCENARIO</u>

BASIC DEMOGRAPHICS & UNDERLYING MEDICAL CONDITIONS

61/F, no known drug allergies. Adverse drug reaction to propranolol, fenofibrate. Underlying primary biliary cholangitis with decompensated cirrhosis on list for liver transplant, hyperlipidemia, left renal angiomyolipoma

CURRENT ADMISSION

Transferred from Hospital W to Hospital Q for further management of decompensated liver cirrhosis complicated with hepatic encephalopathy, hepatorenal syndrome with AKI. Initial Glascow Coma Scale (GCS) E1V1M5 required intubation for airway protection and required continuous renal replacement therapy (CRRT) for acute kidney injury (AKI). Urine output adequate with terlipressin and albumin, subsequently not dialysis dependent, general condition improved and extubated. Left pleural pigtail inserted 24 November 2023, Total cell count (TCC) 180 x10^6/L; polymorphs 53%. Ascitic fluid cell count (20 November 2023) 187 x10^6/L (neutrophil 52%) to (3 December 2023) 71 x10^6/L (neutrophil 35%). Previous left internal jugular vein central venous catheter (CVC) noted pus, removed on 25 November 2023. Noted breakthrough fever while on piperacillin-tazobactam on 2 December 2023, septic workup repeated. Fever downtrend but noted desaturation today

PHYSICAL EXAMINATION FINDINGS

3L O2 supplementation, temperature 37.7'C at time of assessment. Blood pressure and pulse unsupported, not tachycardiac (on beta blocker for cirrhosis), chest bilateral decreased air entry, pansystolic murmur best heard at left lower sternal border, abdomen distended and soft, no lower limb edema calves soft, lines exit site not inflamed looking

INVESTIGATION RESULTS

Left pleural fluid 24 November 2023 culture no growth; IV line tip and pus culture 25 November 2023 no growth; blood and CSU culture 2 December 2023 no growth; ascitic fluid 3 December 2023 culture pending. Absolute Neutrophils Count (ANC) 10.6 x10^9/L → 7.71 x10^9/L, International normalised ratio (INR) 1.7, D-dimer 35.2 mg/L, Serum creatinine (sCr) 175 umol/L [Body weight 45kg, Creatinine Clearance by Cockcroft-Gault Equation (CrCl by C-G) = 21.2 ml/min], Total bilirubin (Bil) 511 umol/L, Alkaline phosphatase (ALP) 140 U/L, Aspartate aminotransferase (AST) 49 U/L, Ammonia 40 umol/L, Chest X-ray (CXR) 4 December 2023 noted new bilateral infiltrates

ANTIMICROBIALS & TREATMENTS

- Piperacillin-tazobactam 20 November 2023 to 2 December 2023;
- meropenem 2 December 2023 until now

Clinical scenario 32

<u>CLINICAL SCENARIO</u>

BASIC DEMOGRAPHICS & UNDERLYING MEDICAL CONDITIONS

M/82, No known drug allergies. Coronary artery disease, Atrial fibrillation on Novel Oral Anticoagulants (NOAC). Lower urinary tract symptoms with a history of raised prostate specific antigen (PSA) and biopsy done twice showing no malignancy.

CURRENT ADMISSION
Admitted 11 to 21 November 2023 to Hospital Y for Toxic epidermal necrolysis (TEN), previously attended accident and emergency department or outpatient clinic for upper respiratory tract infection (URTI) symptoms, given amoxicillin-clavulanate and panadeine (panadol plus codeine), developed rash over face and scalp on 6 November 2023, with mucosal eruptions over eyes, lips, and mouth. Started on intravenous immunoglobulin (IVIG) on 20 November 2023 and 22 November 2023. Previously tolerating ciprofloxacin, amoxicillin, and azithromycin. Overall body surface area (BSA, 80% with diffuse erythema with hemorrhagic crusts over lips, perioral and periorbital area. Dusky targetoid patches over neck and chest, thighs. Epidermal detachment over abdomen, axillae, inner thighs, buttocks, most areas over back.
PHYSICAL EXAMINATION FINDINGS
Patient afebrile.
INVESTIGATION RESULTS
White blood cell (WBC) 10.23 x10^9/L, Absolute Neutrophils Count (ANC) 8.39 x10^9/L, Serum creatinine (sCr) 81 umol/L, estimated glomerular filtration rate (eGFR) 62 ml/min/1.73 m^2. Worsening Alanine aminotransferase (ALT). Chest X-ray (CXR) 21 November 2023 no evidence of consolidation. Blood culture on 15 November 2023 grew Methicillin-resistant Staphylococcus aureus (MRSA), no repeated blood culture for documented clearance. Wound swab on 15 November 2023 grew MRSA, Peptoniphilus species, Elizabethkingia miricola. Eye swab on 15 November 2023 grew MRSA, Chryseobacterium arthrosphaerae, Eikenella corrodens. Already taken blood culture today. Wound swab taken pending further incubation.
ANTIMICROBIALS & TREATMENTS
- Levofloxacin 16-17 November 2023;
- Ciprofloxacin 21 November 2023 until now;
- Vancomycin 17 November 2023 until now

Clinical scenario 33

CLINICAL SCENARIO
BASIC DEMOGRAPHICS & UNDERLYING MEDICAL CONDITIONS
40/M, No known drug allergies. From New Zealand, International school teacher, keeping 2 adopted dogs at home since 9 months ago (Poodle and cockapoo). Underlying hypertension and hyperlipidemia, past history of autoimmune hemolytic anemia in 1998 with splenectomy done and follow-up in New Zealand. Received Pneumovax 23 [Pneumococcal Polysaccharide Vaccine (PPSV23)] in 2019, Bexsero (meningococcal group B vaccine) (1) and MenACWY Vaccine (Meningococcal group A, C, W-135 and Y conjugate vaccine) in 2022. Not on any antimicrobial; also history of encephalitis in 2004 and trauma related R tibia fracture and deep vein thrombosis.
CURRENT ADMISSION
Admitted for fever. Recent travelled to Bangkok 9 to 12 November 2023 with no raw food intake, rural area visits, high risk sexual activities. Fever with chills, myalgia,

malaise since 21 November 2023 with bifrontal headache and mild neck stiffness. No chest pain, shortness of breath, joint pain noted. Lumbar puncture done today.

PHYSICAL EXAMINATION FINDINGS

Similar headache and neck stiffness. Fever down, stable, room air. Chest clear, heart sound with no murmur, abdomen soft, neurology unremarkable, drip site not inflamed looking, calves soft.

INVESTIGATION RESULTS

Blood culture 21 November 2023 Streptococcus pneumoniae [Penicllin minimum inhibitory concentration (MIC) 0.38 ug/ml], 3 sets blood culture 22 November 2023 pending; sputum urine culture 21 November 2023 pending. Absolute Neutrophils Count (ANC) 14.91 x10^9/L down to 5.62 x10^9/L, Absolute Lymphocyte Count (ALC) 4.72 x10^9/L, C-reactive protein (CRP) 12.91 mg/dl, Serum creatinine (sCr) 73 umol/L, Liver function test unremarkable, Chest X-ray (CXR) no obvious consolidation

ANTIMICROBIALS & TREATMENTS

- Piperacillin-tazobactam 21 November 2023;
- ceftriaxone 21 November 2023 until now;
- doxycycline 21 to 23 November 2023

Clinical scenario 34

<u>CLINICAL SCENARIO</u>

BASIC DEMOGRAPHICS & UNDERLYING MEDICAL CONDITIONS

M/59. No known drug allergies. Works as a hawker, sells fish. Smoker, heavy drinker. Hypertension. Minor coronary artery disease.

CURRENT ADMISSION

Admitted on 17 November 2023 for tenosynovitis, presented with increasing swelling over second metacarpophalangeal joint (MCP) dorsum for 2 months. Seen by a general practitioner, given 1 week of Augmentin with no improvement. Magnetic resonance imaging (MRI) hand with contrast on 2 November 2023: synovial thickening at multiple sites of right hand and wrist, suggestive of infective cause vs inflammatory arthropathy. Synovectomy of wrist and tenonectomy of hand on 20 November 2023 at Hospital D, intraoperatively noted extensor compartments with significant synovitis, with little finger significant tenosynovitis. Yellowish fluid seen within tendon sheath. Bacterial culture grew Methicillin-sensitive Staphylococcus aureus (MSSA) by broth culture. Synovectomy at Hospital D 27 November 2023 mild residual tenosynovitis along third to fifth compartment. Flexor side moderate tenosynovitis along flexor pollicis longus (FPL), flexor digitorum profundus (FDP) & flexor digitorum superficialis (FDS). Significant synovitis along whole index finger towards radial bursa.

INVESTIGATION RESULTS

White blood cell (WBC) 8.50 x10^9/L, Absolute Neutrophils Count (ANC) 5.62 x10^9/L, Renal function normal. Deranged liver function. Histology of both intraoperative specimens pending. Acid fast bacilli (AFB) culture pending.
ANTIMICROBIALS & TREATMENTS
• Amoxicillin-clavulanate 17 November 2023 until now.

Clinical scenario 35

<u>CLINICAL SCENARIO</u>
BASIC DEMOGRAPHICS & UNDERLYING MEDICAL CONDITIONS
F/83. No known drug allergies. Osteoporosis. History of spinal stenosis L4/5 with Exploration and decompression of spinal canal February 2009. Right osteoarthritis of hip with total hip replacement in Hospital B June 2017. Hypertension. Impaired fasting glucose.
CURRENT ADMISSION
Left medial thigh sarcoma post wide excision and vascular reconstruction 10 October 2023. Noted persistent unhealthy, purulent wound bed during round. Transferred back on 6 November 2023 for urgent computed tomography (CT) scan. Fluid collection 8.6x6.7x14.1cm at anterior aspect of upper thigh with rim-enhancement and gas locule. Intra-operatively noted 8x3cm patch in skin necrosis, 300 ml turbid fluid, medial edge of rectus femoris unhealthy and friable. Vascular graft assessed by vascular team as proximal graft noted inside collection on CT, strongly pulsatile, proceed with muscular flap coverage.
PHYSICAL EXAMINATION FINDINGS
Afebrile. Blood pressure and pulse unsupported, on room air. Not septic looking. Drip site clean. Foley bag urine clear no sediment. Drains noted over the left thigh wound. Patient nil active complaint. No skin rash noted.
INVESTIGATION RESULTS
Haemoglobin (Hb) 8.3 g/dL, White blood cell (WBC) 5.58 x10^9/L, Platelet (Plt) 393 x10^9/L, Serum creatinine (sCr) 140 umol/L, Alkaline phosphatase (ALP) 107 U/L, Alanine aminotransferase (ALT) 7 U/L, Creatinine Clearance by Cockcroft-Gault Equation (CrCl by C-G) = 20.9 ml/min, Blood culture (6 November 2023) pending. Tissue culture (7 November 2023) Enterobacter cloacae complex, Klebsiella pneumoniae, Proteus mirabilis. Wound swab/Aspirate culture (7 November 2023) Enterobacter cloacae complex, Morganella morganni, Klebsiella pneumoniae, Proteus mirabilis. Fluid culture (7 November 2023) Morganella morganni, Klebsiella pneumoniae, Proteus mirabilis, Enterococcus faecalis
ANTIMICROBIALS & TREATMENTS
• Cefazolin 7 November 2023
• Piperacillin-tazobactam 7 November 2023 until now

Clinical scenario 36

<u>CLINICAL SCENARIO</u>

BASIC DEMOGRAPHICS & UNDERLYING MEDICAL CONDITIONS

75/M. No known drug allergies. Alcoholic liver cirrhosis. Hepatic encephalopathy. Esophageal varices, recurrent bleeding. Hepatorenal syndrome (not renal replacement therapy candidate). Spontaneous bacterial peritonitis. Ascites requiring tapping every 2 weeks. Diuretic resistant ascites (off aldactone). Not for transjugular intrahepatic portosystemic shunt (TIPS) due to the history of hepatic encephalopathy. Keep carvedilol for the history of esophageal varices bleeding. Hepatitis B surface antigen (HBs Ag) Negative. Hepatitis B surface antibody (HBs Ab) Positive (11-100 mIU/mL). Hepatitis B core antibody (HBc Ab) positive. Hepatitis C antibody (HCV Ab) negative. Diabetes mellitus

CURRENT ADMISSION

Admit 19 November 2023 x hepatic encephalopathy and coffee ground vomiting. Intubated in ICU same day. 19 November 2023 Oesophago-Gastro-Duodenoscopy (OGD): Two small post esophageal varices banding ulcer without active bleeding. The esophageal varices above were Forrest 1 only (minimally elevated veins above esophageal mucosal surface). Complete obliteration of the esophageal varices on air insufflation was noted. Decided not for further hemostasis. Fr 12 feeding tube inserted under direct vision avoiding the two areas of post banding ulcer. Extubated (20 November 2023). Added rifaximin.

PHYSICAL EXAMINATION FINDINGS

Afebrile. Abdomen distended. Non-tender on palpation. No loin pain. No dysuria.

INVESTIGATION RESULTS

Catheter Specimen Urine (CSU; 27 November 2023): Extended-Spectrum Beta-Lactamases (ESBL)-producing Klebsiella pneumoniae. Renal function test fluctuating around baseline

Clinical scenario 37

<u>CLINICAL SCENARIO</u>

BASIC DEMOGRAPHICS & UNDERLYING MEDICAL CONDITIONS

60/F. No known drug allergies. Non-smoker, Non-drinker. Freelance worker (food designer). Previously good past health. Single, lives with elderly father (on foley catheter); mother lives in old aged home. Admit Hospital R 1 to 2 December 2023 for acute liver failure. 2 weeks of epigastric and abdominal discomfort with reduced appetite. Seen General practitioner, followed by Traditional Chinese Medicine doctor. Patient's father last seen patient well on Wednesday (29 November 2023). No response from patient over the following 2 days. Found collapsed in room (1 December 2023). Traditional chinese medicine reviewed by Poison information center (PIC). Less likely to be related to Traditional chinese medicine due to short duration of use and symptom onset prior to traditional Chinese medicine use. Elective intubation. Coagulopathy correction by fresh frozen plasma and vitamin K. Left femoral vein Central Venous Catheters (CVC) insertion.

CURRENT ADMISSION
Transferred Hospital Q 2 December 2023. Cytomegalovirus antibody IgG Positive (immune); Consistent with past CMV infection. Hepatitis B surface antigen (HBs Ag) Negative, Hepatitis B surface antibody (HBs Ab) Positive (>100 mIU/ml HepB, immune); Hepatitis B core antibody (HBc Ab; total) Positive. Hepatitis C antibody (HCV Ab) negative. Human immunodeficiency virus (HIV) combined serology negative. Liver transplant team seen, no contraindication for liver transplantation
PHYSICAL EXAMINATION FINDINGS
Kick of fever. Non-responsive (Ammonia 118 umol/L). Clinically no stigmata of chronic liver disease). Abdomen soft, non-tender. No skin rash. No cervical lymphadenopathy.

Clinical scenario 38

CLINICAL SCENARIO
BASIC DEMOGRAPHICS & UNDERLYING MEDICAL CONDITIONS
M/54, No known drug allergies. Metastatic carcinoma of gallbladder with metastasis to distal lymph nodes, peritoneal disease, segment 8 and 4 liver tumor, multiple regional and distal left neck lymph nodes. Recent admission in September 2023 for long stricture at common hepatic duct (CHD) with endoscopic retrograde cholangiopancreatography (ERCP) and stenting done, biliary sepsis treated with a course of Meropenem. Last admitted for acute cholangitis with raised bilirubin, jaundice and tea colored urine. Right upper quadrant tenderness as well with fever. ERCP performed noted tight hilar stricture progressed, stent to left duct and right posterior sectoral duct (RPSD). Computed tomography (CT) (28 September 2023) shows dilated left and right intrahepatic duct, possibility of stent occlusion given persistent dilated left intrahepatic ducts. Right internal-external percutaneous transhepatic biliary drainage (PTBD) performed on 7 October 2023. Bile culture grew Pseudomonas aeruginosa resistant to imipenem given a course of Ceftolozane-tazobactam and discharged
CURRENT ADMISSION
Readmitted on 25 October 2023 for increased abdominal distension, pain and fever. Ultrasound abdomen performed on 26 October 2023 shows liver parenchymal disease, septated gallbladder with wall thickening, pancreatic cystic lesion and left renal cyst. ERCP performed on 27 October 2023, old pigtail stents removed, 2 stents inserted into left and right anterior hepatic ducts.
PHYSICAL EXAMINATION FINDINGS
Fever 38.3'C. Blood pressure and pulse unsupported. on room air. Not septic looking. Abdomen tenderness over the middle area and lower quadrants. PTBD output positive. Calves are soft. No lower limb edema.
INVESTIGATION RESULTS
Haemoglobin (Hb) 9.3 g/dL, White blood cell (WBC) 6.27 x10^9/L, Platelet (Plt) 358 x10^9/L, Serum creatinine (sCr) 45 umol/L, Bilirubin (Bil) 33 umol/L, Alkaline

phosphatase (ALP) 373 U/L, Alanine aminotransferase (ALT) 20 U/L, Sputum culture (25 October 2023) oral commensals, Urine culture (25 October 2023) no growth, Ascitic fluid culture (25 October 2023) Enterococcus faecium, Staphylococcus haemolyticus, Bile culture (25 October 2023) Stenotrophomonas maltophilia, Klebsiella pneumoniae, Enterococcus faecium, Escherichia coli. Blood culture (25 October 2023) pending

ANTIMICROBIALS & TREATMENTS

- Ceftolozane-tazobactam 25 October 2023 until now

Clinical scenario 39

CLINICAL SCENARIO

BASIC DEMOGRAPHICS & UNDERLYING MEDICAL CONDITIONS

M/61. No known drug allergies. Ex-smoker, non-drinker.

CURRENT ADMISSION

Pulmonary tuberculosis (TB) diagnosed in China with pleural fluid Mycobacterium tuberculosis (TB) complex DNA polymerase chain reaction (PCR) detected, with sputum grew M. tuberculosis (sensitive to isoniazid, rifampicin, pyrazinamide, ethambutol), transferred to Hospital N for management, started on isoniazid, rifampicin, pyrazinamide, and ethambutol (HRZE) since 23 September 2023, and piperacillin-tazobactam since 24 September 2023. Started to develop diffuse maculopapular rash over face, trunk, and 4 limbs since initiation of tuberculosis medication, with derangement of liver function test since 29 October 2023. Blood test showed eosinophilia with atypical lymphocytosis. Hepatitis marker unremarkable. Ultrasound of the hepatobiliary system showed hepatosplenomegaly, with diffusely thickened gallbladder wall. Computed tomography (CT) of the thorax, abdomen and pelvis showing large lung abscess with air-fluid level at left lower lung, with small cavitating lesions and centrilobular nodules in both lungs. Bilateral pleural effusion. Seen by dermatology in Hospital TM, commented as Drug Reaction with Eosinophilia and Systemic Symptoms (DRESS), started on hydrocortisone since 3 November 2023 (100 mg IV every 8 hours until now). All anti-tuberculosis medication stopped on admission, temporarily switched to amikacin, levofloxacin, and ethambutol 2 to 6 November 2023, then stopped again.

Transferred to Hospital Q for liver transplantation workup on 10 November 2023. Currently still on hydrocortisone 100mg IV every 8 hours.

PHYSICAL EXAMINATION FINDINGS

Body weight around 63.8kg. Vitals stable, afebrile. Tired looking. Jaundice. Chest clear, with occasional crepitations. Heart sound dual no murmur. Abdomen soft, non-tender. Drip site well. Generalized erythema with no vesicles seen and no mucosal involvement. Bilateral pitting edema.

INVESTIGATION RESULTS

White blood cell (WBC) 21.18 x10^9/L. Absolute Neutrophils Count (ANC) 15.12 x10^9/L. Absolute Lymphocyte Count (ALC) 3.47 x10^9/L. Platelet (Plt) 227 x10^9/L.

Eosinophil 0.01 x10^9/L. Serum creatinine (sCr) 85 umol/L. estimated glomerular filtration rate (eGFR) 84 ml/min/1.73 m^2. Bilirubin (Bil) 385 umol/L. Alkaline phosphatase (ALP)105 U/L. Alanine aminotransferase (ALT) 911 U/L. Aspartate aminotransferase (AST) 282 U/L. Fasting glucose 6.1 mmol/L. Urine on 1 November 2023 grew Extended-Spectrum Beta-Lactamases (ESBL)-producing Escherichia coli. Last blood culture on 1 November 2023 no growth

ANTIMICROBIALS & TREATMENTS

- Amoxicillin-clavulanate 1 to 3 November 2023;
- Piperacillin-tazobactam 7 to 9 November 2023;
- Meropenem 9 November 2023 until now.

Clinical scenario 40

<u>CLINICAL SCENARIO</u>

BASIC DEMOGRAPHICS & UNDERLYING MEDICAL CONDITIONS

16/M. No known drug allergies. G6PD deficiency. Lives with parents and younger brother, and domestic helper. Secondary school student (year 11).

CURRENT ADMISSION

Refer from Private Hospital 30 November 2023 for Dengue fever. School trip from 19 to 24 November 2023 to Thailand; hiking and mosquito bites positive. Fever onset on 29 November 2023. Associated with malaise, rhinorrhoea. Private blood test Dengue virus NS1 Antigen Positive, IgM and IgG Negative. Nasopharyngeal swabs (NPS) on 30 November 2023: Coronary virus OC43 RNA detected.

Day 5 post-symptom onset. Tired looking. Sore throat and dysphagia. Subjective improved today. No other focal complaints.

PHYSICAL EXAMINATION FINDINGS

Noted scattered petechiae over bilateral lower limbs. Right medial forearm also have a few petechiae. No other skin rash. Left sided cervical lymphadenopathy, non-tender on palpation. Abdomen soft, non-tender. No joint pain.

Artificial intelligence (AI)-generated outputs

1. Clinical scenario 1

GPT-4.0

1. Chronological Events:
Year	Clinical Event
2006	Diagnosed with pulmonary tuberculosis (TB), bilateral pleural effusion, positive M. tuberculosis PCR.
2007	Pelvic abscess, CT-guided aspiration, AFB smear positive, culture negative; Laparotomy and drainage, anti-TB treatment till July 2008.
2010	Diagnosed with Systemic Lupus Erythematosus (SLE), class III+V lupus nephritis.
2011	Started treatment with prednisolone and azathioprine.
2012	Stopped azathioprine due to leukopenia.
2020	Relapsed lupus nephritis, started on hydroxychloroquine, mycophenolate mofetil, and continued prednisolone.
2023	Admitted for fever (since 18 November).

2. Clinical Problem List:
Status	Clinical Issue
Active	Fever since 18 November 2023
Active	Mild right chest wall pain
Chronic	Hypertension
Chronic	Crouzon's syndrome
Chronic	Apert's syndrome
Chronic	Mental retardation
Chronic	Hearing impairment
Chronic	History of pulmonary tuberculosis (TB)
Chronic	History of pelvic abscess
Chronic	Systemic Lupus Erythematosus (SLE) with lupus nephritis

3. Potential Life-Threatening Complications:
- Sepsis from unresolved infection
- Acute exacerbation of lupus nephritis leading to renal failure
- Pancytopenia leading to severe infections or bleeding

4. Clinical Findings:
Organ System	Anticipated Findings
General	Swinging low grade fever
Cardiovascular	Blood pressure and pulse unsupported, on room air

Lymphatic	No palpable cervical/groin lymphadenopathy
Respiratory	Chest clear
Gastrointestinal	Abdomen soft non-tender, no change in bowel habit
Musculoskeletal	Calves soft, no lower limb edema
Skin	Redness over left lateral finger, vasculitic skin rash

5. Working Diagnoses:
- Infection (etiology pending investigation results: fever, CRP elevated)
- Lupus flare (based on history of SLE with lupus nephritis and vasculitic skin rash)
- Potential drug-related effects (pancytopenia with history of medication adjustments)

6. Relevant Investigations:

Investigation	Justification
Complete Blood Count (CBC)	To monitor known pancytopenia and detect any changes that could indicate infection or lupus flare
Blood cultures	To identify causative organism of the fever
Urine Analysis	To assess renal function in the context of lupus nephritis
Renal Function Tests (RFTs)	To monitor known lupus nephritis and assess current renal function
Autoimmune Serology (ANA, anti-dsDNA, C3, C4)	To assess SLE activity
Procalcitonin	To differentiate between bacterial infection and other causes of fever

7. Management Plan:
a. Continue broad-spectrum antibiotics pending microbiological results.
b. Monitor vital signs and blood counts closely.
c. Adjust immunosuppressive therapy if lupus flare is confirmed.
d. Supportive care for symptoms, including pain management.
e. Consider imaging studies if there is no improvement or if any localizing symptoms/signs develop.
f. Patient education on signs of worsening infection or lupus flare.

8. Executive Summary:
The patient, a 56-year-old female with a complex medical history including SLE with lupus nephritis, hypertension, and a history of TB, is currently admitted with fever and chest pain. Immediate concerns include ruling out sepsis, monitoring for lupus flare, and managing pancytopenia. The patient is on broad-spectrum antibiotics and further investigations are pending. A careful balance of immunosuppression is necessary to control SLE without increasing the risk of infection. Close monitoring and patient education are essential components of the management strategy.

cGPT-4

1. Chronological Events:

Year	Clinical Issue
2006	Pulmonary tuberculosis (TB) with bilateral pleural effusion, positive for Mycobacterium tuberculosis complex DNA PCR
2007	Noted pelvic abscess, CT-guided aspiration and drainage; fluid AFB smear positive, culture negative; laparotomy and drainage
2008	Completed anti-TB treatment
2010	Diagnosis of Systemic Lupus Erythematosus (SLE) with Class III+V lupus nephritis
2011	Started treatment with prednisolone and azathioprine
2012	Stopped azathioprine due to leukopenia
2020	Relapse of lupus nephritis; started treatment with hydroxychloroquine, mycophenolate mofetil, and prednisolone
2023	Current admission for fever

2. Clinical Problem List:

Status	Clinical Issue
Chronic	Crouzon's syndrome and Apert's syndrome
Chronic	Mental retardation
Chronic	Hearing impairment
Chronic	Hypertension
Chronic	Systemic Lupus Erythematosus (SLE) with lupus nephritis
Active	Fever since 18 November 2023
Active	Mild right chest wall pain

3. Potential Life-Threatening Complications:
- Severe infection due to immunosuppressive therapy for SLE
- Sepsis or bacteremia, considering the current fever and past history of TB and pelvic abscess
- Lupus flare with potential for major organ involvement

4. Clinical Findings:

Organ System	Anticipated Findings
General	Swinging low-grade fever
ENT	Hearing impairment; history of left parotid swelling
Respiratory	Clear chest currently; history of TB
Cardiovascular	Hypertension
Musculoskeletal	Redness over left lateral finger; non-tender movement
Renal	History of lupus nephritis; may present with proteinuria or renal impairment
Immune	Signs of SLE activity, such as rash or vasculitis

5. Working Diagnoses:
- Fever of unknown origin, possibly related to infection or lupus flare
- SLE activity, given the history of lupus nephritis and pancytopenia
- Possible recurrent or new infection, considering the immunosuppressed state

6. Relevant Investigations:

Investigation	Justification
Blood culture	To identify causative agent for fever and rule out bacteremia/sepsis
C-reactive protein (CRP)	To assess inflammation which may indicate infection or lupus activity
Procalcitonin	To differentiate between bacterial infection and other causes of fever
Urinalysis	To monitor for lupus nephritis activity, detect proteinuria or hematuria
Complement levels (C3, C4)	To evaluate for lupus activity, as low levels can be associated with flares
Anti-double-stranded DNA antibodies	To assess for lupus activity, as these antibodies can increase during a flare
Chest X-ray	To rule out respiratory infections or complications, given the history of TB

7. Management Plan:
- Continue empirical antibiotic therapy with piperacillin-tazobactam and doxycycline until the causative agent and source of fever are identified.
- Monitor vital signs closely for signs of sepsis.
- Adjust immunosuppressive therapy for SLE as needed based on disease activity and risk of infection.
- Provide supportive care for fever and any other symptoms.
- Follow up on pending investigations, including blood cultures, CRP, procalcitonin, and complement levels, to guide further management.
- Consider consultation with rheumatology to manage SLE and nephrology for kidney involvement.

8. Executive Summary:
The patient, a 56-year-old female with a complex medical history including SLE and a past history of TB, is currently admitted with an unexplained fever. Management includes broad-spectrum antibiotics while awaiting culture and inflammatory marker results. Given her immunosuppressed state, close monitoring for signs of severe infection is crucial, along with potential adjustment of her immunosuppressive regimen. Collaboration with rheumatology and nephrology specialists is recommended to address her SLE and renal involvement.

Gemini Pro

1. Chronological Events:

Date	Event
July 2006	Pulmonary tuberculosis (TB) diagnosed, bilateral pleural effusion noted.
March 2007	Pelvic abscess noted, CT-guided aspiration and drainage performed, AFB smear positive but culture negative.
July 2007	Laparotomy and drainage for pelvic abscess, peritoneal fluid AFB smear and culture negative.
July 2008	Anti-TB treatment completed.
2010	Systemic Lupus Erythematosus (SLE) diagnosed, presenting with fever, vasculitic skin rash, pancytopenia, and active serology.
March 2011	Renal biopsy performed, showing combined membranous and focal proliferative lupus nephritis. Azathioprine added to prednisolone treatment.
2012	Azathioprine discontinued due to leukopenia.
March 2020	Lupus nephritis relapse, increased urine protein noted.
November 18, 2023	Patient admitted for fever of unknown origin, mild right chest wall pain, no localizing symptoms.
December 1, 2023	Piperacillin-tazobactam and doxycycline initiated.
December 4, 2023	Blood culture drawn.

2. Clinical Problem List:

Active	Chronic
Fever of unknown origin	Hypertension
Right chest wall pain	Crouzon's syndrome
Lupus nephritis	Apert's syndrome
Mental retardation	Hearing impairment
Pancytopenia	Pulmonary tuberculosis
Pelvic abscess	SLE

3. Potential Life-Threatening Complications:
* Sepsis
* Multi-organ failure
* End-stage renal disease
* Lupus cerebritis
* Pulmonary embolism

4. Clinical Findings:

Organ System	Findings
General	Swinging low-grade fever
Respiratory	Mild right chest wall pain, clear lungs
Cardiovascular	Blood pressure and pulse unsupported on room air

Gastrointestinal	Soft, non-tender abdomen
Musculoskeletal	Redness over left lateral finger, non-tender on movement
Integumentary	No rash

5. Working Diagnoses:
* Sepsis of unknown origin
* Lupus nephritis
* Pulmonary tuberculosis

6. Relevant Investigations:

Investigation	Justification
Blood culture	To identify the causative organism of sepsis.
Urine analysis	To assess renal function and monitor lupus nephritis activity.
Chest X-ray	To evaluate the extent of pulmonary tuberculosis and monitor for complications.
Sputum AFB smear and culture	To confirm the diagnosis of pulmonary tuberculosis.
Anti-nuclear antibody (ANA) titer	To assess SLE activity.
Anti-double-stranded DNA (anti-dsDNA) antibodies	To assess SLE activity.
Complement levels (C3, C4)	To assess SLE activity.

7. Management Plan:
* Administer broad-spectrum antibiotics to cover potential pathogens causing sepsis.
* Monitor for signs and symptoms of sepsis and organ dysfunction.
* Perform source control measures to identify and treat the underlying cause of sepsis.
* Continue treatment for lupus nephritis with hydroxychloroquine, mycophenolate mofetil, and prednisolone.
* Monitor renal function and adjust medications as needed.
* Perform regular chest X-rays to monitor the extent of pulmonary tuberculosis and for complications.
* Continue anti-tuberculosis therapy as prescribed.

8. Executive Summary:
A 56-year-old female with a history of hypertension, Crouzon's syndrome, Apert's syndrome, mental retardation, hearing impairment, pulmonary tuberculosis, SLE, and lupus nephritis presented with fever of unknown origin and mild right chest wall pain. She is currently being treated with piperacillin-tazobactam and doxycycline for sepsis of unknown origin. Further investigations are needed to identify the underlying cause of sepsis. The patient's lupus nephritis is being managed with hydroxychloroquine, mycophenolate mofetil, and prednisolone. She is also receiving anti-tuberculosis therapy. Close monitoring and management of complications are essential.

Claude 2

1. Chronological Events

Date	Event
2006	Diagnosed with pulmonary TB
2007	Pelvic abscess drainage
2010	Diagnosed with SLE
2011	Treated for SLE with prednisolone and azathioprine
2012	Stopped azathioprine due to leukopenia
2020	Relapsed lupus nephritis
Nov 2023	Admitted for fever

2. Clinical Problem List

Problem	Status
SLE with lupus nephritis	Active
History of pulmonary TB	Chronic
History of pelvic abscess	Chronic
Fever of unknown origin	Active

3. Potential Life-Threatening Complications
- Sepsis/septic shock from undiagnosed infection
- Acute kidney injury from active lupus nephritis
- Disease flare from immunosuppression

4. Clinical Findings

Organ System	Expected Findings
Constitutional	Fever, malaise
HEENT	Oral ulcers
Respiratory	Clear on auscultation
Cardiovascular	Tachycardia
Abdominal	Normal bowel sounds
Extremities	Non-blanching rash, arthritis

5. Working Diagnoses
- SLE flare
- Infection (e.g. UTI, pneumonitis)
- Medication reaction
- Malignancy

6. Relevant Investigations

Investigation	Rationale

Urinalysis	Assess for UTI
Blood cultures	Diagnose bacteremia
Chest X-ray	Evaluate for pneumonia
CT abdomen/pelvis	Assess for intra-abdominal infection
ANA, dsDNA	Confirm SLE flare
C3, C4	Assess immune complex disease
SPEP	Screen for multiple myeloma

7. Management Plan
- Admit patient for close monitoring
- Start empiric broad spectrum antibiotics after cultures obtained to cover nosocomial pathogens
- Continue hydroxychloroquine and prednisone, hold MMF pending workup
- IV fluids for hydration
- Monitor for complications of SLE flare e.g. nephritis, cytopenias
- Further imaging and biopsies as needed based on clinical course

8. Executive Summary
This 56F with history of SLE and TB presented with fever concerning for infection vs SLE flare. Empiric antibiotics should be started while evaluating for source with cultures, imaging, and serologies. Immunosuppression should be tailored to clinical status. Close monitoring is needed to quickly identify and treat any life-threatening complications.

2. Clinical scenario 2

GPT-4.0

1. Chronological Events:
Year/Month	Event
2017	Diagnosed with carcinoma of the stomach, operation performed
May 2023	PET-CT showed mild diffuse increased FDG uptake in gastric mucosa and healing fracture at left anterior fifth rib
November 2023	MRI showed irregular thick-walled lesion within left paraspinal musculature from L2 to L4 with bony erosion
January 2024	Presented with progressive low back pain, loss of weight, and pending CT-guided biopsy

2. Clinical Problem List:
Status	Clinical Issues
-	
Active	Progressive low back pain
Active	Weight loss

Active	Lesion in left paraspinal musculature with bony erosion
Chronic	Carcinoma of stomach (currently in remission)
Chronic	Post-operative state of stomach carcinoma surgery
Chronic	History of healing rib fracture

3. Potential Life-Threatening Complications:
- Paraspinal muscle lesion with bony erosion could indicate invasive malignancy or infection leading to spinal instability or neurological compromise.
- Unintended weight loss may signify cancer recurrence or metastasis.
- Elevated CRP and ESR could be associated with a severe inflammatory or infectious process.

4. Clinical Findings:
Organ System	Anticipated Findings
-	
Musculoskeletal	Localized tenderness, possible deformity or abnormal movement in lumbar spine region
Neurological	Decreased motor strength in lower limbs (proximal power four plus out of five)
General	Signs of weight loss such as cachexia

5. Working Diagnoses:
- Paraspinal abscess or neoplasm with potential spinal involvement.
- Metastatic disease or recurrence of gastric carcinoma.
- Possible infection given the elevated inflammatory markers (CRP, ESR).

6. Relevant Investigations:
Investigation	Justification
CT-guided biopsy of lesion	To determine the nature of the lesion (infectious, neoplastic)
Sputum culture	To rule out respiratory infection given the history of non-respiratory symptoms
Urine routine microscopy	To exclude urinary tract infection or renal pathology as a cause of back pain or systemic symptoms
Serum tumor markers	To evaluate for possible cancer recurrence or metastasis
Lumbosacral spine X-ray	To further assess known collapse at L1 and potential progression

7. Management Plan:

- Immediate: Admit for pain management and close monitoring of neurological status.
- Diagnostic: Prioritize CT-guided biopsy to characterize the lesion. Ensure the processing of sputum culture and urine microscopy.

- Supportive: Nutritional support considering weight loss and potential cachexia.
- Preventive: Monitor for signs of infection, and prepare for antimicrobial therapy based on culture results.
- Follow-up: Schedule regular follow-up for chronic management of stomach carcinoma remission and overall health.

8. Executive Summary:

The patient is a 63-year-old male with a history of stomach carcinoma in remission, currently presenting with progressive low back pain and unintentional weight loss. Imaging reveals a concerning lesion in the left paraspinal musculature with bony erosion. Immediate management should focus on pain control and obtaining a biopsy to guide further treatment. The elevated inflammatory markers and history suggest a differential including infection or malignancy. A comprehensive management plan will address both diagnostic evaluation and supportive measures while preparing for potential complications related to the identified clinical issues.

cGPT-4

1. Chronological Events:

Date	Event
2017	Carcinoma of stomach diagnosed; operation performed
May 2023	PET-CT shows mild diffuse increased FDG uptake in gastric mucosa and healing fracture at left anterior fifth rib
Nov 22, 2023	MRI shows irregular lesion in left paraspinal musculature with bony erosion
Current Admission	Presented with progressive low back pain and weight loss; awaiting CT-guided biopsy

2. Clinical Problem List:

Status	Clinical Issue
Active	Progressive low back pain
Active	Weight loss
Chronic	History of carcinoma of stomach (currently in remission)
Chronic	Allergy to ampicillin and cloxacillin
Chronic	Healing fracture at left anterior fifth rib

3. Potential Life-Threatening Complications:
- Spinal cord compression due to paraspinal mass lesion
- Metastatic disease or local recurrence of previously diagnosed gastric carcinoma

4. Clinical Findings:

Organ System	Anticipated Findings
Musculoskeletal	Tenderness and possible deformity in the lumbar region
Neurological	Bilateral lower limb proximal muscle weakness

5. Working Diagnoses:
- Paraspinal abscess or neoplasm with possible spinal involvement
- Metastatic carcinoma
- Tuberculosis (despite no known history or contact)

6. Relevant Investigations:

Investigation	Justification
CT-guided biopsy	To obtain a definitive diagnosis of the paraspinal lesion
Complete blood count with differential	To evaluate for infection or malignancy (ANC and ALC already known)
Sputum culture	To rule out tuberculosis or other respiratory infections
Urine routine microscopy	To check for urinary system involvement

7. Management Plan:
- Arrange for the CT-guided biopsy as soon as possible to determine the nature of the paraspinal lesion.
- Start a broad-spectrum antibiotic after discussing with the patient and considering his allergy to ampicillin and cloxacillin
- The first dose of antibiotics should be administered by a professional capable of managing anaphylaxis, and the patient should be monitored closely for any adverse reactions
- Daily review by a nurse or similar healthcare professional, with regular blood tests to monitor for signs of infection or toxicity from the medications
- If OPAT (Outpatient Parenteral Antimicrobial Therapy) is considered, ensure that the team includes a medical lead, an infection specialist, an antimicrobial pharmacist, and a specialist nurse
- The patient's management plan should be agreed upon between the OPAT service and the referring specialty, with defined lines of responsibility and systems for urgent assessment or readmission if required

8. Executive Summary:
The patient, a 63-year-old man with a history of gastric carcinoma, presents with low back pain and weight loss. Imaging suggests a paraspinal lesion with spinal involvement. A CT-guided biopsy is pending to determine the nature of the lesion. Management should include broad-spectrum antibiotics, daily clinical reviews, and

multidisciplinary team oversight. The potential for OPAT should be assessed in line with national guidelines and the patient's specific needs.

Gemini Pro

1. Chronological Events:

Date	Event
2017	Carcinoma of stomach diagnosed and surgical resection performed
May 2023	PET-CT scan reveals mild diffuse increased FDG uptake in the gastric mucosa and focal increased FDG uptake with sclerosis and callus formation at the left anterior fifth rib, suggestive of healing fracture
November 22, 2023	Private MRI scan demonstrates an irregular, thick-walled, peripherally enhancing cystic or necrotic lesion within the left paraspinal musculature, extending from L2 to L4 with bony erosion and infiltration
Current	Patient presents with progressive low back pain, weight loss, and no associated respiratory, bowel, urinary, or neurological symptoms

2. Clinical Problem List:

Active	Chronic
Low back pain	Carcinoma of stomach (in remission)
Weight loss	

3. Potential Life-Threatening Complications:

- Spinal cord compression
- Sepsis
- Multi-organ failure

4. Clinical Findings:

Organ System	Findings
Musculoskeletal	Low back pain, bilateral lower limb proximal power four plus out of five
Respiratory	No respiratory symptoms
Cardiovascular	No cardiovascular symptoms
Gastrointestinal	Weight loss, no bowel symptoms
Neurological	No neurological symptoms
Genitourinary	No urinary symptoms

5. Working Diagnoses:
- Metastatic carcinoma of the stomach

- Spinal epidural abscess

6. Relevant Investigations:
Investigation	Justification
CT-guided biopsy of the paraspinal lesion	To confirm the diagnosis of metastatic carcinoma or spinal epidural abscess
Sputum culture and urine routine microscopy	To rule out pulmonary or urinary tract infection as a potential source of sepsis
Chest X-ray	To evaluate for pulmonary metastases or pneumonia
Blood cultures	To rule out systemic infection
MRI of the spine with gadolinium contrast	To assess the extent of spinal cord compression and plan for surgical intervention if necessary

7. Management Plan:
- Administer broad-spectrum antibiotics to cover potential bacterial pathogens while awaiting culture results |
- Perform CT-guided biopsy of the paraspinal lesion to confirm the diagnosis |
- Consult neurosurgery for evaluation of spinal cord compression and potential surgical intervention |
- Administer analgesics for pain management |
- Monitor for signs of sepsis and multi-organ failure |

8. Executive Summary:
- A 63-year-old male with a history of carcinoma of the stomach presents with low back pain and weight loss |
- Imaging studies reveal a paraspinal lesion suggestive of metastatic carcinoma or spinal epidural abscess |
- Investigations are recommended to confirm the diagnosis and assess the extent of spinal cord compression |
- Management includes broad-spectrum antibiotics, analgesics, and consultation with neurosurgery |
- Close monitoring for signs of sepsis and multi-organ failure is essential |

Claude 2

1. Chronological Events
Event
63 year old male with past medical history of gastric carcinoma status post surgery in 2017, currently in remission
Presented with 5 months history of progressive low back pain more on left side, associated with weight loss

| Private MRI showed irregular thick-walled cystic lesion in left paraspinal musculature from L2-L4 with bony erosion |
| Pending CT-guided biopsy |

2. Clinical Problem List

Clinical Problem	Active/Chronic
Low back pain	Active
Weight loss	Active
History of gastric carcinoma, status post surgery in 2017, currently in remission	Chronic

3. Potential Life-Threatening Complications
- Spinal cord compression from vertebral involvement of paraspinal mass
- Infection or abscess formation from paraspinal mass
- Disseminated malignancy

4. Clinical Findings

Organ System	Anticipated Findings
Neurological	Bilateral lower limb proximal muscle weakness
Musculoskeletal	Tenderness over lumbar spine, limited range of motion of lumbar spine
General	Cachexia, pallor

5. Working Diagnoses
- Paraspinal tumor (e.g. metastasis, lymphoma)
- Osteomyelitis with abscess formation
- Musculoskeletal causes of back pain (e.g. spondylodiscitis)

6. Relevant Investigations

Investigation	Justification
CT-guided biopsy of paraspinal mass	To obtain sample for histopathological diagnosis
Blood cultures	To evaluate for bacteremia/infective cause
MRI lumbar spine	For further evaluation of paraspinal mass, vertebral involvement
PET-CT	For systemic evaluation of cancer staging

7. Management Plan
- Initiate CT-guided biopsy of paraspinal mass for definitive diagnosis
- Start empirical antibiotics to cover for spinal infection
- Neurosurgical consultation for management of impending spinal cord compression

- Oncology consultation if biopsy confirms malignancy
- Analgesia, physiotherapy for back pain
- Nutritional support for cachexia/weight loss
- Thromboprophylaxis
- Counseling and psychosocial support

8. Executive Summary

63 year old male with history of gastric carcinoma, presented with back pain and weight loss. MRI showed invasive paraspinal mass concerning for malignancy/infection. Plan for urgent biopsy, spinal surgery consult for cord compression, empirical antibiotics, nutrition/pain management and thromboprophylaxis. Await biopsy results for definitive oncological management if malignancy confirmed.

3. Clinical scenario 3

GPT-4.0

1. Chronological Events

Date	Event
1 Sep 2023	Left frontal supratentorial tumour total excision; Histology: metastatic small cell carcinoma.
1 Oct 2023	Onset of right-sided weakness.
5 Oct 2023	Index admission; Contrast-enhanced CT brain; Left craniectomy; Start of IV ceftriaxone and metronidazole.
9 Nov 2023	PET-CT shows extra-axial collection and hypermetabolic lung nodule.
13 Nov 2023	MRI brain shows resolving collection, cerebral edema, and rim enhancing lesion.
21 Nov 2023	Seizure; added phenytoin to levetiracetam.
2 Dec 2023	Fever and sore throat; restarted antibiotics.
4 Dec 2023	Transfer back to Hospital Q.
Undated	Physical examination findings noted.

2. Clinical Problem List

Status	Clinical Issues
Active	Right-sided weakness
Active	Postoperative infection
Active	Fever and sore throat
Active	Seizure
Active	Skin rash
Chronic	Metastatic small cell carcinoma
Chronic	History of smoking

Chronic	Social drinking
Chronic	History of senile cataract operation
Chronic	History of open appendicectomy

3. Potential Life-Threatening Complications
- Sepsis or systemic infection due to resistant or inadequately treated Propionibacterium acnes infection.
- Progression of metastatic lung cancer, particularly with central nervous system involvement.
- Seizures leading to status epilepticus.
- Complications from brain metastases, such as increased intracranial pressure or hemorrhage.

4. Clinical Findings

Organ System	Anticipated Findings
Neurological	Focal weakness, signs of potential seizure activity
Integumentary	Diffuse erythematous maculopapular rash
Cardiovascular	Heart sounds dual, no murmurs
Pulmonary	Equal air entry bilaterally, no abnormal lung sounds
Gastrointestinal	Abdomen soft, non-tender

5. Working Diagnoses
- Postoperative infection with Propionibacterium acnes.
- Metastatic small cell lung carcinoma with brain involvement.
- Right-sided weakness secondary to brain metastasis or post-surgical changes.
- Seizure disorder, potentially exacerbated by metastatic brain lesions.
- Drug-induced rash or infection-related rash.

6. Relevant Investigations

Investigation	Justification
Blood cultures	Identify causative organism and guide antibiotic therapy.
Repeat brain imaging (CT/MRI)	Assess post-surgical changes and monitor for metastasis progression.
Chest imaging (CT/PET-CT)	Evaluate primary lung cancer and search for additional metastases.
Skin biopsy	Determine etiology of skin rash.
Seizure focus evaluation (EEG, MRI)	Characterize and localize seizure activity for management.

7. Management Plan

- Review antibiotic regimen and adjust based on culture sensitivities to address postoperative infection.
- Engage Oncology for the management of small cell lung carcinoma, considering systemic therapy.
- Optimize antiepileptic drug therapy to prevent further seizures, with close monitoring for drug levels and interactions.
- Address skin rash with potential allergen withdrawal and dermatology consultation.
- Rehabilitation services for right-sided weakness and overall mobility enhancement.
- Ensure supportive care, including pain management and nutritional support.

8. Executive Summary
A 71-year-old male with a recent neurosurgical intervention for a metastatic brain lesion presents with postoperative infection, seizures, and a new skin rash. Management includes tailored antibiotics, enhanced seizure control, and oncologic evaluation for the primary lung cancer. Monitoring and supportive care are critical, given the complexity of his conditions and the risk of life-threatening complications. Multidisciplinary coordination is essential for optimal outcomes.

cGPT-4

1. Chronological Events:

Date	Event
1 September 2023	Total excision of left frontal supratentorial tumour; histology confirmed metastatic small cell carcinoma.
1 October 2023	Onset of right-sided weakness.
5 October 2023	Index admission; CT brain showing multiloculated rim-enhancing extra-axial collection at left frontoparietal craniotomy site. Left craniectomy with frank pus found.
5 October - 1 December 2023	IV ceftriaxone and IV metronidazole administration.
9 November 2023	PET-CT shows extra-axial collection and hypermetabolic left lower lobe nodule.
13 November 2023	MRI brain shows resolution of epidural collection, reduced cerebral edema, and a possible haemorrhagic brain metastasis.
21 November 2023	Seizure occurrence; phenytoin added to treatment.
2 December 2023	Fever and sore throat; antibiotics restarted. Increasing drowsiness.
4 December 2023	Transfer back to Hospital Q.

2. Clinical Problem List:

Status	Clinical Issues

Active	Right-sided weakness, postoperative infection, seizure, fever and sore throat, worsening drowsiness, diffuse erythematous maculopapular skin rash.
Chronic	History of smoking, social drinking, senile cataract with operation, open appendicectomy, left frontal brain tumour, metastatic small cell lung carcinoma.

3. Potential Life-Threatening Complications:
- Sepsis from postoperative infection.
- Further progression of metastatic small cell lung carcinoma.
- Increased intracranial pressure from brain metastasis leading to herniation.
- Complications from seizures such as status epilepticus.

4. Clinical Findings:

Organ System	Anticipated Findings
Neurological	Hemiparesis, signs of increased intracranial pressure, seizure activity.
Respiratory	Abnormal breath sounds if pneumonia or tumour is causing obstruction.
Integumentary	Rash possibly related to drug reaction or infection.
General	Fever, signs of systemic infection or sepsis.

5. Working Diagnoses:
- Postoperative infection with Propionibacterium acnes.
- Metastatic small cell lung carcinoma with potential brain metastasis.
- Seizure disorder secondary to brain lesions.
- Drug reaction or new infection causing fever and skin rash.

6. Relevant Investigations:

Investigation	Justification
Repeat contrast-enhanced MRI or CT brain	To assess for progression of brain lesions and effectiveness of treatment.
Chest CT scan	To evaluate the primary lung cancer and check for new metastasis or complications.
Blood cultures and sensitivity	To identify causative organisms for the new onset fever and tailor antibiotic treatment.
Electroencephalogram (EEG)	To assess seizure activity and guide antiepileptic therapy.
Skin biopsy of rash	To determine the etiology of the rash, whether it is infectious, inflammatory, or drug-related.

7. Management Plan:
- Continue monitoring and adjusting antiepileptic drugs as needed for seizure control.
- Consideration for oral switch of antibiotics if infection is under control

- Weekly multidisciplinary team review to monitor response to treatment and adjust the management plan
- Regular reviews by a nurse or similar healthcare professional while on therapy
- Enhanced cleaning and review of antibiotic prescribing in case of healthcare-associated infection
- Immediate management of any new signs of sepsis, including but not limited to, broadening antibiotic coverage and supportive care.
- Consultation with oncology for the management of lung cancer and potential brain metastasis.

8. Executive Summary:
The patient is a 71-year-old male with a history of smoking and a recent operation for a brain tumour, who presents with postoperative infection, seizures, and new symptoms of fever and rash. The management plan includes continued antiepileptic therapy, monitoring and possible oral switch of antibiotics, and regular multidisciplinary reviews. Investigations such as repeat imaging and blood cultures are necessary to guide further treatment. There is a need for close monitoring of potential complications from the underlying lung cancer and brain metastasis.

Gemini Pro

1. Chronological Events:

Date	Event
1 September 2023	Left frontal supratentorial tumour total excision
5 October 2023	Index admission for right-sided weakness
5 October 2023	Contrast-enhanced CT brain shows multiloculated rim-enhancing extra-axial collection at left frontoparietal craniotomy site
5 October 2023	Left craniectomy reveals frank pus over epidural space
5 October 2023 - 1 December 2023	Intravenous ceftriaxone and metronidazole for 8 weeks
9 November 2023	PET-CT shows extra-axial collection at craniectomy site, hypermetabolic left lower lobe nodule
13 November 2023	Contrast-enhanced MRI brain shows resolution of epidural collection
21 November 2023	Seizure while on levetiracetam
2 December 2023	Fever, sore throat, worsening drowsiness
4 December 2023	Transfer back to Hospital Q

2. Clinical Problem List:

Active	Chronic
Right-sided weakness	Uncomplicated senile cataract

Epidural abscess	Open appendicectomy
Left frontal brain tumour	Left frontal supratentorial tumour
Lung cancer	Smoker of 50 years
Seizure	Social drinker

3. Potential Life-Threatening Complications:
* Sepsis
* Meningitis
* Brain abscess
* Respiratory failure
* Cardiac arrest

4. Clinical Findings:

Organ System	Anticipated Physical Examination Findings
Integumentary	Diffuse, generalised erythematous maculopapular skin rash
Respiratory	Air entry equal bilaterally
Cardiovascular	Heart sounds dual, no murmur
Neurological	Right-sided weakness
Gastrointestinal	Abdomen soft, non-tender

5. Working Diagnoses:
* Epidural abscess
* Lung cancer
* Seizure disorder

6. Relevant Investigations:

Investigation	Justification
Blood culture	To identify the causative organism of the epidural abscess
Chest X-ray	To assess the extent of lung cancer and identify any complications
CT scan of the brain	To evaluate the extent of the epidural abscess and monitor its response to treatment
MRI of the brain with contrast	To further characterize the epidural abscess and assess for any underlying structural abnormalities
PET-CT scan	To assess the metabolic activity of the lung cancer and identify any metastatic lesions
Electroencephalogram (EEG)	To evaluate the seizure disorder and determine the type of seizure

7. Management Plan:
* Antibiotics: Continue intravenous antibiotics (ceftriaxone and metronidazole) for the treatment of the epidural abscess.

* Surgery: Consider surgical drainage of the epidural abscess if it does not respond to antibiotics.
* Anticonvulsants: Continue levetiracetam and add phenytoin for the management of seizures.
* Palliative care: Provide supportive care for the lung cancer, including pain management and oxygen therapy.
* Close monitoring: Monitor the patient closely for signs of sepsis, meningitis, brain abscess, respiratory failure, and cardiac arrest.

8. Executive Summary:
The patient is a 71-year-old male with a history of lung cancer, left frontal brain tumour, and epidural abscess. He presented with right-sided weakness and fever. Investigations revealed an epidural abscess, lung cancer, and seizure disorder. The patient is currently receiving antibiotics for the epidural abscess and anticonvulsants for the seizure disorder. He is also being monitored closely for signs of sepsis, meningitis, brain abscess, respiratory failure, and cardiac arrest.

Claude 2

1. Chronological Events
Date	Event
1 September 2023	Left frontal brain tumor total excision. Histology: metastatic small cell carcinoma, lung primary
1 October 2023	Onset of right sided weakness
5 October 2023	Admission for right sided weakness. CT brain showed postoperative epidural collection. Left craniectomy done.
5 October 2023 to 1 December 2023	IV ceftriaxone and metronidazole
13 October 2023	MRI brain showed resolving epidural collection
9 November 2023	PET-CT showed hypermetabolic left lower lobe lung nodule, suspected primary cancer
13 November 2023	MRI brain showed resolved epidural collection, stable metastases
21 November 2023	Seizure, started on levetiracetam
2 December 2023	Fever, sore throat, worsening drowsiness
4 December 2023	Transferred back to Hospital Q

2. Clinical Problem List
Problem	Status
Metastatic small cell lung carcinoma	Active
Postoperative epidural collection	Resolved
Seizures	Active

| Fever, sore throat, altered mental status | Active |

3. Potential Life-Threatening Complications
- Herpes simplex encephalitis
- Brain edema and raised intracranial pressure from metastases
- Status epilepticus

4. Clinical Findings

System	Expected Findings
General	Tachycardia, fever, altered mental status
HEENT	Oropharyngeal erythema
Cardiovascular	Tachycardia
Respiratory	Normal breath sounds
Abdominal	Soft, non-tender
Neurologic	Lethargy, disorientation, right sided weakness
Dermatologic	Maculopapular rash

5. Working Diagnoses
- Herpes simplex encephalitis
- Breakthrough seizures
- Medication reaction/rash

6. Relevant Investigations

Investigation	Rationale
MRI brain with contrast	Evaluate for evidence of encephalitis or progression of brain metastases
EEG	Confirm seizure activity
CSF analysis (cell count, protein, glucose, HSV PCR, cultures)	Diagnose or exclude encephalitis
Complete blood count, metabolic panel	Assess response to antibiotics, organ function
Chest X-ray	Assess for pneumonia
Anticonvulsant drug levels	Ensure therapeutic levels

7. Management Plan
- Admit to intensive care for close monitoring
- Start IV acyclovir for suspected HSV encephalitis
- Continue antiepileptics (levetiracetam, phenytoin) and monitor drug levels
- Obtain stat infectious disease consult
- If sepsis, start broad spectrum antibiotics after cultures obtained
- If drug reaction, stop phenytoin and consider short course steroids

- Avoid any further neurosurgical procedures until infection treated
- Control seizures, agitation and raised intracranial pressure medically
- Address goals of care with family given poor prognosis of metastatic cancer

8. Executive Summary

This 71-year-old man with metastatic small cell lung cancer developed postoperative encephalitis after a craniectomy, presenting with seizures, altered mental status and rash. He requires ICU monitoring, empiric acyclovir, antiepileptics, evaluation for drug reaction, and SEA consultation to diagnose and manage this life-threatening presentation. His prognosis is guarded due to advanced cancer.

4. Clinical scenario 4

GPT-4.0

1. Chronological Events:

Date	Event
25 Oct 2023	Admission for multiple medical issues, including new onset heart failure (HFrEF) with LVEF ~30%, complete total occlusion of the left anterior descending artery, fast atrial fibrillation, metastatic adenocarcinoma of lung.
25-27 Oct 2023	Amoxicillin-clavulanate administered.
27 Oct 2023	Blood culture with no growth.
27-31 Oct 2023	Piperacillin-tazobactam administered.
31 Oct 2023	PET-CT showed features suggestive of lung cancer with extensive metastases, right adrenal metastasis. Meropenem started.
25 Oct - 10 Nov 2023	Doxycycline administered.
11 Nov 2023	Meropenem discontinued.
20 Nov 2023	Piperacillin-tazobactam restarted.
22 Nov 2023	Blood culture pending, NPS respiratory virus panel pending. Doxycycline restarted. ANC elevated, INR 1.5.
Not specified	Desaturation leading to 100% oxygen requirement and transfer back to Hospital G for further management.
Not specified	Repeated scan shows progression of right upper lobe consolidation, multiple intrapulmonary metastasis, lymphangitis carcinomatosis, new right pleural effusion, no pulmonary embolism.

2. Clinical Problem List:

Status	Clinical Problems
Active	New onset heart failure (HFrEF)
Active	Complete occlusion of left anterior descending artery
Active	Fast atrial fibrillation

Active	Metastatic adenocarcinoma of lung
Active	Desaturation requiring 100% oxygen
Active	Right upper lobe consolidation
Active	Right pleural effusion
Chronic	Hyperlipidemia
Chronic	History of closed PDA
Chronic	History of repaired CoA

3. Potential Life-Threatening Complications:
- Acute decompensated heart failure
- Hemodynamic instability due to fast atrial fibrillation
- Acute respiratory failure from lung metastasis and pleural effusion
- Complications from cancer such as superior vena cava syndrome or spinal cord compression

4. Clinical Findings:

Organ System	Anticipated Findings
Cardiovascular	Pansystolic murmur, possible signs of heart failure
Respiratory	Bilateral crepitations, signs of respiratory distress
Abdominal	Soft abdomen, no hepatosplenomegaly noted
Extremities	Calves soft, no signs of deep vein thrombosis
General	Requires 100% oxygen, no fever, no signs of infection at drip site

5. Working Diagnoses:
- Decompensated heart failure with reduced ejection fraction (HFrEF)
- Untreated complete occlusion of the left anterior descending artery
- Fast atrial fibrillation
- Advanced metastatic adenocarcinoma of the lung
- Respiratory failure requiring 100% oxygen supplementation

6. Relevant Investigations:

Investigation	Justification
Repeat echocardiogram	Assess current LVEF and cardiac function
Chest X-ray or CT scan	Evaluate pleural effusion and lung metastases
Complete blood count (CBC)	Monitor for anemia, infection, or other hematological conditions
Liver and renal function tests	Assess baseline organ function due to cancer and drug therapy
Repeat blood cultures	Rule out sepsis
Serial monitoring of CEA levels	Monitor cancer progression or response to therapy
Therapy-specific cancer markers	If applicable, for targeted therapy options

| Electrolytes, including calcium and magnesium | Monitor for electrolyte imbalances secondary to cancer or therapies |

7. Management Plan:
- Continue supplemental oxygen and monitor oxygen saturation levels
- Monitor hemodynamics and manage heart failure with appropriate medications (e.g., diuretics, ACE inhibitors)
- Consider anticoagulation for atrial fibrillation and assess need for rate or rhythm control
- Oncology referral for the management of metastatic adenocarcinoma which may include palliative care, systemic chemotherapy, or targeted therapy as appropriate
- Pain management and supportive care for cancer-related symptoms
- Evaluate need for pleural effusion drainage if causing significant respiratory compromise
- Nutritional support and physical therapy for maintenance of muscle mass and function
- Engage palliative care services for holistic care, including symptom management and advanced care planning

8. Executive Summary:
The patient is a 62-year-old female with a complex medical history including heart failure with reduced ejection fraction, coronary artery disease, and metastatic lung adenocarcinoma, presenting with respiratory failure requiring 100% oxygen. Immediate concerns include the management of decompensated heart failure, fast atrial fibrillation, and the complications arising from widespread metastatic cancer. The management plan involves a multidisciplinary approach, addressing cardiac and oncologic conditions, close monitoring, and supportive care. Palliative care should be involved early to manage symptoms and to assist with complex decision-making given the advanced stage of cancer.

cGPT-4

1. Chronological Events:

Date	Clinical Issue
25 Oct 2023	Admission for multiple medical issues
	New onset heart failure with reduced ejection fracture
	Coronary angiogram: LAD complete total occlusion
	Fast atrial fibrillation
31 Oct 2023	PET-CT showed features suggestive of metastatic adenocarcinoma of lung
26 Oct 2023	CEA level at 26 ng/ml
	EGFR negative
	Biopsy: Metastatic carcinoma, adenocarcinoma

28 Oct 2023	Blood culture with no growth
22 Nov 2023	Blood culture pending
	NPS respiratory virus panel pending

2. Clinical Problem List:

Status	Clinical Issue
Active	New onset heart failure with reduced ejection fracture
	Coronary angiogram: LAD complete total occlusion
	Fast atrial fibrillation
	Metastatic adenocarcinoma of lung
Chronic	Hyperlipidemia
	History of closed patent ductus arteriosus (PDA)
	Repaired coarctation of aorta (CoA)

3. Potential Life-Threatening Complications:
- Acute decompensated heart failure
- Arrhythmia leading to hemodynamic instability
- Rapid progression of metastatic adenocarcinoma with respiratory failure

4. Clinical Findings:

Organ System	Anticipated Findings
Cardiovascular	Pansystolic murmur, possible signs of heart failure
Respiratory	Desaturation, bilateral crepitations, pleural effusion
General	Requirement for 100% oxygen supplementation

5. Working Diagnoses:
- Heart failure with reduced ejection fracture (HFrEF)
- Coronary artery disease (CAD) with LAD occlusion
- Metastatic adenocarcinoma of the lung

6. Relevant Investigations:

Investigation	Justification
Echocardiogram	Assess cardiac structure and function
Repeat PET-CT	Evaluate progression of metastatic disease
Blood culture	Rule out infection as cause of fever and desaturation
Full blood count	Assess for anemia and signs of infection
Serum electrolytes	Monitor and manage fluid status and renal function
Liver function tests	Assess hepatic involvement by metastasis
Repeat CEA	Monitor tumor marker for lung cancer progression

7. Management Plan:

- Continue oxygen therapy to maintain adequate oxygenation.
- Symptomatic management of heart failure, including diuretics for fluid overload.
- Anticoagulation therapy for atrial fibrillation and to prevent thromboembolic events.
- Pain management and palliative care consultations for metastatic cancer.
- Consideration of targeted therapies or chemotherapy for adenocarcinoma after oncology review.
- Monitor for signs of infection and adjust antimicrobial therapy as needed

8. Executive Summary:
The patient is a 62-year-old female with a history of hyperlipidemia, closed PDA, and repaired CoA, currently facing new onset HFrEF, complete occlusion of the LAD, fast atrial fibrillation, and metastatic adenocarcinoma of the lung. Immediate management includes oxygen supplementation, heart failure treatment, arrhythmia control, and cancer therapy consideration. Vigilant monitoring for complications and potential infections is crucial. Palliative care consultation is recommended to address quality of life and symptom management.

Gemini Pro

1. Chronological Events:
Date	Clinical Issue
25 October 2023	Admission for multiple medical issues, including new-onset heart failure with reduced ejection fraction (HFrEF), coronary angiogram showing left anterior descending artery complete total occlusion, fast atrial fibrillation, and metastatic adenocarcinoma of lung
31 October 2023	Positron Emission Tomography-Computed Tomography (PET-CT) reveals features suggestive of lung cancer with bilateral intrapulmonary metastases, bilateral pleural metastases, extensive nodal metastases, and right adrenal metastasis
28 October 2023	Urine Legionella antigen negative
22 November 2023	Nasopharyngeal swabs (NPS) respiratory virus panel pending
22 November 2023	Blood culture pending

2. Clinical Problem List:
Active	Chronic
New-onset heart failure with reduced ejection fraction (HFrEF)	Hyperlipidemia
Coronary angiogram showing left anterior descending artery complete total occlusion	History of closed patent ductus arteriosus (PDA) and repaired coarctation of aorta (CoA)
Fast atrial fibrillation	Metastatic adenocarcinoma of lung
Pneumonia	

3. Potential Life-Threatening Complications:
* Cardiogenic shock
* Pulmonary embolism
* Stroke
* Respiratory failure
* Sepsis

4. Clinical Findings:

Organ System	Findings
Respiratory	Bilateral crepitations
Cardiovascular	Pansystolic murmur best heard at left lower sternal border
Gastrointestinal	Abdomen soft
Musculoskeletal	Calves soft

5. Working Diagnoses:
* Pneumonia
* Metastatic adenocarcinoma of lung
* Heart failure with reduced ejection fraction (HFrEF)
* Atrial fibrillation

6. Relevant Investigations:

Investigation	Justification
Chest X-ray	To assess the extent of pneumonia and identify any underlying lung pathology
Blood cultures	To identify any potential bloodstream infection
Sputum culture	To identify the causative organism of pneumonia and determine antibiotic susceptibility
Respiratory virus panel	To rule out any respiratory viral infection
Echocardiogram	To assess cardiac function and identify any structural abnormalities
Electrocardiogram (ECG)	To evaluate heart rhythm and identify any arrhythmias

7. Management Plan:
* Initiate broad-spectrum antibiotics to cover potential bacterial pathogens, such as piperacillin-tazobactam or meropenem.
* Administer oxygen therapy to maintain adequate oxygenation.
* Monitor cardiac function closely and provide supportive care, such as diuretics and ACE inhibitors, for heart failure.
* Consider anticoagulation therapy to prevent thromboembolic complications.
* Palliative care and symptom management for metastatic adenocarcinoma of lung.

8. Executive Summary:

A 62-year-old female with a history of hyperlipidemia, closed patent ductus arteriosus (PDA), and repaired coarctation of aorta (CoA) presented with new-onset heart failure, atrial fibrillation, and metastatic adenocarcinoma of lung. She developed pneumonia during her hospital stay. The patient requires broad-spectrum antibiotics, oxygen therapy, and supportive care for her cardiac and respiratory conditions. Palliative care and symptom management are necessary for her metastatic lung cancer.

Claude 2

1. Chronological Events

Date	Event
25 October 2023	Admitted for new onset heart failure, fast atrial fibrillation, metastatic lung cancer
26 October 2023	CEA 26 ng/ml, EGFR negative
27 October 2023	Changed antibiotics
28 October 2023	PET-CT showed metastases, Legionella antigen negative
31 October 2023	Changed antibiotics
31 October 2023	Biopsy showed metastatic adenocarcinoma
20 November 2023	Changed antibiotics
22 November 2023	Blood culture pending, NPS respiratory panel pending

2. Clinical Problem List

Active Problems	Chronic Problems
Heart failure with reduced ejection fraction	Hyperlipidemia
Fast atrial fibrillation	History of closed PDA and repaired CoA
Metastatic lung adenocarcinoma	
Lymphangitis carcinomatosis	
Right pleural effusion	

3. Potential Life-Threatening Complications
- Respiratory failure from lymphangitis carcinomatosis and pleural effusion
- Hemodynamic instability from heart failure and arrhythmia
- Sepsis from immunosuppression

4. Clinical Findings

System	Findings
Respiratory	Tachypnea, hypoxia requiring oxygen, bilateral crepitations
Cardiovascular	Tachycardia, pansystolic murmur
Abdominal	Soft, non-tender

| Extremities | Pitting edema |

5. Working Diagnoses
- Metastatic lung adenocarcinoma
- Lymphangitis carcinomatosis
- Right pleural effusion
- Heart failure with reduced ejection fraction
- Fast atrial fibrillation

6. Relevant Investigations

Investigation	Justification
Sputum bacterial culture	Evaluate for bacterial pneumonia
Blood cultures	Evaluate for bacteremia/sepsis
Respiratory virus PCR panel	Evaluate for viral pneumonia
TB testing	Evaluate for TB pneumonia
CT chest	Assess cancer progression and complications
Echocardiogram	Evaluate heart failure severity
Electrocardiogram	Evaluate arrhythmia

7. Management Plan
- Oxygen supplementation to maintain saturation >92%
- Analgesia for comfort
- Diuretics for fluid overload
- Anticoagulation for atrial fibrillation
- Beta-blockers for rate control
- Antibiotics for pneumonia per culture results
- Thoracentesis for large pleural effusions
- Palliative radiation or chemotherapy for cancer
- Address goals of care and code status

8. Executive Summary
This 62F with metastatic lung cancer, heart failure, and atrial fibrillation has respiratory failure likely from lymphangitis and pleural effusion. She is at risk for sepsis and cardiorespiratory collapse. Key next steps are optimizing respiratory status, continuing antibiotics, evaluating culture results, considering thoracentesis, and addressing goals of care.

5. Clinical scenario 5

GPT-4.0

1. Chronological Events

| Date | Event |

Feb 2018	Diagnosed with multiple myeloma (ISS1 IgG MM).
Mar-Jul 2018	Treated with Bortezomib, cyclophosphamide, and dexamethasone (VCD).
22 Aug 2018	Underwent autologous stem-cell transplantation (ASCT).
Aug 2020	Relapse of multiple myeloma.
Nov 2020-Apr 2021	Treated with Daratumumab, lenalidomide, and dexamethasone (Dara Rd).
Jan-Aug 2023	Treated with Isatuximab, carfilzomib, and dexamethasone (Isa Kd).
21-23 Oct 2023	Received chemotherapy in Hangzhou for CAR T-Cell Therapy.
24-25 Oct 2023	Received CAR T-Cell Therapy.
Early Nov 2023	Developed fever, lasted for 6 days, then subsided for 4 days.
12 Nov 2023	Admitted for neutropenic fever.
14 Nov 2023	Ultrasound showed diffuse increased liver echogenicity and other findings.
15 Nov 2023	Blood and stool workup, sputum culture, and chest X-ray performed.

2. Clinical Problem List

Status	Clinical Issues
Active	Neutropenic fever
Active	Post-CAR T-Cell Therapy complications
Active	Dry cough with no sputum
Active	Watery diarrhea
Chronic	Diabetes mellitus
Chronic	Ischemic heart disease
Chronic	Multiple myeloma (in remission with recent relapse)
Chronic	Liver echogenicity suggesting parenchymal disease
Chronic	Gallstones and possible adenomyomatosis

3. Potential Life-Threatening Complications

- Neutropenic fever: Risk of severe sepsis or septic shock.
- Post-CAR T-Cell Therapy complications: Cytokine release syndrome (CRS), immune effector cell-associated neurotoxicity syndrome (ICANS), infection due to immunosuppression.
- Multiple myeloma and recent chemotherapy: Increased risk of opportunistic infections and bleeding due to thrombocytopenia.

4. Clinical Findings

Organ System	Anticipated Findings
General	Swinging fever, tiredness
Cardiovascular	Blood pressure and pulse unsupported, absence of lower limb edema

Respiratory	Chest clear
Gastrointestinal	Abdomen soft non-tender, watery diarrhea
Hepatic	Possible hepatomegaly or tenderness if liver involvement is present
Musculoskeletal	No joint pain, calves are soft
Hematologic	Signs of anemia or bleeding due to thrombocytopenia may be present
Dermatologic	Absence of rash

5. Working Diagnoses
- Neutropenic fever likely due to bacterial or fungal infection.
- Possible post-CAR T-Cell Therapy complications (e.g., CRS, ICANS).
- Gastrointestinal disturbance, etiology to be determined (infectious vs. medication-related).
- Liver abnormalities suggestive of drug-induced liver injury or infection.

6. Relevant Investigations

Test	Justification
Blood cultures	To identify causative organism(s) for neutropenic fever
Stool cultures	To determine the cause of diarrhea
Chest X-ray	To rule out pneumonia or other respiratory complications
Liver function tests	To assess liver injury and to monitor the progression of liver disease
Serum electrolytes	To monitor for electrolyte imbalances due to diarrhea
CT scan of abdomen	To evaluate liver echogenicity and gallbladder changes more precisely
CAR T-Cell Therapy complications specific assays	To detect CRS or ICANS if clinically suspected

7. Management Plan
- Continue broad-spectrum antimicrobials (Meropenem, Voriconazole) until specific pathogens are identified or ruled out.
- Monitor complete blood counts, vitals, and organ function closely, adjust supportive care as needed.
- Evaluate for CAR T-Cell Therapy complications and treat accordingly (e.g., tocilizumab for CRS, supportive care for ICANS).
- Manage diabetes with insulin or oral hypoglycemics as needed, with adjustments for current illness and treatments.
- Maintain strict aseptic technique and consider antifungal and antiviral prophylaxis given immunosuppression.
- Nutritional support and fluid management to address diarrhea and prevent dehydration.

8. Executive Summary

The patient, a 65-year-old female with a history of multiple myeloma and recent CAR T-Cell Therapy, presents with neutropenic fever, dry cough, and watery diarrhea. The clinical scenario suggests an infectious etiology for the fever, possibly compounded by post-therapy complications. Immediate concerns include infection due to neutropenia and potential complications from recent immunotherapy. Management has appropriately prioritized broad-spectrum antimicrobials and supportive care while awaiting specific diagnostic results. Monitoring for CAR T-Cell Therapy complications is essential, with a readiness to initiate targeted treatments for complications like CRS. The management plan emphasizes the importance of maintaining organ function, prophylactic treatments, and strict infection control practices.

cGPT-4

1. Chronological Events:

Date	Event
Feb 2018	Diagnosed with Multiple Myeloma (ISS1 IgG MM)
Mar-Jul 2018	Treated with Bortezomib, cyclophosphamide, and dexamethasone (VCD)
22 Aug 2018	Underwent Autologous stem-cell transplantation (ASCT)
Aug 2020	Relapsed
Nov 2020-Apr 2021	Treated with Daratumumab, lenalidomide, and dexamethasone (Dara Rd)
Jan-Aug 2023	Treated with Isatuximab, carfilzomib, and dexamethasone (Isa Kd)
21-23 Oct 2023	Received chemotherapy for CAR T-Cell Therapy in Hangzhou
24-25 Oct 2023	CAR T-Cell Therapy
12 Nov 2023	Admitted for neutropenic fever
14 Nov 2023	Ultrasound showing signs suggestive of parenchymal disease
15 Nov 2023	Investigation results received, pending stool workup

2. Clinical Problem List:

Status	Clinical Issues
Active	Neutropenic fever
Active	Swinging fever
Active	Dry cough
Active	Watery diarrhea
Chronic	Diabetes Mellitus
Chronic	Ischaemic heart disease
Chronic	Multiple Myeloma
Chronic	Allergies to multiple antibiotics

3. Potential Life-Threatening Complications:

- Sepsis due to neutropenic fever
- Opportunistic infections secondary to immune suppression from chemotherapy and underlying multiple myeloma
- Complications from underlying Ischaemic heart disease

4. Clinical Findings:

Organ System	Anticipated Findings
General	Fatigue, fever
Respiratory	Clear chest on auscultation
Gastrointestinal	Watery diarrhea, non-tender abdomen
Hepatic	Suggestive of parenchymal disease from ultrasound
Hematologic	Neutropenia, anemia, thrombocytopenia

5. Working Diagnoses:
- Bacterial infection secondary to neutropenic fever
- Viral respiratory infection due to Enterovirus/Rhinovirus RNA detected
- Drug-induced liver injury or fatty liver as suggested by ultrasound
- Gastrointestinal infection as a cause of diarrhea

6. Relevant Investigations:

Investigation	Justification
Blood culture	To identify causative organism for neutropenic fever
Stool culture and sensitivity	To identify causative organism for diarrhea
Liver function tests	To monitor liver status and evaluate for drug-induced liver injury
Repeat chest X-ray	To evaluate for progression of respiratory symptoms
Complete blood count	To monitor for hematologic recovery post chemotherapy and CAR T-Cell therapy

7. Management Plan:
- Continue broad-spectrum antibiotics (Meropenem) and antifungal (Voriconazole)
- Monitor vital signs closely for signs of sepsis.
- Supportive care for neutropenia, including growth factor support if indicated.
- Manage blood glucose levels to optimize diabetic control.
- Continue acyclovir prophylaxis and pentamidine inhalation to prevent opportunistic infections.
- Consider gallbladder evaluation due to findings on ultrasound.
- Regular interdisciplinary team reviews to adjust therapy as needed
- Ensure safe and effective medication administration and monitoring for adverse reactions

8. Executive Summary:
The patient is a 65-year-old female with multiple myeloma and a history of recent CAR T-Cell therapy, now presenting with neutropenic fever. She has active symptoms of fever, cough, and diarrhea, with underlying chronic conditions including diabetes and ischemic heart disease. Immediate concerns include potential sepsis and complications from underlying conditions. Management includes continuation of broad-spectrum antibiotics and antifungals, close monitoring, and supportive care. Further investigations are necessary to identify causative agents and adjust the treatment plan accordingly.

Gemini Pro

1. Chronological Events:

Date	Event
February 2018	Multiple myeloma (ISS1 IgG MM) diagnosed
March to July 2018	Bortezomib, cyclophosphamide and dexamethasone (VCD) treatment
22 August 2018	Autologous stem-cell transplantation (ASCT) performed
August 2020	Relapse of multiple myeloma
November 2020 to April 2021	Daratumumab with lenalidomide and dexamethasone (Dara Rd) treatment
January to August 2023	Isatuximab, carfilzomib and dexamethasone (Isa Kd) treatment
21 to 23 October 2023	Chemotherapy received in Hangzhou for Chimeric Antigen Receptor (CAR) T-Cell Therapy
24 to 25 October 2023	CAR T-Cell therapy received in Hangzhou
12 November 2023	Admitted to the hospital with neutropenic fever
13 November 2023	Sputum culture and nasopharyngeal swabs (NPS) collected
14 November 2023	Ultrasound of the abdomen performed, Voriconazole treatment started
15 November 2023	Chest X-ray (CXR) performed, Stool workup initiated
16 November 2023	Blood culture collected

2. Clinical Problem List:

Active	Chronic
Neutropenic fever	Diabetes mellitus
Enterovirus/Rhinovirus infection	Ischaemic heart disease
Multiple myeloma	Allergy to amoxicillin-clavulanate, ampicillin, thalidomide, cotrimoxazole

3. Potential Life-Threatening Complications:
* Sepsis
* Respiratory failure
* Multi-organ failure

4. Clinical Findings:

Organ System	Anticipated Physical Examination Findings
Respiratory	Fever, swinging fever, dry cough, no sputum
Cardiovascular	Unsupported blood pressure and pulse
Gastrointestinal	Watery diarrhea
Hepatobiliary	Diffuse increased liver echogenicity, suggestive of parenchymal disease like fatty liver, non dilated biliary tract, gallstones, focal cystic areas within gallbladder wall, query adenomyomatosis
Musculoskeletal	Soft calves, no lower limb edema
Lymphatic	No palpable cervical lymphadenopathy
Integumentary	No rash

5. Working Diagnoses:
* Neutropenic fever
* Enterovirus/Rhinovirus infection
* Multiple myeloma relapse

6. Relevant Investigations:

Investigation	Justification
Complete blood count (CBC) with differential	To monitor neutrophil count and assess for cytopenias
Serum electrolytes, creatinine, liver function tests	To assess organ function and electrolyte balance
Blood cultures	To identify and guide appropriate antimicrobial therapy
Sputum culture	To identify potential respiratory pathogens
Stool workup	To identify potential gastrointestinal pathogens
Chest X-ray	To evaluate for pulmonary infiltrates or consolidation
Abdominal ultrasound	To assess for hepatobiliary abnormalities
Bone marrow biopsy	To evaluate for multiple myeloma relapse

7. Management Plan:
* Continue broad-spectrum antibiotics (Meropenem and Voriconazole) until culture results are available.
* Administer granulocyte colony-stimulating factor (G-CSF) to stimulate neutrophil production.
* Monitor for signs and symptoms of sepsis and respiratory failure.

* Provide supportive care, including intravenous fluids and electrolytes.
* Consider antiviral therapy for Enterovirus/Rhinovirus infection if symptoms persist.
* Evaluate for multiple myeloma relapse and initiate appropriate therapy if confirmed.

8. Executive Summary:
A 65-year-old female with a history of multiple myeloma, diabetes mellitus, and ischaemic heart disease presented with neutropenic fever and Enterovirus/Rhinovirus infection. She received chemotherapy and CAR T-Cell therapy in Hangzhou for multiple myeloma relapse. Investigations revealed diffuse increased liver echogenicity and focal cystic areas within the gallbladder wall. The patient is currently being treated with broad-spectrum antibiotics and supportive care. Further investigations are needed to evaluate for multiple myeloma relapse.

Claude 2

1. Chronological Events
Date	Event
February 2018	Diagnosed with multiple myeloma, started on bortezomib, cyclophosphamide and dexamethasone (VCD)
March - July 2018	Ongoing VCD therapy
22 August 2018	Underwent autologous stem cell transplant
August 2020	Relapse of multiple myeloma
November 2020 - April 2021	Started on daratumumab, lenalidomide and dexamethasone (Dara Rd)
January - August 2023	Treated with isatuximab, carfilzomib and dexamethasone (Isa Kd)
21-23 October 2023	Received chemotherapy prior to CAR T-cell therapy
24-25 October 2023	Underwent CAR T-cell therapy
6 days after CAR T-cell therapy	Developed fever
4 days after fever onset	Fever resolved temporarily
12 November 2023	Admitted with neutropenic fever

2. Clinical Problem List
Status	Problems
Active	Febrile neutropenia, diarrhea, cough
Chronic	Multiple myeloma, diabetes mellitus, ischemic heart disease

3. Potential Life-Threatening Complications
- Sepsis/septic shock from neutropenic fever
- Respiratory failure from pneumonia

- Clostridioides difficile colitis leading to toxic megacolon

4. Anticipated Physical Examination Findings

System	Findings
General	Febrile, fatigued/ill-appearing
HEENT	No cervical lymphadenopathy
Respiratory	Clear lungs
Cardiovascular	Tachycardia, hypotension
Abdominal	Soft, non-tender
Extremities	No lower limb edema

5. Working Diagnoses
- Febrile neutropenia
- Clinical evidence: fever, neutropenia, recent chemotherapy
- Diarrhea
- Clinical evidence: watery diarrhea
- Viral upper respiratory infection
- Clinical evidence: cough, enterovirus/rhinovirus on respiratory PCR panel

6. Relevant Investigations

Investigation	Justification
Blood cultures	To identify bacterial pathogen causing febrile neutropenia
Stool culture	To identify bacterial pathogen causing diarrhea
Stool C. difficile toxin	To evaluate for C. difficile colitis
Chest X-ray	To evaluate for pneumonia
Blood count, renal function	Monitor neutropenia, electrolyte abnormalities

7. Management Plan
- Continue broad spectrum antibiotics (meropenem) to cover for febrile neutropenia
- IV fluids for hydration support
- Antivirals if influenza/RSV identified from respiratory panel
- Antidiarrheals if infectious workup negative
- C. difficile testing if diarrhea worsens
- Daily monitoring of counts, organ function
- Sepsis management if patient deteriorates clinically
- Transfusion support for severe anemia/thrombocytopenia
- Prompt imaging and respiratory support if respiratory decompensation
- Consider discharge when counts recover and patient is afebrile >48 hours

8. Executive Summary

This 65-year-old female with multiple myeloma and diabetes mellitus presented with febrile neutropenia, diarrhea and cough after recent CAR T-cell therapy. She is at risk for severe sepsis and requires broad antibiotics, fluid support and close monitoring of counts/organ function. C. difficile colitis should be evaluated if diarrhea persists. Prompt intervention is needed for any clinical deterioration.

6. Clinical scenario 6

GPT-4.0

1. Chronological Events

Date	Event
May 2022	Streptococcus suis bacteraemia and meningitis; ventriculitis on MRI
October 2022	MRSA bacteraemia
November 2022	MRSA bacteraemia
December 2022	Gallium scan negative for active infection; Plain CT shows left hip erosion
28 Nov 2023	Admission for CT assessment; CT reveals collection near left hip suggestive of infection
2 Dec 2023	Left hip aspiration with elevated nucleated cell count; culture pending

2. Clinical Problem List

Status	Clinical Issues
Active	Suspected septic arthritis with gluteal abscess
Chronic	Hypertension
Chronic	Diabetes mellitus
Chronic	Hyperlipidaemia
Chronic	Left central retinal artery occlusion (CRAO)
Chronic	Bilateral hip heterotopic ossification
Chronic	Recurrent seizure
Chronic	Deafness in left ear

3. Potential Life-Threatening Complications
- Septic shock secondary to septic arthritis and potential gluteal intramuscular abscess
- Recurrent MRSA bacteraemia
- Further seizures potentially leading to status epilepticus
- Hip erosion progressing to pathological fracture

4. Clinical Findings

Organ System	Anticipated Findings

Musculoskeletal	Absence of hip pain; possible decreased range of motion
Neurological	Signs of seizure activity; altered mental status
Auditory	Decreased hearing in left ear
Vascular	Signs of peripheral vascular disease due to diabetes

5. Working Diagnoses
- Septic arthritis with gluteal intramuscular abscess
- Recurrent MRSA bacteraemia
- Heterotopic ossification with potential hip erosion

6. Relevant Investigations

Investigation	Justification
Blood cultures	Identify causative organism for targeted treatment
Repeated MRI or CT	Assess progression of hip erosion and abscess
ESR/CRP	Monitor inflammation status
Hip aspiration culture	Confirm infection and identify causative bacteria

7. Management Plan
- Antimicrobial Therapy: Empirical broad-spectrum antibiotics while awaiting culture results, with subsequent adjustment based on sensitivities.
- Surgical Evaluation: Consult orthopedics for potential debridement of the hip abscess.
- Seizure Management: Continue antiepileptics and monitor levels; assess need for adjustment.
- Supportive Care: Bed rest, nutritional support, and physiotherapy to prevent deconditioning.
- Multidisciplinary Approach: Involve infectious disease specialists, neurologists, and cardiologists in management.
- Monitoring: Regular vitals, blood glucose, and pressure monitoring, along with inflammatory markers.

8. Executive Summary
The patient, a 67-year-old male with a complex medical history including diabetes, hypertension, and previous infections, presents with potential septic arthritis and a gluteal intramuscular abscess. Immediate concerns include managing suspected septic arthritis and preventing recurrent MRSA bacteraemia and seizure complications. Investigations should focus on identifying the causative organism and monitoring the inflammatory response. A comprehensive management plan should include tailored antibiotic therapy, surgical consultation for the abscess, and a multidisciplinary approach to address his chronic conditions and prevent further complications.

cGPT-4

1. Chronological Events:

Date	Clinical Event
May 2022	Streptococcus suis bacteraemia and meningitis
May 2022	Ventriculitis on MRI brain, recurrent seizure
May 2022	External ventricular drainage, vancomycin treatment
October 2022	MRSA bacteraemia
November 2022	MRSA bacteraemia
December 2022	Gallium scan showed no active infection
December 2022	CT abdomen and pelvis found left hip erosion
28 November 2023	CT Left Hip: Suspected septic arthritis with abscess
2 December 2023	Left hip aspiration, culture pending

2. Clinical Problem List:

Status	Clinical Issue
Active	Suspected left hip septic arthritis with abscess
Chronic	Hypertension
Chronic	Diabetes mellitus
Chronic	Hyperlipidaemia
Chronic	Left central retinal artery occlusion (CRAO)
Chronic	Bilateral hip heterotopic ossification
Chronic	Left ear deafness

3. Potential Life-Threatening Complications:
- Septic arthritis could lead to rapid joint destruction, systemic infection, and sepsis.
- Recurrent MRSA bacteraemia may result in endocarditis or metastatic infection.
- Status post meningitis and ventriculitis increases the risk for recurrent CNS infections or complications.

4. Clinical Findings:

Organ System	Anticipated Findings
Musculoskeletal	Absence of hip pain (as per patient denial), possible hip joint effusion or edema
Neurological	History of seizures may indicate abnormal neurological findings
Cardiovascular	Possible findings related to hypertension or sequelae of bacteraemia
Dermatological	Signs of infection or systemic illness, e.g., pallor, diaphoresis

5. Working Diagnoses:
- Septic arthritis with gluteal intramuscular abscess of the left hip.
- Possible recurrent MRSA bacteraemia without current evidence of active infection.

- Post-infectious sequelae of Streptococcus suis infection including meningitis and ventriculitis.

6. Relevant Investigations:

Investigation	Justification
Blood cultures	To identify causative organisms for potential recurrent bacteraemia
Joint fluid Gram stain and culture	To confirm diagnosis of septic arthritis and identify causative organisms
ESR, CRP	To monitor inflammation and response to treatment
Serial neurologic exams	To detect any new or worsening CNS complications

7. Management Plan:
- Continue monitoring vital signs and laboratory parameters to assess for signs of infection or systemic inflammation.
- Administer the first dose of any new antibiotic therapy under supervision to manage potential anaphylaxis
- Daily review by a nurse or similar healthcare provider with a view to oral switch and weekly review in a multidisciplinary team meeting
- Ensure all equipment used in patient care is properly cleaned and maintained to prevent nosocomial infections
- Consider the patient's home environment for any necessary modifications or support due to his bedbound status
- Follow up with the culture results from the left hip aspiration and adjust antibiotic therapy accordingly.
- Evaluate the need for surgical intervention for the left hip condition depending on the culture results and clinical response to antibiotics.

8. Executive Summary:
The patient has a complex medical history, including recent infectious complications and suspected left hip septic arthritis with abscess. A management plan should include close monitoring, tailored antibiotic therapy, and multidisciplinary review. Investigations should focus on confirming the diagnosis and monitoring for complications. Preventative measures for nosocomial infections and a consideration of the patient's home environment are also crucial due to his bedbound state.

Gemini Pro

1. Chronological Events:

Date	Clinical Issue
May 2022	Streptococcus suis bacteraemia and meningitis

October and November 2022	Methicillin-resistant Staphylococcus aureus (MRSA) bacteraemia
December 2022	Gallium scan: No evidence of active infection
December 2022	Plain CT abdomen and pelvis: Left hip erosion
28 November 2023	Admitted for CT assessment
28 November 2023	Contrast CT Left Hip: Worrisome septic arthritis with gluteal intramuscular abscess
2 December 2023	Left hip aspiration: Elevated WBC, CRP, ESR; culture pending

2. Clinical Problem List:

Active	Chronic
Septic arthritis of the left hip	Hypertension
Diabetes mellitus	Hyperlipidaemia
Recurrent seizure	Left central retinal artery occlusion (CRAO)
Bilateral hip heterotopic ossification	

3. Potential Life-Threatening Complications:
- Sepsis
- Disseminated intravascular coagulation (DIC)
- Multi-organ failure
- Death

4. Clinical Findings:

Organ System	Anticipated Findings
Musculoskeletal	Left hip pain, swelling, erythema, warmth
Integumentary	Possible skin lesions over the left hip
Cardiovascular	Tachycardia, hypotension
Respiratory	Tachypnea, hypoxia
Neurological	Altered mental status, seizures
Gastrointestinal	Nausea, vomiting, abdominal pain

5. Working Diagnoses:
- Septic arthritis of the left hip
- Gluteal intramuscular abscess

6. Relevant Investigations:

Investigation	Justification
Blood cultures	To identify the causative organism and determine antibiotic sensitivities

Left hip aspiration and culture	To confirm the diagnosis of septic arthritis and guide antibiotic therapy
Magnetic resonance imaging (MRI) of the left hip	To assess the extent of the infection and evaluate for complications
Complete blood count (CBC)	To monitor for anemia, leukocytosis, and thrombocytopenia
Electrolytes, creatinine, and liver function tests	To assess for electrolyte imbalances, renal function, and liver function
Urinalysis	To assess for urinary tract infection
Chest X-ray	To assess for pneumonia or other pulmonary complications

7. Management Plan:
- Start broad-spectrum antibiotics (e.g., vancomycin and cefepime) to cover common pathogens causing septic arthritis
- Administer pain medication (e.g., acetaminophen or nonsteroidal anti-inflammatory drugs) as needed
- Perform surgical drainage of the left hip abscess
- Provide supportive care, including intravenous fluids, electrolytes, and blood products as needed
- Monitor for complications, such as sepsis, DIC, and multi-organ failure

8. Executive Summary:
- The patient is a 67-year-old male with a history of hypertension, diabetes mellitus, hyperlipidaemia, left central retinal artery occlusion, bilateral hip heterotopic ossification, Streptococcus suis bacteraemia, meningitis, and MRSA bacteraemia.
- He was admitted to the hospital for evaluation of left hip pain and swelling.
- Contrast CT of the left hip revealed a multiloculated rim-enhancing collection worrisome for septic arthritis with gluteal intramuscular abscess.
- Left hip aspiration revealed elevated WBC, CRP, and ESR; culture is pending.
- The patient is afebrile and denies hip pain.
- The working diagnoses are septic arthritis of the left hip and gluteal intramuscular abscess.
- Investigations include blood cultures, left hip aspiration and culture, MRI of the left hip, CBC, electrolytes, creatinine, liver function tests, urinalysis, and chest X-ray.
- Management includes broad-spectrum antibiotics, surgical drainage of the left hip abscess, pain medication, supportive care, and monitoring for complications.

Claude 2

1. Chronological Events
Date	Event

May 2022	Streptococcus suis bacteraemia and meningitis, complicated by ventriculitis on MRI brain and recurrent seizure. Received EVD with vancomycin intraventricular irrigation.
October 2022	MRSA bacteraemia
November 2022	MRSA bacteraemia
December 2022	Gallium scan: No evidence of active infection.
28 November 2023	Admission for CT assessment of left hip
2 December 2023	Left hip aspiration performed

2. Clinical Problem List

Problem	Active/Chronic
Left ear deafness	Chronic
Hypertension	Chronic
Diabetes mellitus	Chronic
Hyperlipidemia	Chronic
Left CRAO	Chronic
Bilateral hip heterotopic ossification	Chronic
History of S. suis meningitis/ventriculitis	Active
History of MRSA bacteraemia	Active
Left hip septic arthritis	Active
Left hip intramuscular abscess	Active

3. Potential Life-Threatening Complications
- Sepsis/septic shock from uncontrolled left hip septic arthritis/abscess
- Recurrent CNS complications from prior meningitis - increased ICP, hydrocephalus, seizure
- End-organ damage from uncontrolled infection - renal failure, DIC

4. Anticipated Physical Examination Findings

System	Expected Findings
General	Febrile, ill-appearing
HEENT	No focal neurologic deficits
Cardiovascular	Tachycardia, hypotension (if septic)
Pulmonary	Tachypnea
Abdominal	Soft, non-tender
Musculoskeletal	Limited range of motion, pain with movement of left hip
Neurologic	No focal deficits

5. Working Diagnoses
- Septic arthritis of left hip
- Left gluteal intramuscular abscess

- Recurrent infection with MRSA

6. Relevant Investigations

Investigation	Justification
Blood cultures	To identify bacteremia
MRI pelvis	Further characterize extent of infection
Transthoracic echocardiogram	Evaluate for infectious endocarditis
Repeat aspiration of left hip	Confirm diagnosis and identify organism

7. Management Plan
- Admit to hospital for IV antibiotics
- Start empiric IV vancomycin to cover MRSA
- Orthopedic surgery consult for possible I&D of left hip joint and abscess
- Repeat imaging (MRI) to further characterize infection
- Blood cultures, repeat hip aspiration to identify organism
- Evaluate for endocarditis with TTE
- Monitor for sepsis and multi-organ failure
- Physical therapy for rehabilitation once infection cleared
- Follow CRP, ESR to monitor treatment response
- Long-term suppressive antibiotics may be needed given hardware

8. Executive Summary

67M with history of S. suis meningitis now admitted for left hip septic arthritis and abscess. Workup needed for recurrent MRSA infection. Immediate priority is IV antibiotics and source control. Must monitor for sepsis, CNS complications, end-organ damage from uncontrolled infection.

7. Clinical scenario 7

GPT-4.0

1. Chronological Events

Date	Event
October 2022	Diagnosis: Stage IV Mantle Cell Lymphoma
December 2022 - March 2023	Treatment: Hyper-CVAD-R regimen
June - July 2023	Treatment: Syphilis treated with doxycycline
30 August 2023	Procedure: Myeloablative Haplo-PBSCT
3 November 2023	Admission: Right ankle abscess; Incision and drainage performed
3 November 2023	Investigation: Wound swab culture, Nocardia species identified, sensitivity pending
6 November 2023	Investigation: Blood culture negative

9 November 2023	Investigation: CT thorax and brain, no brain abnormality, lung nodule and lymph nodes static, spleen enlarged
11 November 2023	Start Treatment: G-CSF
12 November 2023	Investigation: Blood culture and CXR pending/no consolidation

2. Clinical Problem List

Status	Clinical Issues
Active	Right ankle abscess
Active	Fever
Chronic	Stage IV Mantle Cell Lymphoma
Chronic	Post Myeloablative Haplo-PBSCT
Chronic	Cervical Intraepithelial Neoplasia
Chronic	History of Syphilis
Chronic	G6PD deficiency (in donor)
Chronic	Cytomegalovirus serostatus (donor sero-negative, recipient sero-positive)

3. Potential Life-Threatening Complications
- Sepsis from Nocardia species infection
- Neutropenic fever possibly leading to severe sepsis
- Complications of Graft-versus-host disease (GVHD) post-Haplo-PBSCT
- Relapse or progression of Mantle Cell Lymphoma

4. Clinical Findings

Organ System	Anticipated Findings
General	Fever
Cardiovascular	Blood pressure and pulse unsupported on room air
Respiratory	Chest clear on examination
Abdominal	Soft, non-tender abdomen
Musculoskeletal	Right ankle wound on dressing
Hematologic	Absolute neutrophil count borderline requiring G-CSF
Lymphatic	No palpable lymphadenopathy
Dermatologic	No other skin lesion detected
Neurologic	No signs of infection in the brain per CT

5. Working Diagnoses
- Right ankle abscess due to Nocardia species
- Post-transplantation complications including possible GVHD
- Neutropenia post Haplo-PBSCT
- Anemia and thrombocytopenia possibly related to chemotherapy or bone marrow suppression post-transplant

6. Relevant Investigations
Investigation	Justification
Wound swab culture sensitivity	To guide targeted antimicrobial therapy for Nocardia species
Blood culture	To rule out systemic infection or bacteremia
PET-CT	To assess potential malignancy activity and source of infection
Complete blood count	To monitor neutrophil count and assess for anemia and thrombocytopenia
Liver and renal function tests	To monitor organ function due to medication toxicity and disease process
Echocardiogram	To exclude infective endocarditis given the presence of Hickman catheter

7. Management Plan
- Continue monitoring and supportive care including G-CSF for neutropenia.
- Await sensitivity results and adjust antimicrobial therapy according to Nocardia species susceptibility.
- Manage fever and potential sepsis with broad-spectrum antibiotics until cultures and sensitivities are finalized.
- Monitor for signs of GVHD and manage as appropriate.
- Supportive care for anemia and thrombocytopenia, including possible transfusions.
- Close monitoring of renal and liver function due to ongoing medication regimen.
- Consider consultation with Infectious Disease and Hematology for multidisciplinary approach.

8. Executive Summary
A 53-year-old female with a history of Stage IV Mantle Cell Lymphoma post Hyper-CVAD-R treatment and recent Haplo-PBSCT presents with a right ankle abscess and fever. The abscess is due to Nocardia species, with sensitivity pending, and she is currently on broad-spectrum antibiotics. The patient has neutropenia requiring G-CSF and is post-transplant, which necessitates close monitoring for potential GVHD. A comprehensive management plan focusing on infection control, supportive care for hematologic abnormalities, and post-transplant complications is recommended. Coordination with a multidisciplinary team is essential for her complex care.

cGPT-4

1. Chronological Events:
Date	Major Clinical Issue
October 2022	Diagnosed with Stage IV Mantle cell lymphoma

December 2022 - March 2023	Treatment with HyperCVAD-R regimen
June - July 2023	Treated for syphilis with doxycycline
30 August 2023	Myeloablative Haplo-PBSCT
3 November 2023	Admission for right ankle abscess, Incision and drainage
9 November 2023	CT thorax and brain with contrast
12 November 2023	Blood culture taken, CXR performed

2. Clinical Problem List:
Status	Clinical Issues
Active	Right ankle abscess
Active	Fever
Active	Neutropenia (borderline ANC, requiring G-CSF)
Chronic	Stage IV Mantle cell lymphoma
Chronic	Cervical intraepithelial neoplasia
Chronic	History of syphilis
Chronic	Cyclosporin post-transplant

3. Potential Life-Threatening Complications:
- Untreated or unresolved abscess could lead to systemic infection or sepsis.
- Neutropenia increases the risk of severe infections that can be life-threatening, especially in post-transplant patients with a compromised immune system.
- Relapse or progression of Mantle cell lymphoma could be life-threatening.

4. Clinical Findings:
Organ System	Anticipated Examination Findings
General	Fever
Cardiovascular	Blood pressure and pulse unsupported on room air
Respiratory	Chest clear
Abdomen	Soft, non-tender
Musculoskeletal	Right ankle wound on dressing
Skin	No additional skin lesions noted on lower limbs
Neurological	No abnormalities in the brain detected by CT scan

5. Working Diagnoses:
- Right ankle abscess due to Nocardia species.
- Neutropenia post peripheral blood stem cell transplantation.
- Fever of unknown origin in an immunocompromised host.
- Spleen size increase, suggesting possible infection or lymphoma involvement.

6. Relevant Investigations:
| Investigation | Justification |

|---------------|---------------|
Wound swab culture and sensitivity	To guide targeted antimicrobial therapy for the ankle abscess
Blood cultures	To identify possible bloodstream infection or sepsis
CT thorax and brain with contrast	To exclude metastasis or other sources of infection
PET-CT	To evaluate for potential lymphoma activity or other malignancies
Complete blood count	To monitor for neutropenia and other hematological abnormalities

7. Management Plan:
- Continue current antimicrobial therapy with imipenem-cilastatin and amikacin until culture results are available.
- Monitor absolute neutrophil count and continue G-CSF as needed to manage neutropenia.
- Perform incision and drainage of abscess if required, and adjust antibiotics based on sensitivity results.
- Close monitoring for signs of sepsis or other infections.
- Prepare for initiation of appropriate treatment if relapse of lymphoma is identified on PET-CT.

8. Executive Summary:
The patient is a 53-year-old female with a history of Stage IV Mantle cell lymphoma, currently with an active right ankle abscess and fever. She is in a post-transplant state and experiencing borderline neutropenia. Immediate concerns include managing the abscess and preventing sepsis. Future diagnostics include a pending PET-CT to assess for lymphoma activity. The management plan includes continued antimicrobials, G-CSF support, and close monitoring for complications.

Gemini Pro

1. Chronological Events:
Date	Event
October 2022	Stage IV Mantle cell lymphoma diagnosis
December 2022 - March 2023	HyperCVAD-R regimen
30 August 2023	Myeloablative Haplo-PBSCT
June - July 2023	Syphilis treatment with doxycycline
3 November 2023	Admission to Hospital N for right ankle abscess
3 November 2023	Incision and drainage of right ankle abscess
6 November 2023	Blood culture
9 November 2023	CT thorax and brain with contrast
11 November 2023	Administration of G-CSF

12 November 2023	Blood culture
12 November 2023	CXR

2. Clinical Problem List:

Active	Chronic
Right ankle abscess	Stage IV Mantle cell lymphoma
Nocardia species infection	Syphilis
Neutropenia	Cervical intraepithelial neoplasia

3. Potential Life-Threatening Complications:
* Sepsis
* Disseminated Nocardia infection
* Hematologic complications (e.g., severe anemia, thrombocytopenia)
* Graft-versus-host disease

4. Clinical Findings:

Organ System	Anticipated Findings
General	Fever, tachycardia, hypotension
Respiratory	Clear lungs
Cardiovascular	Normal blood pressure, no signs of infective endocarditis
Gastrointestinal	Soft, non-tender abdomen
Musculoskeletal	Right ankle wound, no palpable lymphadenopathy
Integumentary	No skin lesions
Neurological	Normal mental status

5. Working Diagnoses:
* Nocardia species right ankle abscess
* Nocardia species pneumonia (possible)
* Nocardia species disseminated infection (possible)

6. Relevant Investigations:

Investigation	Justification
Blood cultures	To identify the causative organism and guide antimicrobial therapy
Sputum culture	To identify the causative organism and guide antimicrobial therapy
Chest X-ray	To assess for pulmonary involvement
CT scan of the chest and abdomen	To assess for disseminated infection
PET-CT scan	To assess for disseminated infection and evaluate the response to therapy

7. Management Plan:

* Continue empiric antimicrobial therapy with imipenem-cilastatin and amikacin |
* Monitor for signs and symptoms of disseminated infection |
* Perform PET-CT scan to evaluate the extent of infection |
* Provide supportive care, including fluid resuscitation, oxygen therapy, and blood product transfusions as needed |
* Consider surgical debridement of the right ankle abscess if necessary |

8. Executive Summary:
The patient is a 53-year-old female with a history of Stage IV mantle cell lymphoma who presented with a right ankle abscess caused by Nocardia species. She is currently neutropenic due to recent myeloablative haploidentical peripheral blood stem cell transplantation. The patient is being treated with empiric antimicrobial therapy and is being monitored for signs and symptoms of disseminated infection. A PET-CT scan will be performed to evaluate the extent of infection.

Claude 2

1. Chronological Events

Date	Event
October 2022	Diagnosed with stage IV Mantle cell lymphoma
December 2022 to March 2023	Received HyperCVAD-R chemotherapy regimen
30 August 2023	Underwent haploidentical PBSCT
June to July 2023	Treated for syphilis with 4 weeks of doxycycline
3 November 2023	Admitted for right ankle abscess, incision and drainage done
6 November 2023	Blood culture negative
7 November 2023	Started on imipenem-cilastatin and amikacin
9 November 2023	CT thorax and brain showed no new lesions
11 November 2023	Received G-CSF for borderline neutropenia
12 November 2023	Ongoing fever, pending blood culture and CXR

2. Clinical Problem List

Status	Clinical Problems
Active	Febrile neutropenia, right ankle nocardial abscess
Chronic	Stage IV mantle cell lymphoma s/p haploidentical PBSCT, CMV seropositivity, cervical intraepithelial neoplasia

3. Potential Life-Threatening Complications
- Sepsis/septic shock from nocardial infection
- Respiratory failure from pneumonia
- Intracranial spread of nocardial infection
- Bleeding from thrombocytopenia

4. Clinical Findings

Organ System	Expected Findings
Cardiovascular	Tachycardia
Respiratory	Normal breath sounds
Abdominal	Soft, non-tender
Lymphoreticular	Generalized lymphadenopathy
Musculoskeletal	Right ankle wound
Neurological	Normal mental status

5. Working Diagnoses
- Febrile neutropenia
- Suspected nocardial bacteremia
- Disseminated nocardiosis
- Right ankle abscess
- Rule out pneumonia
- Post hematopoietic stem cell transplant
- On immunosuppression

6. Relevant Investigations

Investigation	Rationale
Blood cultures	Diagnose bacteremia
Imaging - CXR, CT chest	Assess for pulmonary nocardiosis
Tissue culture of ankle wound	Confirm Nocardia and determine antimicrobial susceptibilities
Serum galactomannan	Rule out invasive aspergillosis
Cyclosporine drug level	Ensure therapeutic level, avoid toxicity

7. Management Plan
- Continue imipenem-cilastatin and amikacin to cover for likely nocardiosis
- Daily monitoring of blood counts, kidney function, electrolytes
- Obtain susceptibilities of Nocardia isolate to guide targeted antimicrobial therapy
- Avoid aminoglycosides if kidney function declines
- Consult ID for addition of TMP-SMX or linezolid depending on susceptibilities
- If fevers persist, evaluate for other sites of infection e.g. lungs, CNS
- Transfuse pRBCs for anemia and platelets for bleeding prophylaxis
- Follow up on PET-CT to assess lymphoma status
- Discharge planning: ensure close outpatient follow-up once stable

8. Executive Summary

53F s/p haploidentical PBSCT in Aug 2023 for mantle cell lymphoma, now admitted for right ankle nocardial abscess and febrile neutropenia. She requires broad spectrum antibiotics, further workup for disseminated nocardiosis, supportive care, and close monitoring for complications. Treatment will be guided by antimicrobial susceptibilities. Long term lymphoma surveillance is needed.

8. Clinical scenario 8

GPT-4.0

1. Chronological Events:

Date	Event
April 2023	Diagnosis of B-cell acute lymphoblastic leukemia (B-ALL).
April-August 2023	Treatment with HyperCVAD regimen plus dasatinib.
15 August 2023	Bone marrow examination showing regenerating marrow with mildly left-shifted granulopoiesis. No excessive blasts.
28 September 2023	Peripheral Blood Stem Cell Transplantation (PBSCT). Donor sero-negative, recipient sero-positive for cytomegalovirus. Cytokine Release Syndrome grade 1, treated with tocilizumab.
10 October 2023	Development of fever, started on Meropenem.
After 10 October 2023	Development of generalized morbilliform rash, suspected drug eruption, switched to levofloxacin. Skin biopsy done, started on steroids and MMF for GvHD.
Day 15 post-PBSCT	Current physical examination findings.

2. Clinical Problem List:

Status	Clinical Issues
Active	Fever post-PBSCT
Active	Generalized morbilliform rash
Active	Cytokine Release Syndrome (resolved with tocilizumab)
Chronic	B-cell acute lymphoblastic leukemia (B-ALL)
Chronic	CMV sero-positive status

3. Potential Life-Threatening Complications:
- Severe infection due to immunosuppression post-PBSCT.
- Severe Graft-versus-host disease (GvHD).
- Relapse of B-cell acute lymphoblastic leukemia (B-ALL).

4. Clinical Findings:

Organ System	Findings
General	Fever, generalized morbilliform rash, facial swelling.

Cardiovascular	Blood pressure and pulse unsupported.
Respiratory	Chest clear, on room air.
Gastrointestinal	Abdomen soft non-tender.
Musculoskeletal	Calves are soft, no lower limb edema.
Integumentary	Hickman line insertion site non-tender with no surrounding erythema.
Neurological	Neurologically well.

5. Working Diagnoses:
- Graft-versus-host disease (GvHD).
- Adverse cutaneous drug reaction (ACDR).
- Post-transplant infectious complications (etiologies to be determined from pending cultures and antigen tests).

6. Relevant Investigations:

Investigation	Justification
Complete blood count (CBC)	To monitor for cytopenias.
Serum creatinine & electrolytes	To assess renal function.
Liver function tests (LFTs)	To monitor for hepatic involvement in GvHD or drug-induced liver injury.
Procalcitonin & CRP	Biomarkers for systemic inflammation or infection.
CXR	To rule out pulmonary infections.
Skin biopsy	To differentiate between GvHD and drug reaction.
Blood, urine, stool cultures	To identify potential infectious agents.
Aspergillus and Cryptococcus antigen tests	To exclude opportunistic fungal infections.

7. Management Plan:
- Continue monitoring vital signs and clinical status closely.
- Maintain broad-spectrum antimicrobials until infectious etiologies are excluded or specifically identified.
- Manage GvHD with steroids and MMF, adjusting based on skin biopsy results and clinical response.
- Supportive care including transfusions for anemia or thrombocytopenia as indicated by CBC results.
- Implement prophylactic measures for potential opportunistic infections due to immunosuppression.
- Evaluate the need for antifungal therapy continuation based on antigen test results.
- Consider consultation with infectious disease specialists for complex antimicrobial management.

8. Executive Summary:

The 33-year-old female patient with a history of B-ALL has undergone PBSCT and developed fever and a rash, raising concerns for GvHD and drug reactions. Management involves broad-spectrum antibiotics, steroids, and MMF for suspected GvHD, with close monitoring. Investigations are ongoing to rule out infectious etiologies. The patient's care requires a multi-disciplinary approach, including hematology, dermatology, and infectious disease specialists to address the complex interplay of post-transplant complications.

cGPT-4

1. Chronological Events:

Date	Clinical Event
April 2023	Diagnosis of B-cell acute lymphoblastic leukemia (B-ALL)
April - August 2023	Treatment with Hyper-CVAD regimen plus dasatinib
15 August 2023	Bone marrow examination showing regenerating marrow
28 September 2023	Peripheral Blood Stem Cell Transplantation (PBSCT)
1 October 2023	Cytokine Release Syndrome grade 1, treated with tocilizumab
10 October 2023	Fever onset, treatment with Meropenem
Post-PBSCT	Development of morbilliform rash
Following rash development	Consultation with Dermatology, suspected drug eruption
Post rash treatment	Switch to levofloxacin, skin biopsy, and initiation of steroids and MMF for GvHD

2. Clinical Problem List:

Status	Clinical Issues
Active	Fever, morbilliform rash, possible GvHD, possible drug eruption
Chronic	B-cell acute lymphoblastic leukemia (B-ALL)

3. Potential Life-Threatening Complications:
- Cytokine Release Syndrome: Though currently grade 1, it can escalate causing severe systemic inflammation.
- Graft-versus-Host Disease (GvHD): Can lead to severe organ dysfunction.
- Infection: Given the immunocompromised state, any infection can rapidly become life-threatening.

4. Clinical Findings:

Organ System	Findings
General	Fever, morbilliform rash

Cardiovascular	Blood pressure and pulse unsupported
Respiratory	Chest clear, on room air
Gastrointestinal	Abdomen soft, non-tender
Musculoskeletal	Calves soft, no lower limb edema
Integumentary	Generalized morbilliform rash, no active discharge
Neurological	Neurologically well
Vascular Access	Hickman line insertion site non-tender, no erythema

5. Working Diagnoses:
- Graft-versus-Host Disease (GvHD)
- Adverse Cutaneous Drug Reaction (ACDR)
- Persistent post-transplantation fever, possible infection

6. Relevant Investigations:

Investigation	Justification
Skin biopsy	To differentiate between GvHD and drug reaction
Blood cultures	To identify possible bloodstream infection
Urine and stool cultures	To rule out enteric or urinary sources of infection
Aspergillus and Cryptococcus antigens	To exclude opportunistic fungal infections
Procalcitonin and CRP levels	To evaluate the extent of inflammation and infection

7. Management Plan:
- Continue monitoring vitals and clinical signs of GvHD or infection.
- Manage fever and potential infections with broad-spectrum antimicrobials while awaiting culture results.
- Adjust immunosuppression to manage suspected GvHD with steroids and MMF.
- Consider antifungal prophylaxis due to immunosuppressed status and potential exposure to Aspergillus or Cryptococcus.
- Provide supportive care including transfusions for anemia and thrombocytopenia as indicated by lab results.
- Regular follow-up with Hematology and Dermatology for the management of leukemia and skin findings.

8. Executive Summary:
The patient is a 33-year-old female with B-ALL post-PBSCT presenting with fever and a morbilliform rash, indicating potential GvHD or drug reaction. Immediate attention is focused on managing the cytokine release syndrome and preventing life-threatening complications such as severe infection or organ dysfunction due to GvHD. Investigations including skin biopsy and cultures are underway to guide treatment, which currently includes broad-spectrum antimicrobials, immunosuppressants, and supportive care. Close monitoring and interdisciplinary collaboration are imperative for her ongoing management.

Gemini Pro

1. Chronological Events:

Date	Event
April 2023	B-cell acute lymphoblastic leukemia (B-ALL) diagnosed.
April to August 2023	Hyper-cyclophosphamide, vincristine, Adriamycin, and dexamethasone (HyperCVAD) regimen plus dasatinib given.
15 August 2023	Latest bone marrow examination showed regenerating marrow post-chemotherapy with mildly left-shifted granulopoiesis and no excessive blasts.
28 September 2023	Peripheral Blood Stem Cell Transplantation (PBSCT) performed.
29 September 2023	Cotrimoxazole and Meropenem started.
1 October 2023	Cytokine Release Syndrome grade 1 on day 1 given tocilizumab.
10 October 2023	Fever developed, Meropenem stopped and levofloxacin started.
11 October 2023	Urine culture showed Enterococcus faecium 1500 CFU/ml.
12 October 2023	Posaconazole stopped and micafungin started.
13 October 2023	Chest X-ray (CXR) showed no obvious consolidation.

2. Clinical Problem List:

Active	Chronic
Fever	B-cell acute lymphoblastic leukemia (B-ALL)
Generalized morbilliform rash	
Neutropenia	
Thrombocytopenia	
Enterococcus faecium bacteriuria	

3. Potential Life-Threatening Complications:
* Sepsis
* Graft-versus-host disease (GvHD)
* Invasive fungal infection

4. Clinical Findings:

Organ System	Anticipated Physical Examination Findings
General	Swinging fever, facial swelling
Respiratory	Chest clear
Cardiovascular	Blood pressure and pulse unsupported
Gastrointestinal	Abdomen soft non-tender
Musculoskeletal	Calves are soft, no lower limb edema
Skin	Generalized morbilliform rash over thigh, upper limb, chest, trunk and back
Neurological	Neurologically well

5. Working Diagnoses:
* Neutropenic fever
* Enterococcus faecium bacteriuria
* Drug eruption
* Graft-versus-host disease (GvHD)

6. Relevant Investigations:

Investigation	Justification
Complete blood count (CBC) with differential	To monitor hematologic status
Serum electrolytes, creatinine, and liver function tests	To assess organ function
Blood cultures	To identify bloodstream infection
Urine culture and sensitivity	To identify urinary tract infection
Stool culture	To identify gastrointestinal infection
Aspergillus antigen and Cryptococcus antigen	To rule out invasive fungal infection
Skin biopsy	To confirm the diagnosis of drug eruption or GvHD

7. Management Plan:
* Continue empiric antibiotics for neutropenic fever until culture results are available.
* Start broad-spectrum antibiotics for Enterococcus faecium bacteriuria.
* Continue steroids and Mycophenolate mofetil (MMF) for treatment of GvHD.
* Monitor for signs and symptoms of invasive fungal infection.
* Provide supportive care, including fluids, electrolytes, and blood products as needed.

8. Executive Summary:
The patient is a 33-year-old female with B-cell acute lymphoblastic leukemia (B-ALL) who underwent Peripheral Blood Stem Cell Transplantation (PBSCT) on 28 September 2023. She developed neutropenic fever, Enterococcus faecium bacteriuria, and a generalized morbilliform rash. The working diagnoses are neutropenic fever, Enterococcus faecium bacteriuria, drug eruption, and Graft-versus-host disease (GvHD). The management plan includes empiric antibiotics, broad-spectrum antibiotics for Enterococcus faecium bacteriuria, steroids and Mycophenolate mofetil (MMF) for treatment of GvHD, and monitoring for signs and symptoms of invasive fungal infection.

Claude 2

1. Chronological Events

Date	Event
April 2023	Diagnosed with B-cell acute lymphoblastic leukemia (B-ALL)
April to August 2023	Treated with HyperCVAD plus dasatinib

15 August 2023	Bone marrow examination showed regenerating marrow post-chemotherapy with no excessive blasts
28 September 2023	Underwent peripheral blood stem cell transplantation (PBSCT)
1 October 2023	Developed cytokine release syndrome, treated with tocilizumab
10 October 2023	Developed fever
11 October 2023	Developed generalized morbilliform rash, suspected to be drug eruption
13 October 2023	Skin biopsy performed, started on steroids and MMF for GVHD treatment

2. Clinical Problem List

Problem	Status
B-cell ALL	Chronic
PBSCT	Active
Cytokine release syndrome	Active
Fever	Active
Generalized morbilliform rash	Active
Potential GVHD	Active

3. Potential Life-Threatening Complications
- Sepsis
- Disseminated fungal infection
- Severe GVHD

4. Clinical Findings

System	Expected Findings
General	Fever, facial swelling
Respiratory	Clear on auscultation
Cardiovascular	Tachycardia
Abdominal	Soft, non-tender
Extremities	Generalized morbilliform rash, no edema
Neurological	Well

5. Working Diagnoses
- Febrile neutropenia
- Drug eruption
- Graft-versus-host disease
- Invasive fungal infection

6. Relevant Investigations

Investigation	Rationale

Blood cultures	To identify any bacteremia or fungemia
Antimicrobial sensitivities	To guide antibiotic therapy
Aspergillus antigen	Screen for invasive aspergillosis
Cryptococcus antigen	Screen for disseminated cryptococcal infection
Skin biopsy	Diagnose etiology of rash
Bronchoalveolar lavage	Rule out pulmonary fungal infection if persistent fevers

7. Management Plan
- Continue levofloxacin to cover neutropenic fever pending cultures
- Micafungin for empiric antifungal coverage
- Topical steroids for skin rash
- Increase hydration to maintain urine output > 0.5 ml/kg/hr
- Daily monitoring of CBC, renal function, LFTs
- Transfuse pRBCs for Hb < 7 g/dL, platelets for counts < 10 x 10^9/L
- Consider G-CSF if prolonged neutropenia
- Frequent skin inspection for progression of rash
- Consider skin biopsy if rash worsening despite steroids
- Bronchoalveolar lavage if cultures negative and fevers persist > 72 hours

8. Executive Summary
33F s/p PBSCT for B-ALL with morbilliform rash, fevers and neutropenia concerning for drug eruption, invasive fungal infection and GVHD. Recommend continuing antibiotics, antifungals, hydration, transfusion support, and steroids while monitoring counts, cultures, rash progression. Further tests like skin biopsy and BAL may be indicated.

9. Clinical scenario 9

GPT-4.0

1. Chronological Events

Date	Event
Not specified	Diagnosis of coronary artery disease; PCI performed
Not specified	Diagnosis of atrial fibrillation; started on dabigatran
Not specified	Diagnosis of gastroesophageal reflux disease (GERD)
Not specified	Iron deficiency anaemia diagnosed; not for endoscopy
Not specified	History of traumatic subdural haematoma (SDH)
Not specified	Admission to old aged home
25 November 2023	Bilateral groin superficial wound swab: Diphtheroid bacilli, MRSA, MDRA
Not specified	Scald injury by hot soup over inner thighs; 2% TBSA
Coming Monday	Planned emergency operation

2. Clinical Problem List

Status	Clinical Issue
Chronic	Coronary artery disease with PCI
Chronic	Atrial fibrillation on dabigatran
Chronic	Gastroesophageal reflux disease (GERD)
Chronic	Iron deficiency anaemia
Chronic	History of traumatic subdural haematoma (SDH)
Active	Scald injury by hot soup (~2% TBSA)
Active	MDRO screening positive for MDRA and MRSA

3. Potential Life-Threatening Complications
- Sepsis due to MDRO (MDRA, MRSA) colonization, particularly in the context of an acute burn wound.
- Acute renal failure due to possible sepsis and pre-existing compromised creatinine clearance.
- Exacerbation of underlying coronary artery disease due to stress from the burn injury and potential surgery.
- Hemorrhagic complications or stroke due to anticoagulation therapy (dabigatran) in the setting of an upcoming emergency operation.

4. Clinical Findings

Organ System	Anticipated Findings
Cardiovascular	Irregular rhythm due to atrial fibrillation
Integumentary	Localized burn wound on inner thighs
Hematological	Signs of anaemia
Neurological	Any sequelae from previous subdural haematoma (SDH)

5. Working Diagnoses
- Scald injury with deep dermal involvement (~2% TBSA)
- Methicillin-resistant Staphylococcus aureus (MRSA) colonization
- Multi-Drug Resistant Acinetobacter (MDRA) colonization
- Chronic coronary artery disease with previous PCI
- Atrial fibrillation on dabigatran
- Gastroesophageal reflux disease (GERD)
- Iron deficiency anaemia
- History of traumatic subdural haematoma (SDH)

6. Relevant Investigations

Investigation	Justification

Complete Blood Count (CBC)	To monitor for signs of infection or worsening anaemia
Blood cultures	To rule out systemic infection given positive MDRO colonization and prior to surgery
Renal function tests (Repeat sCr and CrCl)	To monitor renal function pre-operatively and assess for any acute changes
Liver function tests (LFTs)	To ensure liver function is adequate for potential anesthesia and drug metabolism
ECG	To monitor atrial fibrillation and cardiac status
Wound culture of burn area	To identify any infection in the burn wound
Echocardiogram	To assess cardiac function pre-operatively

7. Management Plan
- Burn Care: Strict aseptic technique for wound care to prevent infection, pain management, and fluid resuscitation as needed.
- Surgical Evaluation: Review by the burn team and surgical team to assess the need for debridement or skin grafting.
- Antibiotic Stewardship: Consultation with infectious disease specialists for potential prophylactic or pre-operative antibiotic therapy, considering MRSA and MDRA colonization.
- Cardiac Management: Cardiology consult for perioperative management of atrial fibrillation and coronary artery disease. Adjustments to dabigatran therapy in the context of surgery.
- Anemia Management: Evaluation for the need for iron supplementation or transfusion prior to surgery.
- Renal Function Monitoring: Close monitoring of renal function and adjustment of medications accordingly.
- MDRO Management: Implement contact precautions to prevent spread of MDROs, and possibly decolonization strategies.

8. Executive Summary
An 89-year-old male with a complex medical history including coronary artery disease, atrial fibrillation, GERD, iron deficiency anaemia, and a history of SDH, is currently admitted with a 2% TBSA scald injury and positive MDRO colonization. Immediate life-threatening complications include potential sepsis and renal failure, exacerbated by the patient's cardiac conditions and upcoming emergency surgery. Management should focus on meticulous burn care, pre-operative optimization, and strict infection control measures. A multidisciplinary team approach involving surgery, cardiology, infectious diseases, and possibly hematology is essential to navigate the perioperative period effectively. The patient's advanced age and comorbidities necessitate careful monitoring and proactive management to prevent complications.

cGPT-4

1. Chronological Events:

Date	Event
Past	PCI for coronary artery disease
Past	Diagnosis of atrial fibrillation; initiated on dabigatran
Past	Diagnosis of GERD
Past	Diagnosis of iron deficiency anaemia; decision against endoscopy
Past	Traumatic subdural haematoma
Current Admission Date	Scald injury by hot soup over inner thighs; ~2% TBSA deep dermal injury
Planned Date	Emergency operation scheduled for coming Monday

2. Clinical Problem List:

Status	Clinical Issue
Chronic	Coronary artery disease with PCI
Chronic	Atrial fibrillation on dabigatran
Chronic	Gastroesophageal reflux disease (GERD)
Chronic	Iron deficiency anaemia
Chronic	History of traumatic subdural haematoma (SDH)
Active	Scald injury by hot soup

3. Potential Life-Threatening Complications:
- Infection of the scald injury, especially given the patient's colonization with MDRA and MRSA.
- Complications related to atrial fibrillation, such as stroke.
- Anemia-related complications, potentially worsening cardiac issues.
- Risks associated with emergency surgery, given the patient's advanced age and comorbidities.

4. Clinical Findings:

Organ System	Anticipated Findings
Integumentary	Deep dermal injury to inner thighs
Cardiovascular	Findings consistent with coronary artery disease and atrial fibrillation
Hematologic	Signs of iron deficiency anemia
Neurologic	Findings related to history of subdural haematoma, if any

5. Working Diagnoses:
- Deep dermal burn injury due to scalding.
- Coronary artery disease post-PCI.
- Atrial fibrillation on anticoagulation therapy.

- Gastroesophageal reflux disease.
- Iron deficiency anemia.
- Carriage of MDRA and MRSA.

6. Relevant Investigations:

Investigation	Rationale
Wound culture	To assess for infection of the scald injury and guide antimicrobial therapy
CBC	To monitor for anemia or signs of infection
ECG	To monitor atrial fibrillation status
Liver and renal function tests	To evaluate baseline organ function pre-operatively and for drug dosing

7. Management Plan:
- Continue to monitor wound closely for signs of infection, given the MDRA and MRSA colonization.
- Follow standard burn care protocols, ensuring adequate pain management and wound care.
- Ensure appropriate anticoagulation management pre- and post-operation, balancing the risk of bleeding with thrombosis.
- Monitor hemoglobin levels and manage iron deficiency anemia accordingly, possibly with iron supplementation.
- Proactively manage GERD to prevent potential exacerbation due to stress of injury and upcoming surgery.
- If surgery is to proceed, involve a multidisciplinary team to manage the complex needs of the patient, including cardiology, surgery, and infectious disease specialists.

8. Executive Summary:
The elderly patient with a history of significant cardiac issues and a traumatic brain injury has been admitted for a scald injury requiring surgical intervention. The patient is at risk of infection due to colonization with MDRA and MRSA, and careful monitoring and management are crucial. A comprehensive plan addressing wound care, anticoagulation management, anemia, and GERD is recommended to mitigate potential complications. Multidisciplinary collaboration is essential for the upcoming surgery and overall care.

Please note that the above analysis and recommendations are based on the limited clinical information provided and may need to be adjusted with more patient data or as the clinical situation evolves.

Gemini Pro

1. Chronological Events

Date	Event
25 November 2023	Bilateral groin superficial wound swab: Diphtheroid bacilli, MRSA, MDRA
Not specified	History of traumatic subdural haematoma (SDH)
Not specified	Coronary artery disease with percutaneous coronary intervention (PCI)
Not specified	Atrial fibrillation on dabigatran
Not specified	Gastroesophageal reflux disease (GERD)
Not specified	Iron deficiency anaemia not for endoscopy
Not specified	Scald injury by hot soup over inner thighs, ~2% total body surface area (TBSA) deep dermal injury
Not specified	Emergency operation planned for coming Monday

2. Clinical Problem List

Active	Chronic
Scald injury	Coronary artery disease
Atrial fibrillation	Gastroesophageal reflux disease
Iron deficiency anaemia	
Multi-Drug Resistant Acinetobacter (MDRA)	
Methicillin-resistant Staphylococcus aureus (MRSA)	

3. Potential Life-Threatening Complications
* Sepsis
* Disseminated intravascular coagulation (DIC)
* Multi-organ failure
* Cardiac arrhythmias
* Stroke

4. Clinical Findings

Organ System	Anticipated Physical Examination Findings
Cardiovascular	Regular rate and rhythm, no murmurs, rubs, or gallops
Respiratory	Clear to auscultation bilaterally, no wheezes, rales, or rhonchi
Gastrointestinal	Soft, non-tender abdomen, no hepatosplenomegaly
Neurological	Alert and oriented, no focal neurological deficits
Musculoskeletal	Examination of the burn wound reveals erythema, edema, and blistering
Integumentary	Bilateral groin superficial wound swab: Diphtheroid bacilli, MRSA, MDRA

5. Working Diagnoses
* Scald injury
* Atrial fibrillation
* Coronary artery disease
* Gastroesophageal reflux disease
* Iron deficiency anaemia
* Multi-Drug Resistant Acinetobacter (MDRA) colonization
* Methicillin-resistant Staphylococcus aureus (MRSA) colonization

6. Relevant Investigations

Investigation	Justification
Complete blood count (CBC)	To assess for anemia, infection, and inflammation
Comprehensive metabolic panel (CMP)	To assess for electrolyte abnormalities, renal function, and liver function
Blood culture	To rule out sepsis
Wound culture	To identify the causative organism and guide antibiotic therapy
Electrocardiogram (ECG)	To assess for arrhythmias
Echocardiogram	To assess for structural heart disease
Colonoscopy	To evaluate for the cause of iron deficiency anaemia

7. Management Plan
* Scald injury:
* Clean and dress the wound daily.
* Apply silver sulfadiazine cream to the wound.
* Monitor for signs of infection.
* Administer pain medication as needed.
* Atrial fibrillation:
* Continue dabigatran.
* Monitor for signs of bleeding.
* Coronary artery disease:
* Continue aspirin and statin therapy.
* Monitor for signs of angina.
* Gastroesophageal reflux disease:
* Administer proton pump inhibitor (PPI).
* Recommend lifestyle modifications, such as avoiding caffeine and alcohol.
* Iron deficiency anaemia:
* Administer oral iron supplements.
* Investigate the cause of iron deficiency anaemia.
* Multi-Drug Resistant Acinetobacter (MDRA) colonization:
* Contact precautions.
* Monitor for signs of infection.
* Methicillin-resistant Staphylococcus aureus (MRSA) colonization:

* Contact precautions.
* Monitor for signs of infection.

8. Executive Summary
An 89-year-old male with a history of coronary artery disease, atrial fibrillation, gastroesophageal reflux disease, iron deficiency anaemia, and scald injury is admitted for emergency surgery. He is colonized with MDRA and MRSA. The patient's management plan includes wound care, antibiotics, and supportive care.

Claude 2

1. Chronological Events
Date	Event
Unknown	89/M with medical history of CAD s/p PCI, AF on dabigatran, GERD, iron deficiency anemia, traumatic SDH
Current admission	Admitted for scald injury to inner thighs, 2% TBSA deep dermal burn, plan for emergency surgery on Monday

2. Clinical Problem List
Active Problems	Chronic Problems
Scald burn injury to inner thighs, 2% TBSA deep dermal burn	CAD s/p PCI, AF on dabigatran, GERD, iron deficiency anemia, traumatic SDH

3. Potential Life-Threatening Complications
- Sepsis from burn wound infection
- Exsanguination from burn wound bleeding

4. Clinical Findings
Cardiovascular	Normal heart sounds, irregularly irregular rhythm, no murmurs
Respiratory	Clear to auscultation bilaterally, normal work of breathing
Abdominal	Soft, non-tender, no masses
Extremities	Scald injury to inner thighs with 2% TBSA deep dermal burn
Neurological	Awake, alert, orientated x3

5. Working Diagnoses
- Deep dermal burn injury to inner thighs
- MRSA and MDRA colonization
- Atrial fibrillation
- CAD s/p PCI
- GERD

- Iron deficiency anemia
- Traumatic SDH

6. Relevant Investigations

Investigation	Justification
Wound culture	Evaluate for wound infection
Blood cultures	Evaluate for bacteremia/sepsis
CBC	Evaluate infection/anemia
BMP	Evaluate renal function for antibiotic dosing
CRP	Marker of inflammation to monitor infection
ECG	Document cardiac rhythm, evaluate for ischemia
CXR	Evaluate for pulmonary process from infection

7. Management Plan
- IV antibiotics to cover MRSA and MDRA
- Strict hand hygiene and contact precautions
- Consult burn surgery for debridement/grafting
- Analgesia for wound pain
- DVT prophylaxis
- Maintain euvolemia
- Monitor for signs of sepsis
- Address goals of care given advanced age

8. Executive Summary
89/M with deep dermal burn injury to inner thighs admitted for emergency surgery on Monday. Actively monitor for wound infection and bleeding given MRSA/MDRA colonization. Address goals of care given advanced age.

10. Clinical scenario 10

GPT-4.0

1. Chronological Events

Date	Event
2019	History of caecal tumour; right hemicolectomy; lesion deemed benign
August 2023	History of Covid-19
September 2023	History of red eye
11 October 2023	Admitted to Hospital N for chest pain; elevated troponin I; managed as acute coronary syndrome
13 October 2023	Presented with swinging fever; later subsided
14 October 2023	Blood culture taken (no growth); first dose of Augmentin given post-culture

18 October 2023	Coronary angiogram showed mild coronary artery stenosis
24 October 2023	Transesophageal echocardiogram detected a 2 mm perforation in non-coronary cusp with severe AR; start of investigation for infective endocarditis
24-25 October 2023	Three sets of blood cultures taken; IV vancomycin started
5-9 November 2023	Developed fever with chills and rigor; Piperacillin-tazobactam added to treatment
9 November 2023	New onset cytopenia and skin rash; switched vancomycin to daptomycin
15 November 2023	CT abdomen and pelvis showed non-specific lesion in segment 4b
17 November 2023	Discharged against medical advice from Hospital N; admitted to Hospital Q for further management
18 November 2023	Started on IV ceftriaxone
6-17 November 2023	Received IV piperacillin-tazobactam
9-15 November 2023	Received IV daptomycin
24 October - 9 November 2023	Received IV vancomycin
22-23 October 2023	Antibiotic-free period
16-21 October 2023	Oral Amoxicillin-clavulanate
14-16 October 2023	IV Amoxicillin-clavulanate
Ongoing since 13 November 2023	Persistent fever

2. Clinical Problem List

Status	Clinical Issues
Active	Acute coronary syndrome
Active	Suspected infective endocarditis
Active	Severe aortic regurgitation
Active	Fever with chills and rigor
Active	New onset cytopenia
Active	Persistent fever since 13 November 2023
Chronic	History of caecal tumour (benign) post right hemicolectomy
Chronic	History of Covid-19 (August 2023)
Chronic	History of red eye (September 2023)

3. Potential Life-Threatening Complications

- Severe aortic regurgitation could lead to congestive heart failure.
- Infective endocarditis could cause septic emboli, acute valvular dysfunction, or heart block.
- Cytopenia could result in severe bleeding or infection due to insufficient blood cell counts.

4. Clinical Findings

Organ System	Anticipated Findings

Cardiovascular	Diastolic murmur, collapsing pulse, signs of congestive heart failure
Dermatological	Skin rash (resolved at present)
Hematological	Evidence of cytopenia such as pallor or petechiae
Infectious	Fever, chills, rigor (indicative of an ongoing infection)

5. Working Diagnoses

- Infective endocarditis
- Acute coronary syndrome (resolved)
- Drug-induced fever and cytopenia (potentially due to antibiotics)
- Severe aortic regurgitation (potentially secondary to endocarditis)

6. Relevant Investigations

Investigation	Justification
Repeat blood cultures	To identify causative organism of suspected endocarditis despite antibiotic therapy
Bartonella serology	To rule out Bartonella as a cause for endocarditis; pending result noted
Echocardiogram follow-up	To assess the progression of aortic regurgitation and valve integrity
Full blood count	To monitor and assess the extent of cytopenia
Drug level monitoring (e.g., vancomycin, daptomycin)	To ensure therapeutic levels and avoid toxicity

7. Management Plan

- Continue IV ceftriaxone as per infectious disease recommendation.
- Monitor hemodynamics and consider cardiology consult for management of severe aortic regurgitation.
- If fever persists, revise antibiotic therapy based on culture results and sensitivity patterns.
- Regular full blood counts to monitor cytopenia, manage with supportive care or modification of drug therapy if druginduced.
- Consideration of surgical intervention if infective endocarditis and severe aortic regurgitation are not medically manageable.
- Symptomatic treatment for fever, including antipyretics and cooling measures.
- Strict monitoring for signs of heart failure or other complications.

8. Executive Summary

A 64-year-old male with a complex medical history including a benign caecal tumour and recent Covid-19 infection is suspected of having infective endocarditis following an acute coronary syndrome event. He has severe aortic regurgitation and has been treated with multiple antibiotics causing possible drug-induced fever and cytopenia.

The management plan includes continuing IV ceftriaxone, regular monitoring of full blood counts, echocardiogram follow-up, and potential surgical intervention for endocarditis. It is crucial to adjust treatment based on culture results and consultation with cardiology and infectious disease specialists.

cGPT-4

1. Chronological Events:

Date	Event
August 2023	Patient had Covid-19.
September 2023	Patient had a history of red eye.
11 October 2023	Admitted with chest pain and elevated troponin I, managed as acute coronary syndrome.
13 October 2023	Presented with swinging fever.
14 October 2023	Blood culture taken with no growth. First dose of augmentin given.
18 October 2023	Coronary angiogram showed mild coronary artery stenosis.
24 October 2023	TEE revealed thickened non-coronary cusp with 2mm perforation and severe AR, suspected endocarditis.
24-25 October 2023	Three sets of blood cultures taken post initiation of vancomycin.
5-9 November 2023	Fever with chills and rigor; piperacillin-tazobactam added to treatment.
9 November 2023	New onset cytopenia and skin rash; vancomycin switched to daptomycin.
15 November 2023	CT abdomen and pelvis showed no significant pathology.
17 November 2023	Discharged against medical advice from Hospital N and admitted to Hospital Q for further management.
13 November 2023	Another episode of fever.
18 November 2023	IV ceftriaxone started and continued till present.

2. Clinical Problem List:

Status	Clinical Issues
Active	Suspected infective endocarditis
Active	Fever with chills and rigor
Active	New onset cytopenia
Active	Skin rash
Chronic	History of caecal tumour (benign lesion)
Chronic	History of Covid-19 (recovered)
Chronic	History of red eye
Chronic	Mild coronary artery stenosis

Chronic	Severe aortic regurgitation

3. Potential Life-Threatening Complications:

The immediate life-threatening complications associated with the outlined clinical problems include:

- Heart failure due to severe aortic regurgitation.
- Systemic embolism from vegetations in infective endocarditis.
- Sepsis and septic shock due to ongoing infections.

4. Clinical Findings:

Organ System	Anticipated Finding
Cardiovascular	Diastolic murmur, collapsing pulse
Integumentary	Resolved skin rash
Hematological	Cytopenia
General	Fever, chills, rigor

5. Working Diagnoses:

- Suspected infective endocarditis.
- Fever of unknown origin (rule out nosocomial infection, drug fever).

6. Relevant Investigations:

Investigation	Justification
Repeat blood cultures	To identify causative organism, especially pre-antibiotic therapy.
Bartonella serology	As part of differential diagnosis for culture-negative endocarditis.
Echocardiogram	To assess cardiac structure and function, monitor AR severity.
Full blood count	To monitor cytopenia, assess for infection or drug reaction.
Drug levels (e.g., daptomycin)	To ensure therapeutic levels and avoid toxicity.

7. Management Plan:

Considering the guidelines and the need for rigorous clinical oversight, the management plan should include:

- Continuation of IV ceftriaxone with consideration for other antibiotics based on culture results and sensitivities.
- Close monitoring for signs of heart failure or embolic events.
- Daily reviews by a nurse or similar health professional, with the possibility of oral switch when appropriate.
- Weekly multidisciplinary team meetings to review the patient's progress and adjust treatment plans as necessary.

- Arrange for a follow-up echocardiogram to monitor the progression of aortic regurgitation and the status of the vegetations.

8. Executive Summary:
The patient, a 64-year-old male with a complex medical history, is suspected of having infective endocarditis with severe aortic regurgitation. He has experienced multiple episodes of fever and has been on various antibiotics. The management plan involves rigorous clinical monitoring, appropriate antibiotic therapy guided by culture results, and a multidisciplinary approach to address the potential complications. Repeat investigations, especially blood cultures and echocardiograms, are crucial in guiding treatment and monitoring the patient's progress.

Gemini Pro

1. Chronological Events:

Date	Clinical Issue
August 2023	History of COVID-19
September 2023	History of red eye
October 11, 2023	Admitted to Hospital N for chest pain and elevated troponin I
October 13, 2023	Swinging fever
October 14, 2023	Blood culture drawn, first dose of Augmentin administered
October 18, 2023	Coronary angiogram reveals mild coronary artery stenosis
October 22-23, 2023	Antibiotics-free period
October 24, 2023	Transesophageal echocardiogram shows thickened non-coronary cusp with perforation and severe aortic regurgitation
October 24-25, 2023	Three sets of blood cultures performed
October 26, 2023	IV vancomycin initiated
November 5-9, 2023	Fever, chills, and rigor
November 9, 2023	Piperacillin-tazobactam added to vancomycin
November 9, 2023	Vancomycin switched to daptomycin due to cytopenia and skin rash
November 13, 2023	New episode of fever
November 15, 2023	Contrast-enhanced CT abdomen and pelvis shows no significant pathology
November 17, 2023	Discharged against medical advice from Hospital N
November 17, 2023	Admitted to Hospital Q for further management
November 18, 2023	IV ceftriaxone initiated

2. Clinical Problem List:

Active Clinical Problems	Chronic Clinical Problems
Fever	History of caecal tumor (right hemicolectomy in 2019)

Chills and rigor	Ex-smoker
Cytopenia	Social drinker
Skin rash	
Severe aortic regurgitation	
Suspected infective endocarditis	

3. Potential Life-Threatening Complications:
- Septic shock
- Disseminated intravascular coagulation (DIC)
- Multi-organ failure
- Endocarditis-related stroke
- Aortic dissection

4. Clinical Findings:

Organ System	Anticipated Physical Examination Findings
General	Not septic looking
Skin	No skin rash
Cardiovascular	Diastolic murmur, collapsing pulse
Respiratory	No findings mentioned
Gastrointestinal	No findings mentioned
Neurological	No findings mentioned

5. Working Diagnoses:
- Infective endocarditis
- Aortic regurgitation
- Cytopenia
- Skin rash

6. Relevant Investigations:

Investigation	Justification
Blood cultures	To identify the causative organism of infective endocarditis
Transthoracic echocardiogram (TTE)	To assess the severity of aortic regurgitation and evaluate for vegetation
Transesophageal echocardiogram (TEE)	To provide a detailed evaluation of the aortic valve and vegetation
Complete blood count (CBC)	To monitor cytopenia
Skin biopsy	To determine the cause of the skin rash
Bartonella serology	To rule out Bartonella infection
Brucella serology	To rule out Brucella infection
Mycoplasma serology	To rule out Mycoplasma infection
Coxiella serology (anti-phase I/II)	To rule out Coxiella infection

7. Management Plan:
- Continue IV ceftriaxone for 4 weeks to treat suspected infective endocarditis.
- Monitor blood cultures for growth and adjust antibiotics accordingly.
- Perform TTE and TEE to assess the severity of aortic regurgitation and evaluate for vegetation.
- Monitor CBC to monitor cytopenia and consider transfusion if necessary.
- Perform skin biopsy to determine the cause of the skin rash and initiate appropriate treatment.
- Provide supportive care, including fluid resuscitation, vasopressors, and mechanical ventilation if necessary.

8. Executive Summary:
A 64-year-old male with a history of caecal tumor, ex-smoker, and social drinker presented with fever, chills, rigor, cytopenia, and skin rash. He was diagnosed with infective endocarditis, aortic regurgitation, cytopenia, and skin rash. Investigations revealed severe aortic regurgitation and vegetation on TEE. The patient was started on IV ceftriaxone for 4 weeks and supportive care. Monitoring of blood cultures, CBC, and skin biopsy is ongoing.

Claude 2

1. Chronological Events

Date	Event
August 2023	History of COVID-19 infection
September 2023	History of red eye
11 October 2023	Admitted for chest pain, elevated troponin I. Diagnosed with acute coronary syndrome.
13 October 2023	1 day of swinging fever, subsequently subsided. Blood culture done.
14 October 2023	First dose of Augmentin given.
18 October 2023	Coronary angiogram showed mild coronary artery stenosis.
24 October 2023	Transesophageal echocardiogram showed thickened non-coronary cusp with perforation and severe AR.
24-25 October 2023	3 sets of blood cultures done. IV vancomycin started.
5-9 November 2023	Fever, chills and rigor. Piperacillin-tazobactam added.
9 November 2023	Vancomycin switched to daptomycin due to cytopenia and rash.
15 November 2023	CT abdomen and pelvis showed no significant findings.
17 November 2023	Discharged from Hospital N, admitted to Hospital Q.
13 November 2023	Persistent fever.

2. Clinical Problem List

Problem	Active/Chronic
Coronary artery disease	Chronic
Infective endocarditis	Active
Fever	Active
Cytopenia	Active
Skin rash	Active

3. Potential Life-Threatening Complications
- Septic emboli to brain or other organs
- Heart failure from severe valvular regurgitation
- Immune complex-mediated glomerulonephritis

4. Anticipated Physical Examination Findings

System	Expected Findings
Cardiovascular	Diastolic murmur, collapsing pulse
Dermatologic	No current rash
Other systems	Likely unremarkable

5. Working Diagnoses
- Infective endocarditis
- Drug fever from antimicrobials
- Drug-induced cytopenia
- Drug rash

6. Relevant Investigations

Investigation	Justification
Blood cultures	To identify causative organism
Serology for Bartonella, Coxiella, Brucella	Rule out atypical organisms
Autoimmune workup	Evaluate for immune complex disease
Skin biopsy	If rash recurs, to evaluate possible vasculitis

7. Management Plan
- Continue IV antibiotics to complete recommended duration
- Monitor blood counts and renal function for adverse effects
- Repeat blood cultures 48-72 hours after antibiotics changed
- Repeat TEE in 2-3 weeks to evaluate valve vegetation
- Evaluate need for valve surgery if vegetation grows or persists
- Address modifiable risk factors for infective endocarditis
- Provide IE prevention education prior to discharge
- Follow up closely after discharge to monitor for complications

8. Executive Summary
64M with history of infective endocarditis on recent TEE, presenting with persistent fever on IV antibiotics. Main priorities are confirming diagnosis, identifying organism, monitoring for complications like septic emboli, managing adverse effects, determining if valve surgery is indicated, and planning post-discharge care.

11. Clinical scenario 11

GPT-4.0

1. Chronological Events

Date	Event
2021	Chemotherapy with Pembrolizumab for Stage IV squamous cell carcinoma lung, 35 cycles.
May 2023	Last PET-CT showing metabolic complete remission.
21 Nov 2023	Admission for high fever associated with left lower limb cellulitis, improved with antibiotics.
21 Nov 2023	Persistent high fever, admitted through accident and emergency department.
21 Nov 2023	Noted diplopia (left sixth cranial nerve palsy) and photophobia.
22 Nov 2023	Lumbar puncture performed.
21 Nov 2023	Initiation of Piperacillin-tazobactam.
22 Nov 2023	Start of Meropenem, Ceftriaxone, and Acyclovir.

2. Clinical Problem List

Status	Clinical Issues
Active	High fever with history of left lower limb cellulitis
Active	Diplopia with left sixth cranial nerve palsy
Active	Photophobia
Chronic	Diabetes Mellitus
Chronic	Hyperlipidaemia
Chronic	Depression
Chronic	Stage IV squamous cell carcinoma of the lung (currently in remission)

3. Potential Life-Threatening Complications
- Sepsis secondary to uncontrolled infection
- Meningoencephalitis or central nervous system infection due to cranial nerve palsy and altered mental status
- Diabetic ketoacidosis or hyperosmolar hyperglycemic state due to diabetes
- Tumor lysis syndrome from chemotherapy, though less likely given the current remission status

4. Clinical Findings

Organ System	Anticipated Findings
Neurological	Altered mental status, diplopia, photophobia
Musculoskeletal	Normal muscle strength (power 5/5 in all limbs)
Integumentary	No rash or signs of cellulitis on examination
Ophthalmological	Inability to assess extraocular movements fully

5. Working Diagnoses

- Left sixth cranial nerve palsy
- Central nervous system infection
- Post-infectious/inflammatory syndrome
- Recurrent cellulitis or another infection not yet identified

6. Relevant Investigations

Investigation	Justification
Repeat Blood Cultures	To rule out ongoing sepsis/bacteremia
MRI Brain with and without contrast	To evaluate for intracranial pathology causing cranial nerve palsy
HbA1c	To assess glycemic control in the context of diabetes
Repeat Lumbar Puncture	To obtain more cerebrospinal fluid for analysis if clinical suspicion persists

7. Management Plan

- Continue broad-spectrum antibiotics until a definitive source of infection is identified or ruled out.
- Optimize glycemic control to prevent complications related to diabetes.
- Close neurological observation and repeat imaging if neurological status worsens.
- Consider infectious disease and neurology consults for multidisciplinary approach to diagnosis and management.
- Monitor for signs of sepsis or other life-threatening complications, and manage supportively as needed.

8. Executive Summary

A 76-year-old female with a history of diabetes, hyperlipidaemia, depression, and Stage IV squamous cell carcinoma of the lung (in remission) is currently admitted due to persistent high fever and neurological symptoms, including diplopia. She is on broad-spectrum antibiotics with previous improvement of cellulitis. Workup including lumbar puncture has not identified a definitive infectious source, although meningitis/encephalitis has been ruled out by PCR. Management should include continued antibiotic therapy, glycemic control, and further diagnostic workup for her

neurological symptoms, with consideration for potential life-threatening complications such as sepsis or central nervous system infection.

cGPT-4

1. Chronological Events:

Date	Event
2021	Begin Pembrolizumab for Stage IV squamous cell ca lung
May 2023	Metabolic complete remission confirmed by PET-CT
21 November 2023	Admission for high fever, previous left lower limb cellulitis
22 November 2023	Noted diplopia (left sixth cranial nerve palsy), photophobia, Lumbar puncture performed

2. Clinical Problem List:

Status	Clinical Issues
Active	High fever, Diplopia, Photophobia, Left sixth cranial nerve palsy
Chronic	Diabetes mellitus, Hyperlipidaemia, Depression, Stage IV squamous cell ca lung (in remission)

3. Potential Life-Threatening Complications:
 - Septicemia or systemic infection
 - Potential central nervous system infection or malignancy causing cranial nerve palsy

4. Clinical Findings:

Organ System	Anticipated Findings
Neurological	Altered mental status, Diplopia, Photophobia, Possible partial cranial nerve palsy
Musculoskeletal	Power 5/5 over all four limbs
Integumentary	Absence of rash

5. Working Diagnoses:
 - Fever of unknown origin (possible infectious, neoplastic, or drug-related etiology)
 - Left sixth cranial nerve palsy (etiology to be determined)

6. Relevant Investigations:

Investigation	Justification
Repeat blood cultures	No growth so far, persistent fever could indicate ongoing infection

CT or MRI of the brain	Investigate cranial nerve palsy, rule out central nervous system
Review of current and recent medications	Assess for drug-related etiologies of fever and neurological symptoms
Serum inflammatory markers (e.g., ESR, CRP)	Evaluate for systemic inflammation or infection

7. Management Plan:
- Continue broad-spectrum antimicrobials as per previous regimen: Piperacillin-tazobactam, Meropenem, Ceftriaxone, and Acyclovir until further evidence is obtained.
- Daily clinical reviews to monitor response to therapy, with consideration for oral switch when appropriate.
- Weekly multidisciplinary team meetings to reassess the management plan.
- Engage in enhanced cleaning and monitoring for infection control, particularly given the patient's immunocompromised state.
- Regularly review antibiotic prescribing to avoid contributing to selection pressure and resistance.
- Implement a risk assessment and management strategy to safeguard the patient.

8. Executive Summary:
The patient is a 76-year-old female with a complex history including diabetes, hyperlipidaemia, depression, and Stage IV squamous cell lung cancer in remission, currently admitted for high fever and neurological symptoms. Management involves broad-spectrum antimicrobials with ongoing investigations to identify the source of fever and neurological symptoms. Risk management strategies are in place, with regular multidisciplinary reviews to refine the treatment plan.

Gemini Pro

1. Chronological Events:
Date	Event
2021	Started chemotherapy with Pembrolizumab for 35 cycles
May 2023	Last PET-CT showed metabolic complete remission
21 November 2023	Admitted for high fever associated with left lower limb cellulitis
22 November 2023	Lumbar puncture performed, CSF analysis showed elevated TCC, Lymphocyte and Monocyte, elevated CSF Protein, normal CSF Glucose
22 November 2023	Started on ceftriaxone and acyclovir

2. Clinical Problem List:
Active	Chronic
High fever	Diabetes mellitus

Diplopia (left sixth cranial nerve palsy)	Hyperlipidaemia
Photophobia	Depression
Left lower limb cellulitis	Stage IV squamous cell ca lung

3. Potential Life-Threatening Complications:
- Sepsis
- Meningitis
- Encephalitis

4. Clinical Findings:

Organ System	Anticipated Findings
Neurological	Fluctuating Glasgow coma scale, difficulty answering questions, diplopia, photophobia
Respiratory	No definite evidence of consolidation on CXR
Cardiovascular	No specific findings mentioned
Gastrointestinal	No specific findings mentioned
Musculoskeletal	Power 5 out of 5 over all four limbs
Integumentary	No rash seen over body

5. Working Diagnoses:
- Meningitis
- Encephalitis

6. Relevant Investigations:

Investigation	Justification
Repeat lumbar puncture	To obtain sufficient CSF for further analysis, including culture, Gram stain, and additional molecular testing
Blood culture	To rule out systemic infection
MRI brain with contrast	To assess for structural abnormalities and inflammation in the brain
Serology for syphilis, HIV, and Lyme disease	To rule out other potential causes of meningitis and encephalitis
Additional CSF testing	To rule out other potential causes of meningitis and encephalitis, such as cryptococcal antigen, herpes simplex virus PCR, varicella-zoster virus PCR, enterovirus PCR

7. Management Plan:
- Continue broad-spectrum antibiotics (ceftriaxone and acyclovir) until culture results are available |
- Monitor for signs and symptoms of sepsis and meningitis |
- Perform repeat lumbar puncture to obtain sufficient CSF for further analysis |

- Administer empiric antifungal therapy if cryptococcal antigen is positive |
- Consider antiviral therapy if herpes simplex virus, varicella-zoster virus, or enterovirus is detected in the CSF |
- Provide supportive care, including fluids, electrolytes, and oxygen therapy |

8. Executive Summary:
- The patient is a 76-year-old female with a history of stage IV squamous cell ca lung and diabetes mellitus, presenting with high fever, diplopia, and photophobia.
- Lumbar puncture revealed elevated CSF TCC, Lymphocyte and Monocyte, elevated CSF Protein, and normal CSF Glucose.
- The working diagnoses are meningitis and encephalitis.
- Investigations are recommended to identify the specific cause of the infection.
- Treatment includes broad-spectrum antibiotics, empiric antifungal therapy if cryptococcal antigen is positive, and supportive care.

Claude 2

1. Chronological Events
Date	Event
2021	Diagnosed with stage IV squamous cell carcinoma of the lung, started on pembrolizumab
May 2023	PET-CT showed complete metabolic remission
November 2023	Admitted for fever and left lower limb cellulitis
22 November 2023	Developed diplopia and photophobia, lumbar puncture performed

2. Clinical Problem List
Problem	Active/Chronic
Stage IV lung cancer	Chronic
Diabetes mellitus	Chronic
Hyperlipidemia	Chronic
Depression	Chronic
Fever	Active
Left lower limb cellulitis	Active
Diplopia and photophobia	Active

3. Potential Life-Threatening Complications
- Severe infection leading to septic shock
- Meningitis or encephalitis leading to altered mental status
- Cancer progression or metastasis

4. Anticipated Physical Examination Findings

System	Expected Findings
Neurological	Cranial nerve palsy, photophobia, altered mental status
Cardiovascular	Tachycardia, hypotension (if septic shock)
Pulmonary	Crackles, rhonchi (if pneumonia)
Extremities	Cellulitis

5. Working Diagnoses
- Meningitis: supported by fever, meningeal signs, CSF leukocytosis
- Encephalitis: supported by altered mental status, fever, CSF leukocytosis
- Brain metastasis: supported by cancer history and neurological deficits
- Sepsis: supported by fever and elevated white blood cell count

6. Relevant Investigations

Investigation	Rationale
Blood cultures	To identify bacteremia source of sepsis
CSF bacterial culture	To isolate causative organism for meningitis
CSF viral PCR	To identify viral cause of meningitis/encephalitis
CSF cytology	To assess for malignant cells indicating brain metastasis
CT brain	To assess for structural abnormalities or metastases
MRI brain	Better visualization of brain parenchyma for metastases

7. Management Plan
- Address any immediate life-threatening issues such as airway/breathing compromise or septic shock with fluids/pressors
- Continue empiric antibiotics to cover bacterial meningitis (e.g. ceftriaxone) and HSV encephalitis (acyclovir)
- Monitor for signs of increased intracranial pressure and deteriorating mental status
- Obtain neurosurgery consult for possibility of dexamethasone and/or surgical intervention if brain metastasis suspected
- Monitor culture results to tailor antibiotics once organism identified
- Consult oncology regarding cancer treatment if malignancy progression suspected
- Address hyperglycemia in setting of infection
- Provide supportive care including hydration, electrolyte repletion, physical/occupational therapy

8. Executive Summary
This elderly female with stage IV lung cancer presented with fever and new neurological deficits concerning for meningitis, encephalitis or brain metastasis. Empiric antibiotics and antivirals were started. Further workup with CSF cultures,

neuroimaging and cancer re-staging is recommended to determine underlying etiology and guide definitive treatment.

12. Clinical scenario 12

GPT-4.0

1. Chronological Events

Date	Event
2007	Patient had syncope and was labeled allergic to penicillin; has not tried other beta-lactam antibiotics
Pre-2023	Diagnosed with poliomyelitis, ischemic heart disease, paraumbilical hernia, and chronic hepatitis B cirrhosis
March 2023	Received first Transarterial chemoembolization (TACE)
July 2023	Received second TACE
Sep 2023	Atypical cells found in right pleural effusion; multifocal HCC and collapsed right lower lobe on CT
12 Oct 2023	Ultrasound-guided ascitic drainage showed atypical cells
5 Nov 2023	Admitted for ascites and paraumbilical hernia
6 Nov 2023	CT showed umbilical hernia with collection; operation for mesh repair; peritoneal fluid culture no growth
24 Nov 2023	Transferred to Hospital Q for further management
25 Nov 2023	CT showed enlarged heterogenous hyperdense subcutaneous lesion at paraumbilical region
27 Nov 2023	Operation for incision and drainage of infected hematoma; cultures no growth
1 Dec 2023	Developed fever; Blood culture grew Staphylococcus aureus, susceptibility pending

2. Clinical Problem List

Status	Clinical Issues
Active	Syncope (2007), labeled allergic to penicillin
Active	Chronic hepatitis B cirrhosis
Chronic	Ischemic heart disease
Chronic	Paraumbilical hernia
Active	Multifocal hepatocellular carcinoma
Active	Ascites
Active	Right pleural effusion with atypical cells
Active	Infected hematoma post-operation
Active	Fever, Blood culture positive for Staphylococcus aureus
Chronic	Poliomyelitis with lower limb power zero

3. Potential Life-Threatening Complications
- Hepatocellular Carcinoma: Risk of liver failure, hemorrhage, or metastasis.
- Cirrhosis: Risk of variceal bleeding, hepatic encephalopathy, or spontaneous bacterial peritonitis.
- Infected Hematoma: Risk of sepsis or abscess formation.
- Staphylococcus aureus Bacteremia: Risk of septicemia, endocarditis, or metastatic infections.

4. Clinical Findings

Organ System	Physical Examination Findings
General	Low-grade fever, general condition well
Respiratory	Occasional crepitations
Cardiovascular	Heart sound dual, no murmur
Abdominal	Distended, soft, non-tender
Extremities	Finger clubbing, no DVT

5. Working Diagnoses
- Multifocal Hepatocellular Carcinoma
- Infected Hematoma Post-Hernia Repair
- Staphylococcus aureus Bacteremia
- Ascites secondary to cirrhosis
- Right lower lobe atelectasis

6. Relevant Investigations

Investigation	Justification
Blood culture and sensitivity	To determine antibiotic susceptibility for Staphylococcus aureus
Repeat CT or ultrasound of abdomen	To evaluate the status of ascites, and the liver lesions
Echocardiogram	To rule out infective endocarditis due to positive blood culture
Hepatic function panel	To monitor the status of chronic hepatitis B cirrhosis and HCC
Ascitic fluid analysis	To diagnose spontaneous bacterial peritonitis

7. Management Plan
- Infection Control: Continue with Levofloxacin and Metronidazole until culture sensitivities are available, then adjust antibiotics accordingly.
- Cancer Management: Continue with current oncological treatments, monitor tumor progression, and adjust treatments as necessary.
- Ascites Management: Diuretics for ascites, salt restriction, and periodic paracentesis if needed.

- Nutritional Support: Ensure adequate nutrition, considering the patient's liver disease.
- Pain Management: Appropriate analgesia while avoiding drugs with hepatic metabolism.
- Physiotherapy: For the maintenance of respiratory function and prevention of complications due to immobility.

8. Executive Summary

The patient, a 55-year-old male with a history of penicillin allergy, poliomyelitis, ischemic heart disease, and chronic hepatitis B cirrhosis, presented with complications including multifocal hepatocellular carcinoma treated with TACE and DEB-TACE, and a recent episode of syncope. The latest admission is for ascites management and complications post-hernia repair, with a Staphylococcus aureus bacteremia diagnosed. Immediate life-threatening complications to monitor include liver failure, hemorrhage, sepsis, and potential infective endocarditis. The management plan includes antibiotic therapy guided by culture sensitivities, continuous oncological treatment monitoring, ascites management, nutritional support, pain management, and physiotherapy. The patient's care requires a multidisciplinary approach due to the complexity of the medical history and current clinical issues.

cGPT-4

1. Chronological Events:

Date	Event
2007	Presented with syncope, labeled allergic to penicillin.
March 2023	Received first round of Transarterial chemoembolization (TACE).
July 2023	Received second round of TACE.
September 2023	Atypical cells found in right pleural effusion; CT showed multifocal hepatocellular carcinoma with collapsed right lower lobe.
12 October 2023	USG-guided ascitic drainage showed atypical cells.
5 November 2023	Admitted for ascites with paraumbilical hernia.
6 November 2023	CT showed umbilical hernia, operation for mesh repair of hernia. Peritoneal fluid culture showed no growth.
24 November 2023	Transferred to Hospital Q for further management.
25 November 2023	CT showed heterogenous hyperdense subcutaneous lesion with interval enlargement at paraumbilical region. Started on Levofloxacin.
27 November 2023	Operation for incision and drainage of infected hematoma; intra-operative culture no growth.
1 December 2023	Developed fever; blood culture positive for Staphylococcus aureus. Started on Metronidazole.

2. Clinical Problem List:

| Status | Clinical Issue |

Active	Allergy to penicillin
Active	Ischemic heart disease
Active	Chronic hepatitis B cirrhosis
Active	Multifocal hepatocellular carcinoma
Active	Paraumbilical hernia repair with post-operative complications
Active	Infected hematoma
Active	Fever with Staphylococcus aureus bacteremia
Chronic	Poliomyelitis with lower limb power zero
Chronic	Right pleural effusion with atypical cells
Chronic	Ascites

3. Potential Life-Threatening Complications:
- Hepatic encephalopathy due to chronic hepatitis B cirrhosis.
- Further spread or worsening of multifocal hepatocellular carcinoma.
- Septicemia caused by Staphylococcus aureus with the potential for metastatic infection or septic shock.
- Complications due to ischemic heart disease exacerbated by infection or stress on the body.

4. Clinical Findings:

Organ System	Anticipated Findings
Cardiovascular	Stable vitals, dual heart sounds with no murmurs
Respiratory	Occasional crepitations, right lower lobe atelectasis
Abdominal	Distension, soft, non-tender on palpation
Musculoskeletal	Finger clubbing, power zero out of five in lower limbs

5. Working Diagnoses:
- Postoperative wound infection or infected hematoma.
- Staphylococcus aureus bacteremia.
- Decompensated chronic hepatitis B cirrhosis with ascites.
- Ongoing management of multifocal hepatocellular carcinoma.

6. Relevant Investigations:

Investigation	Justification
Repeat blood cultures	To monitor the effectiveness of antibiotic therapy and to rule out ongoing bacteremia.
Liver function tests (LFTs)	To assess the status of liver function and progression of cirrhosis.
Alpha-fetoprotein (AFP)	As a tumor marker for hepatocellular carcinoma.

| Echocardiogram | To evaluate heart function given the history of ischemic heart disease. |
| Ultrasound of abdomen | To assess ascites and evaluate for potential complications such as spontaneous bacterial peritonitis. |

7. Management Plan:
- Continue Levofloxacin and Metronidazole as per susceptibility testing results.
- Evaluate the necessity of alternative antibiotics post susceptibility testing, considering penicillin allergy and potential cross-reactivity with other beta-lactam antibiotics.
- Close monitoring for signs of hepatic encephalopathy, including mental status assessments and ammonia levels.
- Monitor for signs of ischemic heart disease exacerbation and manage accordingly.
- Arrange for a multidisciplinary team meeting to discuss the ongoing management of hepatocellular carcinoma and possible palliative care options.

8. Executive Summary:
The patient, a 55-year-old male with a complex medical history including chronic hepatitis B cirrhosis with multifocal hepatocellular carcinoma and a recent operation for paraumbilical hernia, presents with postoperative complications and Staphylococcus aureus bacteremia. Management includes ongoing antibiotic therapy with close monitoring and multidisciplinary discussions for the management of his cancer. Given the patient's allergy to penicillin, careful consideration of antibiotic selection is necessary. Further investigations are required to monitor the status of his underlying chronic conditions and response to therapy.

Gemini Pro

1. Chronological Events:

Date	Clinical Issue
2007	Syncope
March 2023	Transarterial chemoembolization (TACE)
July 2023	Transarterial chemoembolization (TACE)
September 2023	Atypical cells in right pleural effusion
12 October 2023	USG-guided ascitic drainage
5 November 2023	Admitted for ascites with paraumbilical hernia
6 November 2023	CT scan shows umbilical hernia with a 1.6 x 1.2cm lobulated rim enhancing collection
6 November 2023	Mesh repair of hernia
24 November 2023	Transferred to Hospital Q for further management
25 November 2023	CT scan shows a 8.6x3x6.8cm heterogenous hyperdense subcutaneous lesion at paraumbilical region

27 November 2023	Incision and drainage with intra-operatively noted infected haematoma
1 December 2023	Fever, blood culture positive for Staphylococcus aureus
1 December 2023	Levofloxacin and Metronidazole started

2. Clinical Problem List:

Active	Chronic
Abdominal distension	Poliomyelitis infection
Occasional pain over ascitic fluid drain site	Ischemic heart disease
Cough	Paraumbilical hernia
Fever	Chronic hepatitis B cirrhosis
Staphylococcus aureus bacteremia	Multifocal hepatocellular carcinoma
Infected haematoma	
Right lower lobe atelectasis	

3. Potential Life-Threatening Complications:
* Sepsis
* Respiratory failure
* Liver failure
* Endocarditis
* Deep vein thrombosis
* Pulmonary embolism

4. Clinical Findings:

Organ System	Anticipated Physical Examination Findings
Respiratory	Occasional crepitations in the chest
Cardiovascular	Dual heart sounds, no murmurs
Integumentary	Finger clubbing, no other signs of infective endocarditis
Abdominal	Distended, soft, non-tender on palpation

5. Working Diagnoses:
* Staphylococcus aureus bacteremia
* Infected haematoma
* Right lower lobe atelectasis
* Chronic hepatitis B cirrhosis
* Multifocal hepatocellular carcinoma
* Paraumbilical hernia

6. Relevant Investigations:

Investigation	Justification

Blood cultures	To identify the causative organism and determine antimicrobial susceptibility
Chest X-ray	To assess the extent of atelectasis and rule out other pulmonary complications
Abdominal ultrasound	To evaluate the extent of the infected haematoma and assess for any intra-abdominal complications
Liver function tests	To monitor liver function and assess for any hepatic decompensation
Renal function tests	To assess renal function and adjust antimicrobial dosing if necessary
Coagulation studies	To assess for any coagulopathy and guide management

7. Management Plan:
* Antimicrobial therapy: Vancomycin and Ceftriaxone
* Surgical consultation for management of the infected haematoma
* Respiratory physiotherapy and bronchodilators for the right lower lobe atelectasis
* Continue monitoring of liver function and renal function
* Supportive care including fluids, electrolytes, and nutritional support

8. Executive Summary:
The patient is a 55-year-old male with a history of poliomyelitis, ischemic heart disease, paraumbilical hernia, chronic hepatitis B cirrhosis, and multifocal hepatocellular carcinoma. He presented with abdominal distension, occasional pain over the ascitic fluid drain site, cough, and fever. Blood cultures were positive for Staphylococcus aureus. He was diagnosed with Staphylococcus aureus bacteremia, infected haematoma, right lower lobe atelectasis, chronic hepatitis B cirrhosis, and multifocal hepatocellular carcinoma. Treatment includes antimicrobial therapy, surgical consultation for management of the infected haematoma, respiratory physiotherapy and bronchodilators for the right lower lobe atelectasis, and supportive care.

Claude 2

1. Chronological Events
Date	Event
2007	Presented with syncope after penicillin administration
Prior to admission	Diagnosed with poliomyelitis resulting in lower limb paralysis, ischemic heart disease, paraumbilical hernia, chronic hepatitis B with cirrhosis and multifocal HCC
March 2023	Received TACE for HCC
July 2023	Received second TACE for HCC
September 2023	Incidental finding of atypical cells in right pleural effusion, CT showed multifocal HCC with collapsed right lower lobe

12 October 2023	Ascitic drainage showed atypical cells
5 November 2023	Admitted for ascites and paraumbilical hernia
6 November 2023	CT showed umbilical hernia with rim enhancing collection, no growth on fluid culture
24 November 2023	Transferred to Hospital Q for further management
25 November 2023	CT showed enlarged subcutaneous lesion at paraumbilical region
27 November 2023	Incision and drainage of infected hematoma, cultures no growth
1 December 2023	Developed fever, blood culture grew Staphylococcus aureus

2. Clinical Problem List

Active Problems	Chronic Problems
Infected paraumbilical hematoma	Poliomyelitis with lower limb paralysis
Fever	Ischemic heart disease
Ascites	Paraumbilical hernia
Right pleural effusion	Chronic hepatitis B with cirrhosis
Multifocal hepatocellular carcinoma	Multifocal hepatocellular carcinoma
Staphylococcus aureus bacteremia	Penicillin allergy

3. Potential Life-Threatening Complications
- Sepsis from Staphylococcus aureus bacteremia
- Hepatorenal syndrome from progressive liver failure
- Spontaneous bacterial peritonitis
- Hepatic encephalopathy
- Variceal bleeding
- Disease progression of hepatocellular carcinoma

4. Anticipated Physical Examination Findings

System	Findings
General	Low grade fever, well appearing
HEENT	No evidence of icterus
Cardiac	Dual heart sounds, no murmurs
Pulmonary	Occasional crackles
Abdominal	Distended, soft, non-tender, fluid wave, umbilical hernia
Extremities	Finger clubbing, lower limb paralysis

5. Working Diagnoses
- Infected paraumbilical hematoma
- Ascites secondary to cirrhosis
- Staphylococcus aureus bacteremia
- Right pleural effusion concerning for malignant effusion

- Decompensated cirrhosis
- Hepatocellular carcinoma

6. Investigations

Investigation	Rationale
Blood cultures	Diagnose bacteremia
Ascitic fluid analysis	Assess for spontaneous bacterial peritonitis
Pleural fluid analysis	Diagnose malignant effusion
Hepatic function panel	Assess liver function
INR	Assess coagulation status
Hepatic ultrasound	Evaluate liver parenchyma and screen for hepatocellular carcinoma
Alpha-fetoprotein	Tumor marker to monitor hepatocellular carcinoma

7. Management Plan
- Treat bacteremia with intravenous antibiotics per susceptibilities
- Manage ascites with diuretics and paracentesis as needed
- Consider transjugular intrahepatic portosystemic shunt (TIPS) for refractory ascites
- Continue HCC surveillance and consider locoregional therapies
- Monitor for complications of cirrhosis - screen for varices, hepatic encephalopathy, hepatorenal syndrome
- Avoid hepatotoxic medications
- Consider liver transplantation evaluation

8. Executive Summary
This 55 year old man with cirrhosis, HCC, and paraplegia presents with an infected paraumbilical hematoma and Staphylococcus aureus bacteremia. Key issues are treating the infection, managing complications of cirrhosis including ascites and pleural effusion, HCC surveillance, and preventing sepsis and organ failure. Necessary workup includes cultures, liver and cancer screening.

13. Clinical scenario 13

GPT-4.0

1. Chronological Events:

Date	Clinical Event
24 Nov 2023	Admission for perforated peptic ulcer with gross peritonitis
24 Nov 2023	Presentation with epigastric pain radiating to the back
24 Nov 2023	Development of shock and anuric AKI
24 Nov 2023	CT scan showing gross pneumoperitoneum with free fluid
25 Nov 2023	Operation: patch repair of 2 cm anterior D1 ulcer

Post-antibiotics	Pus culture grew Candida glabrata
24 Nov - 1 Dec 2023	Administration of Piperacillin-tazobactam
24 Nov 2023 - Now	Administration of Amoxicillin-clavulanate and Micafungin

2. Clinical Problem List:

Status	Clinical Issue
Chronic	Schizophrenia
Chronic	Obesity
Chronic	Diabetes mellitus (HbA1c 5.0%)
Chronic	Hypertension
Active	Perforated peptic ulcer (PPU)
Active	Gross peritonitis
Active	Shock
Active	Anuric acute kidney injury (AKI)
Active	Gross pneumoperitoneum
Active	Candida glabrata infection

3. Potential Life-Threatening Complications:
- Perforated Peptic Ulcer: Risk of sepsis, ongoing peritonitis.
- Gross Peritonitis: Can lead to severe sepsis or septic shock.
- Shock: May result in multi-organ failure.
- Anuric AKI: Potential progression to chronic kidney disease or the need for renal replacement therapy.
- Candida glabrata Infection: Possibility of systemic fungal infection, which is harder to treat due to inherent antifungal resistance.

4. Clinical Findings:

Organ System	Anticipated Findings
Cardiovascular	Hypotension, tachycardia (due to shock)
Respiratory	Tachypnea (compensatory response to shock)
Abdominal	Tenderness, rigidity (signs of peritonitis)
Renal	Oliguria or anuria (due to AKI)
Neurological	Altered mental status (if in shock)
Integumentary	Possible jaundice (if liver function affected)
Endocrine	Signs of diabetes management (glucose monitor)

5. Working Diagnoses:
- Perforated peptic ulcer with gross peritonitis.
- Anuric acute kidney injury secondary to shock.
- Systemic Candida glabrata infection.

6. Relevant Investigations:

Investigation	Justification
Repeat WBC and ANC	To monitor for infection/sepsis
Serum electrolytes	To evaluate electrolyte imbalances secondary to AKI
Repeat sCr and eGFR	To monitor kidney function
Blood cultures	To identify any ongoing or new bacterial infections
Repeat liver function tests	To monitor liver function in the context of systemic infection
Repeat CT abdomen	To assess for any ongoing abdominal pathology
Echocardiogram	To evaluate cardiac function post-shock

7. Management Plan:
- Continue broad-spectrum antifungals due to the presence of Candida glabrata.
- Monitor and support vital signs to address shock, including fluid resuscitation and vasopressors if necessary.
- Monitor renal function closely, consider renal replacement therapy if no improvement in anuria.
- Nutritional support considering obesity and diabetes status.
- Tight glycemic control in the context of diabetes and acute illness.
- Proton pump inhibitor (PPI) therapy for peptic ulcer disease.
- Regular psychiatric evaluation to manage schizophrenia and monitor for medication interactions.
- Monitor and manage electrolyte imbalances.

8. Executive Summary:
The patient, a 48-year-old male with a complex medical history including schizophrenia, obesity, well-controlled diabetes, and hypertension, was admitted with a perforated peptic ulcer leading to gross peritonitis and subsequent shock with anuric AKI. A CT scan confirmed gross pneumoperitoneum. Surgery was performed, and postoperative cultures grew Candida glabrata. The management plan includes continuation of antifungal therapy, hemodynamic support for shock, closerenal monitoring, glycemic control, and psychiatric support. Potential life-threatening complications include sepsis, septic shock, multi-organ failure, and systemic fungal infection due to inherent antifungal resistance. Immediate actions involve stabilizing the patient's hemodynamics, preventing further renal damage, and managing the systemic fungal infection effectively.

cGPT-4

1. Chronological Events:

Date	Event

24 November 2023	Admission for perforated peptic ulcer with gross peritonitis
24 November 2023	Development of shock with anuric AKI
24 November 2023	CT scan showing gross pneumoperitoneum with free fluid
25 November 2023	Operation revealing 2 cm anterior D1 ulcer with patch repair
Post-Operative	Pus culture growing Candida glabrata; Blood culture no growth

2. Clinical Problem List:

Status	Clinical Issues
Active	Perforated peptic ulcer
Active	Gross peritonitis
Active	Shock
Active	Anuric acute kidney injury
Chronic	Schizophrenia
Chronic	Obesity
Chronic	Diabetes mellitus
Chronic	Hypertension

3. Potential Life-Threatening Complications:
- Septic shock due to gross peritonitis and PPU.
- Multi-organ failure secondary to shock and AKI.
- Fungal sepsis due to Candida glabrata infection.

4. Clinical Findings:

Organ System	Anticipated Findings
Cardiovascular	Tachycardia, hypotension
Respiratory	Tachypnea, labored breathing
Abdominal	Guarding, rebound tenderness
Renal	Oliguria or anuria

5. Working Diagnoses:
- Perforated peptic ulcer with resultant gross peritonitis.
- Septic shock.
- Acute kidney injury (anuric).
- Candida glabrata superinfection.

6. Relevant Investigations:

Investigation	Justification
Repeat WBC count	Assess for response to treatment and current infection status
Repeat sCr and eGFR	Monitor renal function and progression of AKI
Follow-up blood cultures	Ensure no bacterial growth and monitor for fungemia

| Follow-up imaging (CT)| Evaluate the success of surgical intervention and any ongoing abdominal pathology |

7. Management Plan:
Given the severity of the patient's condition, the management should include:
- Continuation of broad-spectrum antimicrobials with consideration for antifungal coverage due to Candida glabrata growth. Consultation with an infectious disease specialist may be necessary for optimal antimicrobial therapy.
- Supportive care for shock, including fluid resuscitation and vasopressors as needed.
- Renal replacement therapy (RRT) for anuric AKI if indicated.
- Nutritional support considering the patient's obesity and diabetes.
- Psychiatric management for schizophrenia.
- Close monitoring and daily review by a nurse or similar healthcare professional, with weekly reviews in a multidisciplinary team meeting to monitor progress and adjust the treatment plan as necessary.

8. Executive Summary:
The patient, a 48-year-old male with a history of schizophrenia, obesity, diabetes, and hypertension, presented with a perforated peptic ulcer and gross peritonitis, leading to septic shock and anuric AKI. Surgical intervention was successful, but the patient developed a Candida glabrata superinfection. Ongoing management includes broad-spectrum antimicrobials, supportive care for shock, potential RRT, and multidisciplinary monitoring. Close attention to the patient's chronic conditions and psychiatric needs is crucial for comprehensive care.

Gemini Pro

1. Chronological Events:

Date	Event
24 November 2023	Admitted for perforated peptic ulcer (PPU) with gross peritonitis; shock with anuric acute kidney injury (AKI); CT scan showed gross pneumoperitoneum with free fluid.
25 November 2023	Operation revealed gross peritoneal contamination with turbid fluid and a 2 cm anterior first part of duodenum (D1) ulcer; patch repair performed.
24 November 2023	Pus culture (after antibiotics) grew Candida glabrata.
24 November 2023	Amoxicillin-clavulanate initiated.
24 November 2023	Piperacillin-tazobactam initiated.
26 November 2023	Micafungin initiated.
1 December 2023	Piperacillin-tazobactam discontinued.

2. Clinical Problem List:

Active	Chronic
Perforated peptic ulcer (PPU)	Schizophrenia
Gross peritonitis	Obesity
Anuric acute kidney injury (AKI)	Diabetes mellitus
Candida glabrata peritonitis	Hypertension

3. Potential Life-Threatening Complications:
* Sepsis
* Multi-organ failure
* Death

4. Clinical Findings:

Organ System	Anticipated Findings
Respiratory	Tachypnea, dyspnea, hypoxia
Cardiovascular	Tachycardia, hypotension, oliguria
Gastrointestinal	Abdominal pain, distension, tenderness, guarding, rebound
Renal	Oliguria, anuria
Neurological	Altered mental status, seizures

5. Working Diagnoses:
* Perforated peptic ulcer (PPU)
* Gross peritonitis
* Anuric acute kidney injury (AKI)
* Candida glabrata peritonitis

6. Relevant Investigations:

Investigation	Justification
Complete blood count (CBC)	To assess for anemia, leukocytosis, and thrombocytopenia.
Comprehensive metabolic panel (CMP)	To assess for electrolyte imbalances, renal function, and liver function.
Blood cultures	To identify the causative organism of the peritonitis.
Urine analysis	To assess for signs of infection or AKI.
Abdominal X-ray	To visualize the extent of the pneumoperitoneum.
Abdominal CT scan	To further evaluate the extent of the peritonitis and identify any complications.
Candida glabrata culture and sensitivity	To determine the susceptibility of the organism to antifungal agents.

7. Management Plan:

* Medical Management:
* Intravenous fluids and electrolytes
* Antibiotics (piperacillin-tazobactam and micafungin)
* Proton pump inhibitor (PPI)
* Hemodialysis for AKI
* Surgical Management:
* Exploratory laparotomy with repair of the perforated ulcer
* Supportive Care:
* Nutritional support
* Pain management
* Monitoring for complications

8. Executive Summary:
A 48-year-old male with a history of schizophrenia, obesity, diabetes mellitus, and hypertension presented with a perforated peptic ulcer complicated by gross peritonitis, anuric AKI, and Candida glabrata peritonitis. He underwent surgical repair of the ulcer and is currently receiving antibiotics and antifungal therapy. He requires hemodialysis for AKI. The patient is at high risk for sepsis and multi-organ failure. Close monitoring and aggressive management are necessary to prevent these complications.

Claude 2

1. Chronological Events

Date	Event
Prior to admission	Schizophrenia, obesity, diabetes mellitus, hypertension
24 November 2023	Admitted for perforated peptic ulcer with peritonitis and shock. Developed anuric AKI.
24 November 2023	CT showed pneumoperitoneum and free fluid
25 November 2023	Exploratory laparotomy showed D1 ulcer repair
After antibiotics	Pus culture grew Candida glabrata

2. Clinical Problem List

Problem	Active/Chronic
Perforated peptic ulcer	Active
Peritonitis	Active
Shock	Active
Anuric AKI	Active
Candida glabrata peritonitis	Active
Schizophrenia	Chronic
Obesity	Chronic
Diabetes mellitus	Chronic

Hypertension	Chronic

3. Potential Life-Threatening Complications
- Septic shock
- Multi-organ failure
- Disseminated candidiasis

4. Clinical Findings

System	Expected Findings
Cardiovascular	Tachycardia, hypotension
Respiratory	Tachypnea
Gastrointestinal	Abdominal tenderness, guarding, rigidity
Renal	Oliguria/anuria

5. Working Diagnoses
- Perforated peptic ulcer
- Peritonitis
- Septic shock
- Acute kidney injury
- Candida glabrata intra-abdominal infection

6. Relevant Investigations

Investigation	Rationale
Blood cultures	Identify bacteremia/fungemia
Antibiotic levels	Ensure therapeutic levels, adjust dosing
Inflammatory markers (CRP, procalcitonin)	Assess response to treatment
Kidney function tests (creatinine, eGFR)	Monitor kidney function
Fluid balance charts	Guide fluid therapy
Abdominal CT scan	Assess disease extent, complications

7. Management Plan
- ICU care with close hemodynamic monitoring
- Early goal-directed therapy for septic shock
- Broad-spectrum antibiotics (piperacillin/tazobactam, micafungin)
- Source control with adequate peritoneal washout and drainage
- Renal replacement therapy if severe AKI
- Strict glucose control
- Enteral nutrition if bowel function intact
- Physical and psychiatric rehabilitation

8. Executive Summary

This 48-year-old male with perforated peptic ulcer disease developed septic shock and multi-organ failure. He requires close ICU monitoring, antimicrobial therapy, source control, renal support, and multidisciplinary care to address his active clinical issues and prevent further complications.

14. Clinical scenario 14

GPT-4.0

1. Chronological Events:

Date	Event
June 2023	Tenckhoff catheter removed due to E. coli CAPD peritonitis
14 November 2023	Tenckhoff catheter reinserted
25 November 2023	Blood culture isolated Campylobacter fetus; started on amoxicillin-clavulanate and intraperitoneal cefazolin and amikacin
27 November 2023	Repeat blood culture showed no growth
29 November 2023	Azithromycin treatment initiated

2. Clinical Problem List:

Status	Clinical Issues
Active	Fever, vomiting, productive cough
Active	CAPD peritonitis (resolved, current treatment for prevention)
Chronic	Diabetes mellitus with nephropathy
Chronic	End-stage renal failure on CAPD
Chronic	Hypertension
Chronic	Right persistent clinically significant macular edema (CSME)

3. Potential Life-Threatening Complications:
- Sepsis from Campylobacter fetus bacteremia
- Peritonitis recurrence or complications from recent catheter reinsertion
- Diabetic complications including hyperglycemic hyperosmolar state (HHS)
- Complications of end-stage renal disease including hyperkalemia, fluid overload, and uremia

4. Clinical Findings:

Organ System	Anticipated Findings
General	Fever, possible signs of dehydration from vomiting
Respiratory	Productive cough, but clear chest on examination
Cardiovascular	Stable blood pressure and pulse rate, no murmur
Abdominal	Soft abdomen, no signs of peritonitis on examination
Neurological	GCS full, no neurological deficits

5. Working Diagnoses:
- Campylobacter fetus bacteremia
- CAPD-related infection (resolved but under treatment)
- Diabetic nephropathy with end-stage renal disease
- Hypertensive cardiovascular disease
- Right persistent CSME

6. Relevant Investigations:

Investigation	Justification
Repeat blood cultures	To ensure clearance of Campylobacter bacteremia
PDF cultures	To rule out recurrent or ongoing peritoneal infection
Complete blood count	To monitor for infection, ANC for immune status
Serum electrolytes	To manage electrolyte imbalances in renal failure
HbA1c	To assess long-term glycemic control
Ophthalmologic exam	To monitor CSME

7. Management Plan:
- Continue current antimicrobial therapy as per sensitivities (amoxicillin-clavulanate, azithromycin, intraperitoneal cefazolin, and amikacin) and reassess based on culture results and clinical response.
- Monitor renal function closely and adjust drug dosages as necessary.
- Careful monitoring of blood glucose levels and adjustment of diabetic medication.
- Regular monitoring of vital signs and fluid status to manage hypertension and prevent fluid overload.
- Ophthalmologic follow-up for CSME.
- Evaluate the need for any additional vaccinations or prophylactic measures to prevent future infections.
- Nutritional support considering the patient's catabolic state due to infection and underlying chronic illnesses.

8. Executive Summary:
A 72-year-old female with a complex medical history including diabetes with nephropathy and end-stage renal failure on CAPD presents with fever, vomiting, and a productive cough. She has a history of CAPD peritonitis and recent catheter reinsertion. Current management includes targeted antimicrobial therapy for a confirmed Campylobacter fetus bacteremia. Key management strategies involve ongoing infection control, close monitoring of renal function, and managing her chronic conditions. Future prevention and close follow-up are crucial given her risk for recurrent infections and complications from her chronic diseases.

cGPT-4

1. Chronological Events:

Date	Event
June 2023	Tenckhoff catheter removal due to E. coli CAPD peritonitis
14 Nov 2023	Tenckhoff catheter reinsertion
25 Nov 2023	Admission for fever, vomiting, productive cough
25 Nov 2023	Start of amoxicillin-clavulanate and intraperitoneal antimicrobials
25 Nov 2023	Blood culture positive for Campylobacter fetus
27 Nov 2023	Repeated blood culture with no growth
29 Nov 2023	Switch from amoxicillin-clavulanate to azithromycin

2. Clinical Problem List:

Status	Clinical Issue
Active	Fever, vomiting, productive cough
Active	Campylobacter fetus bacteremia
Chronic	Diabetes mellitus with nephropathy
Chronic	End-stage renal failure on CAPD
Chronic	Hypertension
Chronic	Right CSME

3. Potential Life-Threatening Complications:
- Septic shock from Campylobacter fetus bacteremia.
- Complications from end-stage renal failure including electrolyte imbalances and fluid overload.

4. Clinical Findings:

Organ System	Anticipated Findings
General	Fever reduction post-antimicrobial therapy
Respiratory	Productive cough, clear chest on examination
Cardiovascular	Stable blood pressure and pulse, no murmurs
Abdominal	Soft abdomen, heparin block site not inflamed
Ophthalmologic	Evidence of CSME

5. Working Diagnoses:
- Campylobacter fetus bacteremia.
- Right CSME secondary to diabetes mellitus.
- Complications of end-stage renal disease and CAPD.

6. Relevant Investigations:

Investigation	Justification

Repeat blood cultures	To confirm clearance of Campylobacter fetus bacteremia
Serum electrolytes	To monitor for electrolyte imbalances due to renal failure
Ophthalmologic exam	To assess the status of CSME

7. Management Plan:
Continue monitoring the patient's response to azithromycin for Campylobacter fetus bacteremia, keeping in mind potential complications such as antibiotic resistance and drug reactions. Ensure optimal management of diabetes and hypertension to prevent further progression of CSME and renal complications. Monitor and adjust peritoneal dialysis regimen to manage end-stage renal disease. Regular follow-up with a multidisciplinary team, including a specialist nurse and a nephrologist, should be implemented.

8. Executive Summary:
The patient, a 72-year-old female with multiple chronic conditions, was admitted with symptoms indicative of an infection and was found to have Campylobacter fetus bacteremia. She is currently responding to azithromycin. Chronic conditions including end-stage renal disease, diabetes with nephropathy, hypertension, and CSME require ongoing management. A multidisciplinary approach, including regular monitoring and specialist consultations, is recommended to manage her complex health issues.

Gemini Pro

1. Chronological Events:

Date	Event
June 2023	Tenckhoff catheter removed due to Escherichia coli CAPD peritonitis
14 November 2023	Tenckhoff catheter reinserted
25 November 2023	Admitted for fever, vomiting, and productive cough
25 November 2023	Blood culture positive for Campylobacter fetus
25 November 2023	Started on amoxicillin-clavulanate and intraperitoneal cefazolin and amikacin
27 November 2023	Repeated blood culture no growth
29 November 2023	Switched to azithromycin

2. Clinical Problem List:

Active	Chronic
Fever	Diabetes mellitus
Vomiting	Nephropathy
Productive cough	End-stage renal failure
Right persistent clinically significant macular edema	
CAPD peritonitis	

3. Potential Life-Threatening Complications:
* Sepsis
* Acute kidney failure
* Peritonitis
* Respiratory failure

4. Clinical Findings:

Organ System	Anticipated Physical Examination Findings
General	Fever, tachycardia, tachypnea
Respiratory	Productive cough, clear chest bilaterally
Cardiovascular	Normal blood pressure, no murmurs, rubs, or gallops
Gastrointestinal	Vomiting, abdominal pain, diarrhea
Neurological	Full Glasgow coma scale, no focal deficits
Musculoskeletal	No rash, joint pain, or swelling
Integumentary	No rash or skin lesions

5. Working Diagnoses:
* Campylobacter fetus bacteremia
* CAPD peritonitis
* Pneumonia

6. Relevant Investigations:

Investigation	Justification
Blood culture	To identify the causative organism of bacteremia
Peritoneal dialysis fluid culture	To identify the causative organism of peritonitis
Chest X-ray	To assess for pneumonia or other pulmonary abnormalities
Complete blood count	To assess for anemia, leukocytosis, or thrombocytopenia
Electrolytes and renal function tests	To assess for electrolyte imbalances and renal function
Liver function tests	To assess for liver dysfunction

7. Management Plan:
* Continue antibiotics for Campylobacter fetus bacteremia
* Monitor for signs and symptoms of sepsis
* Provide supportive care, including fluids and electrolytes
* Monitor renal function and adjust dialysis as needed
* Monitor respiratory status and provide oxygen therapy as needed
* Provide nutritional support as needed
* Monitor for complications and treat as appropriate

8. Executive Summary:
A 72-year-old female with diabetes mellitus, nephropathy, end-stage renal failure, and right persistent clinically significant macular edema presented with fever, vomiting, and productive cough. Blood culture grew Campylobacter fetus. She was started on antibiotics and supportive care. The patient's condition improved, and she was discharged home. The patient should be followed closely for complications.

Claude 2

1. Chronological Events

Date	Event
Prior to current admission	Diabetes mellitus with nephropathy, end stage renal failure on CAPD. Hypertension. Right CSME.Tenckhoff catheter removed June 2023 due to E. coli CAPD peritonitis.
14 November 2023	Reinsertion of Tenckhoff catheter.
3 days prior to admission	Onset of fever, vomiting and productive cough without diarrhea.
Day of admission	Admitted for fever, vomiting and cough. PDF WBC 1484 down to 69 with intraperitoneal antimicrobials.
25 November 2023	Blood culture positive for Campylobacter fetus. Started on amoxicillin-clavulanate.
27 November 2023	Repeat blood culture no growth.
29 November 2023	Changed from amoxicillin-clavulanate to azithromycin.

2. Clinical Problem List

Clinical Problem	Active/Chronic
Diabetes mellitus with nephropathy	Chronic
End stage renal failure on CAPD	Chronic
Hypertension	Chronic
Right CSME	Chronic
E. coli CAPD peritonitis (June 2023)	Resolved
Fever, vomiting, productive cough (current admission)	Active
Campylobacter fetus bacteremia	Active

3. Potential Life-Threatening Complications
- Septic shock from Campylobacter fetus bacteremia
- Acute respiratory failure from pneumonia
- Cardiac events (e.g. arrhythmia) from electrolyte abnormalities in setting of end stage renal failure

4. Anticipated Physical Examination Findings

Organ System	Expected Findings
Constitutional	Tachycardia, fever
HEENT	Oropharyngeal erythema
Respiratory	Crackles/rales, bronchial breath sounds, egophony
Cardiovascular	Tachycardia, hypotension
Abdominal	Tenderness over Tenckhoff catheter site
Neurologic	Alert and oriented

5. Working Diagnoses
- Campylobacter fetus bacteremia
- Pneumonia
- Volume depletion in setting of vomiting/poor oral intake
- Electrolyte abnormalities (e.g. hypokalemia, hyponatremia)

6. Relevant Investigations

Investigation	Justification
Sputum culture	To identify respiratory pathogen
Chest X-ray	To evaluate for pneumonia
Serum electrolytes	To assess for abnormalities that may cause cardiac issues
Kidney function tests	To monitor renal status

7. Management Plan
- Continue appropriate intravenous antibiotics (azithromycin)
- Hydration and correction of electrolyte abnormalities
- Oxygen supplementation and monitoring for respiratory failure
- Hemodynamic monitoring for sepsis
- Removal of Tenckhoff catheter may be warranted if source of infection
- Infectious diseases consultation

8. Executive Summary
72F with ESRD on CAPD complicated by Campylobacter fetus bacteremia after recent Tenckhoff catheter reinsertion presents with fever, vomiting and cough. Septic shock, pneumonia, and cardiac events are potential life-threatening issues. Management includes continued IV antibiotics, hydration, hemodynamic monitoring, and possible Tenckhoff catheter removal.

15. Clinical scenario 15

GPT-4.0

1. Chronological Events

Date	Event

July 2022	Last chemotherapy session for palliative radiotherapy
Early October 2023	Onset of persistent watery diarrhea
November 2023	Discovery of rectal mass; biopsy showing poorly differentiated carcinoma
15 November 2023	PET-CT indicating multiple carcinomas, right hydronephrosis, and urinary bladder wall thickening
22 November 2023	Blood culture positive for Gram-negative bacilli, other culture results pending

2. Clinical Problem List

Status	Clinical Issues
Active	Persistent watery diarrhea
Active	Poorly differentiated carcinoma of the rectum
Active	Synchronous carcinoma of the transverse colon
Active	Progression of carcinoma of pylorus
Active	Right hydronephrosis
Active	Diffuse urinary bladder wall thickening
Chronic	Unresectable carcinoma of pylorus

3. Potential Life-Threatening Complications
- Severe dehydration and electrolyte imbalance due to persistent diarrhea.
- Sepsis due to Gram-negative bacteremia.
- Acute kidney injury secondary to obstructive hydronephrosis.
- Progressive anemia and thrombocytopenia could lead to hemorrhage or severe infection.

4. Clinical Findings

Organ System	Anticipated Findings
General	Tired appearance
Gastrointestinal	Lower abdominal tenderness, diarrhea
Hematologic	Occasional fragmented red blood cells (indicative of microangiopathic process)
Renal	Elevated serum creatinine
Hepatic	Elevated total bilirubin and ALP

5. Working Diagnoses
- Metastatic carcinoma with primary in the pylorus and secondary in the rectum and transverse colon.
- Gram-negative sepsis.
- Acute kidney injury due to obstructive uropathy.

- Diarrhea of undetermined etiology, possibly related to underlying malignancy or a side effect of chemotherapy.

6. Relevant Investigations

Investigation	Justification
Stool culture & sensitivity	To identify causative organism of diarrhea and guide antimicrobial therapy
Complete Blood Count with differential	To assess extent of neutrophilia, anemia, and thrombocytopenia
Serum electrolytes	To evaluate for electrolyte imbalances due to diarrhea
Kidney function tests (Urea, Creatinine)	To monitor the extent of renal impairment
Liver function tests	To assess hepatic involvement and function
Imaging (e.g., ultrasound or CT of abdomen)	To evaluate hydronephrosis and urinary bladder wall thickening
Repeat PET-CT	To reassess the extent of carcinomatosis

7. Management Plan
- Initiate broad-spectrum antibiotics to cover Gram-negative organisms while awaiting sensitivity results.
- Provide supportive care for diarrhea, including fluid and electrolyte replacement.
- Address the acute kidney injury by evaluating for the need for a nephrostomy or stenting to relieve obstruction.
- Transfuse blood products as necessary to manage anemia and thrombocytopenia.
- Consider palliative care consultation for symptom management and quality of life discussions.
- Evaluate the need for further chemotherapy or radiotherapy for cancer management.

8. Executive Summary
A 76-year-old male with a history of unresectable pylorus carcinoma, now presenting with persistent watery diarrhea and diagnosed with poorly differentiated carcinoma of the rectum and progression of known malignancy. The patient is also showing signs of sepsis, acute kidney injury, and severe hematologic abnormalities. Immediate management should focus on stabilizing the patient's hemodynamic status, managing sepsis, and addressing the renal obstruction. Long-term management will require coordination of palliative care to address the patient's quality of life and ongoing cancer treatment.

cGPT-4

1. Chronological Events:

Date	Event

July 2022	Last chemotherapy for palliative radiotherapy
Early October	Onset of persistent diarrhea
November 2023	Rectal mass noted, biopsy showing poorly differentiated carcinoma
15 November	PET-CT showing rectal tumor, synchronous carcinoma of transverse colon, and others
22 November	Blood culture showing Gram-negative bacilli, awaiting culture results

2. Clinical Problem List:

Status	Clinical Issues
Active	Persistent diarrhea
Active	Poorly differentiated carcinoma (rectal mass)
Chronic	Unresectable carcinoma of pylorus
Active	Carcinoma of transverse colon
Active	Right hydronephrosis
Active	Diffuse urinary bladder wall thickening

3. Potential Life-Threatening Complications:
- Sepsis due to Gram-negative bacilli infection.
- Acute renal failure, indicated by increased serum creatinine and hydronephrosis.
- Severe anemia and thrombocytopenia, which may lead to hemorrhage or impaired clotting.

4. Clinical Findings

Organ System	Findings
General Appearance	Tired looking
Abdomen	Lower abdominal diffuse tenderness
Renal	Indirect evidence of renal impairment (hydronephrosis)
Integumentary	Possible jaundice (elevated total bilirubin)

5. Working Diagnoses:
- Metastatic colorectal cancer with progression.
- Sepsis likely secondary to urinary tract infection or colonic bacterial translocation.
- Acute renal failure secondary to obstruction (hydronephrosis).

6. Relevant Investigations:

Investigation	Justification
Stool culture	To identify causative organism of diarrhea
Continued blood culture	To identify and confirm Gram-negative bacilli
Urine culture	To identify the cause of noted pyuria and bacteriuria
Renal ultrasound	To further evaluate hydronephrosis and renal function

7. Management Plan:
- Immediate broad-spectrum antibiotic therapy to cover Gram-negative organisms, considering the risk of sepsis.
- Supportive care for diarrhea and hydration status.
- Consideration of nephrostomy to relieve hydronephrosis if indicated by renal ultrasound.
- Transfusion support for anemia and thrombocytopenia if clinically warranted.
- Pain management, nutritional support, and palliative care consultation for overall symptom control and quality of life improvement.
- Regular monitoring and assessment, with multidisciplinary team review.

8. Executive Summary:
The patient presents with multiple complications from advanced metastatic colorectal cancer, including persistent diarrhea, sepsis, and acute renal failure. Immediate management includes broad-spectrum antibiotics and supportive measures for renal impairment and hematological abnormalities. A multidisciplinary approach is required for ongoing care, with emphasis on the comfort and quality of life of the patient. Regular monitoring is essential for any changes in the patient's condition.

Gemini Pro

1. Chronological Events:

Date	Event
Early October 2023	Onset of watery diarrhea
November 2023	Rectal mass noted
November 2023	Biopsy of rectal mass reveals poorly differentiated carcinoma
15 November 2023	PET-CT scan shows rectal tumor, synchronous carcinoma of transverse colon, progression of carcinoma of pylorus, right hydronephrosis, and diffuse urinary bladder wall thickening
22 November 2023	Blood culture drawn, Gram-negative bacilli noted, culture pending
Current	Persistent diarrhea with no improvement

2. Clinical Problem List:

Active	Chronic
Persistent diarrhea	Unresectable carcinoma of pylorus
Rectal mass	Gastrojejunal bypass
Poorly differentiated carcinoma of rectum	Last chemotherapy July 2022
Synchronous carcinoma of transverse colon	Palliative radiotherapy if symptomatic
Progression of carcinoma of pylorus	

Right hydronephrosis	
Diffuse urinary bladder wall thickening	
Gram-negative bacteremia	

3. Potential Life-Threatening Complications:
* Sepsis
* Perforation of the gastrointestinal tract
* Obstruction of the gastrointestinal tract
* Renal failure
* Bladder rupture

4. Clinical Findings:

Organ System	Anticipated Physical Examination Findings
General	Tired looking, stable afebrile
Gastrointestinal	Lower abdominal diffuse tenderness, persistent watery diarrhea four to five times per day
Respiratory	No obvious consolidation on CXR

5. Working Diagnoses:
* Persistent diarrhea of unknown etiology
* Rectal carcinoma
* Synchronous carcinoma of transverse colon
* Progression of carcinoma of pylorus
* Right hydronephrosis
* Diffuse urinary bladder wall thickening
* Gram-negative bacteremia

6. Relevant Investigations:

Investigation	Justification
Stool culture	To identify the causative organism of the diarrhea
Urine culture	To identify the causative organism of the bacteriuria
Abdominal CT scan	To evaluate the extent of the gastrointestinal tumors and to look for signs of obstruction or perforation
Renal ultrasound	To assess the severity of the hydronephrosis and to rule out other causes of renal dysfunction
Cystoscopy	To evaluate the bladder wall thickening and to rule out other causes of bladder dysfunction

7. Management Plan:
* Initiate empiric antimicrobial therapy for the Gram-negative bacteremia

* Perform stool culture and urine culture to identify the causative organisms of the diarrhea and bacteriuria, respectively
* Perform abdominal CT scan, renal ultrasound, and cystoscopy to evaluate the extent of the gastrointestinal tumors, the severity of the hydronephrosis, and the bladder wall thickening, respectively
* Consult with oncology and surgical teams to discuss further management of the gastrointestinal tumors
* Consult with nephrology team to discuss management of the hydronephrosis
* Provide supportive care, including fluid and electrolyte replacement, nutritional support, and pain management

8. Executive Summary:
The patient is a 76-year-old male with a history of unresectable carcinoma of the pylorus and gastrojejunal bypass who presents with persistent diarrhea, rectal mass, and Gram-negative bacteremia. Investigations are needed to identify the cause of the diarrhea and bacteremia, and to evaluate the extent of the gastrointestinal tumors, hydronephrosis, and bladder wall thickening. Management includes empiric antimicrobial therapy, supportive care, and consultation with relevant specialists.

Claude 2

1. Chronological Events

Date	Event
Early October 2023	Onset of watery diarrhea
November 2023	Rectal mass noted
15 November 2023	Biopsy - poorly differentiated carcinoma, PET-CT showed rectal tumor, synchronous carcinoma of transverse colon, carcinoma of pylorus disease progression, right hydronephrosis and diffuse urinary bladder wall thickening
22 November 2023	Blood culture - Gram negative bacilli, urine culture - numerous WBCs, leukocyte esterase large, bacteria seen

2. Clinical Problem List

Clinical Problem	Active/Chronic
Watery diarrhea	Active
Rectal mass	Active
Poorly differentiated carcinoma of rectum	Active
Synchronous carcinoma of transverse colon	Active
Carcinoma of pylorus with disease progression	Active
Right hydronephrosis	Active
Diffuse urinary bladder wall thickening	Active
Febrile neutropenia	Active

Pancytopenia	Active
Acute kidney injury	Active

3. Potential Life-Threatening Complications
- Sepsis from neutropenia and possible bacteremia/urosepsis
- Gastrointestinal bleeding from rectal mass
- Acute kidney injury progressing to renal failure
- Electrolyte abnormalities from diarrhea leading to arrhythmias

4. Anticipated Physical Examination Findings

Organ System	Expected Findings
General	Tired looking, afebrile
Abdominal	Diffuse tenderness, palpable rectal mass
Rectal	Rectal mass

5. Working Diagnoses
- Febrile neutropenia
- Bacteremia/urosepsis
- Poorly differentiated rectal carcinoma with local progression and metastases
- Synchronous transverse colon carcinoma
- Progression of pyloric carcinoma
- Acute kidney injury
- Electrolyte abnormalities

6. Relevant Investigations

Investigation	Justification
Blood cultures	To identify bacteremia source
Urine culture and sensitivities	To identify urosepsis source
Stool culture	To identify gastrointestinal source potentially causing diarrhea
CBC, renal panel, LFTs, coagulation profile	Monitor counts, organ functions
Fluid and electrolyte panel	Assess electrolyte abnormalities
CT abdomen and pelvis	Assess metastases and hydronephrosis
ECG	Assess for arrhythmias

7. Management Plan
- Treat neutropenic sepsis aggressively with broad spectrum IV antibiotics after collecting cultures
- IV fluid resuscitation for AKI
- Electrolyte replacement
- Pain control for abdominal pain
- Bowel rest for diarrhea

- Consult surgery for diverting colostomy for rectal obstruction and bleeding risk
- Consult oncology regarding chemotherapy options
- Avoid nephrotoxic drugs
- Regular monitoring of counts, organ functions, fluid status
- Early identification and treatment of complications - sepsis, bleeding, renal failure, electrolyte imbalance

8. Executive Summary

76-year-old male with refractory gastrointestinal malignancies now admitted with diarrhea and neutropenic sepsis. Aggressively manage sepsis, acute kidney injury, electrolyte imbalance. Address cancer progression with oncology. Prevent complications like gastrointestinal bleeding, renal failure, arrhythmias.

16. Clinical scenario 16

GPT-4.0

1. Chronological Events

Date	Event
March 2023	Whipple procedure performed for carcinoma of the gallbladder.
August 2023	CT scan showed recurrence in the right upper quadrant and nodal recurrence.
2-6 Nov 2023	Cefuroxime and metronidazole administered.
4 Nov 2023	Left PTBD bile culture positive for Klebsiella pneumoniae, sensitive to amoxicillin-clavulanate.
6 Nov 2023	Initiated on Meropenem.
20 Nov 2023	Noted swinging fever and USG showed prominent right intrahepatic ducts.
27 Nov 2023	Right external-internal PTBD performed, left PTBD removed.
28 Nov 2023	Right PTBD bile culture taken. Urine routine microscopy not indicative of UTI.
29 Nov 2023	Blood culture taken.

2. Clinical Problem List

Status	Clinical Issues
Active	MRSA carrier
Active	Carcinoma of gallbladder recurrence with portal vein invasion
Active	Deranged liver function tests
Active	Persistent swinging fever since 20 November 2023
Active	Tachycardia with lowish blood pressure
Chronic	History of Whipple procedure

3. Potential Life-Threatening Complications
- Sepsis due to persistent swinging fever, tachycardia, and lowish blood pressure.
- Biliary sepsis from complicated intrahepatic ducts and PTBD.
- Portal vein thrombosis due to portal vein invasion.
- Acute kidney injury indicated by serum creatinine levels.

4. Clinical Findings

Organ System	Anticipated Findings
Cardiovascular	Tachycardia, low blood pressure.
Respiratory	Chest clear (no evidence of pneumonia or effusion).
Abdominal	Abdomen soft, bile stain output from right PTBD (evidence of drainage).
Skin	No inflammation at drip site, no signs of septic emboli.
Musculoskeletal	Calves soft (no evidence of deep vein thrombosis).

5. Working Diagnoses
- Biliary tract infection (likely secondary to Klebsiella pneumoniae per left PTBD culture).
- Recurrent gallbladder carcinoma with portal vein invasion.
- Possible sepsis or bacteremia (pending blood culture results).

6. Relevant Investigations

Investigation	Justification
Blood culture	To identify causative organism and antibiotic sensitivity for bacteremia.
Right PTBD bile culture	To confirm the presence of infection and its antibiotic sensitivity.
Repeat Liver function tests	To monitor the liver function and response to PTBD.
Serum creatinine	To assess renal function, especially in the context of sepsis.
CT abdomen	To evaluate the extent of recurrent disease and complications of PTBD.
Procalcitonin level	To evaluate for systemic inflammation suggestive of bacterial infection.

7. Management Plan
- Continue broad-spectrum antibiotics (Meropenem) until culture results are available and then de-escalate based on sensitivities.
- Monitor vital signs closely and support hemodynamics with fluid resuscitation and vasopressors if needed.
- Assess need for further drainage or revision of the PTBD if obstructed or infected.
- Consult oncology for the management of recurrent carcinoma and evaluate for palliative care if appropriate.

- Supportive care for symptoms and prevention of complications such as thromboembolism.

8. Executive Summary
The patient is a 38-year-old female with a complex medical history including MRSA carriage and recurrent gallbladder carcinoma post-Whipple procedure. She presents with persistent fever, possible sepsis, and liver function abnormalities. Management has included broad-spectrum antibiotics and biliary drainage with PTBD. Pending cultures and further investigations will guide the targeted treatment. The management plan focuses on addressing the infectious complications, monitoring and supporting organ functions, and consulting oncology for the management of her recurrent malignancy.

cGPT-4

1. Chronological Events:

Date	Event
March 2023	Whipple procedure for carcinoma of the gallbladder.
August 2023	CT shows right upper quadrant and nodal recurrence, portal vein invasion.
2-6 Nov 2023	Cefuroxime and metronidazole therapy.
4 Nov 2023	Left PTBD bile culture shows Klebsiella pneumoniae.
6 Nov 2023	Start of Meropenem therapy.
20 Nov 2023	Noted persistent swinging fever; Deranged liver function test.
27 Nov 2023	Right external-internal PTBD done, left PTBD removed.
28 Nov 2023	Right PTBD bile culture taken.
29 Nov 2023	Blood culture taken; persistent fever and tachycardia.

2. Clinical Problem List:

Status	Clinical Issue
Active	MRSA carrier status.
Chronic	Carcinoma of the gallbladder with recurrence post-Whipple.
Active	Portal vein invasion.
Active	Persistent swinging fever since 20 November 2023.
Active	Deranged liver function test.
Active	Tachycardia and low blood pressure.

3. Potential Life-Threatening Complications:
- Sepsis due to ongoing infection potentially indicated by swinging fever, tachycardia, and deranged liver function tests.
- Portal vein thrombosis due to invasion by carcinoma, which can lead to portal hypertension and variceal bleeding.

4. Clinical Findings:

Organ System	Anticipated Findings
Cardiovascular	Tachycardia, possibly hypotension.
Abdominal	Soft abdomen, potentially with signs of hepatomegaly.
Skin	Bile stain output from right PTBD site.

5. Working Diagnoses:
- Cholangitis due to Klebsiella pneumoniae infection.
- Post-operative recurrence of gallbladder carcinoma.
- Potential sepsis secondary to persistent infection.

6. Relevant Investigations:

Investigation	Justification
Right PTBD bile culture	To identify causative organism and appropriate antimicrobial therapy.
Blood culture	To check for systemic infection and sepsis.
Liver function tests	To monitor liver function and effectiveness of PTBD.
Imaging studies (CT/MRI)	To assess extent of carcinoma recurrence and portal vein involvement.

7. Management Plan:
- Continue with Meropenem until culture results are available and reassess antibiotic therapy based on sensitivities.
- Close monitoring of vital signs and clinical status to detect signs of sepsis or deterioration.
- Regular blood tests including liver function tests, CRP, and full blood count.
- Consideration of anticoagulation if portal vein thrombosis is confirmed.
- Multidisciplinary team involvement, including oncology, for management of gallbladder carcinoma recurrence.
- Pain management and supportive care as needed.
- Patient education on signs of infection and when to seek immediate care.

8. Executive Summary:

The patient is a 38-year-old female with a history of MRSA carriage and recurrent gallbladder carcinoma post-Whipple procedure, presenting with signs of infection and deranged liver tests. Management includes ongoing Meropenem therapy with close monitoring and further investigations to guide treatment. The multidisciplinary approach is essential for the management of carcinoma recurrence and potential sepsis. Immediate attention to changes in clinical status is imperative for the prevention of life-threatening complications.

Gemini Pro

1. Chronological Events:

Date	Clinical Event
March 2023	Whipple procedure performed for carcinoma of gallbladder.
August 2023	CT scan reveals right upper quadrant and nodal recurrence, portal vein invasion, and lymph node involvement.
November 2023	Patient admitted for deranged liver function tests.
November 2023	Breakthrough fever occurs, prompting urgent left percutaneous transhepatic biliary drainage (PTBD).
November 2023	Patient transferred to Hospital T for further management.
November 2023	Patient transferred back due to persistent fever on meropenem.
November 2023	USG reveals prominent right intrahepatic ducts.
November 2023	Right external-internal PTBD performed, left PTBD removed.

2. Clinical Problem List:

Active Problems	Chronic Problems
Persistent swinging fever	Carcinoma of gallbladder
Tachycardia	Methicillin-resistant Staphylococcus aureus (MRSA) carrier
Low blood pressure	

3. Potential Life-Threatening Complications:
- Sepsis
- Multi-organ failure
- Cholangitis
- Liver failure

4. Clinical Findings:

Organ System	Findings
General	Persistent swinging fever, tachycardia, low blood pressure
Respiratory	Chest clear
Cardiovascular	Tachycardia, low blood pressure
Gastrointestinal	Bile stain output from right PTBD
Musculoskeletal	Calves soft
Skin and Soft Tissue	Drip site not inflamed

5. Working Diagnoses:
- Cholangitis
- Biliary sepsis
- Portal vein thrombosis

6. Relevant Investigations:

Investigation	Justification
Blood culture	To identify the causative organism of the bloodstream infection.
Urine routine microscopy	To rule out urinary tract infection as a source of fever.
Liver function tests	To assess liver function and monitor response to treatment.
Abdominal ultrasound	To evaluate the biliary system and assess for complications such as cholangitis or biliary obstruction.
CT scan of the abdomen	To further evaluate the extent of the biliary and liver disease, and to assess for complications such as portal vein thrombosis.

7. Management Plan:
- Administer broad-spectrum antibiotics to cover potential pathogens, including Klebsiella pneumoniae and other Enterobacteriaceae.
- Perform urgent biliary drainage to relieve biliary obstruction and reduce the risk of cholangitis.
- Monitor liver function tests closely and provide supportive care as needed.
- Consider anticoagulation therapy if portal vein thrombosis is confirmed.
- Provide nutritional support as needed.

8. Executive Summary:
The patient is a 38-year-old female with a history of carcinoma of the gallbladder and MRSA carriage who presented with persistent swinging fever, tachycardia, and low blood pressure. Investigations revealed cholangitis and possible biliary sepsis. The patient was treated with broad-spectrum antibiotics, biliary drainage was performed, and supportive care was provided. Further investigations are needed to assess the extent of the biliary and liver disease and to evaluate for complications such as portal vein thrombosis.

Claude 2

1. Chronological Events

Date	Event
March 2023	Whipple surgery done for carcinoma of gallbladder.
August 2023	CT scan showed recurrence in right upper quadrant and nodes.
November 2023	Admitted for deranged LFTs.
November 2023	Left PTBD done for fever.
20 November 2023	USG showed prominent right ducts.
27 November 2023	Right external-internal PTBD done, left PTBD removed.
Since 20 November 2023	Persistent swinging fever.

2. Clinical Problem List

Problem	Active/Chronic
Carcinoma of gallbladder, status post Whipple	Chronic
Recurrent disease in liver and nodes	Active
Deranged LFTs	Active
Fever	Active
Prominent right ducts	Active

3. Potential Life-Threatening Complications
- Sepsis from biliary source of infection
- Liver failure from biliary obstruction
- Disease progression with metastases

4. Anticipated Physical Examination Findings

System	Expected Findings
General	Tachycardia, hypotension
Respiratory	Clear chest
Cardiovascular	Tachycardia
Abdominal	Soft abdomen, no peritonitis
Extremities	No calf tenderness

5. Working Diagnoses
- Biliary sepsis
- Mechanical biliary obstruction
- Liver dysfunction from biliary obstruction
- Recurrent cancer with liver invasion

6. Relevant Investigations

Investigation	Justification
Blood cultures	To identify bacteremia source
Bile cultures	To identify biliary infection
Imaging (MRI/CT)	Assess cancer recurrence and biliary obstruction
LFTs	Assess liver dysfunction from biliary obstruction
Tumor markers	Monitor cancer progression

7. Management Plan
- Initiate broad spectrum antibiotics for suspected biliary sepsis
- Urgent imaging to assess biliary obstruction and cancer progression
- Interventional radiology for biliary drainage if obstruction identified
- Surgical resection if localized recurrence amenable to surgery
- Palliative stenting if widespread disease with biliary obstruction

- Address cancer progression with chemotherapy/radiation as indicated
- Supportive care with IV fluids, nutrition, pain control
- Monitor for and mitigate complications of sepsis and liver failure

8. Executive Summary
38F with recurrent gallbladder cancer after Whipple surgery now with swinging fevers concerning for biliary sepsis. Requires broad spectrum antibiotics, imaging to assess recurrent cancer and biliary drainage, interventional radiology for biliary decompression if obstructed, and addressing cancer progression. Close monitoring for sepsis and liver failure complications.

17. Clinical scenario 17

GPT-4.0

1. Chronological Events

Date	Event
14 Oct 2023	Patient intubated and started on Sulbactam + Moxifloxacin and methylprednisolone.
15 Oct 2023	Transferred to Hospital N; treatment escalated to Imipenem + Moxifloxacin.
16 Oct 2023	Bronchoalveolar lavage culture showed Candida tropicalis; other PCRs negative.
17 Oct 2023	Patient extubated.
18 Oct 2023	Weaned to nasal cannula.
25 Oct 2023	CT pulmonary angiogram showed poor opacification and ground glass opacities in the lungs.
28 Oct 2023	Noted deranged liver function; transferred to Hospital Q.
28 Oct 2023	CT revealed emphysematous cystitis, tiny distal common duct stones and sludges.
31 Oct 2023	ERCP performed; impression of recurrent pyogenic cholangitis.
3 Nov 2023	Blood culture grew Enterococcus faecium.
6-7 Nov 2023	Repeat ERCP with stent insertion due to swollen papilla.
16-21 Oct 2023	Treated with Piperacillin-tazobactam.
22 Oct 2023	Started on Meropenem.
8 Nov 2023	Started on Vancomycin.

2. Clinical Problem List

Status	Clinical Issues
Active	Aspiration pneumonia
Active	Suspected transient ischemic attack
Active	Bilateral pneumonitis

Active	Emphysematous cystitis
Active	Recurrent pyogenic cholangitis
Active	Deranged liver function
Chronic	Bulbous urethral stricture
Chronic	Hypertension
Chronic	Asthma

3. Potential Life-Threatening Complications

- Respiratory failure from bilateral pneumonitis and aspiration pneumonia.
- Septic shock from recurrent pyogenic cholangitis and blood culture positive for Enterococcus faecium.
- Acute kidney injury secondary to sepsis or contrast-induced nephropathy.
- Stroke from transient ischemic attack.

4. Clinical Findings

Organ System	Anticipated Findings
Respiratory	Decreased breath sounds, crackles, wheezing
Cardiovascular	Normal heart sounds, no signs of endocarditis
Abdominal	Soft, non-tender abdomen
Extremities	No edema, normal peripheral pulses
Neurological	Alertness, no focal neurological deficits

5. Working Diagnoses

- Aspiration pneumonia
- Transient ischemic attack
- Bilateral pneumonitis
- Emphysematous cystitis
- Recurrent pyogenic cholangitis

6. Relevant Investigations

Investigation	Justification
Repeat CT brain	To assess progression or resolution of atrophic changes and rule out further ischemic events.
Liver function tests	To monitor liver status and response to treatment for cholangitis.
Repeat blood cultures	To ensure the resolution of bacteremia with Enterococcus faecium.
Follow-up CT pelvis	To evaluate the resolution of emphysematous cystitis.
Echocardiogram	To exclude endocarditis given the bacteremia with Enterococcus faecium.

7. Management Plan

- Continue respiratory support as needed, with oxygen therapy and possible non-invasive or invasive ventilation if respiratory status worsens.
- Monitor hemodynamic status closely and manage blood pressure to prevent further ischemic events.
- Adjust antibiotic therapy based on culture results and antibiotic sensitivity, ensuring adequate coverage for Enterococcus faecium.
- Consider antifungal treatment for Candida tropicalis if indicated by clinical status and response to antibiotics.
- Monitor liver function tests closely and manage cholangitis aggressively to prevent sepsis and liver failure.
- Implement deep vein thrombosis prophylaxis given the patient's immobilization and recent travel history.
- Involve urology for the management of emphysematous cystitis and monitor for urinary tract infections.
- Regularly reassess the need for the biliary stent and plan for

8. Executive Summary
The patient is an 86-year-old male with a history of bulbous urethral stricture, hypertension, and asthma who suffered an unwitnessed fall and aspiration pneumonia, along with a suspected transient ischemic attack, during travel in China. He has undergone intubation and received escalating antibiotic therapy with a noted Enterococcus faecium bacteremia and recurrent pyogenic cholangitis. Management has included bronchoscopic evaluation, bronchoalveolar lavage, and ERCP with stent insertion. Current challenges include respiratory management, liver function derangement, and the prevention of septic complications. The management plan involves continued antibiotic therapy tailored to culture results, close monitoring of respiratory and liver status, and necessary interventions, including urological assessment and anticoagulation prophylaxis.

cGPT-4

1. Chronological Events:
Date	Event
14 October	Intubated and started on Sulbactam + Moxifloxacin with methylprednisolone
15 October	Transferred to Hong Kong, admitted to Hospital N
16 October	BAL culture positive for Candida tropicalis
17 October	Extubated
18 October	Weaned to nasal cannula
25 October	CT pulmonary angiogram showed patchy ground glass opacities, fibrosis/atelectasis
28 October	Noted deranged liver function, transferred to Hospital Q

28 October	CT shows emphysematous cystitis, tiny distal common duct stones and sludges, fibrosis and traction bronchiectasis
31 October	ERCP performed, mildly dilated common bile duct
3 November	Blood culture grew Enterococcus faecium
6 & 7 November	Repeat ERCP with stent insertion

2. Clinical Problem List:

Status	Clinical Issue
Active	Aspiration pneumonia
Active	Suspected transient ischemic attack
Active	Bilateral pneumonitis
Active	Emphysematous cystitis
Active	Recurrent pyogenic cholangitis
Active	Deranged liver function
Chronic	Bulbous urethral stricture
Chronic	Hypertension
Chronic	Asthma

3. Potential Life-Threatening Complications:
- Respiratory failure from aspiration pneumonia and bilateral pneumonitis.
- Septic shock from bloodstream infection with Enterococcus faecium.
- Acute ischemic stroke from suspected transient ischemic attack.

4. Clinical Findings:

Organ System	Anticipated Findings
Respiratory	Decreased breath sounds, wheezing
Cardiovascular	Hypertension, regular rhythm
Abdominal	Soft, non-tender
Neurological	Potential focal neurological deficits

5. Working Diagnoses:
- Aspiration pneumonia
- Bilateral pneumonitis
- Transient ischemic attack (TIA)
- Emphysematous cystitis
- Recurrent pyogenic cholangitis

6. Relevant Investigations:

Investigation	Justification
Repeat CT brain	To assess for progression of atrophic changes

Repeat CT thorax	To monitor resolution of bilateral pneumonitis
Liver function tests	To monitor deranged liver function
Repeat ultrasound abdomen	To assess for resolution of emphysematous cystitis and biliary sludges
Echocardiogram	To rule out cardiac sources of emboli in TIA
Blood cultures	To monitor for ongoing bloodstream infection

7. Management Plan:
- Continue Meropenem and Vancomycin to cover for Enterococcus faecium and other potential bacterial pathogens.
- Monitor respiratory status closely and provide supportive care as needed, including oxygen therapy and possibly non-invasive ventilation if respiratory distress reoccurs.
- Review and adjust antibiotic therapy based on culture results and sensitivity patterns.
- Monitor liver function and manage any identified complications, such as cholangitis, with both medical and possibly further endoscopic interventions.
- Follow up with neurological evaluation to assess for any sequelae of the suspected TIA and adjust management accordingly.
- Schedule a repeat CT pelvis in 1 month to assess for resolution of emphysematous cystitis as recommended by urology.
- Consider anticoagulation therapy if TIA is confirmed and depending on the risk assessment for stroke versus bleeding complications.

8. Executive Summary:
An 86-year-old male with a history of bulbous urethral stricture, hypertension, and asthma experienced an aspiration pneumonia with suspected TIA during travel. Following empirical antibiotic treatment and mechanical ventilation, the patient was extubated and weaned to nasal cannula. Complications include emphysematous cystitis and recurrent pyogenic cholangitis. Management includes Meropenem and Vancomycin, close monitoring of respiratory and liver function, and follow-up imaging. Blood cultures indicated Enterococcus faecium infection, which is being addressed with targeted antibiotic therapy.

Gemini Pro

1. Chronological Events:
Date	Clinical Issue
Prior to 14 October 2023	Bulbous urethral stricture, Hypertension, Asthma
14 October 2023	Aspiration pneumonia with suspected transient ischemic attack
15 October 2023	Transferred to Hong Kong and admitted to Hospital N
16 October 2023	BAL culture: Candida tropicalis
17 October 2023	Extubated
18 October 2023	Weaned down to nasal cannula

25 October 2023	CT pulmonary angiogram: poor opacification of the subsegmental branch of left lower lobe lateral basal segment pulmonary artery. Patchy ground glass opacities and fibrosis/atelectasis at both lungs.
28 October 2023	Transferred to Hospital Q
28 October 2023	Deranged liver function
29 October 2023	Urine culture: Candida glabrata
31 October 2023	ERCP performed: mildly dilated common bile duct but with no common bile duct stone, intrahepatic ducts dilated, impression: recurrent pyogenic cholangitis. Urine culture: Candida glabrata
3 November 2023	Blood culture: Enterococcus faecium
6 November 2023	Repeat ERCP: swollen papilla, common bile duct cannulated with stent insertion
7 November 2023	Repeat ERCP: swollen papilla, common bile duct cannulated with stent insertion. Bile culture: Enterococcus faecium. Blood culture: Enterococcus faecium

2. Clinical Problem List:

Active	Chronic
Aspiration pneumonia	Bulbous urethral stricture
Deranged liver function	Hypertension
Recurrent pyogenic cholangitis	Asthma
Emphysematous cystitis	
Candida glabrata funguria	
Enterococcus faecium bacteremia	

3. Potential Life-Threatening Complications:
* Sepsis
* Respiratory failure
* Liver failure
* Cholangitis

4. Clinical Findings:

Organ System	Anticipated Findings
Respiratory	Clear chest auscultation
Cardiovascular	Dual heart sounds
Gastrointestinal	Soft, non-tender abdomen
Genitourinary	No suprapubic tenderness
Musculoskeletal	Soft calves, no lower edema

5. Working Diagnoses:
* Aspiration pneumonia

* Emphysematous cystitis
* Recurrent pyogenic cholangitis
* Enterococcus faecium bacteremia
* Candida glabrata funguria

6. Relevant Investigations:

Investigation	Justification
Blood cultures	To identify the causative organism of bacteremia
Urine cultures	To identify the causative organism of funguria
Liver function tests	To assess liver function and monitor for worsening
Abdominal ultrasound	To assess for biliary dilatation and choledocholithiasis
Chest X-ray	To assess for pneumonia and monitor for progression or resolution

7. Management Plan:
* Continue antibiotics for Enterococcus faecium bacteremia
* Monitor liver function tests closely and provide supportive care as needed
* Consider antifungal therapy for Candida glabrata funguria
* Perform repeat urine cultures to monitor for resolution of funguria
* Schedule follow-up appointments with relevant specialists for ongoing management of chronic conditions

8. Executive Summary:
The patient is an 86-year-old male with a history of aspiration pneumonia, emphysematous cystitis, recurrent pyogenic cholangitis, Enterococcus faecium bacteremia, and Candida glabrata funguria. He is currently being treated with antibiotics for bacteremia and is being monitored for liver function. A management plan has been developed to address the patient's active clinical problems and prevent complications.

Claude 2

1. Chronological Events

Date	Event
14 October 2023	Diagnosed with aspiration pneumonia and suspected TIA in Jiangxi, China. Started on Sulbactam + Moxifloxacin and methylprednisolone. Intubated.
15 October 2023	Transferred to Hospital N in Hong Kong.
16 October 2023	Bronchoalveolar lavage culture grew Candida tropicalis.
17 October 2023	Extubated.
18 October 2023	Weaned to nasal cannula.
25 October 2023	CT pulmonary angiogram showed subsegmental PE in left lower lobe.

28 October 2023	Transferred to Hospital Q.
31 October 2023	ERCP showed mild CBD dilation, no stones.
3 November 2023	Blood culture grew Enterococcus faecium.
6-7 November 2023	Repeat ERCP, CBD stented.

2. Clinical Problem List

Problem	Active/Chronic
Aspiration pneumonia	Active
Suspected TIA	Active
Emphysematous cystitis	Active
Sludged common bile duct	Active
Recurrent pyogenic cholangitis	Active
Enterococcal bacteremia	Active
Bulbous urethral stricture	Chronic
Hypertension	Chronic
Asthma	Chronic

3. Potential Life-Threatening Complications
- Worsening respiratory failure and hypoxemia from aspiration pneumonia
- Recurrence of TIA → CVA with permanent neurological deficits
- Sepsis from enterococcal bacteremia
- Emphysematous cystitis → urosepsis
- Cholangitis from biliary obstruction leading to septic shock

4. Anticipated Physical Examination Findings

System	Expected Findings
Respiratory	Bilateral coarse crackles
Cardiovascular	Tachycardia, new murmur
Abdominal	RUQ tenderness, Murphy's sign
Genitourinary	Suprapubic tenderness
Neurological	Any new neurological deficits

5. Working Diagnoses
- Aspiration pneumonia
- TIA
- Enterococcal urinary tract infection with bacteremia
- Acute cholangitis
- Emphysematous cystitis

6. Relevant Investigations

Investigation	Justification

|-|-|
Sputum & blood cultures	To identify causative organisms for pneumonia and bacteremia
Liver function tests, lipase	To assess for cholangitis and pancreatitis
CT head	To evaluate for cerebrovascular disease
Transthoracic echocardiogram	To evaluate for endocarditis source of bacteremia
Renal ultrasound	To evaluate emphysematous cystitis

7. Management Plan
- Administer appropriate intravenous antibiotics (e.g. piperacillin-tazobactam)
- Ensure adequate hydration and nutrition
- Monitor vitals and urine output closely
- Repeat blood cultures to document clearance
- Perform echocardiogram to evaluate for endocarditis
- Avoid nephrotoxic medications given acute kidney injury risk
- Consult Urology for evaluation of emphysematous cystitis
- Consult Neurology for ischemic stroke prevention strategies
- Address comorbidities like hypertension and asthma
- Early rehabilitation to prevent deconditioning

8. Executive Summary
This 86-year-old man has multiple active issues including aspiration pneumonia, enterococcal bacteremia, emphysematous cystitis, and possible TIA in the setting of several chronic medical conditions. He requires broad-spectrum IV antibiotics, close monitoring for worsening sepsis, investigation for endocarditis, and multidisciplinary care to address his complex needs. Preventing further complications and restoring baseline function are priorities.

18. Clinical scenario 18

GPT-4.0

1. Chronological Events:
Date	Event
26 Nov - 2 Dec 2023	Travelled to Okinawa; intake of raw fish
30 Nov 2023	Onset of symptoms: fever, cough, sore throat, runny nose, myalgia
2 Dec 2023	Admitted for fever, diarrhea, and vomiting; started on Amoxicillin-clavulanate and Doxycycline
3 Dec 2023	CXR showed RLZ infiltrates; started antimicrobials

2. Clinical Problem List:
Status	Clinical Issues

Active	Fever, cough, sore throat, runny nose, myalgia
Active	Diarrhea and vomiting
Active	Pulmonary infiltrates on CXR
Chronic	Diabetes mellitus (HbA1c 6.4%)
Chronic	Fatty liver
Chronic	Right breast mass (followed up by Hospital T)
Chronic	History of carcinoma of the cervix with hysterectomy (>10 years)

3. Potential Life-Threatening Complications:
- Septic shock due to potential systemic infection
- Acute respiratory distress syndrome (ARDS) due to pulmonary infiltrates
- Severe dehydration and electrolyte imbalance due to diarrhea and vomiting

4. Clinical Findings:

Organ System	Anticipated Findings
Respiratory	Tachypnea, decreased breath sounds, crackles in RLZ
Gastrointestinal	Abdominal tenderness, hyperactive bowel sounds
Cardiovascular	Tachycardia, hypotension (if septic)
General	Fever, diaphoresis

5. Working Diagnoses:
- Community-acquired pneumonia
- Gastroenteritis (considering recent travel and food intake)
- Differential includes foodborne illness, traveler's diarrhea

6. Relevant Investigations:

Investigation	Justification
Blood cultures	To identify causative organism for sepsis
Stool cultures	To identify pathogen causing diarrhea
Repeat CXR	To monitor progression of pulmonary infiltrates
Liver function tests	To assess possible liver involvement
Influenza and other respiratory virus PCR	To rule out viral etiologies
Serologies for common foodborne illnesses	Considering intake of raw fish

7. Management Plan:
- Continue Amoxicillin-clavulanate and Doxycycline while awaiting culture results.
- Supportive care including hydration and electrolyte management.
- Monitor for signs of sepsis and initiate sepsis protocol if indicated.
- Follow up on breast mass and manage comorbid conditions (diabetes, fatty liver).
- Consider consultation with infectious disease for possible foodborne illness.
- Patient education on safe eating practices while traveling.

8. Executive Summary:
The patient, a 62-year-old female with a background of diabetes, fatty liver, and previous carcinoma of the cervix, presented with symptoms suggestive of both respiratory and gastrointestinal infections following recent travel. She is being treated empirically for community-acquired pneumonia and gastroenteritis with broad-spectrum antibiotics. Investigations are underway to identify the causative pathogens. Close monitoring for potential life-threatening complications such as sepsis or ARDS is essential, along with the management of her chronic conditions.

cGPT-4

1. Chronological Events:

Date	Clinical Event
26 Nov 2023	Travel to Okinawa
30 Nov 2023	Onset of fever, cough, sore throat, runny nose, and myalgia
2 Dec 2023	Admission with fever, diarrhea, vomiting
3 Dec 2023	CXR shows pulmonary infiltrates; Start of Amoxicillin-clavulanate and Doxycycline

2. Clinical Problem List:

Status	Clinical Issues
Active	Fever, cough, sore throat, runny nose, myalgia, diarrhea, vomiting, shortness of breath, pulmonary infiltrates
Chronic	Diabetes mellitus, Fatty liver, History of carcinoma of cervix with hysterectomy

3. Potential Life-Threatening Complications:
- Pulmonary infiltrates could indicate a severe respiratory infection which, if not appropriately managed, could progress to respiratory failure.
- Dehydration secondary to diarrhea and vomiting could lead to acute kidney injury, particularly in the context of diabetes mellitus.

4. Clinical Findings:

Organ System	Anticipated Findings
Respiratory	Crackles or decreased breath sounds in RLZ
Gastrointestinal	Signs of dehydration due to diarrhea and vomiting
General	Fever, signs of systemic infection

5. Working Diagnoses:

- Community-acquired pneumonia
- Gastroenteritis, possibly secondary to raw fish intake
- Differential includes travel-related infections

6. Relevant Investigations:

Investigation	Justification
Repeat chest X-ray	To monitor progression of pulmonary infiltrates
Blood cultures	To identify causative organism of potential sepsis
Stool cultures	To determine cause of diarrhea, possible infectious etiology
Influenza and other respiratory virus PCR	To rule out viral causes of respiratory symptoms

7. Management Plan:
- Continue Amoxicillin-clavulanate and Doxycycline until culture results are available, with careful monitoring of clinical response.
- Supportive care, including hydration and symptomatic relief for cough and sore throat.
- Close monitoring of blood sugar levels due to the patient's diabetes.
- Consideration for early oral switch for antibiotics as per recovery.
- Daily review by a nurse or similar healthcare professional.
- Weekly review in a multidisciplinary team meeting, as per OPAT program structure.
- If diarrhea persists, consider adding targeted therapy against suspected gastrointestinal pathogens.

8. Executive Summary:
The patient presents with symptoms suggestive of a respiratory infection potentially complicated by a gastrointestinal illness, against a background of chronic medical conditions. Management consists of continued empiric antimicrobial therapy tailored to culture results, supportive care, and close monitoring. The patient should be reviewed daily and assessed at weekly intervals by a multidisciplinary team. Ongoing evaluations and adjustments to the management plan will be necessary based on the patient's clinical course and investigation results.

Gemini Pro

1. Chronological Events:

Date	Event
26 November 2023 - 2 December 2023	Travel to Okinawa, Japan
30 November 2023	Onset of fever, cough, sore throat, runny nose, and myalgia
2 December 2023	Diarrhea with five to six times watery loose stool
2 December 2023	Admission to the hospital

| 3 December 2023 | Chest X-ray shows pulmonary infiltrates over right lower zone (RLZ) |
| 3 December 2023 | Initiation of Amoxicillin-clavulanate and Doxycycline |

2. Clinical Problem List:

Active	Chronic
Fever	Diabetes mellitus
Cough	Fatty liver
Sore throat	Carcinoma of cervix (status post hysterectomy)
Runny nose	
Myalgia	
Diarrhea	
Shortness of breath	
Pulmonary infiltrates on CXR	

3. Potential Life-Threatening Complications:
* Sepsis
* Respiratory failure
* Acute kidney injury
* Dehydration
* Electrolyte imbalance

4. Clinical Findings:

Organ System	Anticipated Physical Examination Findings
Respiratory	Tachypnea, decreased breath sounds over right lower lung
Cardiovascular	Tachycardia, hypotension
Gastrointestinal	Abdominal pain, tenderness, and distension
Neurological	Lethargy, confusion
Musculoskeletal	Myalgia

5. Working Diagnoses:
* Community-acquired pneumonia (CAP)
* Gastroenteritis
* Urinary tract infection (UTI)

6. Relevant Investigations:

Investigation	Justification
Complete blood count (CBC) with differential	To assess for anemia, leukocytosis, and neutrophilia
Serum electrolytes	To assess for dehydration and electrolyte imbalance

Blood urea nitrogen (BUN) and creatinine	To assess for renal function
Liver function tests (LFTs)	To assess for liver function
Urinalysis and urine culture	To assess for UTI
Sputum culture and sensitivity	To identify the causative organism of CAP
Chest X-ray	To assess for the extent of pneumonia

7. Management Plan:
* Continue antibiotics (Amoxicillin-clavulanate and Doxycycline) for CAP |
* Intravenous fluids and electrolytes for dehydration |
* Antidiarrheal medication for diarrhea |
* Monitor vital signs and oxygen saturation |
* Monitor renal function and liver function |
* Provide supportive care |

8. Executive Summary:
The patient is a 62-year-old female with a history of diabetes mellitus, fatty liver, and carcinoma of the cervix who presented with fever, cough, sore throat, runny nose, myalgia, and diarrhea. Chest X-ray showed pulmonary infiltrates over the right lower zone. The working diagnoses are CAP, gastroenteritis, and UTI. The management plan includes antibiotics, intravenous fluids and electrolytes, antidiarrheal medication, and supportive care.

Claude 2

1. Chronological Events
Date	Events
30 November 2023	Onset of fever, cough, sore throat, runny nose, myalgia
1 December 2023	Developed diarrhea (5-6 times watery loose stools per day) and vomiting
2 December 2023	Admitted to hospital for persistent fever
3 December 2023	Chest X-ray showed RLL infiltrates; started on amoxicillin-clavulanate and doxycycline

2. Clinical Problem List
Problem	Active/Chronic
Fever	Active
Cough	Active
Sore throat	Active
Runny nose	Active
Myalgia	Active
Diarrhea	Active

Vomiting	Active
RLL infiltrates on CXR	Active
Diabetes mellitus	Chronic
Fatty liver	Chronic
History of cervical cancer	Chronic

3. Potential Life-Threatening Complications
- Septic shock from bacterial infection
- Acute respiratory failure from pneumonia

4. Anticipated Physical Examination Findings

System	Expected Findings
General	Fever, tachycardia
HEENT	Pharyngeal erythema, tonsillar exudates
Respiratory	Crackles/rales in RLL, tachypnea
Abdominal	Hyperactive bowel sounds
Neurologic	Normal

5. Working Diagnoses
- Bacterial pneumonia
- Gastroenteritis
- Traveler's diarrhea

6. Relevant Investigations

Investigation	Justification
Blood cultures	To identify bacterial pathogen causing fever and pneumonia
Sputum culture	To identify bacterial pathogen causing pneumonia
Stool culture	To identify bacterial or viral pathogen causing diarrhea
Chest CT scan	To further characterize RLL infiltrates concerning for pneumonia

7. Management Plan
- Continue antibiotics to treat presumed bacterial pneumonia
- IV fluids for hydration
- Antiemetics for vomiting
- Avoid antibiotics for likely viral gastroenteritis once cultures negative
- Monitor for complications like septic shock
- Provide supplemental oxygen for hypoxemia
- Consider escalation of care with respiratory decline

8. Executive Summary

62F with recent travel presented with fever, cough, diarrhea. Found to have RLL infiltrates concerning for pneumonia. She was started on antibiotics. Recommend continuing antibiotics, monitoring for complications like sepsis, obtaining cultures, and escalating care if respiratory status declines.

19. Clinical scenario 19

GPT-4.0

1. Chronological Events

Date	Event
1 Nov 2023	Patient admitted with fever. Blood culture taken, resulting in Escherichia coli growth.
2 Nov 2023	Ultrasound performed, showing Bilobar multifocal HCC.
17 Nov 2023	Transfer to Hospital Q for further management. CT abdomen and pelvis show lesions in liver.
17 Nov 2023	Repeat blood culture, again showing Escherichia coli.

2. Clinical Problem List

Status	Clinical Issues
Active	Fever
Active	Escherichia coli bacteremia
Active	Suspected liver abscesses
Chronic	Diabetes mellitus
Chronic	Hepatitis B virus related hepatocellular carcinoma
Chronic	History of breast cancer, status post mastectomy
Chronic	Schizophrenia
Chronic	Glaucoma
Chronic	History of bleeding duodenal ulcer

3. Potential Life-Threatening Complications
- Sepsis from Escherichia coli bacteremia
- Liver abscess rupture or dissemination
- Decompensated liver cirrhosis from underlying hepatocellular carcinoma
- Upper gastrointestinal bleeding from the history of duodenal ulcer

4. Clinical Findings

Organ System	Anticipated Findings
General	Possible fever, signs of chronic illness
Cardiovascular	Normal heart sounds, no murmur or signs of endocarditis
Abdominal	Soft, non-tender abdomen (no current signs of acute distress)

| Neurological | Mental status changes (if present, could be related to schizophrenia or sepsis) |

5. Working Diagnoses
- Escherichia coli bacteremia
- Bilobar multifocal hepatocellular carcinoma (HCC)
- Potential early liver abscess formation
- Chronic management of schizophrenia, glaucoma, and history of duodenal ulcer

6. Relevant Investigations
Investigation	Justification
Repeat blood cultures	To monitor ongoing bacteremia and effectiveness of treatment
Liver function tests (LFTs)	To assess liver status and potential damage from HCC or abscess
CT or MRI liver with contrast	To evaluate liver abscesses or progression of HCC
Endoscopy	To assess for recurrence of duodenal ulcer if symptoms suggest

7. Management Plan
- Continue entecavir for Hepatitis B.
- Appropriate antibiotic therapy guided by sensitivities for Escherichia coli bacteremia.
- Monitor blood glucose and adjust diabetes medications as necessary.
- Evaluate and manage schizophrenia with psychiatric consultation if indicated.
- Ophthalmology review for glaucoma management.
- Gastroenterology consult for history of duodenal ulcer and potential GI bleeding risk.
- Surgical/oncology review for HCC management and assessment of potential liver abscess.

8. Executive Summary
The patient is a 78-year-old female with a complex medical history including diabetes, Hepatitis B with hepatocellular carcinoma, and schizophrenia. She was admitted with fever and found to have Escherichia coli bacteremia and potential early liver abscess formation. Immediate concerns include managing the bacteremia and liver lesions, as well as monitoring for potential complications such as sepsis or liver dysfunction. A multi-disciplinary approach is required for her chronic conditions, with ongoing assessment and management of her HCC, mental health, and risk of duodenal ulcer bleeding.

cGPT-4

1. Chronological Events:
| Date | Clinical Issues |

1 November 2023	Admitted with fever; Blood culture positive for Escherichia coli
2 November 2023	Ultrasound: Bilobar multifocal hepatocellular carcinoma (HCC)
17 November 2023	CT abdomen/pelvis: Increased size of HCC lesions; possible abscess

2. Clinical Problem List:

Status	Clinical Issues
Active	Escherichia coli bacteremia
Active	Possible early abscess formation in HCC lesions
Chronic	Diabetes mellitus
Chronic	Hepatitis B virus-related hepatocellular carcinoma
Chronic	Carcinoma of right breast (post-mastectomy, on Letrozole)
Chronic	Schizophrenia
Chronic	Glaucoma
Chronic	History of bleeding duodenal ulcer

3. Potential Life-Threatening Complications:
- Septicemia due to Escherichia coli bacteremia
- Hepatic abscess formation leading to sepsis
- Acute exacerbation of underlying chronic conditions

4. Clinical Findings:

Organ System	Anticipated Findings
Cardiovascular	Heart sounds dual, no murmur
Gastrointestinal	Abdomen soft, non-tender
Other	No peripheral signs of infective endocarditis

5. Working Diagnoses:
- Recurrent Escherichia coli bacteremia
- Progression of hepatocellular carcinoma with possible early abscess formation

6. Relevant Investigations:

Investigation	Justification
Repeat Blood cultures	To monitor ongoing bacteremia and effectiveness of treatment
Liver function tests (LFTs)	To assess liver function and impact of HCC
Repeat CT abdomen/pelvis	To follow up on the progression of HCC lesions and assess for abscess formation

7. Management Plan:
- Continue entecavir for Hepatitis B and hepatocellular carcinoma management.
- Initiate broad-spectrum intravenous antibiotics, taking into account previous blood culture sensitivity and patient's allergy status.
- Arrange for a daily review by a nurse or similar healthcare professional, with a view to oral switch when appropriate.
- Weekly multidisciplinary team meetings to review patient's progress and adapt management plan as needed.
- Regular specialist nurse or doctor evaluations, and perform appropriate blood tests at agreed intervals.
- Consider the safety of IV access devices and the storage and safety of any prescribed medications in the home.
- Prepare for possible interventional radiology if abscess formation is confirmed, to perform drainage if indicated.
- Continue psychiatric management for schizophrenia and glaucoma treatment as per standard care.
- Monitor for signs of gastrointestinal bleeding due to a history of a bleeding duodenal ulcer, and manage accordingly.

8. Executive Summary:
A 78-year-old female with a complex medical history including diabetes, HCC, and schizophrenia is currently battling recurrent Escherichia coli bacteremia and possible early abscess formation in HCC lesions. Management includes broad-spectrum IV antibiotics, rigorous monitoring, and multidisciplinary team oversight. The plan also incorporates daily nursing reviews and regular specialist evaluations, with a focus on safety regarding IV access and medication management. The patient's chronic conditions require ongoing treatment and vigilance for complications.

Gemini Pro

1. Chronological Events:

Date	Event
1 November 2023	First admission to Hospital Q for fever.
2 November 2023	Ultrasound (USG) reveals bilobar multifocal HCC.
16 November 2023	Blood culture positive for Escherichia coli.
17 November 2023	Transfer back to Hospital Q for further management.
17 November 2023	Contrast-enhanced computed tomography (CT) abdomen and pelvis shows interval increase in size of hypodense lesion in segment III and a new lobulated hypodense lesion in segment II, suggestive of early abscess formation.

2. Clinical Problem List:

Active Problems	Chronic Problems

|---|---|
Escherichia coli bacteremia	Diabetes mellitus
Hepatitis B virus-related hepatocellular carcinoma	Carcinoma of right breast
Schizophrenia	Glaucoma
Bleeding duodenal ulcer	

3. Potential Life-Threatening Complications:
* Septic shock
* Liver failure
* Hepatic encephalopathy
* Gastrointestinal bleeding
* Perforated duodenal ulcer

4. Clinical Findings:

Organ System	Anticipated Findings
Respiratory	No respiratory distress
Cardiovascular	Dual heart sounds, no murmur
Gastrointestinal	Soft, non-tender abdomen
Neurological	No signs of encephalopathy
Musculoskeletal	No relevant findings

5. Working Diagnoses:
* Escherichia coli bacteremia
* Hepatitis B virus-related hepatocellular carcinoma
* Carcinoma of right breast
* Schizophrenia
* Glaucoma
* Bleeding duodenal ulcer

6. Relevant Investigations:

Investigation	Justification
Complete blood count (CBC)	To assess for anemia, leukocytosis, or thrombocytopenia.
Comprehensive metabolic panel (CMP)	To assess liver and renal function.
Liver function tests (LFTs)	To assess liver function.
Hepatitis B virus (HBV) serology	To confirm hepatitis B infection.
Abdominal ultrasound	To assess for liver cirrhosis and portal hypertension.
Computed tomography (CT) scan of the abdomen and pelvis	To evaluate the extent of hepatocellular carcinoma and to detect any complications.
Esophagogastroduodenoscopy (EGD)	To visualize the duodenal ulcer and assess for bleeding.

7. Management Plan:
* Escherichia coli bacteremia:
* Administer broad-spectrum antibiotics, such as ceftriaxone or piperacillin-tazobactam.
* Monitor for signs of sepsis and septic shock.
* Hepatitis B virus-related hepatocellular carcinoma:
* Continue entecavir therapy.
* Monitor for signs of liver failure and hepatic encephalopathy.
* Consider liver transplantation if the patient meets the criteria.
* Carcinoma of right breast:
* Monitor for signs of recurrence.
* Consider adjuvant therapy, such as radiation therapy or chemotherapy, if indicated.
* Schizophrenia:
* Continue antipsychotic medication.
* Monitor for adverse effects of antipsychotic medication.
* Glaucoma:
* Continue glaucoma medication.
* Monitor for signs of worsening glaucoma.
* Bleeding duodenal ulcer:
* Administer proton pump inhibitors (PPIs) to reduce gastric acid secretion.
* Consider endoscopic therapy, such as endoscopic hemostasis or ulcer repair, if bleeding persists.

8. Executive Summary:
The patient is a 78-year-old female with a history of diabetes mellitus, hepatitis B virus-related hepatocellular carcinoma, carcinoma of the right breast, schizophrenia, glaucoma, and bleeding duodenal ulcer. She was admitted to the hospital with Escherichia coli bacteremia and underwent further evaluation for her hepatocellular carcinoma. The patient is currently afebrile and not septic looking. She has no active complaints. Physical examination findings are unremarkable. The management plan includes broad-spectrum antibiotics for Escherichia coli bacteremia, continued entecavir therapy for hepatitis B virus-related hepatocellular carcinoma, monitoring for signs of liver failure and hepatic encephalopathy, and consideration of liver transplantation if indicated.

Claude 2

1. Chronological Events
Date	Event

Prior to current admission	Diabetes mellitus, Hepatitis B related hepatocellular carcinoma, Right breast carcinoma status post mastectomy with adjuvant Letrozole, Schizophrenia, Glaucoma, History of bleeding duodenal ulcer
1 November 2023	Admitted with fever, Blood culture grew Escherichia coli
2 November 2023	Ultrasound showed bilobar multifocal HCC
17 November 2023	Transferred back to Hospital Q, Contrast CT abdomen showed interval increase in size of segment III lesion concerning for abscess, New segment II lesion
16 November 2023	Blood culture again grew Escherichia coli

2. Clinical Problem List
Active Problems	Chronic Problems
Escherichia coli bacteremia	Diabetes mellitus
Probable liver abscess	Hepatitis B related hepatocellular carcinoma
Progression of hepatocellular carcinoma	Right breast carcinoma status post mastectomy with adjuvant Letrozole
	Schizophrenia
	Glaucoma
	History of bleeding duodenal ulcer

3. Potential Life-Threatening Complications
- Sepsis from Escherichia coli bacteremia
- Rupture of liver abscess resulting in severe abdominal infection
- Progression of hepatocellular carcinoma leading to liver failure

4. Clinical Findings

Organ System	Anticipated Findings
Cardiovascular	Tachycardia from sepsis
Respiratory	Tachypnea from sepsis
Abdominal	Tenderness over liver, possible abdominal distension from liver abscess
Neurological	Confusion from sepsis

5. Working Diagnoses
- Escherichia coli bacteremia
- Probable pyogenic liver abscess
- Progression of hepatocellular carcinoma

6. Relevant Investigations
Investigation	Rationale

Repeat blood cultures	To document clearance of bacteremia
CT abdomen with contrast	To confirm liver abscess and monitor HCC progression
Blood tests: - Complete blood count - Liver function tests - Renal function tests	To assess sepsis severity and impact on end organs

7. Management Plan
- Start empiric intravenous antibiotics to cover enteric gram-negative organisms, guided by culture sensitivities
- Percutaneous drainage of liver abscess by interventional radiology
- Continue HCC surveillance and evaluate treatment options
- Address goals of care given life-limiting HCC
- Monitor for sepsis complications including hypotension, acute kidney injury, DIC
- Correct reversible precipitants including glycemic control in diabetes
- Psychiatric support for schizophrenia
- Ophthalmology review for glaucoma management

8. Executive Summary
This 78-year-old woman with a history of hepatocellular carcinoma presented with Escherichia coli bacteremia and probable pyogenic liver abscess with progression of known HCC. Immediate management should focus on broad-spectrum IV antibiotics, drainage of abscess, and careful monitoring for sepsis complications. However, her underlying advanced HCC needs to be simultaneously addressed with a goals of care discussion.

20. Clinical scenario 20

GPT-4.0

1. Chronological Events
Date	Event
October 2017	Start of HRZE treatment for Right lung abscess with TB complex DNA PCR detected and AFB culture positive.
March 2018	Switch to HR treatment.
July 30, 2018	Completed TB treatment.
October 2023	Increased right upper zone and left middle zone infiltrates on CXR. Bone marrow examination pending.

2. Clinical Problem List
Status	Clinical Issue
Active	Epistaxis with thrombocytopenia
Active	Suspected immune thrombocytopenia
Chronic	Chronic hepatitis B

Chronic	Hypertension
Chronic	Diabetes mellitus A1c 7.1%
Chronic	Asthma
Chronic	Previous right lung abscess (TB)
Chronic	Pneumoconiosis

3. Potential Life-Threatening Complications

- Severe bleeding due to thrombocytopenia which could lead to a hemorrhagic event, especially given the current presentation of epistaxis.
- Recurrence or exacerbation of tuberculosis, especially with abnormal chest X-ray findings.
- Diabetic ketoacidosis or hyperosmolar hyperglycemic state due to diabetes mellitus.
- Asthmatic exacerbation leading to respiratory failure.
- Hepatic decompensation from chronic hepatitis B.

4. Clinical Findings

Organ System	Anticipated Findings
ENT	Epistaxis
Hematologic	Generalized petechiae (low platelet count)
Pulmonary	Fine crepitations over bilateral apical area
Abdominal	Soft non-tender abdomen
Integumentary	Clean drip site

5. Working Diagnoses

- Immune thrombocytopenia
- Tuberculosis (reactivation vs. sequelae of previous infection)
- Pneumoconiosis
- Uncontrolled diabetes mellitus
- Chronic hepatitis B
- Hypertension with possible cardiovascular complications
- Asthma with risk of exacerbation

6. Relevant Investigations

Investigation	Justification
High resolution CT thorax	Assess pulmonary infiltrates and pneumoconiosis
Complete blood count (CBC)	Review platelet count and other hematological parameters
Blood cultures	Rule out underlying infection/bacteremia
Liver function tests (LFTs)	Monitor hepatitis B status and liver function
Hemoglobin A1c	Assess glycemic control
Sputum AFB and TB PCR	Assess for active TB infection

| Bone marrow biopsy| Elucidate cause of thrombocytopenia|

7. Management Plan
- Continue supportive care for epistaxis and monitor hemodynamic status.
- Administer platelet transfusions if necessary to manage thrombocytopenia.
- Initiate or adjust steroid treatment as planned by the haematology team for suspected immune thrombocytopenia.
- Follow-up on bone marrow biopsy results to guide further treatment.
- Tight glycemic control for diabetes mellitus should be ensured.
- Monitor liver function and manage chronic hepatitis B according to current guidelines.
- Administer bronchodilators and ensure asthma control.
- Re-evaluate tuberculosis status with sputum AFB and TB PCR; consider re-initiation of anti-tuberculosis therapy if indicated.
- Perform high resolution CT thorax to evaluate progression of pneumoconiosis and infiltrates.
- Evaluate the need for antihypertensive medication adjustments.
- Consider prophylaxis for stress ulcers due to steroid use and potential for bleeding with thrombocytopenia.

8. Executive Summary
The patient, a 63-year-old male with a history of TB, chronic hepatitis B, hypertension, diabetes, asthma, and pneumoconiosis, presents with active epistaxis and suspected immune thrombocytopenia. Immediate concerns include severe thrombocytopenia potentially causing hemorrhagic complications and the possibility of TB reactivation. Pending investigations include bone marrow biopsy and high resolution CT thorax. The management plan involves supportive care, monitoring, and specific treatments for thrombocytopenia, diabetes, hepatitis B, and asthma, and the potential re-initiation of anti-tuberculosis therapy if indicated.

cGPT-4

1. Chronological Events:

Date	Event
2017	Right lung abscess with TB detected, started HRZE treatment.
March 2018	Switched to HR treatment for TB.
30 July 2018	Completed TB treatment.
Latest CT	Reduction in right upper lobe lesion and cavitation, reduction in right middle lobe consolidation, static nodules and calcified nodes.
Current Admission	Admitted for epistaxis with thrombocytopenia, suspected immune thrombocytopenia. Pending high resolution CT thorax.

2. Clinical Problem List:

Status	Clinical Issues
Active	Epistaxis with thrombocytopenia.
Chronic	Chronic hepatitis B.
Chronic	Hypertension.
Chronic	Diabetes mellitus.
Chronic	Asthma.
Resolved	Right lung abscess (TB).

3. Potential Life-Threatening Complications:
- Severe thrombocytopenia could lead to life-threatening hemorrhages.
- Uncontrolled diabetes may result in diabetic ketoacidosis or hyperosmolar hyperglycemic state.
- Asthma could lead to a severe, potentially fatal asthma attack.
- Reinfection or exacerbation of tuberculosis.

4. Clinical Findings:

Organ System	Anticipated Findings
Respiratory	Fine crepitations over bilateral apical area.
Integumentary	Generalized petechiae.
Hematological	Thrombocytopenia.

5. Working Diagnoses:
- Suspected immune thrombocytopenia.
- Chronic hepatitis B.
- Hypertension.
- Diabetes mellitus.
- Asthma.
- Previous tuberculosis with lung involvement.

6. Relevant Investigations:

Investigation	Justification
Bone marrow examination	To confirm suspected immune thrombocytopenia.
High resolution CT thorax	To assess the state of lung disease and exclude pathology.
Blood glucose monitoring	To manage diabetes mellitus.
Liver function tests	To monitor chronic hepatitis B status.
Sputum AFB culture	To rule out active tuberculosis.

7. Management Plan:

- Continue to assess the patient's thrombocytopenia and manage accordingly, potentially with steroid treatment as planned by the haematology team.
- Monitor and manage blood glucose levels to target diabetes mellitus control.
- Regularly monitor blood pressure and adjust antihypertensive therapy as necessary.
- Maintain asthma treatment regimen and adjust as needed to prevent exacerbations.
- Follow up on high resolution CT thorax to assess for any potential lung pathology that may require intervention.
- Ensure that the patient is educated about the importance of compliance with the management of chronic conditions.

8. Executive Summary:

The patient, a 63-year-old male with a history of tuberculosis treatment, chronic hepatitis B, hypertension, diabetes, and asthma is currently admitted for epistaxis with thrombocytopenia. Management includes steroid treatment for suspected immune thrombocytopenia, monitoring of diabetes and hypertension, and further investigation with a high resolution CT thorax. The patient's chronic conditions require diligent ongoing management to prevent complications.

Gemini Pro

1. Chronological Events:

Date	Event
October 2017	Right lung abscess diagnosed, sputum Mycobacterium tuberculosis (TB) complex DNA polymerase chain reaction (PCR) detected and acid fast bacilli (AFB) culture positive.
October 2017 - March 2018	Started HRZE (isoniazid, rifampicin, pyrazinamide, ethambutol).
March 2018 - July 2018	Switched to HR (isoniazid, rifampicin).
July 2018	Completed TB treatment.
October 2023	Admitted for epistaxis with thrombocytopenia, suspected immune thrombocytopenia, no improvement with intravenous immunoglobulin (IVIG).
October 2023	Chest X-ray (CXR) shows increased right upper zone and left middle zone infiltrates, apical fibrocalcific changes compared to previous CXR.
October 2023	Pending high resolution computed tomography (CT) thorax.

2. Clinical Problem List:

Active	Chronic
Epistaxis	Chronic hepatitis B
Thrombocytopenia	Hypertension
Suspected immune thrombocytopenia	Diabetes mellitus
Increased right upper zone and left middle zone infiltrates	Asthma

Apical fibrocalcific changes	Right lung abscess
Bilateral enlarged, calcified paratracheal nodes	Pneumoconiosis

3. Potential Life-Threatening Complications:
* Pulmonary embolism
* Myocardial infarction
* Stroke
* Sepsis
* Acute respiratory failure
* Renal failure
* Liver failure

4. Clinical Findings:

Organ System	Findings
Respiratory	Fine crepitations over bilateral apical area
Cardiovascular	Blood pressure and pulse unsupported
Integumentary	Generalized petechiae

5. Working Diagnoses:
* Immune thrombocytopenia
* Pulmonary tuberculosis
* Pneumoconiosis
* Hypertension
* Diabetes mellitus

6. Relevant Investigations:

Investigation	Justification
Bone marrow examination	To confirm the diagnosis of immune thrombocytopenia.
High resolution computed tomography (CT) thorax	To assess the extent of the pulmonary infiltrates and to rule out other causes of the infiltrates.
Sputum Mycobacterium tuberculosis (TB) complex DNA polymerase chain reaction (PCR) and acid fast bacilli (AFB) culture	To confirm the diagnosis of pulmonary tuberculosis.
Liver function test	To assess the liver function.
Renal function test	To assess the renal function.

7. Management Plan:
* Transfusion of platelets for thrombocytopenia.
* Steroid treatment for immune thrombocytopenia.
* Anti-tuberculosis treatment for pulmonary tuberculosis.
* Management of hypertension and diabetes mellitus.

* Close monitoring of the patient's condition.

8. Executive Summary:
A 63-year-old male with a history of chronic hepatitis B, hypertension, diabetes mellitus, asthma, and right lung abscess treated for tuberculosis in 2017-2018, presented with epistaxis and thrombocytopenia. Chest X-ray showed increased right upper zone and left middle zone infiltrates. The patient is suspected to have immune thrombocytopenia and pulmonary tuberculosis. Investigations are recommended to confirm the diagnoses. The management plan includes transfusion of platelets, steroid treatment, anti-tuberculosis treatment, and close monitoring.

Claude 2

1. Chronological Events
Date	Event
Past history	Chronic hepatitis B, hypertension, diabetes mellitus, asthma, right lung abscess in 2017 with Mycobacterium tuberculosis infection, completed anti-TB treatment in July 2018
Current admission	Admitted for epistaxis and thrombocytopenia, suspected immune thrombocytopenia

2. Clinical Problem List
Active Problems	Chronic Problems
Epistaxis	Chronic hepatitis B
Thrombocytopenia	Hypertension
Suspected immune thrombocytopenia	Diabetes mellitus
Right upper and left middle zone infiltrates on CXR	Asthma
Increased apical fibrocalcific changes on CXR	Previous right lung abscess and TB in 2017, completed treatment

3. Potential Life-Threatening Complications
- Severe thrombocytopenia can lead to uncontrolled bleeding
- TB reactivation with right lung infiltrates concerning for progression

4. Clinical Findings
Cardiovascular	Unstable vital signs, epistaxis
Respiratory	Fine crepitations over bilateral apical lungs
Abdominal	Soft, non-tender
Neurological	Alert, oriented
Integument	Petechiae, thrombocytopenia

5. Working Diagnoses
- Immune thrombocytopenia
- TB reactivation

6. Relevant Investigations

Investigation	Rationale
Platelet count	Confirm thrombocytopenia, monitor response to treatment
Peripheral blood smear	Evaluate platelet morphology in immune thrombocytopenia
Bone marrow examination	Evaluate megakaryocytes and rule out other causes of thrombocytopenia
High resolution CT chest	Evaluate right lung infiltrates concerning for TB reactivation
Sputum AFB smear and culture	Confirm TB reactivation

7. Management Plan
- Monitor vitals, fluid status, bleeding
- Transfuse platelets for severe thrombocytopenia or bleeding
- Commence steroids for suspected immune thrombocytopenia
- Commence anti-TB treatment if TB reactivation confirmed
- Monitor blood glucose closely with steroids
- Avoid medications that increase bleeding risk
- Address social issues and ensure adherence to treatment

8. Executive Summary
This 63-year-old man with chronic hepatitis B, hypertension, diabetes, asthma and previous TB has presented with epistaxis and thrombocytopenia concerning for immune thrombocytopenia. He requires platelet transfusion, steroids, and close monitoring for complications like bleeding and possible TB reactivation. Investigations including platelet count, bone marrow examination, CT chest and sputum studies should be done. Treatment compliance, glycemic control and social issues need to be addressed.

21. Clinical scenario 21

GPT-4.0

1. Chronological Events

Date	Event
9 Nov 2023	Injury by pet stingray; initial wound infection; administered amoxicillin-clavulanate
13 Nov 2023	Reviewed due to no improvement; added ciprofloxacin

27 Nov 2023	Persistent infection; switched to doxycycline; deep wound swab taken
29 Nov 2023	Started on levofloxacin based on culture results

2. Clinical Problem List

Status	Clinical Issues
Active	Wound infection with necrosis and purulent discharge
Active	Cellulitis up to the elbow
Chronic	Schizophrenia
Chronic	Injury from pet stingray

3. Potential Life-Threatening Complications
- Sepsis from the wound infection
- Necrotizing fasciitis due to the presence of necrotic tissue
- Spread of cellulitis leading to lymphangitis or septicemia

4. Clinical Findings

Organ System	Anticipated Findings
Integumentary	2x4cm wound over right hand dorsum; necrotic tissue; purulent discharges; erythema up to elbow
Musculoskeletal	Exposure of underlying muscles and tendons in the wound
Neurological	Potential altered sensation around wound site due to injury or infection

5. Working Diagnoses
- Complicated skin and soft tissue infection (cSSTI), possibly necrotizing, due to stingray injury
- Ongoing cellulitis with potential for systemic spread

6. Relevant Investigations

Investigation	Justification
Repeat deep wound swab	To check for current microbial flora and antibiotic sensitivities
CRP and ESR	To monitor inflammatory markers and response to treatment
Blood cultures	To rule out bacteremia or sepsis
Hand X-ray	To exclude osteomyelitis
MRI of the hand	To assess the extent of soft tissue involvement

7. Management Plan
- Continue levofloxacin as per sensitivity.

- Surgical debridement of necrotic tissue to reduce the load of infection and remove the source of toxins.
- Supportive care including analgesia, wound care, and tetanus prophylaxis.
- Monitor for signs of systemic infection, such as fever or increased white blood cell count.
- Psychiatric liaison for schizophrenia management and adherence to psychiatric medications during hospital stay.
- Consideration for occupational exposure and pet-related injury prevention education.

8. Executive Summary

A 51-year-old male with schizophrenia on olanzapine and citalopram presented with a worsening stingray-induced wound infection, which has not responded to multiple antibiotics. The wound exhibits necrosis, purulent discharge, and cellulitis extending to the elbow. Management has included antibiotic therapy tailored to culture results and will require surgical debridement for source control. Monitoring for systemic infection and continued psychiatric care are necessary. The comprehensive management plan centers on both infection resolution and prevention of complications, including sepsis and necrotizing fasciitis.

cGPT-4

1. Chronological Events:

Date	Event
9 Nov 2023	Stingray injury over right hand dorsum
9 Nov 2023	Initial treatment with amoxicillin-clavulanate
13 Nov 2023	Reviewed, no improvement; ciprofloxacin added
27 Nov 2023	Persistent infection; switched to doxycycline
27 Nov 2023	Deep wound swab taken
29 Nov 2023	Started on levofloxacin

2. Clinical Problem List:

Status	Clinical Issues
Active	Worsening wound infection
Active	Cellulitic changes up to elbow
Chronic	Schizophrenia
Chronic	Under treatment with olanzapine
Chronic	Under treatment with citalopram

3. Potential Life-Threatening Complications:
- Septicemia or systemic infection due to persistent wound infection and cellulitis.
- Compartment syndrome from spreading cellulitis.

- Necrotizing fasciitis considering exposure to marine bacteria.

4. Clinical Findings:

Organ System	Findings
Skin/Soft Tissue	Wound over right hand dorsum, necrotic tissue, purulent discharge
Musculoskeletal	Underlying muscles and tendons visible
Integumentary	Cellulitic changes with erythema

5. Working Diagnoses:
- Worsening wound infection, possibly complicated by marine bacteria.
- Cellulitis extending to the elbow.

6. Relevant Investigations:

Investigation	Justification
Repeat deep wound swab	To reassess for presence of organisms and antibiotic susceptibility
Blood cultures	To rule out bacteremia or septicemia
CRP and ESR	To monitor inflammation status and response to treatment
Ultrasound of hand	To evaluate for abscess or deeper spread of infection
MRI of hand and forearm	To rule out necrotizing fasciitis or osteomyelitis

7. Management Plan:
- Continue levofloxacin as MSSA and Corynebacterium striatum are sensitive.
- Surgical consultation for possible debridement of necrotic tissue and exploration of the extent of infection.
- Monitor for signs of systemic infection; if present, consider broadening antimicrobial coverage.
- Frequent review by a nurse or similar healthcare professional to assess the progress of infection and possibility of oral switch.
- Weekly multidisciplinary team meeting to evaluate patient status and adjust treatment as necessary.
- Consideration of patient's home environment for safety and suitability for continued outpatient parenteral antimicrobial therapy (OPAT).
- Prospective data collection for audit purposes to detect issues related to the OPAT program.

8. Executive Summary:
The patient has a serious wound infection from a stingray injury, which has worsened despite multiple antibiotics. Current treatment with levofloxacin is appropriate given the sensitivity of isolated organisms. A surgical consultation is needed for possible debridement, and close monitoring is crucial to manage and prevent complications. An

OPAT program may be considered, ensuring the patient's home environment is conducive to such a plan.

Gemini Pro

1. Chronological Events:
Date	Event
9 November 2023	Injured by pet stingray over right hand dorsum.
Same day	Attended Hospital Q accident and emergency department, given amoxicillin-clavulanate.
13 November 2023	Reviewed, added ciprofloxacin due to no improvement in wound.
27 November 2023	Persistent wound infection noted, given doxycycline alone.
27 November 2023	Deep wound swab taken, gram stain no organism seen, WBC negative, culture Corynebacterium striatum, MSSA (sensitive to levofloxacin).
29 November 2023	Levofloxacin started.

2. Clinical Problem List:
Active	Chronic
Wound infection	Schizophrenia
Cellulitis	
Necrotizing fasciitis	
Septic arthritis	
Osteomyelitis	

3. Potential Life-Threatening Complications:
* Sepsis
* Amputation
* Death

4. Clinical Findings:
Organ System	Findings
Integumentary	2x4cm wound over right hand dorsum with underlying muscles and tendons seen, necrotic tissue, purulent discharges, cellulitic changes noted with erythema up to elbow.
Musculoskeletal	Possible osteomyelitis changes.
Respiratory	Stable, afebrile, on room air.
Cardiovascular	Stable vitals.
Neurological	No neurological findings reported.
Gastrointestinal	No gastrointestinal findings reported.

| Genitourinary | No genitourinary findings reported. |
| Hematologic | Normal ANC. |

5. Working Diagnoses:
* Necrotizing fasciitis
* Septic arthritis
* Osteomyelitis

6. Relevant Investigations:
Investigation	Justification
Blood cultures	To identify and characterize any bloodstream infection.
Wound culture	To identify and characterize the causative organism(s) of the wound infection.
X-ray of the right hand	To assess for osteomyelitis.
MRI of the right hand	To further evaluate for osteomyelitis and septic arthritis.
Complete blood count (CBC) with differential	To assess for leukocytosis and neutrophilia.
Erythrocyte sedimentation rate (ESR)	To assess for inflammation.
C-reactive protein (CRP)	To assess for inflammation.

7. Management Plan:
* Administer broad-spectrum antibiotics (e.g., vancomycin and piperacillin-tazobactam) to cover potential pathogens.
* Debride the wound surgically to remove necrotic tissue and promote healing.
* Immobilize the affected hand to prevent further injury.
* Monitor the patient for signs of sepsis and other complications.
* Provide supportive care, including pain management and fluid resuscitation.
* Consider hyperbaric oxygen therapy as an adjunctive therapy.

8. Executive Summary:
The patient is a 51-year-old male with a history of schizophrenia who presented with a worsening wound infection of the right hand dorsum. He has been treated with multiple antibiotics without improvement. The patient is currently afebrile and hemodynamically stable. Physical examination revealed a 2x4cm wound with underlying muscles and tendons seen, necrotic tissue, purulent discharges, and cellulitic changes. Laboratory findings showed normal ANC and elevated ESR. The patient is at risk for life-threatening complications such as sepsis, amputation, and death. A comprehensive management plan has been developed, including broad-spectrum antibiotics, surgical debridement, immobilization, and supportive care.

Claude 2

1. Chronological Events

Date	Event
9 November 2023	Injured by pet stingray over right hand dorsum. Attended Hospital Q accident and emergency department, given amoxicillin-clavulanate.
13 November 2023	Reviewed wound, noted no improvement. Added ciprofloxacin.
27 November 2023	Persistent wound infection noted during review. Given doxycycline alone.
29 November 2023	Admitted for worsening wound infection. Started on levofloxacin.

2. Clinical Problem List

Problem	Status
Wound infection of right hand	Active
Schizophrenia	Chronic

3. Potential Life-Threatening Complications
- Sepsis/septic shock from uncontrolled wound infection
- Osteomyelitis of hand bones leading to loss of function
- Spread of infection to vital organs through bloodstream

4. Anticipated Physical Examination Findings

Organ System	Expected Findings
Hand wound	2x4cm wound over dorsum with visible muscles/tendons, necrotic tissue, purulent discharge, surrounding cellulitis/erythema
Vitals	Stable, afebrile
Systemic	No signs of sepsis or metastatic infection

5. Working Diagnoses
- Infected wound of right hand
- Cellulitis
- Possible osteomyelitis

6. Relevant Investigations

Investigation	Justification
Wound culture	Identify causative organisms and antibiotic sensitivities
Blood cultures	Check for bacteremia/sepsis
ESR, CRP	Assess inflammation/infection
X-ray of hand	Evaluate for osteomyelitis
CBC, renal function	Assess response to infection

7. Management Plan
- Urgent washout and debridement of infected hand wound in operating room
- Obtain wound and blood cultures
- Start broad-spectrum IV antibiotics (vancomycin, piperacillin/tazobactam)
- Monitor for sepsis and metastatic infection
- Hand surgery consult for possible reconstruction
- Infectious disease consult for antibiotic management
- Physical/occupational therapy for hand rehabilitation

8. Executive Summary
51yo male with infected stingray wound on right hand, failed oral antibiotics. At risk for osteomyelitis, sepsis. Requires urgent washout/debridement, IV antibiotics, and monitoring for severe infection. Hand surgery and ID consults recommended to optimize functional recovery.

22. Clinical scenario 22

GPT-4.0

1. Chronological Events

Date	Event
July 2023	Initial CT abdomen/pelvis showed multiorgan involvement.
August 2023	Started SIMPLE (50%) chemotherapy.
2 October 2023	Admitted for second cycle of SIMPLE (50%) chemotherapy.
12 October 2023	Second cycle of SIMPLE (50%) started.
2 November 2023	PET-CT scan showed diffuse swelling and hypermetabolism of the pancreas.
4 November 2023	First contrast-enhanced CT indicated a pseudoaneurysm in the gastroduodenal artery.
5 November 2023	Second CT showed enlargement of the pseudoaneurysm. First coil embolisation performed.
7 November 2023	Second embolisation performed on inferior pancreaticoduodenal artery branches.
11 November 2023	Third CT showed further enlargement of the pseudoaneurysm and increased pancreatic swelling.
13 November 2023	Third embolisation on left gastroepiploic artery due to risk of non-target embolisation.
15 November 2023	Patient began experiencing swinging fever.
16 November 2023	Right external PTBD insertion.
17 November 2023	IV meropenem started.
19 November 2023	IV vancomycin started.
20 November 2023	IV amikacin started.

23 November 2023	Pending PET-CT scan.

2. Clinical Problem List

Status	Clinical Issue
Active	Allergic to IV contrast and gelofusin
Active	Acute pancreatitis with elevated amylase and lipase
Active	Stage IV NK/T-cell lymphoma
Active	Pseudoaneurysm of the gastroduodenal artery
Active	Swinging fever since 15 November 2023
Chronic	History of acute pancreatitis (2022, alcoholism)
Chronic	Ex-smoker and ex-drinker

3. Potential Life-Threatening Complications
- Hemorrhage from the enlarging pseudoaneurysm in the pancreatic head.
- Biliary obstruction could lead to cholangitis or liver failure.
- Sepsis from potential infectious complications.
- Complications from lymphoma, including metabolic disturbances.

4. Clinical Findings

Organ System	Anticipated Findings
General	Fever (swinging fever since 15 November 2023)
Abdominal	Soft, non-tender; possible swelling if there is an abscess or severe pancreatitis
Vascular	Previous vascular access wounds healed, non-tender on palpation
Cardiac	Heart sounds dual, no murmur

5. Working Diagnoses
- NK/T-cell lymphoma, Stage IV
- Pseudoaneurysm of the gastroduodenal artery
- Acute pancreatitis with possible secondary infection
- Fever of unknown origin (potentially related to lymphoma or post-embolisation syndrome)

6. Relevant Investigations

Investigation	Justification
PET-CT scan	To evaluate the status of lymphoma and check for other potential sources of fever
Serial contrast-enhanced CT abdomen and pelvis	To monitor the pseudoaneurysm and the pancreatic inflammation

Blood cultures	To identify any potential bacterial pathogens responsible for the fever
Pancreatic enzymes	To monitor the progression of pancreatitis
Liver function tests	To assess liver involvement and biliary obstruction

7. Management Plan
- Continue monitoring and management of NK/T-cell lymphoma with chemotherapy as tolerated.
- Close observation of the pseudoaneurysm with possible further intervention if it continues to expand or if there is evidence of hemorrhage.
- Manage signs of pancreatitis and prevent/manage complications such as necrosis or infection.
- Antimicrobial therapy should be guided by cultures and sensitivity, with possible de-escalation once pathogens are identified.
- Supportive care for symptoms such as fever and pain.
- Nutritional support considering the patient's pancreatic condition.
- Consider consultation with interventional radiology and possibly vascular surgery for the management of the pseudoaneurysm.
- Regular monitoring with imaging and blood tests to track the progress of the disease and response to treatment.

8. Executive Summary
The patient is a 50-year-old male with a history of acute pancreatitis and Stage IV NK/T-cell lymphoma, currently experiencing complications from a pseudoaneurysm of the gastroduodenal artery and swinging fevers post-chemotherapy. The management plan includes continuation of chemotherapy, vigilant monitoring and potential further embolisation of the pseudoaneurysm, aggressive management of pancreatitis, and targeted antimicrobial therapy. The patient’s condition necessitates a multidisciplinary approach, including oncology, gastroenterology, interventional radiology, and potentially vascular surgery, to address the complex interplay of his clinical issues and to prevent life-threatening complications.

cGPT-4

1. Chronological Events:
Date	Major Clinical Issue
2022	Acute pancreatitis due to alcoholism
July 2023	Initial CT showing multiorgan involvement
August 2023	Start of SIMPLE chemotherapy 50%
2 October 2023	Clinical admission for SIMPLE (II) 50%
2 November 2023	PET-CT scan showing diffuse swelling and hypermetabolism of pancreas

4 November 2023	1st detection of pseudoaneurysm in pancreatic head
5 November 2023	1st Coil embolisation of GDA pseudoaneurysm; Enlargement of pseudoaneurysm
7 November 2023	2nd Embolisation of inferior pancreaticoduodenal artery branches
11 November 2023	Interval enlargement of known pseudoaneurysm; Increased fat stranding
13 November 2023	3rd Left gastroepiploic artery embolisation
16 November 2023	Right external PTBD insertion
15 November 2023 - Present	Swinging fever

2. Clinical Problem List:

Status	Clinical Issue
Active	Allergy to IV contrast, gelofusin
Active	Swinging fever
Active	Pseudoaneurysm in pancreatic head
Active	Elevated amylase and lipase levels
Chronic	Stage IV NK/T-cell lymphoma
Chronic	History of acute pancreatitis
Chronic	Ex-smoker & ex-drinker

3. Potential Life-Threatening Complications:
- Rupture of the enlarging pseudoaneurysm leading to hemorrhage.
- Development of sepsis from persistent fever possibly indicating an ongoing infection.
- Progression of Stage IV NK/T-cell lymphoma.

4. Clinical Findings:

Organ System	Anticipated Findings
General	Swinging fever
Abdominal	Soft non-tender abdomen; Pancreatic pseudoaneurysm
Cardiovascular	Dual heart sounds; No murmur
Skin	Healed vascular access wounds; Non-tender on palpation

5. Working Diagnoses:
- NK/T-cell lymphoma with multiorgan involvement.
- Pancreatic pseudoaneurysm with recurrent enlargement post-embolisation.
- Post-chemotherapy febrile episodes possibly indicative of infection.

6. Relevant Investigations:

Investigation	Justification

PET-CT scan	To evaluate the progression of lymphoma and monitor response to therapy
Contrast-enhanced CT abdomen and pelvis	To monitor the pseudoaneurysm size and complications
Blood cultures	To identify potential causative agents of fever and guide antimicrobial therapy
Amylase and lipase levels	To assess for pancreatic inflammation or damage

7. Management Plan:
- Continue surveillance of the pseudoaneurysm and consider surgical intervention if it continues to enlarge or if there is evidence of impending rupture.
- Maintain current antimicrobial therapy and reassess based on culture results and clinical response.
- Supportive care for symptoms and management of side effects of chemotherapy.
- Close monitoring for signs of lymphoma progression or complications.
- Regular follow-up with oncology for the management of NK/T-cell lymphoma.

8. Executive Summary:
The patient is a 50-year-old male with a history of acute pancreatitis and Stage IV NK/T-cell lymphoma currently experiencing complications from a pancreatic pseudoaneurysm and swinging fevers post-chemotherapy. The management plan includes close monitoring of the pseudoaneurysm, continuation of broad-spectrum antimicrobials, and supportive care, with ongoing oncology follow-up to manage his lymphoma. Immediate life-threatening complications include rupture of the pseudoaneurysm and sepsis.

Gemini Pro

1. Chronological Events:
Date	Event
July 2023	Contrast-enhanced computed tomography (CT) abdomen and pelvis showed multiorgan involvement, including pancreas, lymph nodes, kidneys, lungs, and skin.
August 2023	Started SIMPLE (dexamethasone, methotrexate, ifosfamide, L-asparaginase, etoposide) (50%).
2 October 2023	Clinically admitted for SIMPLE (II) 50% (started 12 October 2023).
2 November 2023	Positron Emission Tomography-Computed Tomography (PET-CT) scan showed diffuse swelling of the pancreas with heterogeneous hypermetabolism and mild peripancreatic strandings.

4 November 2023	Contrast-enhanced CT abdomen and pelvis showed a 2.8 x 4.0 x 2.8cm pseudoaneurysm arising from the gastroduodenal artery (GDA) in the pancreatic head.
5 November 2023	First coil embolization of GDA pseudoaneurysm.
5 November 2023	Contrast-enhanced CT abdomen and pelvis showed enlarging pseudoaneurysm, measuring 3.9 x 5.7 x 4.8cm (WxAPxCC).
7 November 2023	Second embolization of inferior pancreaticoduodenal artery branches.
11 November 2023	Contrast-enhanced CT abdomen and pelvis showed interval enlargement of the known pseudoaneurysm in the pancreatic head, now measuring up to 6.5x7.7x4.8cm (TSxAPxCC).
13 November 2023	Third left gastroepiploic artery embolization.
15 November 2023	Swinging fever.
16 November 2023	Right external PTBD insertion.
17 November 2023	Started IV meropenem.
19 November 2023	Started IV vancomycin.
20 November 2023	Started IV amikacin.
23 November 2023	Pending PET-CT scan.

2. Clinical Problem List:

Active	Chronic
Pancreatic pseudoaneurysm	Stage IV NK/T-cell lymphoma
Abdominal pain	Acute pancreatitis (2022)
Rising amylase and lipase	Ex-smoker
Fever	Ex-drinker
	Allergic to IV contrast, gelofusin

3. Potential Life-Threatening Complications:
* Pancreatic pseudoaneurysm rupture
* Pancreatitis
* Sepsis
* Multiorgan failure

4. Clinical Findings:

Organ System	Anticipated Physical Examination Findings
General	Post-chemotherapy appearance, swinging fever
Respiratory	No abnormal findings
Cardiovascular	Heart sounds dual, no murmur
Gastrointestinal	Abdomen soft non-tender, previous vascular access wounds healed, non-tender on palpation
Neurological	No abnormal findings

| Musculoskeletal | No abnormal findings |
| Integumentary | No abnormal findings |

5. Working Diagnoses:
* Pancreatic pseudoaneurysm
* Pancreatitis
* Sepsis
* Stage IV NK/T-cell lymphoma

6. Relevant Investigations:

Investigation	Justification
Complete blood count	To assess for anemia, leukocytosis, or thrombocytopenia.
Comprehensive metabolic panel	To assess for electrolyte imbalances, renal function, and liver function.
Blood cultures	To identify the causative organism of sepsis.
Abdominal ultrasound	To visualize the pancreatic pseudoaneurysm and assess for complications.
Contrast-enhanced CT abdomen and pelvis	To further evaluate the pancreatic pseudoaneurysm and surrounding structures.
PET-CT scan	To assess the extent of NK/T-cell lymphoma and response to treatment.

7. Management Plan:
* Continue broad-spectrum antibiotics to cover potential pathogens.
* Monitor vital signs and clinical status closely.
* Consult vascular surgery for possible surgical intervention of the pancreatic pseudoaneurysm.
* Continue chemotherapy for NK/T-cell lymphoma as tolerated.
* Provide supportive care, including pain management and nutritional support.

8. Executive Summary:
The patient is a 50-year-old male with Stage IV NK/T-cell lymphoma who is currently experiencing a pancreatic pseudoaneurysm, pancreatitis, and sepsis. He has a history of acute pancreatitis and is an ex-smoker and ex-drinker. The patient is currently receiving broad-spectrum antibiotics and chemotherapy. Investigations are recommended to further evaluate the extent of the disease and complications. A multidisciplinary approach involving medical oncology, vascular surgery, and infectious diseases is necessary for optimal management.

Claude 2

1. Chronological Events

Date	Event
July 2023	Contrast-enhanced CT abdomen and pelvis: multiorgan involvement, including pancreas, lymph nodes, kidneys, lungs, and skin. Percutaneous transhepatic biliary drainage (PTBD) inserted.
August 2023	Started SIMPLE chemotherapy
2 October 2023	Admission for SIMPLE chemotherapy cycle 2
12 October 2023	Started SIMPLE chemotherapy cycle 2
2 November 2023	PET-CT scan: diffuse pancreatic head swelling and hypermetabolism. Mild peripancreatic fat stranding.
4 November 2023	Contrast-enhanced CT abdomen: 2.8 x 4.0 x 2.8 cm pseudoaneurysm arising from gastroduodenal artery (GDA) in pancreatic head.
5 November 2023	Contrast-enhanced CT abdomen: Enlarging GDA pseudoaneurysm, now 3.9 x 5.7 x 4.8 cm. 1st coil embolization of GDA pseudoaneurysm.
7 November 2023	2nd embolization of inferior pancreaticoduodenal artery branches.
11 November 2023	Contrast-enhanced CT abdomen: Interval enlargement of pancreatic head pseudoaneurysm, now 6.5 x 7.7 x 4.8 cm. Worsening pancreatic and biliary ductal dilatation.
13 November 2023	3rd embolization of left gastroepiploic artery.
16 November 2023	Right external PTBD insertion.
15 November 2023	Onset of swinging fevers.

2. Clinical Problem List

Problem	Active/Chronic
Stage IV NK/T-cell lymphoma	Chronic
Malignant biliary obstruction	Chronic
Deranged liver function	Chronic
Pancreatic head pseudoaneurysm	Active
Worsening pancreatic ductal dilatation	Active
Worsening biliary ductal dilatation	Active
Swinging fevers	Active

3. Potential Life-Threatening Complications
- Rupture of enlarging pancreatic head pseudoaneurysm causing torrential bleeding
- Worsening biliary obstruction leading to recurrent cholangitis/cholecystitis
- Progression of lymphoma with multiorgan failure

4. Anticipated Physical Examination Findings

Organ System	Expected Findings

General	Post-chemotherapy appearance, no acute distress
HEENT	No focal neurological deficits, no lymphadenopathy
Cardiovascular	Tachycardia, no murmurs
Pulmonary	Clear on auscultation
Abdominal	Soft, non-tender, no peritonitis
Extremities	No edema

5. Working Diagnoses
- Infectious etiology such as cholangitis or line sepsis causing swinging fevers
- Progression of lymphoma with development of fever
- Mechanical complication such as stent occlusion leading to fevers

6. Relevant Investigations

Investigation	Justification
Blood cultures	To identify any bacteremia or line sepsis
Imaging (MRI or CT) of abdomen	To assess lymphoma burden and re-evaluate pseudoaneurysm, biliary stent patency
LFTs, lipase/amylase	Evaluate for worsening biliary/pancreatic obstruction
CRP, procalcitonin	Markers of inflammation/infection
PET-CT scan	Assessment of lymphoma response to chemotherapy

7. Management Plan
- Continue broad-spectrum IV antibiotics to cover enteric GNB and maintain source control
- Interventional radiology consult for potential pseudoaneurysm coiling/stenting if enlarging
- Surgical evaluation for possible debulking of pancreatic head mass
- Monitor for signs of bleeding, worsening jaundice, cholangitis - prepare for IR or surgical intervention
- If fevers persist, investigate other sources (e.g. line cultures, remove if infected)
- Continue scheduled chemotherapy once infection cleared
- Provide supportive care, nutritional support, pain control as needed

8. Executive Summary
This 50-year-old male with stage IV NK/T cell lymphoma has an enlarging pancreatic head pseudoaneurysm and worsening biliary/pancreatic ductal dilatation causing swinging fevers after chemotherapy. Immediate management should focus on continued broad-spectrum IV antibiotics, close monitoring for bleeding/jaundice, IR or surgical intervention if pseudoaneurysm enlarges further, and infection source control. The fevers are likely infectious but lymphoma progression must be evaluated. Once the infection is treated, chemotherapy should be resumed.

23. Clinical scenario 23

GPT-4.0

1. Chronological Events:

Date	Event
Early 2013	Patient had Intra-uterine Contraceptive Device (IUCD) insertion done.
4-6 Oct 2023	Travelled to Zhanjiang, China. Consumed cooked seafood, chicken, and eggs.
7-10 Oct 2023	Developed diarrhea, resolved with symptomatic medications from a private doctor.
18 Oct 2023	Presented with dry mouth and tiredness, given symptomatic medications and antibiotics.
24 Oct 2023	Attended A&E with worsening symptoms, took traditional Chinese medications.
28 Oct 2023	Admitted with deranged liver function and fever. Underwent various investigations and started on antimicrobials.

2. Clinical Problem List:

Status	Clinical Issues
Active	Deranged liver function
Active	Fever
Active	Diarrhea
Active	Swinging fever
Chronic	IUCD insertion (10 years ago)

3. Potential Life-Threatening Complications:
- Hepatic failure or severe hepatitis leading to coagulopathy and encephalopathy.
- Sepsis or septic shock, especially considering the swinging fever and elevated CRP.
- Complications from potential splenic involvement, such as splenic rupture or infarct.

4. Clinical Findings:

Organ System	Expected Findings
General	Swinging fever up to 39.5°C
Cardiovascular	Blood pressure and pulse unsupported and on room air
Respiratory	Chest clear
Gastrointestinal	Faint spots of erythema over abdomen, possible splenic mass, no RIF tenderness
Musculoskeletal	Calves are soft, no lower limb edema
Integumentary	No notable findings listed

5. Working Diagnoses:
- Hepatitis (viral or drug-induced)

- Bacterial gastroenteritis
- Sepsis of unknown origin
- Complication of IUCD (less likely given the chronicity)

6. Relevant Investigations:

Investigation	Justification
Blood culture	To identify causative organism for sepsis
Stool culture	To identify causative organism for diarrhea
Sputum culture	To exclude respiratory infection
Urine culture	To exclude urinary tract infection
Liver function test	To assess the extent of liver derangement
Ultrasound abdomen	To evaluate palpable mass and check IUCD position
Serology for hepatitis	To identify potential viral etiology for hepatitis

7. Management Plan:
- Continue broad-spectrum antibiotics (Amoxicillin-clavulanate and Doxycycline) until cultures are obtained and reviewed.
- Ensure adequate hydration and electrolyte balance, considering the patient's history of diarrhea.
- Monitor liver function tests closely to assess the progression of liver involvement.
- Evaluate the need for imaging studies such as an ultrasound or CT scan to explore the left upper quadrant mass and the status of the spleen.
- Consider a gastroenterology and infectious disease consultation for further management based on the cultures and serology results.

8. Executive Summary:
The patient, a 51-year-old female with a history of IUCD insertion and recent travel to China, presents with fever, deranged liver function, and diarrhea. Immediate concerns include potential hepatitis, bacterial gastroenteritis, or sepsis. The management plan involves broad-spectrum antibiotics, close monitoring, and targeted investigations. The priority is to stabilize the patient, identify the causative pathogen, and address any complications promptly. The patient's chronic IUCD use is noted but is not the primary focus of current treatment.

cGPT-4

1. Chronological Events:

Date	Event
4-6 Oct	Travel to Zhanjiang, China; consumed cooked seafood
7-10 Oct	Diarrhea for 3-4 days, resolved with medications from doctor
18 Oct	Dry mouth, tiredness, administered symptomatic medications

20 Oct	Diarrhea resumed, pale yellow urine
24 Oct	A&E visit, given symptomatic medications
28 Oct	Admission with deranged liver function, fever, and chest X-ray showing a left pleural effusion.

2. Clinical Problem List:

Status	Clinical Issue
Active	Deranged liver function
Active	Fever
Active	Diarrhea
Chronic	IUCD insertion (10 years ago)

3. Potential Life-Threatening Complications:
- Sepsis or severe sepsis due to possible systemic infection.
- Acute liver failure related to deranged liver function.
- Severe dehydration secondary to persistent diarrhea.

4. Clinical Findings:

Organ System	Anticipated Findings
General	Swinging fever, signs of dehydration (dry mouth)
Abdomen	Erythema, possible spleen tip palpation
Musculoskeletal	Absence of lower limb edema
Skin	Faint spots of erythema over abdomen

5. Working Diagnoses:
- Infectious hepatitis
- Sepsis secondary to a potential bacterial infection
- Gastroenteritis

6. Relevant Investigations:

Investigation	Justification
Blood culture	To identify causative organism for sepsis
Stool culture	To identify pathogen causing diarrhea
Sputum culture	To investigate respiratory infection due to pleural effusion
Urine culture	To exclude urinary tract infection
Liver function tests	To assess the extent of liver damage
CRP	To gauge the inflammatory response

7. Management Plan:
- Continue current antimicrobials, and adjust based on culture results.

- Monitor vital signs, blood pressure, and pulse closely.
- Ensure adequate hydration and nutrition.
- Regularly assess the need for increased ward cleaning and maintenance of equipment.
- Review antibiotic prescribing and adjust based on culture results and sensitivity.
- Daily review by a nurse or similar healthcare professional with expertise in managing infectious diseases.
- Weekly multidisciplinary team meeting to review patient's progress.
- Collection of data for audit and prospective review.

8. Executive Summary:
The patient is a 51-year-old female presenting with deranged liver function and fever post dietary exposure in China. She has been started on amoxicillin-clavulanate and doxycycline. Investigations such as blood, stool, sputum, and urine cultures are pending. The management plan involves continuing antimicrobial therapy, close monitoring, and regular reviews. Adjustments to treatment will be based on lab results and multidisciplinary team assessments.

Gemini Pro

1. Chronological Events
Date	Event
4-6 October 2023	Travelled to Zhanjiang, China and consumed cooked seafood, chicken, eggs, etc.
October 2023	Developed 3-4 days of diarrhea, resolved with symptomatic medications from private doctor.
18 October 2023	Presented with dry mouth and tiredness, given symptomatic medications and 2 days of antibiotics by private.
24 October 2023	Attended accident and emergency department with symptomatic medications again.
28 October 2023	Admitted for deranged liver function and fever.

2. Clinical Problem List
Active Problems	Chronic Problems
Fever	Intra-uterine Contraceptive Device (IUCD) insertion 10 years ago
Diarrhea	
Deranged liver function	
Left pleural effusion	

3. Potential Life-Threatening Complications
| Clinical Problem | Potential Life-Threatening Complications |

Fever	Sepsis, multi-organ failure
Diarrhea	Dehydration, electrolyte imbalance
Deranged liver function	Liver failure
Left pleural effusion	Respiratory failure

4. Clinical Findings

Organ System	Anticipated Physical Examination Findings
Respiratory	Chest clear, swinging fever up to 39.5'C
Cardiovascular	Blood pressure and pulse are unsupported and on room air
Gastrointestinal	Faint spots of erythema over abdomen, query palpable left upper zone mass, query Spleen tip, no right upper quadrant tenderness
Musculoskeletal	Calves are soft
Integumentary	No lower limb edema

5. Working Diagnoses

Probable Diagnoses
Infectious diarrhea
Enteric fever
Leptospirosis
Dengue fever
Malaria

6. Relevant Investigations

Investigation	Justification
Blood culture	To identify the causative organism of fever and deranged liver function
Stool culture	To identify the causative organism of diarrhea
Sputum culture	To identify the causative organism of respiratory symptoms
Urine culture	To identify the causative organism of urinary symptoms
Serology for enteric fever, leptospirosis, dengue fever, and malaria	To confirm the diagnosis of suspected infections
Liver function tests	To assess the extent of liver damage
Renal function tests	To assess renal function
Chest X-ray	To assess the extent of pleural effusion

7. Management Plan

Intervention	Goal
Administer broad-spectrum antibiotics	To treat suspected infections

Provide supportive care	To prevent and manage complications
Monitor vital signs and oxygen saturation	To detect early signs of deterioration
Administer antipyretics	To reduce fever
Administer antidiarrheal agents	To reduce diarrhea
Administer fluids and electrolytes	To correct dehydration and electrolyte imbalance
Monitor liver function tests and renal function tests	To assess response to treatment and detect early signs of complications
Perform serial chest X-rays	To monitor the resolution of pleural effusion

8. Executive Summary
The patient is a 51-year-old female with fever, diarrhea, deranged liver function, and left pleural effusion. She has a history of travel to Zhanjiang, China, where she consumed cooked seafood, chicken, and eggs. The patient is at risk of developing life-threatening complications, including sepsis, multi-organ failure, dehydration, electrolyte imbalance, liver failure, and respiratory failure. A comprehensive management plan has been initiated, including broad-spectrum antibiotics, supportive care, and monitoring. Further investigations are needed to confirm the diagnosis and assess the extent of organ damage.

Claude 2

1. Chronological Events
Date	Event
4-6 October 2023	Travelled to Zhanjiang, China and ate cooked seafood
7-10 October 2023	Developed diarrhea, treated symptomatically
18 October 2023	Presented with dry mouth and tiredness, given antibiotics
24 October 2023	Attended A&E with yellowish diarrhea, pale urine
28 October 2023	Admitted with deranged LFTs and fever

2. Clinical Problem List
Problem	Active/Chronic
Fever	Active
Deranged LFTs	Active
Diarrhea	Active
Left pleural effusion	Active
IUCD in situ	Chronic

3. Potential Life-Threatening Complications
- Sepsis from undiagnosed infection
- Hepatic failure from severe hepatitis
- Pleural effusion causing respiratory compromise

4. Clinical Findings

System	Expected Findings
General	Fever, tachycardia
Respiratory	Left pleural effusion on CXR
Cardiovascular	Tachycardia, hypotension
Abdominal	Hepatomegaly, splenomegaly
Neurological	Normal

5. Working Diagnoses
- Infectious:
- Bacterial gastroenteritis
- Typhoid fever
- Hepatitis A
- Hepatitis E
- Non-infectious:
- Drug-induced liver injury
- Autoimmune hepatitis

6. Relevant Investigations

Investigation	Rationale
Blood culture	Diagnose bacteremia
Stool culture	Diagnose bacterial gastroenteritis
Serology for Hepatitis A, E	Diagnose acute hepatitis
ANA, ASMA, IgG	Assess for autoimmune hepatitis
Ultrasound abdomen	Assess for hepatosplenomegaly

7. Management Plan
- Monitor vitals and fluid status closely
- Provide supportive care - IV fluids, antipyretics
- Empiric antibiotics for suspected infection
- Avoid hepatotoxic drugs
- Consider transfer to higher level of care if clinical deterioration
- Plan for liver biopsy and specialist input if autoimmune/chronic hepatitis suspected

8. Executive Summary

This 51F presented with fever, deranged LFTs and diarrhea after recent travel to China. Workup should focus on infectious hepatitis and bacterial gastroenteritis while also considering drug toxicity and autoimmune etiologies. She is at risk for sepsis and liver failure thus close monitoring is warranted.

24. Clinical scenario 24

GPT-4.0

1. Chronological Events

Year	Event
~2010	Lymphoma treatment with chemotherapy in Hospital S
2011	Laparoscopic cholecystectomy
>10 years ago	Partial gastrectomy in the private sector
September 2022	Completed chemotherapy and radiotherapy for pancreatic carcinoma in Hospital SH
December 2022	PET-CT showing pancreatic malignancy
May 2023	PET-CT showing progression of pancreatic malignancy
June 2023	Right PTBD inserted due to malignant biliary obstruction
August 2023	Left external PTBD inserted
October 2023	Left PTBD internalized; right PTBD removed
October 2023	CT abdomen showing necrotic carcinoma and possible new metastases or abscesses

2. Clinical Problem List

Status	Clinical Issues
Active	Inoperable carcinoma of pancreas
Active	Biliary obstruction with PTBD in place
Active	Right abdominal pain with fever
Chronic	History of gastric ulcer with partial gastrectomy
Chronic	Previous treatment for lymphoma
Chronic	Poor marrow function

3. Potential Life-Threatening Complications
- Acute cholangitis secondary to biliary obstruction
- Sepsis secondary to possible biliary or blood infection
- Progression of metastatic pancreatic cancer
- Acute kidney injury secondary to contrast-induced nephropathy or obstruction

4. Clinical Findings

Organ System	Anticipated Findings
General	Possible jaundice, cachexia due to malignancy
Cardiovascular	Hypotension if septic
Respiratory	Clear chest, unless complications such as pleural effusion
Abdominal	Distension, previous PTBD site erythema, bile in bedside bag, non-tender on examination
Skin	Erythema at PTBD site
Renal	Foley bag urine clear, no sediment

5. Working Diagnoses
- Advanced inoperable carcinoma of the pancreas with biliary obstruction
- Right abdominal pain potentially due to cholangitis or hepatobiliary pathology
- Possible hepatic metastasis or abscesses
- Suspected peritoneal metastasis

6. Relevant Investigations

Investigation	Justification
Blood cultures	To identify causative organisms and appropriate antibiotics for suspected sepsis
Bile culture	To guide targeted antimicrobial therapy for biliary infection
Follow-up PET-CT or MRI	To assess the progression of malignancy and confirm new metastases or abscesses
Liver function tests (LFTs)	To monitor biliary obstruction and liver function status
Serial blood tests (CBC, electrolytes, renal function)	To monitor for sepsis, electrolyte imbalance, and renal function

7. Management Plan
- Continue broad-spectrum antibiotics until culture results are available; adjust based on sensitivities.
- Supportive care for symptoms, including analgesics for pain management.
- Monitor vital signs and organ functions closely to detect early signs of sepsis or organ failure.
- Consider consultation with interventional radiology for evaluation of PTBD function and possible need for adjustments.
- Palliative care consultation to optimize quality of life and manage symptoms related to advanced cancer.
- Nutritional support considering the patient's history of gastric surgery and current malignancy.
- Regular monitoring of complete blood count, renal function, and liver function tests.

8. Executive Summary
The patient is a 77-year-old female with a complex medical history including inoperable pancreatic carcinoma, post-chemotherapy poor marrow function, and history of lymphoma. She presents with right abdominal pain and fever, suggestive of cholangitis or another infection. Current management involves broad-spectrum antibiotics while awaiting culture results. Imaging shows stable pancreatic malignancy but potential new hepatic lesions. A multidisciplinary approach, including interventional radiology and palliative care, is essential for optimizing patient outcomes and quality of life. Monitoring of organ function and timely adjustments in treatment based on clinical and investigation findings are crucial.

cGPT-4

1. Chronological Events:

Date	Event
2010	Completed 5 cycles of chemotherapy for lymphoma
2011	Underwent laparoscopic cholecystectomy
September 2022	Completed chemotherapy and radiotherapy for pancreatic carcinoma
December 2022	PET-CT showing pancreatic malignancy
May 2023	Follow-up PET-CT with similar findings and complications
June 2023	Right PTBD inserted
August 2023	Left external PTBD insertion
October 2023	Internalization of left PTBD
27 October 2023	Admitted for right abdominal pain with fever
28 October 2023	CT abdomen performed

2. Clinical Problem List:

Status	Clinical Issues
Active	Right abdominal pain with fever
Active	Advanced inoperable carcinoma of pancreas
Active	Malignant biliary obstruction
Active	Hepatic lesions suggestive of metastasis or abscesses
Chronic	History of gastric ulcer with partial gastrectomy
Chronic	Prior lymphoma treatment
Chronic	History of cholecystectomy

3. Potential Life-Threatening Complications:
- Sepsis due to infection in the context of biliary obstruction and recent invasive procedures.
- Hemorrhage due to thrombocytopenia and coagulopathy secondary to liver dysfunction.
- Acute renal failure due to systemic infection and underlying poor marrow function.

4. Clinical Findings:

Organ System	Findings
General	Afebrile, unsupported blood pressure and pulse, non-septic appearance
Cardiovascular	Unremarkable (unsupported BP and pulse)
Respiratory	Chest clear
Gastrointestinal	Distended but soft non-tender abdomen
Hepatobiliary	Previous PTBD site mild erythema, left PTBD with bile in bedside bag

| Genitourinary | Clear urine with no sediment |

5. Working Diagnoses:
- Biliary sepsis secondary to malignant biliary obstruction
- Hepatic lesions likely representing metastasis
- Acute renal injury secondary to systemic infection

6. Relevant Investigations:
Investigation	Justification
Blood culture	To identify causative organism of suspected biliary sepsis
Bile culture	To tailor antibiotic therapy based on organism sensitivity
Repeat serum creatinine and electrolytes	To monitor renal function and electrolyte balance
Liver function tests	To assess the extent of liver dysfunction
Imaging (e.g., ultrasound or MRI)	To further characterize hepatic lesions and assess for additional complications

7. Management Plan:
- Continue broad-spectrum antibiotics and consider tailoring based on culture results.
- Monitor vital signs and hemodynamic status closely; provide supportive care as needed.
- Regularly assess pain and manage with appropriate analgesics.
- Ensure that the patient's previous medical history, including lymphoma and surgeries, are taken into account when planning treatment.
- Consider palliative care involvement for symptom management and supportive care discussions.
- Regularly review the patient's status in a multidisciplinary team meeting for any changes in the management plan.

8. Executive Summary:
The patient is a 77-year-old female with a complex medical history including pancreatic cancer and previous lymphoma, presenting with right abdominal pain and fever likely due to biliary sepsis. Investigations revealed hepatic lesions and suspected peritoneal metastasis. Management includes broad-spectrum antibiotics, tailored to culture results, and supportive care. Frequent reassessments and multidisciplinary team discussions are recommended to adjust the care plan as needed. Palliative care involvement is advised for comprehensive symptom management.

Gemini Pro

1. Chronological Events:
| Date | Clinical Issue |

>10 years ago	Gastric ulcer, partial gastrectomy
2011	Laparoscopic cholecystectomy
2010	Lymphoma treated with 5 cycles of chemotherapy
September 2022	Advanced inoperable carcinoma of pancreas on best supportive care (BSC) with chemotherapy and radiotherapy
May 2023	PET-CT shows known pancreatic malignancy with mild increase in uptake
June 2023	Right Percutaneous Transhepatic Biliary Drainage (PTBD) inserted
August 2023	Left external PTBD insertion
October 2023	Internalization of left PTBD
October 28, 2023	Admitted for right abdominal pain with fever
October 28, 2023	CT abdomen with contrast performed
October 27, 2023	Urine culture shows no growth
October 27, 2023	Bile culture pending
October 26, 2023	Blood culture pending
October 27, 2023	Preliminary blood culture result: Enterobacter species

2. Clinical Problem List:

Active Problems	Chronic Problems
Advanced inoperable carcinoma of pancreas	Gastric ulcer
Malignant biliary obstruction	Lymphoma
Right abdominal pain	Laparoscopic cholecystectomy
Fever	
Leukocytosis	
Anemia	
Elevated bilirubin	
Elevated alkaline phosphatase	
Elevated alanine aminotransferase	
Reduced creatinine clearance	
Enterobacter species bacteremia	

3. Potential Life-Threatening Complications:
* Sepsis
* Liver failure
* Pancreatic abscess
* Cholangitis
* Renal failure

4. Clinical Findings:

Organ System	Anticipated Findings

General	Afebrile, blood pressure and pulse unsupported, not septic looking
Respiratory	Chest clear
Cardiovascular	No data
Gastrointestinal	Distended but soft non-tender abdomen, previous PTBD site wound with mild surrounding erythema, left PTBD seen with bile in bedside bag
Genitourinary	Foley bag urine clear, no sediment, drip site clean

5. Working Diagnoses:
* Advanced inoperable carcinoma of pancreas
* Malignant biliary obstruction
* Cholangitis
* Enterobacter species bacteremia
* Anemia

6. Relevant Investigations:

Investigation	Justification
Blood culture	To confirm the presence of Enterobacter species bacteremia and determine antibiotic susceptibility
Urine culture	To rule out urinary tract infection as a source of bacteremia
Bile culture	To identify the causative organism of cholangitis and determine antibiotic susceptibility
Liver function tests	To assess liver function and monitor for cholestasis
Renal function tests	To assess renal function and monitor for renal failure
Complete blood count	To monitor for anemia and leukocytosis
Abdominal ultrasound	To assess for pancreatic abscess or other intra-abdominal complications
CT scan of the abdomen and pelvis	To assess for pancreatic abscess or other intra-abdominal complications

7. Management Plan:
* Administer broad-spectrum antibiotics to cover Enterobacter species, such as meropenem or piperacillin-tazobactam |
* Perform blood cultures and monitor for clinical response to antibiotics |
* Monitor liver and renal function |
* Provide supportive care, including pain management and nutritional support |
* Consider surgical intervention if cholangitis does not respond to antibiotics or if pancreatic abscess develops |

8. Executive Summary:
The patient is a 77-year-old female with advanced inoperable carcinoma of the pancreas and malignant biliary obstruction who presented with right abdominal pain and fever. She was found to have Enterobacter species bacteremia and cholangitis. She

is currently being treated with broad-spectrum antibiotics and supportive care. Her prognosis is poor due to her underlying malignancy.

Claude 2

1. Chronological Events

Date	Event
>10 years ago	Gastric ulcer, partial gastrectomy in private sector
2011	Laparoscopic cholecystectomy
2010	Lymphoma, treated with 5 cycles of chemotherapy, details unavailable
September 2022	Completed chemoradiotherapy for advanced pancreatic cancer
31 May 2023	PET-CT showed progression of pancreatic cancer
June 2023	Right PTBD insertion for malignant biliary obstruction
30 August 2023	Left PTBD insertion after right PTBD failed
October 2023	Left PTBD internalized
28 October 2023	Admitted for right abdominal pain and fever
28 October 2023	CT abdomen showed necrotic pancreatic tumor, new hepatic lesions concerning for metastases or abscesses

2. Clinical Problem List

Clinical Problem	Active/Chronic
Advanced pancreatic cancer	Active
Malignant biliary obstruction	Active
Post-chemotherapy cytopenias	Active
Right upper quadrant pain and fever	Active
Possible cholangitis	Active
Possible liver metastases or abscesses	Active
Chronic kidney disease Stage 3	Chronic
Post-gastrectomy status	Chronic
Post-cholecystectomy status	Chronic
History of lymphoma, treated	Chronic

3. Potential Life-Threatening Complications
- Sepsis/septic shock from cholangitis
- Bleeding from thrombocytopenia
- Acute kidney injury from sepsis

4. Anticipated Physical Examination Findings

Organ System	Anticipated Findings
General	Ill-appearing, febrile, tachycardic

HEENT	Pale conjunctivae, dry mucous membranes
Cardiovascular	Tachycardia, hypotension
Pulmonary	Tachypnea, decreased breath sounds, crackles
Abdominal	RUQ tenderness, distension, jaundice, PTBD in place with purulent drainage
Extremities	Peripheral edema

5. Working Diagnoses
- Cholangitis
- Biliary sepsis
- Liver abscesses
- Disease progression with metastases

6. Relevant Investigations
Investigation	Rationale
Blood cultures	To identify bacteremia
Bile cultures	To identify source of infection
CT abdomen with contrast	To further assess liver lesions, disease progression
CBC, renal function	Monitor cytopenias, kidney injury
Coagulation studies	Assess bleeding risk with thrombocytopenia

7. Management Plan
- Initiate broad spectrum antibiotics for presumed cholangitis/biliary sepsis
- Obtain cultures then optimize antibiotics based on sensitivities
- Address thrombocytopenia cautiously given bleeding risk
- Monitor kidney function and intravascular volume status closely
- Interventional radiology consultation for PTBD revision if ongoing obstruction
- Palliative care consultation for goals of care discussion
- Avoid further chemotherapy due to poor marrow function
- Address nutritional needs
- Provide pain control
- Discuss hospice if appropriate

8. Executive Summary
This 77-year-old female with advanced pancreatic cancer and malignant biliary obstruction presented with right upper quadrant pain and fever concerning for cholangitis. She requires broad spectrum antibiotics and drainage of biliary obstruction along with close monitoring for sepsis complications. Her goals of care should be clarified while optimized symptom management is provided.

25. Clinical scenario 25

GPT-4.0

1. Chronological Events

Date	Event
1995	Radiotherapy for nasopharyngeal carcinoma
Before 5 Oct 2023	Diagnosis of osteoradionecrosis of skull base; underlying chronic conditions present
5 Oct 2023	Excision of lesion of nasopharynx, marsupialisation of nasopharyngeal abscess, turbinate reduction; start of Amoxicillin-clavulanate treatment
8 Oct 2023	Start of cefotaxime and metronidazole treatment
14 Nov 2023	Percutaneously inserted central catheter (PICC) inserted
24 Nov 2023	Reassessment CT showing residual infective collection and intraluminal hypodensity in the right proximal vertebral artery

2. Clinical Problem List

Status	Clinical Problem
Chronic	Hypertension
Chronic	Subclinical hypothyroidism
Chronic	Chronic sinusitis
Chronic	History of nasopharyngeal carcinoma (post-radiotherapy in 1995)
Chronic	Adjustment disorder with mixed anxiety and depressed mood
Active	Osteoradionecrosis of skull base
Active	Left parapharyngeal abscess
Active	Residual infective collection post-surgery
Active	Intraluminal hypodensity in the right proximal vertebral artery

3. Potential Life-Threatening Complications

- Sepsis or systemic inflammatory response syndrome (SIRS) due to unresolved infection.
- Stroke due to involvement of the right proximal vertebral artery.
- Airway obstruction from parapharyngeal abscess.

4. Clinical Findings

Organ System	Anticipated Findings
ENT	Chronic changes due to radiotherapy, evidence of recent surgery, signs of abscess
Neurological	Signs of potential cerebrovascular compromise due to vertebral artery hypodensity
Cardiovascular	Hypertension-related changes
Endocrine	Signs related to subclinical hypothyroidism (may be subtle or absent)

5. Working Diagnoses

- Osteoradionecrosis of the skull base secondary to previous radiotherapy.
- Left parapharyngeal abscess potentially complicating osteoradionecrosis.
- Vascular involvement with intraluminal hypodensity in the right proximal vertebral artery suggesting possible thrombus or stenosis.

6. Relevant Investigations

Investigation	Justification
Repeat CT of the skull base and neck	To assess the extent of osteoradionecrosis and any changes in the abscess and vertebral artery
Blood cultures	To identify any bloodstream infection, especially in the context of PICC
Echocardiogram	To rule out endocarditis, which can be associated with persistent bacteremia
Doppler ultrasound of the right vertebral artery	To evaluate the flow and rule out thrombus or stenosis

7. Management Plan
- Continue IV antibiotics, considering sensitivity patterns: switch from cefotaxime to meropenem due to ESBL-producing K. pneumoniae and adjust other antibiotics accordingly.
- Monitor for signs of sepsis and manage with supportive care as needed.
- Address nutritional status and consider enteral or parenteral nutrition given low body weight.
- Coordinate with ENT, infectious diseases, and vascular surgery for the management of osteoradionecrosis, abscess, and vertebral artery issues.
- Involve psychiatry for management of adjustment disorder.

8. Executive Summary
The patient is a 51-year-old female with a history of nasopharyngeal carcinoma treated with radiotherapy, now presenting with complications of osteoradionecrosis and a parapharyngeal abscess. Imaging suggests residual infection and possible vascular involvement. Current antibiotic therapy may need adjustment based on culture results and sensitivities. Management should include a multidisciplinary approach to address the complex ENT and vascular issues, ensure antibiotic coverage is optimized, and provide support for chronic conditions and mental health.

cGPT-4

1. Chronological Events:

Date	Event

1995	Radiotherapy for nasopharyngeal carcinoma.
Before 5 Oct 2023	Presentation with fever, headache, and nasal discharge.
5 Oct 2023	Excision of lesion of nasopharynx, marsupialisation of abscess, turbinate reduction.
8 Oct 2023	Start of amoxicillin-clavulanate, switched to cefotaxime and metronidazole.
8 Nov 2023	Cultures show ESBL-producing K. pneumoniae and other organisms.
14 Nov 2023	PICC line inserted.
24 Nov 2023	CT shows residual infective collection and intraluminal hypodensity in the right vertebral artery.

2. Clinical Problem List:

Status	Clinical Issues
Active	Osteoradionecrosis of skull base with left parapharyngeal abscess.
Active	Fever, headache, nasal discharge.
Active	ESBL-producing Klebsiella pneumoniae infection.
Chronic	Hypertension.
Chronic	Subclinical hypothyroidism.
Chronic	Chronic sinusitis.
Chronic	History of nasopharyngeal carcinoma post-radiotherapy.
Chronic	Adjustment disorder with mixed anxiety and depressed mood.

3. Potential Life-Threatening Complications:
- Septic thrombophlebitis of the right proximal vertebral artery.
- Potential spread of infection leading to meningitis or brain abscess.
- Sepsis from ongoing infection.

4. Clinical Findings:

Organ System	Expected Findings
ENT	Signs of chronic sinusitis, findings of nasopharyngeal lesion.
Neurological	Signs correlating with headaches, possible focal deficits if intracranial extension.
Vascular	Signs of septic thrombophlebitis if present.

5. Working Diagnoses:
- Osteoradionecrosis of the skull base with secondary infection.
- Left parapharyngeal abscess.
- Chronic sinusitis.
- Subclinical hypothyroidism.
- Hypertension.
- Adjustment disorder with mixed anxiety and depressed mood.

6. Relevant Investigations:

Investigation	Justification
Repeat CT/MRI	To assess the extent of osteoradionecrosis and parapharyngeal abscess, evaluate for intracranial complications.
Blood cultures	To monitor the current status of the bacterial infection and antibiotic susceptibility.
Echocardiogram	To rule out endocarditis due to persistent bacteremia.
Thyroid function tests	To monitor subclinical hypothyroidism.
Psychiatric evaluation	To assess and manage adjustment disorder with mixed anxiety and depressed mood.

7. Management Plan:
- Continue targeted antibiotic therapy based on culture sensitivity (meropenem for ESBL-producing K. pneumoniae).
- Surgical consultation for the management of osteoradionecrosis and to evaluate the need for further debridement.
- Regular monitoring and reassessment by the infectious disease team to adjust antimicrobial therapy as needed.
- Vascular surgery or interventional radiology consultation for the vertebral artery hypodensity.
- Supportive care for symptoms, including pain management and fever control.
- Endocrine follow-up for the management of hypertension and subclinical hypothyroidism.
- Mental health support for adjustment disorder with mixed anxiety and depressed mood.

8. Executive Summary:
The patient, a 51-year-old female with a history of nasopharyngeal carcinoma treated with radiotherapy, presents with osteoradionecrosis and a parapharyngeal abscess complicated by infection with multi-drug resistant organisms. Management includes targeted antimicrobial therapy, surgical intervention, and multidisciplinary care for her chronic conditions and mental health. Regular monitoring and re-evaluation are crucial to address any progression or complications of her conditions.

Gemini Pro

1. Chronological Events:

Date	Clinical Issue
1995	History of nasopharyngeal carcinoma with radiotherapy

5 October 2023	Admission for osteoradionecrosis of skull base with left parapharyngeal abscess
5 October 2023	Excision of lesion of nasopharynx, marsupialisation of nasopharyngeal abscess and turbinate reduction
8 November 2023	Reassessment computed tomography (CT) showed residual infective collection (4.5 cm to 3.6 cm), intraluminal hypodensity in the right proximal vertebral artery extending from the origin to C6 level
14 November 2023	Percutaneously inserted central catheter (PICC) inserted

2. Clinical Problem List:

Active	Chronic
Osteoradionecrosis of skull base	Hypertension
Left parapharyngeal abscess	Subclinical hypothyroidism
Chronic sinusitis	Adjustment disorder with mixed anxiety and depressed mood
ESBL-producing Klebsiella pneumoniae infection	Nasopharyngeal carcinoma

3. Potential Life-Threatening Complications:
* Sepsis
* Meningitis
* Vertebral artery thrombosis
* Stroke
* Osteomyelitis

4. Clinical Findings:

Organ System	Findings
General	Weight loss, afebrile
Head and Neck	Nasal discharge
Respiratory	Chronic sinusitis
Cardiovascular	None
Gastrointestinal	None
Musculoskeletal	None
Neurological	None
Integumentary	None

5. Working Diagnoses:
* Osteoradionecrosis of skull base
* Left parapharyngeal abscess
* ESBL-producing Klebsiella pneumoniae infection
* Chronic sinusitis

6. Relevant Investigations:

Investigation	Justification
Blood cultures	To identify the causative organism and determine its antimicrobial susceptibility
Computed tomography (CT) scan of the head and neck	To assess the extent of osteoradionecrosis and parapharyngeal abscess
Magnetic resonance imaging (MRI) scan of the brain and neck	To rule out intracranial complications such as meningitis or stroke
Transesophageal echocardiogram (TEE)	To evaluate for vertebral artery thrombosis
Bone biopsy	To confirm the diagnosis of osteoradionecrosis

7. Management Plan:
* Antibiotics:
* Meropenem or levofloxacin for ESBL-producing Klebsiella pneumoniae infection
* Ampicillin or penicillin G for Enterococcus faecalis infection
* Fluconazole for Candida glabrata infection
* Surgery:
* Debridement of osteoradionecrosis and parapharyngeal abscess
* Supportive care:
* Nutritional support
* Pain management
* Psychological support

8. Executive Summary:
The patient is a 51-year-old female with a history of nasopharyngeal carcinoma, hypertension, subclinical hypothyroidism, chronic sinusitis, and adjustment disorder with mixed anxiety and depressed mood. She was admitted for osteoradionecrosis of the skull base with a left parapharyngeal abscess. She underwent excision of the lesion of the nasopharynx, marsupialisation of the nasopharyngeal abscess, and turbinate reduction. A reassessment CT scan showed residual infective collection and intraluminal hypodensity in the right proximal vertebral artery. She has ESBL-producing Klebsiella pneumoniae infection. The management plan includes antibiotics, surgery, and supportive care.

Claude 2

1. Chronological Events

Date	Event
1995	History of nasopharyngeal carcinoma with radiotherapy
5 October 2023	Excision of lesion of nasopharynx, marsupialisation of nasopharyngeal abscess and turbinate reduction
8 October 2023	Started on cefotaxime and metronidazole

14 November 2023	Percutaneously inserted central catheter (PICC) inserted
24 November 2023	CT showed residual infective collection, intraluminal hypodensity in right proximal vertebral artery

2. Clinical Problem List

Clinical Problem	Active/Chronic
Osteoradionecrosis of skull base	Active
Left parapharyngeal abscess	Active
Residual infective collection	Active
Intraluminal hypodensity in right proximal vertebral artery	Active
Hypertension	Chronic
Subclinical hypothyroidism	Chronic
Chronic sinusitis	Chronic
History of nasopharyngeal carcinoma	Chronic
Adjustment disorder with anxiety and depressed mood	Chronic

3. Potential Life-Threatening Complications
- Sepsis from uncontrolled infection
- Vertebrobasilar stroke from vertebral artery thrombosis

4. Clinical Findings

Organ System	Anticipated Findings
General	Low weight, anxiety, depressed mood
HEENT	Trismus, limited neck extension, nasal discharge
Cardiovascular	Hypertension
Endocrine	Fatigue, weight changes

5. Working Diagnoses
- Osteoradionecrosis with superimposed bacterial and fungal infection
- Residual left parapharyngeal abscess
- Vertebral artery thrombosis
- Anxiety and depressive disorders
- Malnutrition

6. Relevant Investigations

Investigation	Rationale
Blood cultures	To identify bacteremia or fungemia
Imaging - CT head/neck with contrast	Evaluate extent of osteoradionecrosis, abscess, vascular involvement
Thyroid function tests	Screen for overt hypothyroidism

| Psychiatric evaluation | Formally evaluate anxiety and depressive disorders |

7. Management Plan
- IV antibiotics to cover ESBL Klebsiella, Pseudomonas, Enterococcus, anaerobes
- Antifungals for Candida coverage
- Anticoagulation if vertebral artery thrombosis confirmed
- Multidisciplinary input: ENT surgery, interventional radiology, endocrinology, psychiatry, dietetics
- Optimization of nutrition, psychiatric support
- Close monitoring for sepsis, thromboembolic complications

8. Executive Summary
51F with history of nasopharyngeal carcinoma complicated by osteoradionecrosis and parapharyngeal abscess with persistent infection despite surgery and antibiotics. Also found to have possible vertebral artery thrombosis, chronic anxiety and depression. Requires prolonged IV antibiotics and antifungals, imaging to evaluate vascular thrombosis, multidisciplinary input especially for psychiatric issues and malnutrition. Key is controlling residual infection and preventing life-threatening complications like sepsis and stroke.

26. Clinical scenario 26

GPT-4.0

1. Chronological Events:

Date	Event
7 Nov 2023	Admission for biliary obstruction, liver function derangement, high CA 19.9 detected.
8 Nov 2023	ERCP performed, dilated left IHDs with stricture and stent insertion, atypical cells found.
10 Nov 2023	Bile culture grew E. coli, Lactobacillus, S. maltophilia.
15 Nov 2023	Fever, deranged liver function, ERCP with plastic stent insertion, cytology pending.
18 Nov 2023	Transfer to Hospital Q, CT scan, fever, and bilateral pulmonary infiltrates on CXR.
19 Nov 2023	ERCP, stricture in right duct, dilated right anterior duct.
22 Nov 2023	Pending PET-CT appointment.

2. Clinical Problem List:

Status	Clinical Issue
Active	Biliary obstruction
Active	Derangement of liver function

Active	High CA 19.9 level
Active	ERCP-related pancreatitis
Active	Fever
Active	Bilateral pulmonary infiltrates
Chronic	Diabetes mellitus
Chronic	Hypertension
Chronic	Benign prostate hyperplasia
Chronic	Fatty liver
Chronic	Hepatitis B (on entecavir)
Chronic	Ischaemic heart disease

3. Potential Life-Threatening Complications:
- Sepsis due to the presence of fever and potential bile duct infection.
- Cholangitis leading to severe infection and systemic illness.
- Biliary cirrhosis or liver failure due to chronic obstruction.
- Pancreatitis complications such as necrotizing pancreatitis or pseudocyst formation.
- Metastatic cancer suggested by high CA 19.9 levels.

4. Clinical Findings:

Organ System	Anticipated Findings
General	Fever, possible jaundice
Cardiovascular	Hypertension-related changes
Respiratory	Signs consistent with pulmonary infiltrates, like crackles
Abdominal	Tenderness in the right upper quadrant, hepatomegaly
Endocrine	Signs of poorly controlled diabetes
Genitourinary	Enlarged prostate on digital rectal examination
Neurologic	Alert and oriented unless complicated by sepsis or hepatic encephalopathy

5. Working Diagnoses:
- Biliary obstruction likely due to choledocholithiasis or malignancy.
- Acute pancreatitis secondary to ERCP.
- Cholangitis based on fever, deranged liver function tests, and positive bile culture.
- Pulmonary infiltrates, which could be due to pneumonia or atypical infections.
- Possible underlying malignancy suggested by elevated CA 19.9.

6. Relevant Investigations:

Investigation	Justification
ERCP with cytology	To assess the biliary tree anatomy and obtain samples for malignancy suspicion.

Blood cultures	To identify causative organisms for fever and possible cholangitis.
Chest X-ray	To evaluate the extent and nature of pulmonary infiltrates.
PET-CT	To investigate for metastatic disease due to high CA 19.9.
Liver function tests	To monitor liver function and the severity of biliary obstruction.
Serum lipase/amylase	To assess and monitor the course of pancreatitis.

7. Management Plan:
- Continue broad-spectrum antibiotics (meropenem, levofloxacin, amikacin, vancomycin) while awaiting blood culture results.
- Supportive care for acute pancreatitis, including fluid resuscitation, pain management, and nutritional support.
- Close monitoring of liver function tests and imaging to assess the effectiveness of the biliary stent.
- Consultation with a hepatobiliary surgeon for potential surgical intervention if malignancy is confirmed or obstruction persists.
- Regular monitoring and management of chronic conditions (diabetes, hypertension, fatty liver, hepatitis B, and ischaemic heart disease).
- Pulmonary infiltrates need further evaluation by a respiratory medicine specialist to rule out infectious versus non-infectious causes and appropriate treatment.

8. Executive Summary:
The 70-year-old male with multiple comorbidities presents with biliary obstruction, elevated CA 19.9, and post-ERCP pancreatitis. He has acomplex clinical presentation including fever and pulmonary infiltrates. Management has included broad-spectrum antibiotics and supportive care. Urgent investigations, including ERCP with cytology and PET-CT, are pending to rule out malignancy. A multidisciplinary approach is essential for managing his chronic conditions alongside his acute issues. The management plan focuses on infection control, monitoring for complications, and addressing any underlying malignancy.

cGPT-4

1. Chronological Events:

Date	Clinical Issue
7 November	Admission for biliary obstruction, liver function derangement
7 November	High CA 19.9 level detected
8 November	ERCP showing dilated IHDs, stricture, stent inserted, atypical cells in brush cytology
8 November	Bile culture grew E. coli, Lactobacillus, Stenotrophomonas maltophilia
Post-ERCP	Complication with pancreatitis
15 November	Fever, deranged liver function, repeat ERCP with plastic stent insertion

18 November	Transfer to Hospital Q, fever, CT scan findings
19 November	ERCP showing stricture of right duct

2. Clinical Problem List:

Status	Clinical Issue
Active	Biliary obstruction
Active	Elevated CA 19.9 level
Active	Atypical cells in brush cytology
Active	Pancreatitis
Active	Fever
Active	Liver function derangement
Chronic	Diabetes mellitus
Chronic	Hypertension
Chronic	Benign prostate hyperplasia
Chronic	Fatty liver
Chronic	Hepatitis B (on entecavir)
Chronic	Ischaemic heart disease

3. Potential Life-Threatening Complications:
- Cholangitis due to biliary obstruction
- Pancreatitis potentially leading to multi-organ failure
- Hepatic failure due to ongoing liver function derangement
- Sepsis secondary to bile culture growth and fever

4. Clinical Findings:

Organ System	Anticipated Findings
Hepatic/Biliary	Jaundice, right upper quadrant abdominal pain
Pancreas	Epigastric tenderness, nausea
Cardiovascular	Ischemic changes, hypertension
Endocrine	Signs of poorly controlled diabetes
Renal	Changes in urine output, creatinine levels
Infectious	Fever, signs of sepsis

5. Working Diagnoses:
- Biliary stricture with possible malignant etiology (elevated CA 19.9, atypical cells)
- Acute pancreatitis
- Cholangitis (presence of bacteria in the bile)
- Compensated Hepatitis B infection with possible acute exacerbation

6. Relevant Investigations:

Investigation	Justification

|------------------------|---|
ERCP with brush cytology	To diagnose and treat biliary stricture, assess for malignancy
Bile culture	To identify causative organisms for targeted antimicrobial therapy
Blood cultures	To monitor for bacteremia or sepsis
PET-CT	To assess for malignancy
Liver function tests	To monitor liver status and function

7. Management Plan:
- Continue broad-spectrum antibiotics until culture sensitivities are available, then de-escalate accordingly.
- Maintain entecavir for Hepatitis B management.
- Monitor and support pancreatic function, manage pancreatitis conservatively with hydration, pain control, and nutritional support.
- Prepare for potential biliary surgery pending confirmation of malignancy through cytology and imaging.
- Optimize management of chronic conditions like diabetes and ischemic heart disease.

8. Executive Summary:
The patient presents with a complex case involving biliary obstruction, potential malignancy indicated by elevated CA 19.9 and atypical cells, acute pancreatitis, and a background of chronic conditions including diabetes and Hepatitis B. The patient is currently being managed with broad-spectrum antibiotics and has undergone multiple ERCP procedures. Pending investigations include brush cytology, blood cultures, and PET-CT to guide further management. A multidisciplinary approach will be critical for the management of active and chronic issues.

Gemini Pro

1. Chronological Events:
Date	Clinical Event
7 November 2023	Admitted for biliary obstruction with deranged liver function
8 November 2023	ERCP: dilated left intrahepatic ducts (IHDs) with stricture over left hepatic duct, stent inserted, brush cytology showing atypical cells
10 November 2023	Blood culture
11-17 November 2023	Ceftriaxone (1g IV q24h)
15 November 2023	Fever, deranged liver function, ERCP: similar stricture over left hepatic duct, plastic stent inserted, cytology pending
17 November 2023	Blood culture
18 November 2023	Transferred to Hospital Q, CT scan: prominent right IHDs, mildly dilated left (IHD)

18 November 2023	Fever, body weight 79.3 kg, CXR: bilateral pulmonary infiltrates
18 November 2023	Blood culture
19 November 2023	ERCP: cholangiogram shows dilated and tortuous left duct, segment of stricture of right duct, dilated right anterior duct
19 November 2023	Vancomycin started

2. Clinical Problem List:

Active	Chronic
Biliary obstruction	Diabetes mellitus
Deranged liver function	Hypertension
Cholangitis	Benign prostate hyperplasia
Pancreatitis	Fatty liver
Fever	Hepatitis B surface antigen (HBs Ag) positive
Pulmonary infiltrates	Ischaemic heart disease

3. Potential Life-Threatening Complications:

Clinical Problem	Potential Life-Threatening Complications
Biliary obstruction	Liver failure, sepsis
Cholangitis	Sepsis, liver abscess
Pancreatitis	Pancreatic necrosis, sepsis
Fever	Sepsis
Pulmonary infiltrates	Pneumonia, sepsis

4. Clinical Findings:

Organ System	Anticipated Physical Examination Findings
General	Fever, weight loss
Respiratory	Bilateral pulmonary infiltrates
Cardiovascular	Tachycardia, hypotension
Gastrointestinal	Jaundice, hepatomegaly, splenomegaly
Neurological	Confusion, lethargy

5. Working Diagnoses:

Probable Diagnosis
Cholangitis
Pancreatitis
Biliary obstruction
Sepsis
Pneumonia

6. Relevant Investigations:

Investigation	Justification
Blood cultures	To identify the causative organism(s) of cholangitis and sepsis
Liver function tests	To assess liver function and monitor response to treatment
Abdominal ultrasound	To visualize the biliary tree and assess for any abnormalities
CT scan of the abdomen and pelvis	To assess for any underlying structural abnormalities or complications
PET-CT scan	To further evaluate the extent of the disease and identify any occult lesions
Sputum culture	To identify the causative organism(s) of pneumonia

7. Management Plan:

Strategy	Goal
Antibiotics	To treat cholangitis, sepsis, and pneumonia
Fluid resuscitation	To correct dehydration and maintain hemodynamic stability
Nutritional support	To prevent and treat malnutrition
Pain management	To relieve pain and improve comfort
Respiratory support	To provide oxygen and ventilatory support as needed
Endoscopic retrograde cholangiopancreatography (ERCP)	To relieve biliary obstruction and treat cholangitis
Percutaneous transhepatic cholangiodrainage (PTCD)	To provide biliary drainage if ERCP is unsuccessful or not feasible
Surgery	To remove any obstructing lesions or treat complications such as pancreatic necrosis

8. Executive Summary:
A 70-year-old male with a history of diabetes mellitus, hypertension, and ischaemic heart disease presented with biliary obstruction and deranged liver function. He developed cholangitis, pancreatitis, and sepsis. He was transferred to Hospital Q for further management. Investigations revealed dilated intrahepatic ducts, stricture over the left hepatic duct, and pulmonary infiltrates. He is currently receiving antibiotics, fluid resuscitation, and nutritional support. Further investigations and management, including ERCP or PTCD, are planned.

Claude 2

1. Chronological Events

Date	Event

7 November 2023	Admitted for biliary obstruction with deranged LFT. High CA 19-9.
8 November 2023	ERCP showed left hepatic duct stricture, stent inserted. Atypical cells on cytology.
10-15 November 2023	Post-ERCP pancreatitis.
15 November 2023	Repeat ERCP showed similar left hepatic duct stricture, stent re-inserted.
18 November 2023	Transferred to Hospital Q.
18 November 2023	CT: Dilated right and left IHDs.
19 November 2023	ERCP: Stricture of right hepatic duct.

2. Clinical Problem List

Problem	Active/Chronic
Biliary obstruction	Active
High CA 19-9	Active
Left hepatic duct stricture	Active
Post-ERCP pancreatitis	Active
Right hepatic duct stricture	Active
Diabetes mellitus	Chronic
Hypertension	Chronic
Fatty liver	Chronic
Ischemic heart disease	Chronic

3. Potential Life-Threatening Complications
- Sepsis/septic shock from biliary infection
- Acute kidney injury from sepsis
- Multi-organ failure from sepsis

4. Clinical Findings

System	Expected Findings
General	Fever, tachycardia, hypotension (in sepsis)
HEENT	Jaundice, dry mucous membranes (dehydration)
Respiratory	Tachypnea, crackles (pneumonia)
Cardiovascular	Tachycardia, hypotension, cool extremities (shock)
Abdominal	RUQ tenderness, Murphy's sign, abdominal distension
Neurological	Alert, oriented

5. Working Diagnoses
- Cholangiocarcinoma
- Primary sclerosing cholangitis
- IgG4-related disease

- Biliary strictures post-liver transplant
- Biliary infection/cholangitis

6. Relevant Investigations

Investigation	Justification
PET-CT	Evaluate extent of biliary strictures, assess for metastases
Liver biopsy	Confirm diagnosis of malignancy vs other causes
IgG4 levels	Rule out IgG-related disease
Viral serologies	Assess for contribution of viral hepatitis
MRCP	Further evaluate biliary anatomy
Blood cultures	Identify bacteremia source

7. Management Plan
- Treat infection with antibiotics per culture sensitivities
- Maintain biliary drainage with stents
- Avoid hypotension and ensure adequate perfusion
- Monitor renal function and electrolytes
- Nutritional support
- Pain control
- Minimize immunosuppression
- Arrange urgent PET-CT and liver biopsy
- Involve hepatology, oncology, and surgery services

8. Executive Summary
This 70-year-old man with biliary obstruction and strictures requires an expedited workup for suspected cholangiocarcinoma. Immediate priorities are treating infection, ensuring adequate biliary drainage, and preventing complications like sepsis. Further imaging, tissue diagnosis, and input from oncology are urgently needed to establish a definitive diagnosis and treatment plan.

27. Clinical scenario 27

GPT-4.0

1. Chronological Events:

Date	Event
20 December 2022	Left distal radius fracture with open reduction internal fixation (ORIF)
4 December 2022	Operation for incision and drainage due to right hand tenosynovitis, post fish sting injury
4 December 2022 - Present	Administration of Levofloxacin
20 January 2024	Current admission for right hand tenosynovitis

2. Clinical Problem List:

Active Problems	Chronic Problems
Right hand tenosynovitis post fish sting injury	Adjustment disorder
Post-operative condition from incision and drainage	Previous suicidal gesture
Fever and chills related to recent infection	-
-	History of left distal radius fracture

3. Potential Life-Threatening Complications:
- Sepsis from untreated tenosynovitis and possible systemic infection due to the rapid spread of erythema and swelling.
- Possible resistant infection given the intraoperative specimen culture showed no growth (suggestive of atypical organisms or fastidious pathogens).
- Reoccurrence of suicidal gestures due to underlying psychiatric condition.

4. Clinical Findings:

Organ System	Expected Findings
Musculoskeletal	Swelling, erythema, and possible tenderness in the right hand and wrist area
Integumentary	Signs of previous surgical interventions such as scars or healing wounds
Neurological	Normal function expected given patient is well with no active complaints
Psychiatric	Assessment needed for adjustment disorder and past suicidal behavior

5. Working Diagnoses:
- Right hand tenosynovitis secondary to a fish sting.
- Post-operative changes from recent incision and drainage.
- Rule out infectious diseases, including atypical infections due to negative culture and ongoing fever and chills.
- Chronic: Adjustment disorder with a history of suicidal gestures.

6. Relevant Investigations:

Investigation	Justification
AFB culture	To exclude mycobacterial infection given the negative AFB smear and clinical presentation
Repeat blood cultures	To identify any causative organisms that might not have been caught in previous cultures
CRP and ESR	To monitor the inflammatory response and effectiveness of antibiotic therapy

| Psychiatric evaluation | To assess the mental health status and risk of suicide |

7. Management Plan:
1. Continue monitoring the effectiveness of Levofloxacin therapy through clinical signs and serial CRP measurements.
2. Await AFB culture results to rule out mycobacterial infection and adjust antibiotics as needed.
3. Provide supportive care for symptoms including pain management if required.
4. Regular psychiatric evaluation to monitor for any signs of worsening adjustment disorder or suicidal ideation.
5. Educate the patient on the signs of infection, proper wound care, and when to seek immediate medical attention.
6. Discuss rehabilitation and physiotherapy options for the right hand to promote functional recovery post-tenosynovitis treatment.

8. Executive Summary:
The patient, a 52-year-old female with a history of adjustment disorder and suicidal gestures, presented with right hand tenosynovitis following a fish sting. She underwent incision and drainage with a course of Levofloxacin starting on December 4, 2022. The main concerns include the management of her infection, given the negative cultures, and her underlying psychiatric condition. The management plan includes continued antibiotic therapy, monitoring of inflammatory markers, and psychiatric evaluation. Preventive measures and education are also crucial in her care to avoid potential re-infection and to manage her chronic psychiatric condition.

cGPT-4

1. Chronological Events:
Date	Event
20 Dec 2022	Open Reduction Internal Fixation (ORIF) for left distal radius fracture
4 Dec 2022	Operation for right hand tenosynovitis due to fish sting; incision and drainage performed; noted tenosynovitis of ring and little finger extensor tendons

2. Clinical Problem List:
Status	Clinical Issues
Active	Right hand tenosynovitis
Chronic	Adjustment disorder with previous suicidal gesture

3. Potential Life-Threatening Complications:
Immediate life-threatening complications could include systemic infection (sepsis) from the progression of tenosynovitis if not properly treated.

4. Clinical Findings:

Organ System	Anticipated Findings
Musculoskeletal	Swelling, erythema at the wrist and injury site
Skin	Possible erythema, local warmth

5. Working Diagnoses:
The probable diagnoses that correspond with the clinical evidence are post-operative tenosynovitis and adjustment disorder with a history of suicidal gestures.

6. Relevant Investigations:

Investigation	Justification
WBC Count	To monitor for signs of infection or inflammation
CRP Levels	To assess the level of systemic inflammation
AFB Smear and Culture	To rule out atypical infections like tuberculosis in the context of tenosynovitis

7. Management Plan:
The management plan for this patient should include continued monitoring of infection parameters, ensuring proper wound care, and adherence to prescribed antimicrobial therapy with Levofloxacin. Consideration should be given to transitioning to oral antibiotics when appropriate. Mental health support should be provided to manage the chronic adjustment disorder and monitor for suicidal risk. Regular reviews by a multidisciplinary team are recommended.

8. Executive Summary:
The patient is a 52-year-old female with active right hand tenosynovitis and a chronic adjustment disorder with a history of suicidal gestures. The management plan involves close monitoring of infection parameters, adherence to antimicrobial therapy, mental health support, and regular multidisciplinary reviews. No immediate life-threatening complications are present, but the risk of sepsis warrants careful observation.

Gemini Pro

1. Chronological Events:

Date	Event
20 December 2022	Left distal radius fracture with open reduction internal fixation (ORIF)
4 December 2022	Admitted for right hand tenosynovitis, with history of right hand stung by fish sting during work, together with fever and chills

4 December 2022	Operation for incision and drainage, intra-operatively noted tenosynovitis of ring finger and little finger extensor tendons, with moderate amount of dirty fluid and synovitis
4 December 2022	Levofloxacin started

2. Clinical Problem List:

Active	Chronic
Right hand tenosynovitis	Adjustment disorder
Fever and chills	Left distal radius fracture

3. Potential Life-Threatening Complications:
- Sepsis
- Endocarditis
- Osteomyelitis
- Meningitis

4. Clinical Findings:

Organ System	Anticipated Physical Examination Findings
Integumentary	Erythema and swelling of the right hand and wrist
Musculoskeletal	Tenderness and swelling of the right hand and wrist
Respiratory	Fever and chills
Cardiovascular	Tachycardia
Neurological	No focal neurological deficits

5. Working Diagnoses:
- Right hand tenosynovitis
- Septic arthritis of the right wrist
- Osteomyelitis of the left distal radius

6. Relevant Investigations:

Investigation	Justification
Blood cultures	To identify the causative organism of the infection
Wound culture	To identify the causative organism of the infection
X-ray of the right wrist	To evaluate for osteomyelitis
MRI of the right wrist	To evaluate for tenosynovitis and osteomyelitis

7. Management Plan:
- Continue levofloxacin until culture results are available
- If culture results are positive, adjust antibiotics accordingly
- Immobilize the right hand and wrist

- Elevate the right hand and wrist
- Apply cold compresses to the right hand and wrist
- Monitor for signs and symptoms of sepsis, endocarditis, osteomyelitis, and meningitis
- Provide supportive care as needed

8. Executive Summary:
- The patient is a 52-year-old female with a history of adjustment disorder and a left distal radius fracture who presented with right hand tenosynovitis, fever, and chills.
- The patient underwent incision and drainage of the right hand and wrist, and levofloxacin was started.
- The patient is at risk for sepsis, endocarditis, osteomyelitis, and meningitis.
- The patient should undergo further evaluation with blood cultures, wound culture, X-ray of the right wrist, and MRI of the right wrist.
- The patient should be treated with antibiotics, immobilization, elevation, and cold compresses.

Claude 2

1. Chronological Events

Date	Event
Prior to admission	Right hand sting by fish at work
Prior to admission	Fever and chills
Prior to admission	Erythema and swelling spread from injury site to wrist
4 Dec 2022	Incision and drainage, noted tenosynovitis of ring and little finger extensor tendons
4 Dec 2022	Intraoperative culture no growth, AFB smear negative
4 Dec 2022 to present	Levofloxacin therapy

2. Clinical Problem List

Active Problems	Chronic Problems
Right hand tenosynovitis	Adjustment disorder with previous suicidal gesture
Fever and chills	Left distal radius fracture status post ORIF on 20 Dec 2022

3. Potential Life-Threatening Complications
- Sepsis from untreated infection
- Suicidal gestures from underlying psychiatric condition

4. Clinical Findings

Organ System	Expected Findings
Cardiovascular	Tachycardia from fever

Respiratory	Normal
Gastrointestinal	Normal
Genitourinary	Normal
Musculoskeletal	Swelling, erythema, tenderness of right hand
Neurological	Normal

5. Working Diagnoses
- Right hand tenosynovitis
- Cellulitis of right hand/wrist
- Underlying osteomyelitis of right hand bones

6. Relevant Investigations
Investigation	Rationale
Blood cultures	To identify any bacteremia
CRP	To monitor infection response
Right hand X-ray	To evaluate for osteomyelitis
MRI right hand	Further evaluate for osteomyelitis if X-ray unremarkable

7. Management Plan
- Continue levofloxacin to complete full course
- Splinting and elevation of right hand
- Surgical follow-up for potential further debridement
- Infectious diseases consult for antibiotic recommendations
- Psychiatry consult for evaluation of suicidal risk
- Physical/occupational therapy for hand rehabilitation

8. Executive Summary
52F with right hand tenosynovitis after fish sting at work, on levofloxacin. Main issues are managing infection and monitoring for complications like osteomyelitis. Also need to address high risk psychiatric history and prevent suicidal gestures.

28. Clinical scenario 28

GPT-4.0

1. Chronological Events:
Date	Event
17 Nov 2023	Admitted for acute inferior STEMI with cardiogenic shock.
17 Nov 2023	Coronary angiogram performed, revealing multiple stenoses and thrombotic occlusion in RCA.
17 Nov 2023	Primary PCI to RCA and IABP insertion.
17 Nov 2023	Episodes of hypotension and asystole.

17 Nov 2023	Elective intubation and MCS with VA-ECMO established.
19 Nov 2023	PCRTO conducted.
20 Nov 2023	ECMO decannulation.
21 Nov 2023	Extubation, then HFNC, and weaned to room air.
21 Nov 2023	Developed SIRS, fever with chills and rigor.
23 Nov 2023	Groin drain removed.
24 Nov 2023	Administered 1 dose of prednisolone for polyarticular gout.
Unspecified	Plan for left-sided coronary staged PCI.

2. Clinical Problem List:

Status	Clinical Problem
Active	Acute inferior STEMI with cardiogenic shock
Active	Hypotension and episodes of asystole
Active	SIRS with fever, chills, and rigor
Active	Polyarticular tophaceous gout
Chronic	Hypertension
Chronic	Gout

3. Potential Life-Threatening Complications:
- Cardiogenic shock
- Recurrent myocardial infarction
- Severe systemic infection or sepsis
- Multiorgan failure secondary to prolonged hypotension or shock
- Complications from invasive procedures (e.g., bleeding from ECMO cannulation sites)

4. Clinical Findings:

Organ System	Anticipated Findings
Cardiovascular	Signs of cardiogenic shock, hypotension
Musculoskeletal	Swelling, erythema, and pain in affected joints, particularly the knees and hand joints
Integumentary	Signs of inflammation or infection at procedural sites
Respiratory	Abnormal breath sounds if HFNC recently weaned

5. Working Diagnoses:
- Acute inferior ST-segment elevation myocardial infarction (STEMI)
- Cardiogenic shock
- Systemic inflammatory response syndrome (SIRS)
- Polyarticular tophaceous gout

6. Relevant Investigations:

Investigation	Justification
Repeat coronary angiogram	To assess the status post-PCI and plan for further intervention if necessary
Joint aspiration and crystal analysis	To confirm diagnosis of gout and rule out septic arthritis
Echocardiogram	To assess cardiac function post-STEMI and shock
Serial blood cultures	To monitor for ongoing or new infection

7. Management Plan:
- Continue close hemodynamic monitoring and support as needed.
- Analgesia and anti-inflammatory treatment for gout, potentially including colchicine or further corticosteroids if not contraindicated.
- Optimization of heart failure medications, including beta-blockers, ACE inhibitors, or ARBs, and aldosterone antagonists, as tolerated.
- Further staged PCI for left-sided coronary disease, if indicated.
- Rehabilitation and secondary prevention measures for coronary artery disease.
- Monitor for signs of infection, and adjust antimicrobials based on culture results and clinical status.

8. Executive Summary:
The patient, a 71-year-old male with a history of hypertension and gout, presented with acute inferior STEMI and cardiogenic shock. He underwent primary PCI to the RCA, IABP insertion, and required VA-ECMO support, which has been successfully weaned. He has experienced SIRS and is managing polyarticular gout. The plan includes close monitoring, management of his gout, staged PCI for residual coronary disease, and vigilant infection control. The patient is to be supported hemodynamically and provided with comprehensive cardiac rehabilitation.

cGPT-4

1. Chronological Events:
Date	Event
17 Nov 2023	Admission for acute inferior STEMI with cardiogenic shock.
17 Nov 2023	Coronary angiogram performed; primary PCI to RCA; IABP inserted.
19 Nov 2023	PCRTO conducted.
20 Nov 2023	ECMO decannulation.
21 Nov 2023	Extubation; transitioned from HFNC to room air.
23 Nov 2023	Groin drain removed.
24 Nov 2023	Administered 1 dose of prednisolone 20mg for polyarticular gout.

| Ongoing | Experiencing polyarticular tophaceous gout, systemic inflammatory response, low-grade fever. |

2. Clinical Problem List:

Status	Clinical Issue
Active	Acute inferior STEMI
Active	Cardiogenic shock
Active	Polyarticular tophaceous gout
Active	Systemic inflammatory response
Active	Low-grade fever
Chronic	Hypertension
Chronic	Gout

3. Potential Life-Threatening Complications:
- Recurrent myocardial infarction
- Severe sepsis or septic shock
- Refractory cardiogenic shock
- Gout complications such as joint destruction or renal impairment

4. Clinical Findings:

Organ System	Anticipated Findings
Cardiovascular	Hypotension, signs of heart failure
Musculoskeletal	Swelling and tenderness over affected joints
Integumentary	Tophi, erythema over affected joints
Respiratory	Possible respiratory distress if heart failure ensues
General	Fever, signs of systemic inflammation

5. Working Diagnoses:
- Post-acute myocardial infarction status with cardiogenic shock
- Post-ECMO and IABP support status
- Systemic inflammatory response syndrome
- Polyarticular tophaceous gout exacerbation

6. Relevant Investigations:

Investigation	Justification
Serial Cardiac Enzymes	To monitor for ongoing myocardial damage
Joint Aspiration and Analysis	To confirm gout flare and rule out septic arthritis
Echocardiogram	To assess cardiac function and structure post-AMI and interventions

Complete Blood Count (CBC)	To monitor for leukocytosis or other hematological response to SIRS
Renal Function Tests	To assess renal function in light of gout and recent contrast exposure
Liver Function Tests	To monitor hepatic function, which could be affected by medications used

7. Management Plan:
- Continue monitoring and managing cardiac status, consider left-sided coronary staged percutaneous coronary intervention for remaining stenosis.
- Address the ongoing hypotension, possibly adjusting medications or fluids to maintain adequate perfusion.
- Assess the need for continuation or modification of antibiotic therapy considering the negative blood cultures and potential for antibiotic stewardship.
- Manage the polyarticular gout flare with appropriate medications, considering the patient's renal function and avoid NSAIDs due to cardiac status.
- Plan for multidisciplinary team involvement for comprehensive care and to plan for eventual discharge, which includes the primary care physician and potentially visiting nurses for continued management at home.
- Prospective data collection for the audit of management outcomes and potential complications.

8. Executive Summary:
The patient, a 71-year-old male with a history of hypertension and gout, was admitted due to an acute inferior STEMI complicated by cardiogenic shock. Interventional procedures including PCI and IABP were performed, followed by the establishment and subsequent removal of VA-ECMO. The patient is currently managing a systemic inflammatory response and a polyarticular gout flare. A comprehensive management plan includes continued cardiac monitoring, potential staged coronary intervention, antibiotic stewardship, and management of the gout flare. Coordination with a multidisciplinary team will ensure thorough care and appropriate planning for discharge.

Gemini Pro

1. Chronological Events:
Date	Clinical Event
17 November 2023	Admission for acute inferior ST-segment elevation myocardial infarction (STEMI) with cardiogenic shock
17 November 2023	Coronary angiogram: left main (LM) mild disease, short. Left anterior descending (LAD) multiple 60-70% stenosis, left circumflex (LC) 80%, obtuse marginal (OM) 95% stenosis. Dominant right coronary artery (RCA) with thrombotic

occlusion, Thrombolysis in myocardial infarction (TIMI) Flow 0. Primary percutaneous coronary intervention (PCI) to right coronary artery (RCA). |
17 November 2023	Intra-aortic balloon pump (IABP) inserted
19 November 2023	Pump-controlled retrograde trial off (PCRTO)
20 November 2023	ECMO decannulation
21 November 2023	Extubated
23 November 2023	Groin drain removed
24 November 2023	Given 1 dose of prednisolone 20mg for polyarticular gout
25 November 2023	Started IV meropenem
26 November 2023	Restarted IV vancomycin

2. Clinical Problem List:

Active	Chronic
Polyarticular gout	Hypertension
Systemic inflammatory response syndrome (SIRS)	Gout
Fever

3. Potential Life-Threatening Complications:
* Sepsis
* Cardiogenic shock
* Myocardial infarction
* Arrhythmias
* Renal failure
* Respiratory failure

4. Clinical Findings:

Organ System	Anticipated Physical Examination Findings
Cardiovascular	Tachycardia, hypotension, peripheral edema
Respiratory	Tachypnea, dyspnea, rales
Gastrointestinal	Nausea, vomiting, abdominal pain
Musculoskeletal	Polyarticular tophaceous gout, involving all four extremities, most severe over left knee and right hand joints
Neurological	Confusion, lethargy

5. Working Diagnoses:
* Polyarticular gout
* Systemic inflammatory response syndrome (SIRS)
* Fever of unknown origin (FUO)
* Acute kidney injury (AKI)
* Cardiogenic shock

6. Relevant Investigations:

Investigation	Justification
Blood culture	To identify the causative organism of the fever
Urine culture	To rule out urinary tract infection as a source of the fever
Chest X-ray	To assess for pneumonia or other pulmonary infiltrates
Abdominal ultrasound	To assess for intra-abdominal infection
Echocardiogram	To assess cardiac function and rule out endocarditis
Renal function tests	To assess renal function and rule out AKI

7. Management Plan:
* Antibiotics: Continue IV vancomycin and meropenem until the source of the fever is identified and treated.
* Anti-inflammatory therapy: Continue prednisolone for the treatment of gout.
* Fluid resuscitation: Administer intravenous fluids to correct hypotension and maintain adequate organ perfusion.
* Vasopressors: If hypotension persists despite fluid resuscitation, start vasopressors to support blood pressure.
* Mechanical ventilation: If respiratory failure develops, initiate mechanical ventilation.
* Renal replacement therapy: If AKI develops, initiate renal replacement therapy.
* Percutaneous coronary intervention (PCI): Schedule PCI to address the coronary artery stenosis.

8. Executive Summary:
A 71-year-old male with a history of hypertension and gout presented with acute inferior STEMI and cardiogenic shock. He underwent PCI to the RCA and was placed on ECMO. He developed SIRS and fever of unknown origin. Blood cultures were negative. He is currently being treated with antibiotics, anti-inflammatory therapy, and fluid resuscitation. He is scheduled for PCI to address the coronary artery stenosis.

Claude 2

1. Chronological Events

Date	Event
17 Nov 2023	Admitted for acute inferior STEMI with cardiogenic shock. Underwent coronary angiogram and PCI to RCA. IABP inserted.
17 Nov 2023	Intubated and initiated on VA-ECMO via RFA and RFV due to hypotension and asystole episodes.
19 Nov 2023	PCRTO performed.
20 Nov 2023	ECMO decannulation.
21 Nov 2023	Extubated to HFNC then weaned to room air.

24 Nov 2023	Given prednisolone 20mg for polyarticular gout.
23 Nov 2023	Groin drain removed.

2. Clinical Problem List

Clinical Problem	Active/Chronic
Acute inferior STEMI	Active
Cardiogenic shock	Active
Hypertension	Chronic
Gout	Chronic, acutely exacerbated
SIRS	Active
Polyarticular gout	Active

3. Potential Life-Threatening Complications
- Recurrent myocardial ischemia or infarction
- Decompensated heart failure
- Cardiogenic shock
- Arrhythmias including ventricular tachycardia/fibrillation
- Mechanical complications of MI (VSD, MR, LV aneurysm)

4. Clinical Findings

Organ System	Expected Findings
Cardiovascular	Tachycardia, new murmur, crackles, displaced PMI, edema
Respiratory	Crackles, decreased breath sounds, orthopnea
Musculoskeletal	Joint swelling, erythema, decreased range of motion
Integument	Jaundice, diaphoresis
Neurologic	Orientation, sensory/motor deficits

5. Working Diagnoses
- Acute myocardial infarction
- Heart failure
- Gouty arthritis
- SIRS

6. Relevant Investigations

Investigation	Rationale
ECG	Look for ischemic changes, arrhythmias
Cardiac biomarkers	Evaluate for myocardial injury
Chest x-ray	Assess heart size, pulmonary edema
Echocardiogram	Evaluate cardiac function, complications
Joint aspiration	Diagnose gout, exclude septic arthritis

| Blood cultures | Exclude bacteremia/sepsis |
| CRP | Marker of inflammation |

7. Management Plan
- Monitor vitals, ECG, urine output closely
- Optimize medical therapy for MI (antiplatelets, anticoagulant, statin, ACEI/ARB, beta-blocker)
- Treat heart failure (diuretics, inotropes if needed)
- Further revascularization if indicated
- Treat gout flare (colchicine, NSAIDs if tolerated)
- Avoid precipitating factors for gout
- Physical therapy and mobilization
- Thromboprophylaxis
- Monitor for complications and intervene early

8. Executive Summary
71M s/p acute inferior STEMI with cardiogenic shock status-post PCI to RCA occlusion and ECMO, now with polyarticular gout flare. Key issues are managing sequelae of MI, optimizing medical therapy, treating heart failure. Also treat gout flare and avoid precipitants. Monitor closely for life-threatening complications including arrhythmias, recurrent ischemia, cardiogenic shock.

29. Clinical scenario 29

GPT-4.0

1. Chronological Events:
Date	Event
Dec 2013	Right Wide Local Excision (WLE) plus Sentinel Lymph Node Biopsy (SLNB) for breast carcinoma.
Jan 2014	Completion of right mastectomy.
Mar 2023	Diagnosis of carcinoma of the pancreas. Presentation of abdominal pain and deranged liver function.
May 2023	Whipple's operation performed.
Jun 2023	CT scan showing pancreatic tail collection, reducing in size.
Oct 2023	Admitted for acute cholangitis, dilated biliary tree, PTBD inserted, bile culture grew E. coli.
Oct 2023	Transfer to Hospital Q.
Oct 2023	Fever spike on 21st, subsequent bile cultures grew other bacteria.
Oct 2023	CT showed signs suspicious of intestinal obstruction and possible peritoneal carcinomatosis.

Oct 2023	Hemicolectomy and gastrojejunostomy performed with noted peritoneal metastasis.

2. Clinical Problem List:

Status	Clinical Issues
Active	Acute cholangitis
Active	Malignant biliary obstruction
Active	Suspected intestinal obstruction
Active	Peritoneal metastasis with obstruction to the gastrojejunostomy
Chronic	Carcinoma of right breast (treated with WLE, SLNB, mastectomy)
Chronic	Mixed anxiety-depressive disorder (MADD)
Chronic	External haemorrhoids
Chronic	Carcinoma of pancreas (post Whipple's operation)

3. Potential Life-Threatening Complications:
- Acute cholangitis could lead to sepsis or liver failure.
- Malignant biliary obstruction could cause cholangitis or ascending cholangitis, leading to sepsis.
- Intestinal obstruction can lead to bowel ischemia and perforation.
- Peritoneal metastasis may cause widespread abdominal involvement and bowel obstruction, leading to perforation or septic complications.

4. Clinical Findings:

Organ System	Anticipated Findings
General	Low body weight (31.2 kg), possible cachexia
Gastrointestinal	Signs of obstruction, possible palpable mass due to peritoneal metastasis
Hepatobiliary	Jaundice, right upper quadrant pain due to biliary tree dilatation
Musculoskeletal	Muscle wasting due to malignancy and chronic disease
Neurological	Anxiety-depressive symptoms

5. Working Diagnoses:
- Acute cholangitis
- Malignant biliary obstruction
- Intestinal obstruction likely secondary to peritoneal carcinomatosis
- Peritoneal metastasis from known pancreatic carcinoma
- Chronic sequelae of breast carcinoma

6. Relevant Investigations:

Investigation	Justification

Repeat CT abdomen/pelvis	Assess progression of suspected intestinal obstruction and metastasis.
Liver function tests	Monitor for worsening biliary obstruction and liver damage.
Blood cultures	Identify causative organisms for effective antimicrobial therapy in sepsis.
Complete blood count (CBC)	Monitor for infection or anemia.
Electrolytes, BUN, Creatinine	Assess kidney function in the context of sepsis and obstruction.
CA 19-9, CEA	Tumor markers for pancreatic cancer surveillance.

7. Management Plan:
- Continue broad-spectrum antibiotics to treat acute cholangitis and potential sepsis (considering current cultures and sensitivities).
- Nutritional support due to low body weight and cachexia.
- Pain management as appropriate for abdominal pain.
- Oncology referral for management of peritoneal metastasis, including potential palliative care.
- Gastroenterology consultation for the management of biliary obstruction and gastrointestinal complications.
- Multidisciplinary team management including palliative care, psychology support for MADD, and social services for post-hospital care planning.

8. Executive Summary:
The patient, a 69-year-old female with a history of breast and pancreatic carcinoma, presents with acute cholangitis, suspected intestinal obstruction, and peritoneal metastasis. She has undergone significant surgical interventions including a Whipple's operation and most recently a hemicolectomy and gastrojejunostomy. Immediate life-threatening complications include sepsis from acute cholangitis and complications from intestinal obstruction. Management should focus on infection control

cGPT-4

1. Chronological Events:
Date	Event
December 2013	Carcinoma of right breast, WLE plus SLNB
January 2014	Completion of right mastectomy
March 2023	Diagnosis of carcinoma of pancreas
May 2023	Whipple's operation
June 2023	CT scan showing pancreatic tail collection
14 October 2023	Admission for acute cholangitis
18 October 2023	Transfer to Hospital Q
21 October 2023	Spike of fever

26 October 2023	CT suspicious of intestinal obstruction
27 October 2023	Operation for hemicolectomy and gastrojejunostomy, peritoneal metastasis found

2. Clinical Problem List:

Status	Clinical Issues
Active	Carcinoma of pancreas
Active	Acute cholangitis
Active	Suspected intestinal obstruction
Active	Malignant large bowel obstruction
Active	Peritoneal metastases
Chronic	Mixed anxiety-depressive disorder (MADD)
Chronic	External hemorrhoids
Chronic	History of breast carcinoma

3. Potential Life-Threatening Complications:
- Acute cholangitis can lead to sepsis if not properly managed.
- Intestinal obstruction can cause bowel ischemia and perforation.
- Peritoneal metastases indicate advanced cancer with potential for widespread organ dysfunction.

4. Clinical Findings:

Organ System	Anticipated Findings
Gastrointestinal	Abdominal pain, distension
Hepatobiliary	Jaundice, right upper quadrant pain
Neurological	Anxiety or depressive symptoms

5. Working Diagnoses:
- Carcinoma of the pancreatic head with biliary obstruction
- Acute cholangitis
- Intestinal obstruction secondary to peritoneal carcinomatosis

6. Relevant Investigations:

Investigation	Justification
CT scan	To assess for intestinal obstruction and metastases
Bile culture	To identify causative organisms in cholangitis
Liver function test	To monitor liver function post-obstruction

7. Management Plan:

- Continue with broad-spectrum antibiotics to cover for the bile culture results (E. coli, Enterococcus faecium, Citrobacter species, and Enterobacter cloacae complex).
- Monitor liver function tests and adjust the management plan accordingly.
- Manage the complications of the intestinal obstruction and peritoneal metastases, which may include further surgical interventions or palliative care measures depending on the patient's condition and wishes.
- Address the patient's mixed anxiety-depressive disorder with appropriate psychological support and pharmacotherapy.

8. Executive Summary:
The patient is a 69-year-old female with a history of breast carcinoma and a recent diagnosis of pancreatic cancer, currently presenting with acute cholangitis and suspected intestinal obstruction due to peritoneal metastases. Immediate management includes continuation of broad-spectrum antibiotics and monitoring of liver function. Further evaluation and management of the intestinal obstruction and metastatic disease are crucial. Palliative care considerations and psychological support for the patient's MADD should also be part of the comprehensive care plan.

Gemini Pro

1. Chronological Events:

Date	Clinical Event
December 2013	Carcinoma of right breast with right wide local excision (WLE) plus sentinel lymph node biopsy (SLNB)
January 2014	Completion of right mastectomy
March 2023	Carcinoma of pancreas diagnosed, presenting with abdominal pain and deranged liver function test
May 10, 2023	Whipple's operation performed
June 2023	CT scan shows pancreatic tail collection reducing in size
October 14, 2023	Admitted to Hospital R for acute cholangitis
October 18, 2023	Transferred to Hospital Q
October 21, 2023	Spike of fever, then fever down
October 26, 2023	CT scan shows suspicious intestinal obstruction
October 27, 2023	Hemicolectomy and gastrojejunostomy performed

2. Clinical Problem List:

Active	Chronic
Acute cholangitis	Carcinoma of right breast
Pancreatic cancer	Mixed anxiety-depressive disorder (MADD)
Intestinal obstruction	External haemorrhoids
Peritoneal metastasis	

3. Potential Life-Threatening Complications:
* Sepsis
* Pancreatic fistula
* Intra-abdominal abscess
* Perforation
* Obstruction
* Malignant bowel obstruction
* Liver failure
* Renal failure

4. Clinical Findings:

Organ System	Anticipated Findings
Respiratory	Clear lungs, no evidence of consolidation
Cardiovascular	No abnormalities
Gastrointestinal	Abdominal distension, tenderness, and guarding
Hepatic	Jaundice, hepatomegaly
Renal	No abnormalities
Neurological	No abnormalities

5. Working Diagnoses:
* Acute cholangitis
* Pancreatic cancer
* Intestinal obstruction
* Peritoneal metastasis

6. Relevant Investigations:

Investigation	Justification
Complete blood count (CBC)	To assess for infection and anemia
Comprehensive metabolic panel (CMP)	To assess for electrolyte imbalances and liver and renal function
Liver function tests (LFTs)	To assess for liver damage
Abdominal ultrasound	To visualize the biliary tree and pancreas
CT scan of the abdomen and pelvis	To evaluate the extent of the pancreatic cancer and intestinal obstruction
Colonoscopy	To visualize the colon and rectum

7. Management Plan:
* Antibiotics for acute cholangitis
* Chemotherapy for pancreatic cancer
* Surgical intervention for intestinal obstruction and peritoneal metastasis

* Nutritional support
* Pain management
* Palliative care

8. Executive Summary:
The patient is a 69-year-old female with a history of carcinoma of the right breast, pancreatic cancer, and mixed anxiety-depressive disorder. She presented with acute cholangitis and was found to have intestinal obstruction and peritoneal metastasis. She underwent hemicolectomy and gastrojejunostomy. The patient is at risk for life-threatening complications, including sepsis, pancreatic fistula, intra-abdominal abscess, perforation, obstruction, malignant bowel obstruction, liver failure, and renal failure. A comprehensive management plan has been developed to address the patient's clinical problems and prevent complications.

Claude 2

1. Chronological Events
Date	Event
December 2013	Right breast wide local excision + sentinel lymph node biopsy
January 2014	Right mastectomy
March 2023	Diagnosis of pancreatic head carcinoma with biliary obstruction
10 May 2023	Whipple operation
June 2023	CT scan showed reducing pancreatic tail collection
14 October 2023	Admission for cholangitis
18 October 2023	Transfer to Hospital Q
21 October 2023	Fever spike
26 October 2023	CT scan showed suspicious intestinal obstruction
27 October 2023	Hemicolectomy and gastrojejunostomy

2. Clinical Problem List
Clinical Problem	Active/Chronic
Breast cancer	Chronic
Anxiety/depressive disorder	Chronic
External hemorrhoids	Chronic
Pancreatic cancer	Active
Cholangitis	Active
Intestinal obstruction	Active

3. Potential Life-Threatening Complications
- Sepsis from cholangitis
- Bowel ischemia and perforation from obstruction

4. Anticipated Physical Examination Findings

System	Expected Findings
Constitutional	Tachycardia, fever, cachexia
HEENT	Pallor, scleral icterus
Respiratory	Tachypnea
Cardiovascular	Tachycardia, hypotension
Abdominal	Distension, tenderness, hypoactive bowel sounds
Extremities	Edema

5. Working Diagnoses
- Post-Whipple choledochojejunostomy stricture with cholangitis
- Metastatic pancreatic cancer with colonic obstruction

6. Relevant Investigations

Investigation	Justification
Blood cultures	Diagnose bacteremia
Liver function tests	Assess synthetic function in sepsis
Amylase/lipase	Pancreatic obstruction
Tumor markers (CEA, CA19-9)	Assess cancer burden
CT abdomen	Confirm obstruction, assess cancer spread
Flexible sigmoidoscopy	Evaluate obstructing lesion

7. Management Plan
- IV antibiotics for cholangitis/bacteremia
- IV fluids and supportive care for sepsis
- Urgent surgical relief of obstruction
- Exploration with possible diverting colostomy/ileostomy
- Biopsy of obstructing lesion
- Nutrition consult for supplementation
- PT/OT for rehabilitation
- Palliative care consult for goals of care discussion
- Monitoring for improved biliary drainage after surgery

8. Executive Summary

This 69F with h/o breast and pancreatic cancer, s/p Whipple, developed cholangitis and likely metastatic colonic obstruction. Urgent management includes IV abx, resuscitation, surgical decompression, and biopsy of obstructing lesion. Overall care goals should be discussed given advanced malignancy.

30. Clinical scenario 30

GPT-4.0

1. Chronological Events

Date	Event
2014 & 2022	Percutaneous coronary intervention (PCI)
August 2023	Elective coronary angiogram and PCI leading to cardiogenic shock, multiorgan failure
27 August 2023	Left ventricular assist device (LVAD) implantation
9 November 2023	CRT-D device change
24 November 2023	Transfer back to Hospital Q due to fever and hypotension
29 November 2023	Metabolic acidosis and respiratory distress; intubation; CT abdomen and pelvis
29 and 30 November 2023	Antibiotics for suspected early acalculous cholecystitis

2. Clinical Problem List

Status	Clinical Issues
Active	Cardiogenic shock
Active	Multiorgan failure
Active	Fever and hypotension
Active	Metabolic acidosis
Active	Respiratory distress
Active	Early acalculous cholecystitis
Chronic	ACPA-positive rheumatoid arthritis
Chronic	Chronic hepatitis B carrier
Chronic	History of myocarditis
Chronic	Coronary artery disease with ischemic cardiomyopathy
Chronic	Recurrent ventricular tachycardia episodes
Chronic	LVAD implantation
Chronic	CRT-D device

3. Potential Life-Threatening Complications

- Cardiogenic shock can lead to irreversible organ damage and death.
- Multiorgan failure is an immediate threat to life, requiring intensive support.
- Respiratory distress, if not managed promptly, can result in respiratory failure and cardiopulmonary arrest.
- Early acalculous cholecystitis, if it progresses, can lead to sepsis.

4. Clinical Findings

Organ System	Anticipated Findings
Cardiovascular	Cardiogenic shock, VT episodes
Respiratory	Respiratory distress, high flow O2 requirement

Gastrointestinal	Early signs of cholecystitis
Integumentary	Grade 4 sacral sore
Neurological	Alert and conscious, fully communicable

5. Working Diagnoses
- Cardiogenic shock post PCI
- Multiorgan failure secondary to cardiogenic shock
- Respiratory distress potentially due to underlying infection or cardiac issues
- Early acalculous cholecystitis
- Sepsis secondary to Elizabethkingia spp., Stenotrophomonas maltophilia, and Pseudomonas aeruginosa infection

6. Relevant Investigations

Investigation	Justification
Bronchoalveolar lavage (BAL)	To identify causative pathogens of respiratory infection
Blood cultures	To identify bloodstream infections leading to sepsis
Echocardiography	To assess cardiac function post-cardiogenic shock and LVAD performance
Abdominal ultrasound	To monitor for changes in cholecystitis
Wound cultures	To identify pathogens involved in the sacral sore and guide antibiotic therapy

7. Management Plan
- Continue with IV meropenem, metronidazole, vancomycin, and fluconazole as per the current antimicrobial regimen.
- Monitor vital signs and organ function closely to detect any signs of deterioration.
- Supportive care for respiratory distress including high flow oxygen and mechanical ventilation if necessary.
- Manage metabolic acidosis with appropriate IV fluids and electrolyte correction.
- Regular wound care for the sacral sore and assessment for any signs of infection.
- Consult with surgical team regarding the management of early acalculous cholecystitis.
- Ensure appropriate nutritional support and physiotherapy to prevent muscle wasting and bedsores.

8. Executive Summary
The 62-year-old male with a complex history of ACPA-positive RA, chronic hepatitis B, myocarditis, coronary artery disease, and recurrent VT, complicated by recent cardiogenic shock post-PCI and multiorgan failure, is now facing new active clinical issues including respiratory distress and early acalculous cholecystitis. Immediate life-threatening complications include further organ failure and sepsis. Management

includes continuation of broad-spectrum antibiotics, close monitoring, and supportive care. Investigations are underway to tailor treatment, such as BAL and blood cultures. The focus is on stabilizing the patient's condition and preventing further complications.

cGPT-4

1. Chronological Events:

Date	Event
August 2023	Elective coronary angiogram and PCI, cardiogenic shock, arrest, ECMO, RRT, respiratory support.
27 August 2023	LVAD implantation.
9 November 2023	CRT-D device change.
24 November 2023	Transfer to Hospital Q due to fever and hypotension.
29 November 2023	Metabolic acidosis and respiratory distress, intubation, CT abdomen and pelvis, suspected early acalculous cholecystitis.

2. Clinical Problem List:

Status	Clinical Issues
Active	Cardiogenic shock and arrest
Active	Multiorgan failure
Active	Metabolic acidosis
Active	Respiratory distress
Chronic	ACPA-positive RA
Chronic	Chronic hepatitis B
Chronic	Coronary artery disease
Chronic	Ischaemic cardiomyopathy
Chronic	Recurrent ventricular tachycardia

3. Potential Life-Threatening Complications:
- Acute: Cardiogenic shock, multiorgan failure, metabolic acidosis, respiratory distress.
- Chronic: Potential exacerbation of RA, reactivation of hepatitis B, progression of coronary artery disease leading to myocardial infarction, decompensation of cardiomyopathy, life-threatening arrhythmias due to VT.

4. Clinical Findings:

Organ System	Findings
Cardiovascular	History of cardiogenic shock, VT episodes
Respiratory	Respiratory distress, high flow O2 requirement
Musculoskeletal	Underlying RA
Integumentary	Grade 4 sacral sore

| Gastrointestinal | Suspected early acalculous cholecystitis |

5. Working Diagnoses:
- Cardiogenic shock likely due to underlying ischaemic cardiomyopathy.
- Respiratory distress and metabolic acidosis secondary to multiorgan failure.
- Early acalculous cholecystitis.
- Infection evidenced by fever, hypotension, and positive microbiology cultures.

6. Relevant Investigations:

Investigation	Justification
Bronchoalveolar lavage	To identify causative organisms for respiratory infection
Repeat CT abdomen and pelvis	To monitor the status of suspected cholecystitis and intra-abdominal collections
Blood cultures	To identify bloodstream infections
Liver function tests	To monitor chronic hepatitis B status and function
Echocardiogram	To assess cardiac function post-PCI and LVAD implantation

7. Management Plan:
- Continue IV meropenem and metronidazole for broad-spectrum coverage of detected bacterial pathogens.
- Maintain IV vancomycin until further culture data is available.
- Oral fluconazole as antifungal coverage.
- Monitor antibiotic efficacy and adjust regimen as needed based on microbiology results and clinical response.
- Close monitoring of the patient's cardiac, renal, and hepatic function.
- Regular wound care for the sacral sore to prevent further infection and promote healing.
- Consideration of gallbladder intervention if cholecystitis does not resolve with conservative management.
- Daily review by a nurse and weekly multidisciplinary team meeting to evaluate the patient's progress and treatment plan.

8. Executive Summary:
The patient, a 62-year-old male with a complex medical history including ACPA-positive RA, chronic hepatitis B, coronary artery disease, and recurrent VT, is currently facing multiorgan failure following cardiogenic shock. He is on broad-spectrum antibiotics for multiple infections and requires close monitoring of his cardiac function and potential early acalculous cholecystitis. A multi-disciplinary team approach is critical in managing his care, with regular reviews to tailor his treatment plan.

Gemini Pro

1. Chronological Events:

Date	Clinical Event
August 2023	Elective coronary angiogram and PCI, complicated by cardiogenic shock and on table arrest leading to multiorgan failure, requiring ECMO, RRT, and respiratory support.
27 August 2023	LVAD implantation
9 November 2023	CRT-D device change
24 November 2023	Transfer back to Hospital Q due to fever and hypotension
29 November 2023	Condition complicated by metabolic acidosis and respiratory distress, intubated. Extubated now.
29 November 2023	Contrast-enhanced CT abdomen and pelvis: No sizable rim-enhancing collection over LVAD exit cable exit site. Previously noted left adductor intramuscular collection shows interval reduction in size, now measuring 2.8 x 1.8 x 2.8cm. A 3.9 x 1.1 x 2.4cm subcutaneous collection over the left sacral region with intralesional gas density, which appears to be connected to the overlying skin defect, could represent sacral sore. No sizable rim-enhancing intra-abdominal collection. Small amount of pelvic ascites. Irregular thickened gallbladder wall up to 0.6cm. Small amount of pericholecystic fluid and stranding. Gallbladder is not distended. No hyperdense gallstone. Overall features may represent early cholecystitis.
29 and 30 November 2023	Surgical assessment: to continue antibiotics for suspected early acalculous uncomplicated cholecystitis
30 November 2023	IV meropenem started
30 November 2023	IV metronidazole started

2. Clinical Problem List:

Active	Chronic
Fever	Rheumatoid arthritis
Hypotension	Chronic hepatitis B
Metabolic acidosis	Coronary artery disease with ischaemic cardiomyopathy
Respiratory distress	History of myocarditis
Sacral sore	Recurrent ventricular tachycardia episodes
Early cholecystitis	

3. Potential Life-Threatening Complications:
- Sepsis
- Multiorgan failure
- Cardiac arrhythmias
- Cholecystitis with perforation

4. Clinical Findings:

Organ System	Anticipated Physical Examination Findings

|---|---|
Respiratory	Nasal interface, high flow O2
Cardiovascular	Blood pressure unsupported
Gastrointestinal	Abdomen soft, non-tender
Musculoskeletal	Grade 4 sacral sore

5. Working Diagnoses:
- Sepsis
- Early acalculous uncomplicated cholecystitis
- Sacral sore

6. Relevant Investigations:
Investigation	Justification
Blood cultures	To identify the causative organism and guide appropriate antimicrobial therapy
Urine cultures	To rule out urinary tract infection as a source of sepsis
Chest X-ray	To assess for pneumonia or other pulmonary infiltrates
Abdominal ultrasound	To assess for gallbladder wall thickening, pericholecystic fluid, and gallstones
Wound culture	To identify the causative organism and guide appropriate antimicrobial therapy

7. Management Plan:
- Continue broad-spectrum antibiotics to cover potential pathogens
- Provide supportive care, including fluid resuscitation, vasopressors, and mechanical ventilation as needed
- Monitor for signs of clinical improvement or deterioration
- Perform necessary investigations to confirm the diagnosis and guide further management
- Consult with relevant specialists, including infectious diseases, cardiology, and surgery, as appropriate

8. Executive Summary:
The patient is a 62-year-old male with a history of RA, chronic hepatitis B, coronary artery disease, and recurrent ventricular tachycardia episodes. He presented with fever, hypotension, metabolic acidosis, and respiratory distress. Contrast-enhanced CT abdomen and pelvis revealed early cholecystitis and a sacral sore. The patient is currently receiving broad-spectrum antibiotics and supportive care. Investigations are underway to confirm the diagnosis and guide further management.

Claude 2

1. Chronological Events

Date	Event
August 2023	Elective coronary angiogram and PCI, complicated by cardiogenic shock and cardiac arrest requiring ECMO, RRT, respiratory support
27 August 2023	LVAD implantation
9 November 2023	CRT-D device change
24 November 2023	Transfer back to Hospital Q due to fever and hypotension
29 November 2023	Condition complicated by metabolic acidosis and respiratory distress, intubated
29 November 2023	Contrast CT abdomen and pelvis showed possible early cholecystitis
30 November 2023	Extubated

2. Clinical Problem List

Problem	Status
ACPA-positive rheumatoid arthritis	Chronic
Chronic hepatitis B	Chronic
History of myocarditis	Chronic
Coronary artery disease s/p PCI x2, ventricular tachycardia	Chronic
Cardiogenic shock and cardiac arrest s/p ECMO, RRT	Active
LVAD implantation	Active
Fever and hypotension	Active
Metabolic acidosis and respiratory distress, intubated	Active
Possible early cholecystitis	Active

3. Potential Life-Threatening Complications
- Septic shock from infection
- Recurrent ventricular tachycardia / cardiac arrest
- Device failure (LVAD, CRT-D)
- Hemorrhage from coagulopathy

4. Clinical Findings

System	Findings
Cardiovascular	Tachycardia, hypotension
Respiratory	Tachypnea, increased work of breathing
Abdominal	Soft, non-tender
Integument	Grade 4 sacral sore
Neurologic	Alert, oriented

5. Working Diagnoses

- Sepsis from possible biliary source
- Healthcare-associated pneumonia
- Device-related infection (LVAD, CRT-D)
- Acute kidney injury

6. Relevant Investigations

Investigation	Rationale
Blood cultures	To identify bacteremia source
Sputum cultures	To identify respiratory pathogen
BAL culture	To identify respiratory pathogen
LFTs, lipase	To assess for biliary obstruction, pancreatitis
LVAD cultures	To assess for device infection
CXR	To assess for pneumonia
BNP	To assess volume status, rule out HF exacerbation

7. Management Plan
- ICU monitoring
- Hemodynamic support with fluids, vasopressors
- Broad spectrum antibiotics (meropenem, vancomycin)
- Address source control (drainage of infection if identified)
- Daily cultures to de-escalate antibiotics
- Strict glucose control
- DVT prophylaxis, stress ulcer prophylaxis
- Nutritional support
- Physical therapy for mobilization
- Evaluate LVAD function and settings
- Minimize arrhythmogenic triggers
- Ongoing wound care

8. Executive Summary
This chronically ill patient with multiple cardiac devices developed sepsis likely from pneumonia and possible cholecystitis after LVAD placement. Aggressive hemodynamic support, broad antibiotics, source control, and monitoring for life-threatening complications like arrhythmias and device failure are recommended while pursuing cultures to guide de-escalation.

31. Clinical scenario 31

GPT-4.0

1. Chronological Events

Date	Event

24 Nov 2023	Left pleural pigtail inserted; Pleural fluid analysis: TCC 180 x10^6/L; polymorphs 53%.
25 Nov 2023	Previous left internal jugular vein CVC removed due to noted pus.
20 Nov 2023	Ascitic fluid cell count: 187 x10^6/L (neutrophils 52%).
2 Dec 2023	Breakthrough fever while on piperacillin-tazobactam; septic workup repeated.
3 Dec 2023	Ascitic fluid cell count improvement: 71 x10^6/L (neutrophils 35%).
4 Dec 2023	Noted desaturation; CXR showed new bilateral infiltrates.

2. Clinical Problem List

Status	Clinical Issue
Active	Decompensated liver cirrhosis
Active	Hepatic encephalopathy
Active	Hepatorenal syndrome with AKI
Active	Possible ventilator-associated pneumonia (VAP)
Chronic	Primary biliary cholangitis
Chronic	Hyperlipidemia
Chronic	Left renal angiomyolipoma

3. Potential Life-Threatening Complications

- Hepatic encephalopathy: can lead to increased intracranial pressure, coma, and death.
- Hepatorenal syndrome with AKI: can progress to multiorgan failure.
- Decompensated liver cirrhosis: risks include variceal bleeding, spontaneous bacterial peritonitis, and liver failure.
- VAP or sepsis: can lead to septic shock and acute respiratory distress syndrome (ARDS).

4. Clinical Findings

Organ System	Anticipated Findings
Respiratory	Decreased air entry, new bilateral infiltrates on CXR
Cardiovascular	Pansystolic murmur at left lower sternal border
Abdomen	Distended and soft
Extremities	No lower limb edema; calves soft
Other	Temperature of 37.7’C; desaturation noted

5. Working Diagnoses

- Decompensated liver cirrhosis with hepatic encephalopathy and hepatorenal syndrome.
- Suspected ventilator-associated pneumonia or sepsis due to desaturation and fever.
- Possible spontaneous bacterial peritonitis given the ascitic fluid neutrophil counts.

6. Relevant Investigations

Investigation	Justification
Repeat ascitic fluid analysis and culture	Monitor for infection or improvement in spontaneous bacterial peritonitis (SBP).
Blood cultures	Identify causative organism for sepsis or VAP.
Chest X-ray	Evaluate for progression of pulmonary infiltrates.
Liver function tests	Monitor liver function and progression of liver disease.
Renal function tests	Assess kidney function in the context of hepatorenal syndrome.

7. Management Plan

- Continue with broad-spectrum antibiotics while awaiting culture results; consider antifungal coverage if risk factors are present.
- Monitor hemodynamic status closely, and provide supportive care for hepatorenal syndrome; consider terlipressin and albumin as per protocols.
- Ensure adequate nutritional support and management of hepatic encephalopathy with lactulose and rifaximin.
- Evaluate the need for and timing of liver transplantation; manage complications such as variceal bleeding, ascites, and SBP prophylaxis.
- Pulmonary hygiene measures to prevent VAP, chest physiotherapy, and possibly bronchodilators if indicated.

8. Executive Summary

The patient is a 61-year-old female with a complex clinical presentation including decompensated liver cirrhosis, hepatic encephalopathy, hepatorenal syndrome, and potential VAP or sepsis. Key management priorities include maintaining hemodynamic stability, managing hepatic complications, and addressing potential infections with appropriate antimicrobial therapy. The patient's condition necessitates close monitoring, with adjustments to the treatment plan based on the results of ongoing and repeated investigations. A multidisciplinary approach including hepatology, nephrology, and critical care is essential for optimal management and evaluation for liver transplantation.

cGPT-4

1. Chronological Events:

Date	Clinical Issue
20 Nov 2023	Ascitic fluid cell count 187 x10^6/L (neutrophil 52%)
24 Nov 2023	Pleural pigtail inserted, pleural fluid TCC 180 x10^6/L; polymorphs 53%
25 Nov 2023	Left internal jugular vein CVC removed due to pus

2 Dec 2023	Breakthrough fever on piperacillin-tazobactam; blood, CSU, IV line tip cultures no growth
3 Dec 2023	Ascitic fluid cell count 71 x10^6/L (neutrophil 35%); culture pending
4 Dec 2023	New bilateral infiltrates noted on CXR

2. Clinical Problem List:

Status	Clinical Issue
Active	Decompensated cirrhosis
Active	Hepatic encephalopathy
Active	Hepatorenal syndrome with AKI
Active	Septic workup due to breakthrough fever
Chronic	Primary biliary cholangitis
Chronic	Hyperlipidemia
Chronic	Left renal angiomyolipoma

3. Potential Life-Threatening Complications:
- Acute kidney injury (AKI) potentially leading to chronic kidney disease or end-stage renal failure.
- Decompensated liver cirrhosis progressing to multi-organ failure.
- Hepatic encephalopathy could lead to increased intracranial pressure and coma.
- Pneumonia, as suggested by new bilateral infiltrates on CXR, can lead to severe sepsis or acute respiratory distress syndrome (ARDS).

4. Clinical Findings:

Organ System	Anticipated Findings
Respiratory	Decreased air entry, pleural effusion
Cardiovascular	Pansystolic murmur at left lower sternal border
Abdominal	Distended abdomen
Renal	Adequate urine output with terlipressin and albumin

5. Working Diagnoses:
- Decompensated liver cirrhosis with associated complications (hepatic encephalopathy, hepatorenal syndrome).
- Suspected hospital-acquired pneumonia (judging from new bilateral infiltrates on CXR and desaturation event).
- Possible line-related bloodstream infection (evidenced by pus noted on CVC, though cultures yielded no growth).

6. Relevant Investigations:

Investigation	Justification

Blood cultures	To identify causative agent of sepsis or line-related infection.
Ascitic fluid analysis and culture	To monitor for spontaneous bacterial peritonitis (SBP).
Chest X-ray	To assess for pneumonia or other pulmonary complications.
Liver function tests	To monitor liver status and function.
Renal function tests	To assess kidney function in the context of hepatorenal syndrome.

7. Management Plan:
- Continue hemodynamic support as needed.
- Continue CRRT for AKI if required.
- Monitor and adjust drug dosing according to renal function.
- If pneumonia is confirmed, adjust antibiotic therapy based on culture results and sensitivities.
- Manage hepatic encephalopathy with lactulose and rifaximin.
- Plan for potential liver transplant, ensuring patient is optimized for surgery.

8. Executive Summary:
The patient is a 61-year-old female with decompensated cirrhosis and primary biliary cholangitis, currently facing acute complications including hepatic encephalopathy and hepatorenal syndrome with AKI. She has exhibited signs of possible hospital-acquired pneumonia and sepsis, with a recent breakthrough fever despite antibiotic treatment. A comprehensive management plan addressing renal support, targeted antibiotic therapy, and preparation for liver transplantation is in place. Close monitoring and adjustment of treatment will be critical for managing her complex condition.

Gemini Pro

1. Chronological Events:

Date	Clinical Issue
20 November 2023	Transferred from Hospital W to Hospital Q for further management of decompensated liver cirrhosis complicated with hepatic encephalopathy, hepatorenal syndrome with AKI.
24 November 2023	Left pleural pigtail inserted, TCC 180 x10^6/L; polymorphs 53%.
25 November 2023	Previous left internal jugular vein central venous catheter (CVC) noted pus, removed.
2 December 2023	Breakthrough fever while on piperacillin-tazobactam, septic workup repeated.
3 December 2023	Ascitic fluid cell count 71 x10^6/L (neutrophil 35%).
4 December 2023	Chest X-ray (CXR) noted new bilateral infiltrates.

2. Clinical Problem List:

Active	Chronic
Decompensated liver cirrhosis	Primary biliary cholangitis
Hepatic encephalopathy	Hyperlipidemia
Hepatorenal syndrome	Left renal angiomyolipoma
Acute kidney injury (AKI)	
Sepsis	
Pneumonia	

3. Potential Life-Threatening Complications:
* Hepatic failure
* Renal failure
* Septic shock
* Respiratory failure

4. Clinical Findings:

Organ System	Anticipated Physical Examination Findings
Respiratory	Bilateral decreased air entry, pansystolic murmur best heard at left lower sternal border
Cardiovascular	Not tachycardiac (on beta blocker for cirrhosis)
Gastrointestinal	Abdomen distended and soft
Musculoskeletal	No lower limb edema, calves soft
Integumentary	Lines exit site not inflamed looking

5. Working Diagnoses:
* Decompensated liver cirrhosis
* Hepatic encephalopathy
* Hepatorenal syndrome
* Acute kidney injury (AKI)
* Sepsis
* Pneumonia

6. Relevant Investigations:

Investigation	Justification
Complete blood count (CBC) with differential	To assess for anemia, leukocytosis, and thrombocytopenia.
Comprehensive metabolic panel (CMP)	To assess for electrolyte imbalances, renal function, and liver function.
Liver function tests (LFTs)	To assess for liver damage.
Coagulation studies	To assess for coagulopathy.

Blood cultures	To identify the causative organism of sepsis.
Urine cultures	To identify the causative organism of urinary tract infection.
Sputum cultures	To identify the causative organism of pneumonia.
Chest X-ray	To assess for pneumonia or other pulmonary abnormalities.
Abdominal ultrasound	To assess for liver cirrhosis and hepatorenal syndrome.

7. Management Plan:
* Continue antibiotics for sepsis and pneumonia.
* Monitor for and manage complications of sepsis and pneumonia.
* Provide supportive care, including fluid resuscitation, electrolyte replacement, and nutritional support.
* Consider liver transplant for decompensated liver cirrhosis.
* Manage underlying conditions, such as primary biliary cholangitis, hyperlipidemia, and left renal angiomyolipoma.

8. Executive Summary:
The patient is a 61-year-old female with decompensated liver cirrhosis, hepatic encephalopathy, hepatorenal syndrome, and AKI. She is currently septic and has pneumonia. The patient is at risk for life-threatening complications, including hepatic failure, renal failure, septic shock, and respiratory failure. A comprehensive management plan has been developed to address the patient's current and potential complications.

Claude 2

1. Chronological Events
Date	Event
Prior to admission	Known primary biliary cholangitis with decompensated cirrhosis, on liver transplant list.
Prior to admission	Left renal angiomyolipoma.
Prior to transfer	Admitted to Hospital W for decompensated liver cirrhosis with hepatic encephalopathy and hepatorenal syndrome with AKI. Required intubation and CRRT.
After transfer	Transferred to Hospital Q for further management.
24 Nov 2023	Left pleural pigtail inserted. Pleural fluid analysis: TCC 180 x10^6/L, polymorphs 53%.
20 Nov 2023	Ascitic fluid analysis: TCC 187 x10^6/L, neutrophils 52%.
25 Nov 2023	Previous left IJ CVC removed due to pus.
2 Dec 2023	Breakthrough fever on piperacillin-tazobactam.
3 Dec 2023	Ascitic fluid analysis: TCC 71 x10^6/L, neutrophils 35%.
4 Dec 2023	Desaturation, new bilateral infiltrates on CXR.

2. Clinical Problem List

Clinical Problem	Active/Chronic
Decompensated cirrhosis	Chronic
Hepatic encephalopathy	Active
Hepatorenal syndrome with AKI	Active
Pleural effusion	Active
Ascites	Active
Fever	Active
Bilateral pulmonary infiltrates	Active
Respiratory failure with desaturation	Active

3. Potential Life-Threatening Complications
- Worsening hepatic encephalopathy leading to cerebral edema and herniation
- Progression of hepatorenal syndrome and acute kidney injury
- Sepsis/septic shock from undiagnosed infection
- Acute respiratory failure requiring mechanical ventilation

4. Anticipated Physical Examination Findings

Organ System	Expected Findings
Respiratory	Decreased breath sounds, crackles/rales, respiratory distress
Cardiovascular	Tachycardia, hypotension, diminished peripheral pulses, murmur
Abdominal	Distended abdomen, ascites, collateral circulation
Neurological	Altered mental status, asterixis, hyperreflexia
Integument	Jaundice, palmar erythema, spider angiomata

5. Working Diagnoses
- Spontaneous bacterial peritonitis
- Healthcare-associated pneumonia
- Hepatorenal syndrome
- Hepatic encephalopathy
- Septic shock

6. Relevant Investigations

Investigation	Justification
Blood cultures	To identify bacteremia source of infection
Respiratory virus panel	Assess for viral pneumonia
Sputum cultures	Diagnose bacterial pneumonia
Ascitic fluid cell count/culture	Diagnose spontaneous bacterial peritonitis
Kidney function tests	Monitor hepatorenal syndrome and AKI
Liver function tests	Assess severity of hepatic dysfunction

Coagulation studies	Assess coagulopathy risk
Blood gases	Evaluate respiratory failure
Chest imaging	Visualize pneumonia/effusions

7. Management Plan
- Administer albumin and continue terlipressin for hepatorenal syndrome
- Initiate broad-spectrum antibiotics for possible sepsis
- Consider intubation if respiratory failure worsens
- Continue CRRT if AKI persists
- Monitor for rising intracranial pressure and cerebral edema
- Paracentesis for tense ascites and SBP diagnosis/treatment
- Address coagulopathy with blood products if indicated
- Consider transfer to ICU given clinical instability

8. Executive Summary
61F with decompensated cirrhosis developing fever, desaturation and bilateral infiltrates during hospitalization. Leading diagnoses include healthcare-associated pneumonia and spontaneous bacterial peritonitis. Management priorities are treating presumed infection, supporting respiratory and kidney function, and monitoring for sepsis complications.

32. Clinical scenario 32

GPT-4.0

1. Chronological Events
Date	Event
Early Nov 2023	Symptoms of upper respiratory tract infection (URTI).
6 Nov 2023	Developed rash over face and scalp after taking amoxicillin-clavulanate and panadeine.
11 Nov 2023	Admitted to Hospital Y.
15 Nov 2023	Blood culture and wound swab grew MRSA; Eye swab grew MRSA and other species.
16 Nov 2023	Started Levofloxacin.
17 Nov 2023	Started Vancomycin.
20 Nov 2023	First dose of intravenous immunoglobulin (IVIG).
21 Nov 2023	Second dose of IVIG; Started Ciprofloxacin; Chest X-ray showed no evidence of consolidation.
Date not specified	Developed Toxic epidermal necrolysis (TEN).

2. Clinical Problem List
Status	Clinical Issue

Active	Toxic epidermal necrolysis (TEN)
Active	Upper respiratory tract infection (URTI)
Active	Rash over face and scalp
Active	Mucosal eruptions
Active	MRSA infection
Chronic	Coronary artery disease (CAD)
Chronic	Atrial fibrillation on Novel Oral Anticoagulants (NOAC)
Chronic	Lower urinary tract symptoms (LUTS)
Chronic	Raised prostate specific antigen (PSA)

3. Potential Life-Threatening Complications
- Sepsis due to MRSA infection.
- Acute kidney injury secondary to sepsis or medication.
- Hemodynamic instability due to coronary artery disease and atrial fibrillation exacerbated by infection and stress of illness.
- Widespread skin loss and secondary infection from TEN.

4. Clinical Findings

Organ System	Physical Examination Findings
Integumentary	Diffuse erythema with hemorrhagic crusts, epidermal detachment.
Eyes	Mucosal eruptions over eyes.
Oral	Mucosal eruptions over lips and mouth.
Respiratory	No current findings from CXR.
Cardiovascular	No new findings specified.
Genitourinary	History of LUTS and raised PSA.

5. Working Diagnoses
- Toxic epidermal necrolysis (TEN) secondary to drug reaction.
- MRSA bacteremia and localized infections (skin, eye, wound).
- Upper respiratory tract infection (URTI).
- Chronic conditions: Coronary artery disease, atrial fibrillation, LUTS.

6. Relevant Investigations

Investigation	Justification
Repeat blood culture	To confirm clearance of MRSA bacteremia.
Renal function tests	To monitor for signs of acute kidney injury.
Liver function tests	To assess for worsening liver injury.
Repeat wound swab	To assess for current infection status.
Echocardiogram	To evaluate cardiac function due to underlying CAD and atrial fibrillation.

7. Management Plan
- Continue broad-spectrum antibiotics (Vancomycin and Ciprofloxacin) while awaiting culture results.
- Complete the course of IVIG for TEN.
- Monitor vital signs closely and manage hemodynamics.
- Supportive care for TEN, including wound care and pain management.
- Isolate patient due to MRSA and implement appropriate infection control measures.
- Consult dermatology for TEN management and infectious disease for MRSA management.
- Renal and liver function tests to be done regularly.
- Review and manage underlying chronic conditions.

8. Executive Summary
The patient, an 82-year-old male with a history of coronary artery disease and atrial fibrillation, was admitted for toxic epidermal necrolysis (TEN) and MRSA infections. The TEN is likely drug-induced, with recent URTI treatment being the precipitating factor. Immediate life-threatening complications include sepsis and acute kidney injury. Management includes completing the course of IVIG for TEN and broad-spectrum antibiotics for MRSA, while monitoring organ functions and maintaining supportive care. Coordination between dermatology and infectious disease specialists is crucial for the patient's recovery.

cGPT-4

1. Chronological Events:
Date	Event
Early Nov	Upper respiratory tract infection (URTI) symptoms presented
6 Nov 2023	Rash developed over face and scalp after amoxicillin-clavulanate and panadeine
11 Nov 2023	Admission to Hospital Y
15 Nov 2023	Blood culture grew MRSA; Wound swab grew MRSA, Peptoniphilus species, Elizabethkingia miricola; Eye swab grew MRSA, Chryseobacterium arthrosphaerae, Eikenella corrodens
16 Nov 2023	Levofloxacin started
17 Nov 2023	Ciprofloxacin and Vancomycin started
20 Nov 2023	Intravenous immunoglobulin (IVIG) started
21 Nov 2023	Epidermal detachment noticed; No evidence of consolidation on CXR; Ciprofloxacin continued

2. Clinical Problem List:
| Status| Clinical Issue|

Chronic	Coronary artery disease
Chronic	Atrial fibrillation on NOACs
Chronic	Lower urinary tract symptoms
Chronic	History of raised PSA without malignancy
Active	Toxic epidermal necrolysis (TEN)
Active	Upper respiratory tract infection (URTI)
Active	Rash development post antibiotics
Active	Mucosal eruptions
Active	Worsening of liver function (ALT)
Active	Infection with multi-drug resistant organisms

3. Potential Life-Threatening Complications:
- Septicemia due to MRSA infection, which can lead to septic shock.
- Acute liver injury due to worsening ALT levels.
- Acute renal failure, although not present now, could be a complication with ongoing infection and TEN.
- TEN itself is a life-threatening dermatological condition with potential complications including secondary infections, fluid loss, and multi-organ failure.

4. Clinical Findings:

Organ System	Anticipated Findings
Integumentary	Diffuse erythema, hemorrhagic crusts, epidermal detachment
Ocular	Mucosal eruptions over eyes
Oral	Mucosal eruptions over lips and mouth
Respiratory	No evidence of consolidation on CXR (21 Nov 2023)
Hepatic	Worsening ALT levels indicating liver involvement

5. Working Diagnoses:
- Toxic Epidermal Necrolysis (TEN) likely drug-induced.
- MRSA septicemia.
- Multi-drug resistant organism-related skin, eye, and wound infections.
- Liver function abnormalities secondary to medication or infection.

6. Relevant Investigations:

Investigation	Justification
Blood cultures	To confirm clearance of MRSA and detect any new bacteremia
Wound swabs	To monitor the effectiveness of antibiotic treatment against identified organisms
Eye swabs	To assess the resolution or progression of the ocular infection

| Liver function tests| To evaluate the extent of liver involvement and monitor for drug hepatotoxicity |
| Renal function tests| To monitor renal function given the potential for renal involvement in TEN and sepsis |

7. Management Plan:
- Continue monitoring and treating for MRSA with appropriate antibiotics, considering any potential antibiotic resistance.
- Monitor liver and renal function closely given the current abnormalities and the risk of further deterioration due to ongoing infection or drug effects.
- Continue supportive care for TEN, including wound care, fluid management, and pain control. Depending on the patient's response to IVIG, consider repeating the dose or adjunctive therapies.
- Engage in daily reviews by nursing staff and weekly multidisciplinary team meetings to assess the patient's progress and adjust the management plan accordingly [[1]](https://poe.com/citation?message_id=99121429675&citation=1).
- Implement strategies for the prevention of secondary infections and manage any complications promptly.

8. Executive Summary:
The patient, an 82-year-old male with a history of coronary artery disease, atrial fibrillation, and prior elevated PSA levels, was admitted for Toxic Epidermal Necrolysis (TEN) and complications from an upper respiratory tract infection. The treatment plan includes close monitoring of his MRSA infection and liver function, continued antibiotic therapy, supportive care for TEN, and multidisciplinary reviews. Given the complexities of his case, a thorough and adaptive management strategy is crucial.

Gemini Pro

1. Chronological Events:

Date	Clinical Issue
6 November 2023	Rash over face and scalp
11-21 November 2023	Hospital admission for Toxic epidermal necrolysis (TEN)
15 November 2023	Blood culture grew Methicillin-resistant Staphylococcus aureus (MRSA)
15 November 2023	Wound swab grew MRSA, Peptoniphilus species, Elizabethkingia miricola
15 November 2023	Eye swab grew MRSA, Chryseobacterium arthrosphaerae, Eikenella corrodens
16-17 November 2023	Levofloxacin administered
17 November 2023	Vancomycin administered

20-22 November 2023	Intravenous immunoglobulin (IVIG) administered
21 November 2023	Ciprofloxacin administered
21 November 2023	Chest X-ray (CXR) showed no evidence of consolidation

2. Clinical Problem List:

Active	Chronic
Toxic epidermal necrolysis (TEN)	Coronary artery disease
Methicillin-resistant Staphylococcus aureus (MRSA) bacteremia	Atrial fibrillation
Polymicrobial wound infection	Lower urinary tract symptoms
Worsening Alanine aminotransferase (ALT)	Raised prostate-specific antigen (PSA)

3. Potential Life-Threatening Complications:
- Sepsis
- Multi-organ failure
- Respiratory failure
- Fluid and electrolyte imbalances
- Arrhythmias
- Acute kidney injury

4. Clinical Findings:

Organ System	Anticipated Physical Examination Findings
General	Afebrile
Respiratory	No evidence of consolidation on CXR
Cardiovascular	No findings reported
Gastrointestinal	No findings reported
Neurological	No findings reported
Musculoskeletal	No findings reported
Integumentary	Diffuse erythema with hemorrhagic crusts over lips, perioral and periorbital area. Dusky targetoid patches over neck and chest, thighs. Epidermal detachment over abdomen, axillae, inner thighs, buttocks, most areas over back.

5. Working Diagnoses:
- Toxic epidermal necrolysis (TEN)
- Methicillin-resistant Staphylococcus aureus (MRSA) bacteremia
- Polymicrobial wound infection

6. Relevant Investigations:

Investigation	Justification
Blood culture	Monitor for persistent or recurrent bacteremia
Wound swab	Monitor for changes in wound flora and antimicrobial susceptibility

Urine culture	Assess for urinary tract infection
Serum electrolytes	Monitor for fluid and electrolyte imbalances
Renal function tests	Monitor for acute kidney injury
Liver function tests	Monitor for worsening liver function
Electrocardiogram (ECG)	Monitor for arrhythmias

7. Management Plan:
- Continue IVIG for TEN
- Continue vancomycin for MRSA bacteremia
- Initiate appropriate antimicrobial therapy for polymicrobial wound infection based on culture and susceptibility results
- Monitor for and manage complications such as sepsis, multi-organ failure, respiratory failure, fluid and electrolyte imbalances, arrhythmias, and acute kidney injury
- Provide supportive care including fluid resuscitation, electrolyte replacement, and nutritional support

8. Executive Summary:
The patient is an 82-year-old male with a history of coronary artery disease, atrial fibrillation, and lower urinary tract symptoms who presented with TEN and was found to have MRSA bacteremia and polymicrobial wound infection. He is currently receiving IVIG, vancomycin, and appropriate antimicrobial therapy for the wound infection. The patient's clinical status should be closely monitored for complications, and supportive care should be provided as needed.

Claude 2

1. Chronological Events
Date	Event
Prior to admission	Coronary artery disease, Atrial fibrillation on NOAC, Lower urinary tract symptoms, Raised PSA
6 November 2023	Developed rash after taking amoxicillin-clavulanate and panadeine
11 November 2023	Admitted to Hospital Y
15 November 2023	Blood culture grew MRSA
17 November 2023	Started on vancomycin
20-22 November 2023	Received IVIG
21 November 2023	Started on ciprofloxacin

2. Clinical Problem List
Active Problems	Chronic Problems
Toxic epidermal necrolysis	Coronary artery disease

MRSA bacteremia	Atrial fibrillation
ALT elevation	Lower urinary tract symptoms
Multi-drug resistant infections	Raised PSA

3. Potential Life-Threatening Complications
- Sepsis/septic shock from MRSA bacteremia
- Acute kidney injury from antibiotic use
- Superinfection of denuded skin

4. Clinical Findings

Organ System	Expected Findings
Integumentary	Diffuse erythema, targetoid lesions, epidermal detachment
HEENT	Crusting over lips, erosions of mucosa
Respiratory	Normal breath sounds
Cardiovascular	Tachycardia
Abdominal	Normal
Neurological	Normal

5. Working Diagnoses
- Toxic epidermal necrolysis
- MRSA bacteremia
- Multi-drug resistant infections
- Acute kidney injury
- Sepsis

6. Relevant Investigations

Investigation	Justification
Repeat blood cultures	Document clearance of bacteremia
Wound cultures	Identify infecting organisms for wound infection
Serum creatinine, electrolytes	Monitor kidney function
Liver function tests	Monitor for drug toxicity
Procalcitonin level	Marker for sepsis

7. Management Plan
- Admit to isolation room
- Continue IV vancomycin to treat MRSA
- Topical antiseptics for wound care
- Strict hand hygiene for staff
- Monitor kidney function, electrolytes
- Sepsis management if clinically indicated
- Ophthalmology review for eye care

- Dermatology review for skin care
- Avoid nephrotoxic drugs if possible
- Nutrition review for high calorie dietary support

8. Executive Summary

This 82 year old man with multiple comorbidities developed toxic epidermal necrolysis after taking antibiotics for a URI, now complicated by MRSA bacteremia and multi-drug resistant infections. He requires isolation, continued vancomycin, meticulous wound care, kidney function monitoring, and specialist input from ophthalmology and dermatology to manage this critical illness.

33. Clinical scenario 33

GPT-4.0

1. Chronological Events

Year	Event
1998	Diagnosis of autoimmune hemolytic anemia; splenectomy performed.
2004	History of encephalitis.
2019	Vaccination with Pneumovax 23 (Pneumococcal Polysaccharide Vaccine, PPSV23).
2022	Vaccination with Bexsero (meningococcal group B vaccine) and MenACWY Vaccine (Meningococcal group A, C, W-135 and Y conjugate vaccine).
Nov 9-12, 2023	Travelled to Bangkok.
Nov 21, 2023	Onset of fever with chills, myalgia, malaise; bifrontal headache and mild neck stiffness. Blood culture drawn showing Streptococcus pneumoniae. Started on antimicrobials: Piperacillin-tazobactam, ceftriaxone, and doxycycline.
Nov 22, 2023	Blood cultures pending; other investigations pending.
Date of Admission	Admitted for fever; lumbar puncture performed.

2. Clinical Problem List

Status	Clinical Issues
Active	Fever with chills, myalgia, malaise
Active	Bifrontal headache and mild neck stiffness
Active	Streptococcus pneumoniae bacteremia
Chronic	Hypertension
Chronic	Hyperlipidemia
Chronic	Autoimmune hemolytic anemia (historical, with splenectomy)
Chronic	Past encephalitis
Chronic	History of right tibia fracture and deep vein thrombosis

3. Potential Life-Threatening Complications

- Septicemia and septic shock from Streptococcus pneumoniae bacteremia.
- Potential for meningitis, given the symptoms of headache and neck stiffness, and the patient's history of splenectomy (immunocompromised status).
- Recurrence of deep vein thrombosis due to history and current immobilization.

4. Clinical Findings

Organ System	Anticipated Findings
General	Fever, malaise
Neurological	Headache, neck stiffness, unremarkable neurology exam
Respiratory	Clear chest, no dyspnea
Cardiovascular	Heart sound with no murmur
Abdominal	Soft abdomen
Musculoskeletal	No joint pain, calves soft

5. Working Diagnoses
- Streptococcus pneumoniae bacteremia
- Possible meningitis
- Fever of unknown origin (pending further investigation)

6. Relevant Investigations

Investigation	Justification
Blood cultures	Confirmed Streptococcus pneumoniae, need to monitor for resistance and efficacy of treatment
Lumbar puncture	To assess for meningitis due to neck stiffness and headache
Chest X-ray	To rule out pneumonia, which can be a source of bacteremia
Full blood count	To monitor ANC and ALC, especially given the immunocompromised state post-splenectomy
CRP	To monitor inflammation and response to treatment
Liver function tests	Baseline and to monitor for drug-induced hepatotoxicity
Repeat cultures	To ensure eradication of the bacteremia and to monitor for other potential infections

7. Management Plan
- Continue intravenous antibiotics as per current regimen and adjust based on culture sensitivities.
- Monitor hemodynamics and organ function closely for signs of septic shock.
- Supportive care for fever and hydration.
- Consider imaging studies if any new symptoms arise or if there is no improvement, to rule out other potential sources of infection.
- Prophylactic anticoagulation may be considered due to past history of DVT.
- Neurological monitoring to assess for progression to meningitis.

- Follow up on all pending cultures and adjust antibiotic therapy accordingly.
- Patient education on signs of worsening infection and the importance of compliance with the treatment regimen.

8. Executive Summary
A 40-year-old male with a significant medical history including a splenectomy is admitted with fever and symptoms suggestive of Streptococcus pneumoniae bacteremia, with potential meningitis. He is currently on a broad-spectrum antibiotic regimen. Immediate focus is on monitoring for septic shock and neurological deterioration, with a comprehensive investigative approach to confirm diagnosis and monitor treatment efficacy. The management plan involves continued antibiotic therapy, close monitoring, and supportive care, with adjustments made as further culture results become available.

cGPT-4

1. Chronological Events:

Date	Event
1998	Diagnosed with autoimmune hemolytic anemia, splenectomy performed
2004	History of encephalitis
2019	Received Pneumovax 23 vaccine
2022	Received Bexsero and MenACWY Vaccines
9-12 November 2023	Travelled to Bangkok
21 November 2023	Onset of fever, chills, myalgia, malaise, bifrontal headache, and mild neck stiffness
21 November 2023	Blood culture drawn, started on Piperacillin-tazobactam, ceftriaxone, and doxycycline
22 November 2023	3 sets of blood culture pending; sputum urine culture pending
23 November 2023	Doxycycline discontinued

2. Clinical Problem List:

Status	Clinical Issues
Active	Fever with chills, myalgia, malaise, headache, and neck stiffness
Active	Streptococcus pneumoniae bacteremia (penicillin MIC 0.38 ug/ml)
Chronic	Hypertension
Chronic	Hyperlipidemia
Chronic	Past history of splenectomy

3. Potential Life-Threatening Complications:
- Sepsis from Streptococcus pneumoniae bacteremia which can lead to septic shock.
- Potential for meningitis given the symptoms of headache and neck stiffness.

4. Clinical Findings:

Organ System	Anticipated Findings
General	Fever, malaise
Neurologic	Headache, mild neck stiffness
Respiratory	Clear lungs on auscultation
Cardiovascular	Normal heart sounds, no murmurs
Abdomen	Soft, no splenomegaly due to past splenectomy
Musculoskeletal	No joint pain, calves soft

5. Working Diagnoses:
- Bacteremia due to Streptococcus pneumoniae.
- Possible early meningitis given the patient's symptoms and history of encephalitis.

6. Relevant Investigations:

Investigation	Justification
Blood cultures	To identify causative organism and its antibiotic sensitivities.
Lumbar puncture	To assess for possible meningitis, given the patient's neck stiffness and headache.
Complete blood count	To monitor for signs of infection or other hematologic abnormalities.
CRP level	As an indicator of inflammation or infection.
Liver function tests	To monitor organ function which may be affected by sepsis or medications.

7. Management Plan:
- Continue antibiotic therapy with ceftriaxone pending culture results and sensitivities.
- Maintain hydration and support blood pressure if signs of sepsis progress.
- Monitor vital signs and laboratory values closely, adjusting treatment as necessary.
- Daily review by a nurse or similar, with consideration for an oral switch of antibiotics when appropriate.
- Weekly review in a multidisciplinary team meeting and regular review by a specialist nurse or doctor.
- Prepare for a possible lumbar puncture based on the patient's neurological symptoms and past medical history.

8. Executive Summary:
The patient, a 40-year-old male with a history of splenectomy and recent travel, presented with fever and signs suggestive of bacteremia, possibly complicated by meningitis. Blood cultures have identified Streptococcus pneumoniae, and the patient is on appropriate antibiotics. Close monitoring and daily reviews by healthcare

professionals are essential, with an interdisciplinary team approach for ongoing management. Further investigations including a lumbar puncture may be warranted given the neurological symptoms.

Gemini Pro

1. Chronological Events:

Date	Clinical Issue
9-12 November 2023	Travel to Bangkok
21 November 2023	Fever, chills, myalgia, malaise, bifrontal headache, mild neck stiffness
21 November 2023	Blood culture drawn, Streptococcus pneumoniae isolated
21 November 2023	Piperacillin-tazobactam and ceftriaxone initiated
21 November 2023	Doxycycline initiated
22 November 2023	Additional blood cultures drawn
22 November 2023	Lumbar puncture performed

2. Clinical Problem List:

Active	Chronic
Streptococcus pneumoniae bacteremia	Hypertension
Meningitis	Hyperlipidemia
Encephalitis	Autoimmune hemolytic anemia
Trauma-related R tibia fracture	Deep vein thrombosis

3. Potential Life-Threatening Complications:
- Septic shock
- Meningoencephalitis
- Acute respiratory distress syndrome (ARDS)
- Multi-organ failure

4. Clinical Findings:

Organ System	Anticipated Findings
General	Fever, chills, malaise
Neurological	Headache, neck stiffness, photophobia, altered mental status
Respiratory	Tachypnea, hypoxia
Cardiovascular	Tachycardia, hypotension
Gastrointestinal	Nausea, vomiting, abdominal pain
Musculoskeletal	Myalgia, arthralgia
Integumentary	Rash, petechiae, ecchymosis

5. Working Diagnoses:
- Streptococcus pneumoniae bacteremia
- Meningitis
- Encephalitis

6. Relevant Investigations:

Investigation	Justification
Blood cultures	To confirm the presence of Streptococcus pneumoniae and determine antimicrobial susceptibility
Cerebrospinal fluid (CSF) analysis	To assess for evidence of meningitis or encephalitis, including cell count, glucose, protein, and Gram stain and culture
Chest X-ray	To evaluate for pulmonary infiltrates or other abnormalities
Computed tomography (CT) scan of the head	To assess for structural abnormalities or evidence of encephalitis
Electrocardiogram (ECG)	To evaluate for arrhythmias or other cardiac abnormalities
Complete blood count (CBC)	To assess for anemia, leukocytosis, or thrombocytopenia
Comprehensive metabolic panel (CMP)	To assess for electrolyte imbalances, renal function, and liver function

7. Management Plan:
- Antimicrobial Therapy:
- Continue ceftriaxone 2 g IV every 12 hours until CSF culture results are available.
- If CSF culture is positive for Streptococcus pneumoniae, continue ceftriaxone for a total of 14 days.
- If CSF culture is negative, consider discontinuing ceftriaxone after 7 days.
- Supportive Care:
- Maintain adequate hydration with intravenous fluids.
- Monitor vital signs and oxygen saturation closely.
- Provide antipyretics and analgesics for fever and pain.
- Administer corticosteroids if there is evidence of increased intracranial pressure.
- Neurological Monitoring:
- Monitor neurological status closely for signs of worsening meningitis or encephalitis.
- Consider repeat lumbar puncture if there is clinical deterioration.
- Infection Control:
- Implement standard precautions to prevent the spread of infection.
- Educate the patient and family about the importance of hand hygiene and respiratory hygiene.

8. Executive Summary:

- The patient is a 40-year-old male with a history of hypertension, hyperlipidemia, autoimmune hemolytic anemia, encephalitis, and trauma-related R tibia fracture who presented with fever, chills, myalgia, malaise, bifrontal headache, and mild neck stiffness.
- Blood cultures grew Streptococcus pneumoniae, and a lumbar puncture was performed.
- The patient is currently being treated with ceftriaxone and supportive care.
- The management plan includes continuing antimicrobial therapy, providing supportive care, monitoring neurological status, and implementing infection control measures.

Claude 2

1. Chronological Events

Date	Event
9 to 12 November 2023	Travelled to Bangkok
21 November 2023	Fever with chills, myalgia, malaise, bifrontal headache, mild neck stiffness
21 November 2023	Blood culture: Streptococcus pneumoniae
21 November 2023	Started piperacillin-tazobactam and doxycycline
22 November 2023	Changed to ceftriaxone
22 November 2023	Lumbar puncture done

2. Clinical Problem List

Clinical Problem	Status
Fever	Active
Headache	Active
Neck stiffness	Active
S. pneumoniae bacteremia	Active
Hypertension	Chronic
Hyperlipidemia	Chronic
Past autoimmune hemolytic anemia	Chronic
Past encephalitis	Chronic
Past DVT	Chronic

3. Potential Life-Threatening Complication
- Meningitis
- Brain abscess
- Endocarditis
- Septic arthritis
- Septic shock

4. Clinical Findings

System	Expected Findings
Neurological	Headache, neck stiffness, focal neurological deficits if meningitis
Cardiovascular	Tachycardia, hypotension if septic shock
Respiratory	Crackles if pneumonia
Musculoskeletal	Warm, swollen, tender joints if septic arthritis
Skin	Petechiae if DIC from sepsis

5. Working Diagnoses
- S. pneumoniae bacteremia
- S. pneumoniae meningitis
- Complications of bacteremia: endocarditis, septic arthritis

6. Relevant Investigations

Investigation	Justification
LP cell count, culture, PCR	Diagnose meningitis
Blood cultures	Document bacteremia
Echo	Assess for endocarditis
Joint aspiration	Diagnose septic arthritis
CT brain	Assess for complications e.g. abscess

7. Management Plan
- IV antibiotics: ceftriaxone or cefotaxime +/- vancomycin if high risk of penicillin resistance
- Dexamethasone if meningitis
- Aggressive hydration
- Monitor for complications of bacteremia
- Infectious disease consult
- Repeat blood cultures to document clearance
- Assess need for valve surgery if endocarditis
- Physical therapy if joints involved

8. Executive Summary
This 40-year-old male with S. pneumoniae bacteremia likely has bacterial meningitis given headache and neck stiffness. He is at risk for serious complications like endocarditis, septic arthritis, and brain abscess. Recommend LP, blood cultures, echo, and CT brain to assess for complications. IV ceftriaxone is appropriate. Dexamethasone should be given if meningitis confirmed. Infectious disease consult recommended to manage complications.

34. Clinical scenario 34

GPT-4.0

1. Chronological Events

Date	Event
2 November 2023	MRI hand with contrast: synovial thickening, suggestive of infective cause vs inflammatory arthropathy.
17 November 2023	Admission for tenosynovitis; Started on Amoxicillin-clavulanate.
20 November 2023	Synovectomy of wrist and tenonectomy of hand; Noted significant synovitis and tenosynovitis, yellowish fluid in tendon sheath.
27 November 2023	Follow-up Synovectomy: mild residual tenosynovitis, significant along index finger towards radial bursa.
Undated	Bacterial culture: Growth of Methicillin-sensitive Staphylococcus aureus (MSSA).
Undated	Investigation results: WBC 8.50 x10^9/L, ANC 5.62 x10^9/L, Renal function normal, deranged liver function. Histology and AFB culture pending.

2. Clinical Problem List

Status	Clinical Issue
Chronic	Hypertension
Chronic	Minor coronary artery disease
Chronic	Smoking
Chronic	Heavy alcohol consumption
Active	Tenosynovitis
Active	Synovial thickening
Active	Deranged liver function

3. Potential Life-Threatening Complications
- Septic arthritis leading to systemic infection and sepsis.
- Liver failure due to deranged liver function, potentially exacerbated by heavy alcohol use.

4. Clinical Findings

Organ System	Anticipated Findings
Musculoskeletal	Swelling, tenderness over second MCP, decreased range of motion
Integumentary	Possible erythema and warmth over affected areas
Hepatic	Jaundice, hepatomegaly if liver function is compromised

5. Working Diagnoses
- Infective tenosynovitis due to MSSA
- Possible inflammatory arthropathy
- Liver pathology secondary to alcohol use and infection

6. Relevant Investigations

Investigation	Justification
Blood cultures	To confirm systemic infection and antibiotic sensitivity
Liver function tests	To assess the extent of liver damage and monitor progression
ESR/CRP	To evaluate the inflammatory status
Repeat MRI with contrast	To assess progression/resolution of synovial thickening
Hand X-ray	To rule out osteomyelitis or bone involvement
Histology of intraoperative specimens	To confirm diagnosis and rule out malignancy or other pathology
AFB culture	To rule out mycobacterial infection as a cause of tenosynovitis

7. Management Plan
- Continue broad-spectrum antibiotics until culture sensitivities are known, then de-escalate to targeted therapy.
- Monitor liver function closely; consider abstinence from alcohol and hepatoprotective agents.
- Regular hand physiotherapy post-surgery to preserve function.
- Address cardiovascular risk factors (hypertension, smoking, heavy drinking) with lifestyle modifications and pharmacotherapy.
- Close follow-up to monitor resolution of tenosynovitis and liver function.
- Consider rheumatology referral if inflammatory arthropathy is suspected.

8. Executive Summary
A 59-year-old male with a history of hypertension, minor coronary artery disease, smoking, and heavy alcohol use was admitted with tenosynovitis, unresponsive to initial antibiotic treatment. Investigations revealed MSSA infection and deranged liver function. Urgent concerns include potential sepsis and liver pathology. The management plan includes targeted antibiotic therapy following culture results, monitoring and support for liver function, and addressing chronic cardiovascular risk factors. Further investigations and follow-ups are necessary to guide treatment adjustments and monitor the patient's progress.

cGPT-4

1. Chronological Events:

Date	Event
2 Nov 2023	MRI of the hand showing synovial thickening
17 Nov 2023	Admission for tenosynovitis; started on Amoxicillin-clavulanate
20 Nov 2023	Synovectomy of wrist and tenonectomy of hand

27 Nov 2023	Follow-up synovectomy; mild residual tenosynovitis noted

2. Clinical Problem List:

Status	Clinical Issues
Active	Tenosynovitis
Chronic	Hypertension
Chronic	Minor coronary artery disease
Chronic	Smoking
Chronic	Heavy drinking

3. Potential Life-Threatening Complications:
- Septicemia due to unresolved infective tenosynovitis
- Acute myocardial infarction given underlying coronary artery disease

4. Clinical Findings:

Organ System	Anticipated Findings
Musculoskeletal	Swelling over second MCP joint, synovitis
Cardiovascular	Signs of hypertension, possible CAD symptoms
Respiratory	Signs consistent with smoking history
Integumentary	Possible findings related to heavy alcohol use

5. Working Diagnoses:
- Infective tenosynovitis
- Hypertension
- Minor coronary artery disease

6. Relevant Investigations:

Investigation	Justification
Blood cultures	Identify presence of systemic infection
Histology	Determine the nature of synovitis
Acid fast bacilli (AFB) culture	Rule out mycobacterial infection
Liver function tests	Assess for liver damage due to heavy drinking
Echocardiogram	Evaluate cardiac function due to CAD

7. Management Plan:
- Continue antibiotic therapy as per culture sensitivity results.
- Monitor WBC and ANC levels for signs of infection control or progression.
- Evaluate and address liver function abnormalities.
- Optimize management of hypertension and monitor for cardiac complications.
- Smoking cessation and alcohol use reduction counseling.

- Consider physical therapy post-recovery for hand functionality.
- Regular follow-up for chronic conditions.

8. Executive Summary:
The patient is a 59-year-old male with active tenosynovitis and chronic conditions including hypertension and coronary artery disease. Management includes ongoing antibiotic therapy, monitoring of blood counts and liver function, and addressing lifestyle factors such as smoking and heavy drinking. Further histological and AFB investigations are pending, which will guide the treatment plan. Regular follow-up is crucial for managing chronic conditions and preventing potential life-threatening complications.

Gemini Pro

1. Chronological Events:

Date	Event
2 Months Prior to Admission	Increasing swelling over the second metacarpophalangeal joint (MCP) dorsum
November 2, 2023	Magnetic resonance imaging (MRI) hand with contrast
November 17, 2023	Admission for tenosynovitis
November 20, 2023	Synovectomy of wrist and tenonectomy of hand at Hospital D
November 27, 2023	Synovectomy at Hospital D

2. Clinical Problem List:

Active	Chronic
Tenosynovitis	Hypertension
Minor coronary artery disease	
Deranged liver function	

3. Potential Life-Threatening Complications:
* Septic arthritis
* Osteomyelitis
* Endocarditis
* Meningitis

4. Clinical Findings:

Organ System	Anticipated Findings
Musculoskeletal	Swelling, erythema, tenderness over the second MCP joint
Cardiovascular	Irregular heart rate, murmurs
Respiratory	Tachypnea, hypoxia

| Gastrointestinal | Nausea, vomiting, abdominal pain |
| Neurological | Headache, confusion, seizures |

5. Working Diagnoses:
* Septic tenosynovitis
* Methicillin-sensitive Staphylococcus aureus (MSSA) infection

6. Relevant Investigations:
Investigation	Justification
Blood cultures	To identify the causative organism and determine antibiotic susceptibility
Wound culture	To confirm the presence of MSSA and assess for antibiotic susceptibility
Computed tomography (CT) scan of the hand	To evaluate the extent of the infection and assess for complications
Echocardiogram	To assess for the presence of endocarditis

7. Management Plan:
* Intravenous antibiotics (e.g., vancomycin and rifampin)
* Surgical debridement of the infected tissue
* Immobilization of the affected joint
* Pain management
* Monitoring for complications

8. Executive Summary:
The patient is a 59-year-old male with a history of hypertension and minor coronary artery disease who presented with tenosynovitis of the second MCP joint. MRI revealed synovial thickening suggestive of an infective cause. Synovectomy and tenonectomy were performed, and bacterial culture grew MSSA. The patient is currently receiving intravenous antibiotics and is being monitored for complications. The working diagnosis is septic tenosynovitis due to MSSA.

Claude 2

1. Chronological Events
Date	Event
2 months prior to admission	Onset of swelling over 2nd MCP joint dorsum
1 week prior to admission	Seen by GP, given Augmentin without improvement
2 Nov 2023	MRI hand showed synovitis suggestive of infection or inflammatory arthritis
17 Nov 2023	Admitted for tenosynovitis

20 Nov 2023	Synovectomy and tenonectomy done
Intraoperative	MSSA isolated from tendon sheath
27 Nov 2023	Repeat synovectomy done

2. Clinical Problem List

Problem	Active/Chronic
Tenosynovitis right hand	Active
MSSA infection	Active
Hypertension	Chronic
Coronary artery disease	Chronic

3. Potential Life-Threatening Complications
- Septicemia from untreated MSSA infection
- Metastatic infections like infective endocarditis, osteomyelitis, septic arthritis

4. Anticipated Physical Examination Findings

System	Expected Findings
Musculoskeletal	Swelling, tenderness over right hand and wrist
Cardiovascular	Tachycardia, new murmurs
Respiratory	Tachypnea
Neurological	Altered mental status

5. Working Diagnoses
- Infective tenosynovitis right hand
- MSSA bacteremia
- Possible infective endocarditis

6. Relevant Investigations

Investigation	Justification
Blood cultures	Confirm bacteremia and determine antibiotic susceptibilities
Echocardiogram	Assess for infective endocarditis
MRI spine	Assess for epidural abscess if back pain or neurological deficits
Bone scan	Assess for osteomyelitis if localized bone pain

7. Management Plan
- IV antibiotics: Vancomycin + Rifampicin to cover MSSA
- Repeat blood cultures to document clearance
- Echocardiogram to rule out endocarditis
- MRI spine if any back pain or neurological signs to assess for epidural abscess
- Assess need for surgical washout/debridement of residual infected tissue

- Address modifiable risk factors: smoking cessation, alcohol cessation
- Follow-up blood tests for drug levels, counts, liver function

8. Executive Summary

Mr X, a 59 year old smoker and heavy drinker, underwent drainage of MSSA hand infection but requires ongoing IV antibiotics and investigation for metastatic foci to rule out further complications.

35. Clinical scenario 35

GPT-4.0

1. Chronological Events:

Date	Event
February 2009	Exploration and decompression of spinal canal at L4/L5
June 2017	Total hip replacement for right osteoarthritis at Hospital B
October 10, 2023	Wide excision and vascular reconstruction for left medial thigh sarcoma
November 6, 2023	Urgent CT scan due to persistent unhealthy, purulent wound bed; transfer back to hospital
November 6, 2023	CT findings: Fluid collection in the anterior aspect of upper thigh
November 7, 2023	Intra-operative findings: Skin necrosis, turbid fluid, unhealthy rectus femoris; vascular graft assessment and muscular flap coverage

2. Clinical Problem List:

Active Issues	Chronic Issues
Left medial thigh sarcoma post wide excision	Osteoporosis
Post-surgical wound infection with purulent drainage	History of spinal stenosis L4/5
Impaired fasting glucose	Hypertension
Renal impairment (elevated serum creatinine)	History of right hip osteoarthritis with total hip replacement

3. Potential Life-Threatening Complications:
- Sepsis from wound infection and potential bacteremia
- Acute renal failure due to infection and pre-existing renal impairment
- Compromised vascular graft integrity leading to hemorrhage or graft failure

4. Clinical Findings:

Organ System	Anticipated Findings
Integumentary	Unhealthy, purulent wound bed; skin necrosis at left thigh

Cardiovascular	Strongly pulsatile vascular graft; possible compromised circulation
Musculoskeletal	Friable medial edge of rectus femoris
Renal	Elevated serum creatinine indicating renal impairment

5. Working Diagnoses:
- Postoperative wound infection with necrotizing fasciitis
- Acute renal impairment secondary to sepsis
- Compromised vascular graft at the site of previous reconstruction

6. Relevant Investigations:

Investigation	Justification
Repeat blood cultures	To identify current bloodstream infection and guide antimicrobial therapy
Wound cultures	To tailor antibiotic treatment based on the causative organisms
Renal function tests (e.g., eGFR, electrolytes)	To monitor renal function and detect acute kidney injury
Complete blood count with differential	To assess for anemia and immune response
Imaging of the left thigh (e.g., MRI)	To assess the extent of soft tissue infection and vascular graft status

7. Management Plan:
1. Continue broad-spectrum antibiotics, consider adjusting based on culture results and sensitivities.
2. Surgical debridement of necrotic tissue and assessment of the vascular graft integrity.
3. Supportive care for renal function, including fluid management and monitoring of output and electrolytes.
4. Tight glycemic control considering the impaired fasting glucose.
5. Vascular surgery consult for the assessment of the muscular flap coverage and vascular graft.
6. Multidisciplinary team involvement including infectious disease specialists, nephrologists, and wound care specialists.

8. Executive Summary:
An 83-year-old female with a history of osteoporosis, spinal stenosis, hip replacement, hypertension, and impaired fasting glucose presented with complications following wide excision and vascular reconstruction for left medial thigh sarcoma. The patient has an active post-surgical wound infection with significant systemic implications, including potential sepsis and acute renal impairment. Management includes broad-spectrum antibiotic therapy, surgical intervention, and supportive care with a focus on maintaining renal function and vascular graft integrity. A multidisciplinary approach is essential for optimal outcomes.

cGPT-4

1. Chronological Events:

Date	Event
Feb 2009	Exploration and decompression of spinal canal L4/5.
Jun 2017	Total hip replacement for right osteoarthritis of hip.
10 Oct 2023	Wide excision and vascular reconstruction for left medial thigh sarcoma.
6 Nov 2023	CT scan reveals fluid collection in upper thigh; transferred back to hospital.
7 Nov 2023	Intra-operative findings: skin necrosis, turbid fluid, unhealthy medial edge of rectus femoris. Vascular graft covered with muscular flap.

2. Clinical Problem List:

Status	Clinical Issue
Active	Post-operative wound infection with purulent discharge.
Active	Impaired fasting glucose.
Chronic	Osteoporosis.
Chronic	History of spinal stenosis L4/5.
Chronic	Right osteoarthritis of hip (post total hip replacement).
Chronic	Hypertension.

3. Potential Life-Threatening Complications:
 - Sepsis due to wound infection.
 - Graft infection or failure leading to limb ischemia.
 - Hemorrhage or vascular compromise from the vascular reconstruction site.

4. Clinical Findings:

Organ System	Anticipated Findings
Musculoskeletal	Tenderness, warmth, and possible signs of inflammation around the left thigh wound.
Cardiovascular	Strongly pulsatile graft indicative of good vascular flow, but risk of vascular compromise.
Integumentary	Unhealthy and necrotic patch on the skin; purulent discharge from the wound.
Renal	Elevated serum creatinine indicating impaired renal function.

5. Working Diagnoses:
 - Post-operative wound infection with Enterobacter cloacae complex, Klebsiella pneumoniae, Proteus mirabilis, Morganella morganni, and Enterococcus faecalis.
 - Acute kidney injury secondary to infection or contrast nephropathy post-CT.

- Compromised vascular graft requiring monitoring.

6. Relevant Investigations:

Investigation	Rationale
Repeat blood cultures	To monitor for systemic infection and effectiveness of antibiotics.
Wound swab and tissue cultures	To ascertain current microbial flora and antibiotic sensitivities.
Serum lactate levels	To assess for signs of sepsis.
Renal function tests	To monitor kidney function and progress of acute kidney injury.
Doppler ultrasound of graft	To assess vascular flow and graft integrity.

7. Management Plan:
- Continue broad-spectrum antibiotics piperacillin-tazobactam and consider tailoring based on culture sensitivities.
- Daily review by the nursing team for signs of infection and potential oral antibiotic switch.
- Weekly review in a multidisciplinary team meeting for comprehensive care planning.
- Monitor renal function and consider nephrology consultation for acute kidney injury.
- Regular wound care and assessment for the need for further debridement or surgical intervention.
- Ensure patient's haemodynamic stability and adequate hydration.
- Consider the need for further imaging to assess for abscess or other complications.

8. Executive Summary:
The patient is an 83-year-old female with an infected surgical wound post wide excision and vascular reconstruction for left medial thigh sarcoma. Management includes continued broad-spectrum antibiotics, daily nursing reviews, and weekly multidisciplinary assessments. Investigations should focus on monitoring for systemic infection and renal function. The goal is to manage infection, support renal function, and maintain graft viability.

Gemini Pro

1. Chronological Events

Date	Clinical Event
February 2009	Exploration and decompression of spinal canal for spinal stenosis L4/5
June 2017	Total hip replacement for right osteoarthritis of hip
October 10, 2023	Left medial thigh sarcoma post wide excision and vascular reconstruction

November 6, 2023	Transferred back to hospital for urgent CT scan due to persistent unhealthy, purulent wound bed
November 7, 2023	CT scan reveals fluid collection with rim-enhancement and gas locule at anterior aspect of upper thigh
November 7, 2023	Intra-operative findings include skin necrosis, turbid fluid, and unhealthy medial edge of rectus femoris
November 7, 2023	Vascular team assesses vascular graft and proceeds with muscular flap coverage
November 7, 2023	Blood culture, tissue culture, wound swab/aspirate culture, and fluid culture are collected
November 7, 2023	Cefazolin and piperacillin-tazobactam are initiated

2. Clinical Problem List

Active	Chronic
Left medial thigh sarcoma post wide excision and vascular reconstruction	Osteoporosis
Surgical site infection	History of spinal stenosis L4/5
Hypertension	Impaired fasting glucose

3. Potential Life-Threatening Complications
* Sepsis
* Multi-organ failure
* Death

4. Clinical Findings

Organ System	Anticipated Findings
General	Afebrile, blood pressure and pulse unsupported, not septic looking
Respiratory	No respiratory distress
Cardiovascular	No signs of shock
Gastrointestinal	No abdominal pain or distension
Neurological	No neurological deficits
Musculoskeletal	Drains noted over left thigh wound
Skin	No skin rash

5. Working Diagnoses
* Surgical site infection
* Left medial thigh sarcoma

6. Relevant Investigations

Investigation	Justification

Complete blood count (CBC)	Monitor for anemia, leukocytosis, and thrombocytopenia
Comprehensive metabolic panel (CMP)	Assess renal function, electrolytes, and liver function
Blood culture	Identify the causative organism(s) of the surgical site infection
Tissue culture	Identify the causative organism(s) of the surgical site infection
Wound swab/aspirate culture	Identify the causative organism(s) of the surgical site infection
Fluid culture	Identify the causative organism(s) of the surgical site infection
X-ray of the left thigh	Assess for osteomyelitis or hardware failure
MRI of the left thigh	Assess for soft tissue involvement or abscess formation

7. Management Plan
* Continue broad-spectrum antibiotics (piperacillin-tazobactam) until culture results are available |
* Once culture results are available, tailor antibiotics to target the specific causative organism(s) |
* Monitor for signs and symptoms of sepsis |
* Perform serial wound care and dressing changes |
* Consider surgical debridement if the wound infection does not respond to antibiotics |
* Manage underlying medical conditions (hypertension, impaired fasting glucose) |

8. Executive Summary
The patient is an 83-year-old female with a history of osteoporosis, spinal stenosis, hypertension, and impaired fasting glucose. She underwent left medial thigh sarcoma resection and vascular reconstruction in October 2023. She was transferred back to the hospital in November 2023 for an urgent CT scan due to a persistent unhealthy, purulent wound bed. The CT scan revealed a fluid collection with rim-enhancement and gas locule at the anterior aspect of the upper thigh. Intra-operatively, skin necrosis, turbid fluid, and an unhealthy medial edge of the rectus femoris were noted. The vascular team assessed the vascular graft and proceeded with muscular flap coverage. Blood culture, tissue culture, wound swab/aspirate culture, and fluid culture were collected. Cefazolin and piperacillin-tazobactam were initiated. The patient is currently afebrile, blood pressure and pulse unsupported, on room air, and not septic looking. The working diagnoses are surgical site infection and left medial thigh sarcoma. The management plan includes continuing broad-spectrum antibiotics, monitoring for signs and symptoms of sepsis, performing serial wound care and dressing changes, considering surgical debridement if the wound infection does not respond to antibiotics, and managing underlying medical conditions.

Claude 2

1. Chronological Events

Date	Event
Prior to current admission	History of spinal stenosis L4/5 with decompression. Right hip osteoarthritis status post total hip replacement. Hypertension. Impaired fasting glucose.
10 October 2023	Left medial thigh sarcoma wide excision and vascular reconstruction.
During rounds	Noted persistent unhealthy, purulent wound bed.
6 November 2023	Transferred back for urgent CT scan. Fluid collection with rim enhancement and gas locule noted.
Intraoperative	Skin necrosis, turbid fluid collection, unhealthy rectus femoris noted. Vascular graft assessed as pulsatile, proceed with flap coverage.

2. Clinical Problem List

Active Issues	Chronic Issues
Infected wound bed post-op	Hypertension
Wound dehiscence	Impaired fasting glucose
Rectus femoris necrosis	Osteoporosis
Fluid collection with gas locules	Spinal stenosis status post decompression
Acute kidney injury	Right hip osteoarthritis status post total hip replacement

3. Potential Life-Threatening Complications
- Sepsis/Septic shock from infected fluid collection
- Acute kidney injury progressing to renal failure
- Uncontrolled bleeding from friable vessels in wound bed

4. Clinical Findings

Neurologic	Normal GCS, no deficits
Cardiovascular	Tachycardia, hypotension
Respiratory	Tachypnea
Abdominal	Soft, non-tender
Extremities	Left thigh wound with purulent drainage, skin necrosis

5. Working Diagnoses
- Infected wound dehiscence
- Necrotizing fasciitis
- Abscess/fluid collection with gas locules
- Acute kidney injury

6. Relevant Investigations

Investigation	Rationale

|-|-|
Blood cultures	Identify bacteremia
Imaging - CT scan	Assess wound, look for abscess, gas locules
Tissue culture	Identify organisms for antibiotic coverage
Foley catheter	Monitor urine output given acute kidney injury
Lab tests - CBC, renal panel	Assess degree of infection, renal function

7. Management Plan
- Initiate broad spectrum antibiotics after cultures obtained
- Surgical debridement of necrotic tissue
- Drainage of abscess collection
- Flap coverage of wound once infection controlled
- Avoid nephrotoxins given acute kidney injury
- Maintain euvolemia and renal perfusion
- Strict blood glucose control
- DVT prophylaxis

8. Executive Summary
83yo female with infected thigh wound post-sarcoma resection now with wound dehiscence, necrosis, abscess, and acute kidney injury; requires broad antibiotics, surgical debridement, flap coverage once infection controlled, tight glucose control, DVT prophylaxis, and avoidance of nephrotoxins to prevent sepsis complications and progression of acute kidney injury.

36. Clinical scenario 36

GPT-4.0

1. Chronological Events:
Date	Event
19 November 2023	Admitted for hepatic encephalopathy and coffee ground vomiting
19 November 2023	Intubated in ICU
19 November 2023	OGD performed revealing two small post-esophageal varices banding ulcers
19 November 2023	Complete obliteration of the esophageal varices on air insufflation noted
20 November 2023	Extubated
20 November 2023	Rifaximin added
27 November 2023	CSU test reveals ESBL-producing Klebsiella pneumoniae

2. Clinical Problem List:
Status	Clinical Issues

Active	Hepatic encephalopathy
Active	Coffee ground vomiting
Active	ESBL-producing Klebsiella pneumoniae infection
Chronic	Alcoholic liver cirrhosis
Chronic	Esophageal varices, recurrent bleeding
Chronic	Hepatorenal syndrome
Chronic	Spontaneous bacterial peritonitis
Chronic	Ascites requiring tapping every 2 weeks
Chronic	Diuretic-resistant ascites
Chronic	Diabetes mellitus

3. Potential Life-Threatening Complications:
- Variceal hemorrhage leading to shock or further episodes of hepatic encephalopathy.
- Progressive hepatorenal syndrome leading to kidney failure.
- Spontaneous bacterial peritonitis leading to sepsis.
- Multiorgan failure due to underlying liver disease and acute infections.

4. Clinical Findings:

Organ System	Findings
Abdomen	Distended, non-tender on palpation
Neurological	Signs of hepatic encephalopathy (not detailed)
Renal	Fluctuating renal function test (baseline)

5. Working Diagnoses:
- Worsening of hepatic encephalopathy.
- Gastrointestinal bleeding likely secondary to esophageal varices.
- Urinary tract infection with ESBL-producing Klebsiella pneumoniae.

6. Relevant Investigations:

Investigation	Justification
Repeat OGD	Assess for rebleeding of esophageal varices or other lesions.
Liver function tests	Monitor progression of liver cirrhosis and hepatic function.
Renal function tests	Follow up on hepatorenal syndrome status.
Paracentesis	Evaluate ascites fluid for infection or changes in composition.
Complete blood count	Check for anemia due to bleeding, infection, or bone marrow suppression.
Blood cultures	Identify potential bacteremia or sepsis.

7. Management Plan:
- Continue supportive care for hepatic encephalopathy, including lactulose and rifaximin.

- Monitor for signs of rebleeding and manage varices as indicated.
- Antibiotic therapy tailored to the culture results (sensitivity to ESBL-producing Klebsiella pneumoniae).
- Regular monitoring of renal function and management of hepatorenal syndrome.
- Ascites management with regular paracentesis as needed, and salt restriction.
- Blood glucose control for the management of diabetes mellitus.
- Evaluate candidacy for liver transplantation, considering the patient's overall health status and prognosis.

8. Executive Summary:
This 75-year-old male presents with hepatic encephalopathy, recurrent esophageal varices bleeding, hepatorenal syndrome, spontaneous bacterial peritonitis, and diuretic-resistant ascites, indicative of advanced liver disease. The immediate concern is the management of hepatic encephalopathy and the risk of variceal rebleeding. A urinary tract infection with ESBL-producing Klebsiella pneumoniae has been identified, necessitating targeted antibiotic therapy. A comprehensive management plan addressing the complications of liver cirrhosis and preventing further decompensation is crucial. Liver transplantation evaluation should be considered as a definitive management option.

cGPT-4

1. Chronological Events:

Date	Event
75 years	Patient age
Unknown	Diagnosis of alcoholic liver cirrhosis, hepatic encephalopathy, esophageal varices, hepatorenal syndrome, spontaneous bacterial peritonitis, and diabetes mellitus
Unknown	Ascites development requiring biweekly tapping, diuretic-resistant (off aldactone)
Unknown	Decision not for TIPS due to history of hepatic encephalopathy
Unknown	Positive HBs Ab and HBc Ab, negative HBs Ag and HCV Ab
19 November 2023	Admission for hepatic encephalopathy and coffee ground vomiting
19 November 2023	Intubation and ICU admission
19 November 2023	OGD performed, esophageal varices Forrest 1, no active bleeding
20 November 2023	Extubation and addition of rifaximin
27 November 2023	CSU: ESBL-producing Klebsiella pneumoniae detected

2. Clinical Problem List:

Status	Clinical Issues

Chronic	Alcoholic liver cirrhosis
Chronic	Hepatic encephalopathy
Chronic	Esophageal varices
Chronic	Hepatorenal syndrome
Chronic	Spontaneous bacterial peritonitis
Chronic	Ascites
Chronic	Diabetes mellitus
Active	ESBL-producing Klebsiella pneumoniae infection

3. Potential Life-Threatening Complications:
- Acute on chronic liver failure
- Variceal hemorrhage
- Hepatorenal syndrome progression
- Spontaneous bacterial peritonitis sepsis
- Multi-organ failure

4. Clinical Findings:

Organ System	Findings
General	Afebrile
Abdomen	Distended, non-tender
Renal	No loin pain, no dysuria

5. Working Diagnoses:
- Exacerbation of hepatic encephalopathy
- Gastrointestinal bleeding likely secondary to esophageal varices
- ESBL-producing Klebsiella pneumoniae urinary tract infection

6. Relevant Investigations:

Investigation	Justification
Complete blood count (CBC)	Assess for anemia, infection, or thrombocytopenia
Liver function tests (LFTs)	Monitor liver function and severity of cirrhosis
Renal function tests	Monitor for hepatorenal syndrome progression
Coagulation profile	Assess bleeding risk due to liver dysfunction
Abdominal ultrasound with Doppler	Evaluate ascites and hepatic blood flow
Repeat OGD	Monitor for rebleeding from esophageal varices

7. Management Plan:
- Continue carvedilol for esophageal varices bleeding history.
- Manage hepatic encephalopathy: lactulose, rifaximin, and monitor mental status.
- Antibiotic therapy for ESBL-producing Klebsiella pneumoniae, considering sensitivity.

- Supportive care: fluids, electrolyte management, nutritional support.
- Close monitoring of renal function and consider management options for hepatorenal syndrome.
- Regular paracentesis for ascites management and symptom relief.
- Assess for the need for blood transfusions based on hemoglobin levels and clinical signs of anemia.
- Multidisciplinary team approach for weekly review and daily nursing assessment.
- Prospective data collection for audit and review.

8. Executive Summary:

The patient, a 75-year-old male with a history of alcoholic liver cirrhosis and other complications, was admitted for hepatic encephalopathy and coffee ground vomiting. Given the complexity of his conditions, a multidisciplinary approach is warranted for close monitoring and management, with specific attention to the ESBL-producing Klebsiella pneumoniae infection and potential variceal rebleeding. Management includes continuation of current medications, appropriate antibiotic therapy, and supportive measures, along with regular team reviews and prospective data collection for ongoing assessment.

Gemini Pro

1. Chronological Events:

Date	Clinical Event
19 November 2023	Admission for hepatic encephalopathy and coffee ground vomiting. Intubation in ICU.
19 November 2023	Oesophago-Gastro-Duodenoscopy (OGD): Two small post esophageal varices banding ulcer without active bleeding. Esophageal varices above were Forrest 1 only. Complete obliteration of esophageal varices on air insufflation. Decision made against further hemostasis. Fr 12 feeding tube inserted under direct vision avoiding the two areas of post banding ulcer.
20 November 2023	Extubation.
27 November 2023	Catheter Specimen Urine (CSU) culture: Extended-Spectrum Beta-Lactamases (ESBL)-producing Klebsiella pneumoniae.

2. Clinical Problem List:

Active	Chronic
Hepatic encephalopathy	Alcoholic liver cirrhosis
Spontaneous bacterial peritonitis	Hepatorenal syndrome
Diuretic-resistant ascites	Esophageal varices
Urinary tract infection (ESBL-producing Klebsiella pneumoniae)	Diabetes mellitus

3. Potential Life-Threatening Complications:
* Hepatic encephalopathy: Cerebral edema, coma, death.
* Spontaneous bacterial peritonitis: Sepsis, septic shock, death.
* Diuretic-resistant ascites: Hyponatremia, seizures, death.
* Esophageal varices: Bleeding, aspiration pneumonia, death.
* Urinary tract infection (ESBL-producing Klebsiella pneumoniae): Sepsis, septic shock, death.

4. Clinical Findings:

Organ System	Anticipated Physical Examination Findings
General	Afebrile, abdominal distension
Respiratory	Intubated, breath sounds clear bilaterally
Cardiovascular	No abnormalities
Gastrointestinal	Non-tender abdomen on palpation, no loin pain, no dysuria
Neurological	Not assessed
Musculoskeletal	Not assessed
Integumentary	Not assessed

5. Working Diagnoses:
* Hepatic encephalopathy
* Spontaneous bacterial peritonitis
* Diuretic-resistant ascites
* Esophageal varices
* Urinary tract infection (ESBL-producing Klebsiella pneumoniae)

6. Relevant Investigations:

Investigation	Justification
Complete blood count	To assess for anemia, leukocytosis, or thrombocytopenia.
Liver function tests	To assess liver function and monitor for hepatic encephalopathy.
Renal function tests	To assess renal function and monitor for hepatorenal syndrome.
Electrolytes	To assess for hyponatremia or other electrolyte imbalances.
Coagulation studies	To assess for coagulopathy.
Abdominal ultrasound	To assess for ascites, hepatomegaly, and splenomegaly.
Urine culture	To identify the causative organism and determine antimicrobial susceptibility.

7. Management Plan:
* Hepatic encephalopathy:
* Lactulose enemas or oral lactulose.
* Rifaximin.
* Intravenous fluids and electrolytes.

* Monitor for signs of cerebral edema.
* Spontaneous bacterial peritonitis:
* Intravenous antibiotics (e.g., ceftriaxone and metronidazole).
* Albumin infusion.
* Monitor for signs of sepsis.
* Diuretic-resistant ascites:
* Consider paracentesis.
* Monitor for signs of hyponatremia.
* Esophageal varices:
* Continue carvedilol.
* Consider endoscopic variceal ligation or sclerotherapy.
* Urinary tract infection (ESBL-producing Klebsiella pneumoniae):
* Intravenous antibiotics (e.g., piperacillin-tazobactam or cefepime).
* Monitor for signs of sepsis.

8. Executive Summary:
The patient is a 75-year-old male with alcoholic liver cirrhosis, hepatic encephalopathy, spontaneous bacterial peritonitis, diuretic-resistant ascites, esophageal varices, and a urinary tract infection (ESBL-producing Klebsiella pneumoniae). He is at risk for several life-threatening complications, including cerebral edema, sepsis, and bleeding. The management plan includes lactulose enemas or oral lactulose, rifaximin, intravenous fluids and electrolytes, intravenous antibiotics, albumin infusion, and monitoring for complications.

Claude 2

1. Chronological Events

Date	Event
19 November 2023	Admission for hepatic encephalopathy and coffee ground vomiting. Intubated in ICU. OGD done showing post-banding esophageal ulcer without active bleeding. Feeding tube inserted.
20 November 2023	Extubated
27 November 2023	CSU showed ESBL-producing Klebsiella pneumoniae UTI

2. Clinical Problem List

Problem	Status
Alcoholic liver cirrhosis	Chronic
Hepatic encephalopathy	Chronic, Acute exacerbation
Esophageal varices with recurrent bleeding	Chronic
Hepatorenal syndrome	Chronic
Spontaneous bacterial peritonitis	Chronic

Ascites requiring tapping	Chronic
Diuretic resistant ascites	Chronic
ESBL Klebsiella UTI	Active

3. Potential Life-Threatening Complications
- Variceal bleeding
- Hepatic encephalopathy leading to airway compromise
- Sepsis from SBP or UTI

4. Clinical Findings

System	Expected Findings
General	Cachexia, jaundice, asterixis
Cardiovascular	Tachycardia, hypotension
Respiratory	Tachypnea
Abdominal	Distended abdomen with shifting dullness, no abdominal tenderness
Neurological	Altered mental status, asterixis

5. Working Diagnoses
- Decompensated cirrhosis
- Hepatic encephalopathy
- Spontaneous bacterial peritonitis
- Urinary tract infection with ESBL-producing Klebsiella pneumoniae

6. Investigations

Investigation	Rationale
Complete blood count	Leukocytosis suggests infection
Liver function tests	Assess synthetic function, rule out acute flare
Renal function tests	Assess renal impairment, check electrolyte abnormalities
Blood cultures	Diagnose bacteremia
Ascitic fluid analysis	Rule out SBP
Urine culture	Confirm UTI, guide antibiotic therapy
CT head	Rule out intracranial causes of altered mental status

7. Management Plan
- Admit to ICU for close monitoring
- Initiate airway protection if needed
- Start empiric broad-spectrum antibiotics (carbapenem)
- Look for and treat precipitants of encephalopathy (dehydration, electrolyte abnormalities, infections)
- Consider albumin infusion for SBP
- Monitor for signs of bleeding, endoscopic intervention if needed

- Nephrology input for hepatorenal syndrome
- Infectious disease input for choice of antibiotics based on cultures
- Avoid sedatives, hypnotics, opioids
- Nutrition consult for low protein diet

8. Executive Summary

This 75-year-old male with alcoholic cirrhosis presented with hepatic encephalopathy and gastrointestinal bleeding. He has multiple complications of cirrhosis including recent ESBL Klebsiella UTI. He needs close monitoring in ICU, empiric antibiotics, and management of precipitants while looking for life-threatening complications like variceal bleed and sepsis.

37. Clinical scenario 37

GPT-4.0

1. Chronological Events

Date	Event
Before 17 November 2023	Patient in previously good health.
17-29 November 2023	Epigastric and abdominal discomfort with reduced appetite.
29 November 2023	Father last saw patient well.
1 December 2023	Patient collapsed and found unresponsive.
1-2 December 2023	Admitted to Hospital R; diagnosed with acute liver failure; underwent elective intubation and treatments for coagulopathy.
2 December 2023	Transferred to Hospital Q; tested for various infections and evaluated for liver transplantation.

2. Clinical Problem List

Status	Clinical Issues
Active	Acute liver failure
Active	Epigastric and abdominal discomfort
Active	Reduced appetite
Active	Non-responsive state (Ammonia 118 umol/L)
Chronic	Past CMV infection (CMV IgG positive)
Chronic	Immunity to Hepatitis B (HBs Ab and HBc Ab positive)

3. Potential Life-Threatening Complications

- Acute liver failure can lead to encephalopathy (evidenced by high ammonia levels and non-responsive state), coagulopathy, renal failure, and sepsis.
- Hepatic encephalopathy could progress to cerebral edema and intracranial hypertension.
- Coagulopathy may result in spontaneous bleeding or hemorrhage.

4. Clinical Findings

Organ System	Anticipated Findings
General	Fever, signs of fatigue
Neurological	Altered mental status
Gastrointestinal	Abdominal discomfort
Hepatic	Jaundice, hepatic encephalopathy
Hematological	Bruising or bleeding due to coagulopathy

5. Working Diagnoses
- Acute liver failure of unknown etiology
- Hepatic encephalopathy
- Possible drug-induced hepatotoxicity or viral hepatitis despite negative serologies

6. Relevant Investigations

Investigation	Justification
Liver function tests	To assess the severity and progression of liver failure
Ammonia levels	To monitor hepatic encephalopathy
Abdominal ultrasound	To visualize liver structure and detect possible obstructions
Toxicology screen	To rule out drug-induced hepatotoxicity
Autoimmune liver disease panel	To rule out autoimmune hepatitis
Blood cultures	To rule out sepsis as a complication of liver failure

7. Management Plan
- Immediate management of acute liver failure, including maintaining airway protection (continued intubation) and hemodynamic stability.
- Correction of coagulopathy with fresh frozen plasma and vitamin K.
- Assessment and correction of electrolyte imbalances and acid-base disorders.
- Lactulose and rifaximin for the treatment of hepatic encephalopathy.
- Consideration of liver transplantation due to severity of liver failure.
- Monitor for signs of infection, sepsis, and apply supportive measures as needed.

8. Executive Summary

The patient is a 60-year-old female with acute liver failure, currently non-responsive with an elevated ammonia level. She has a history of good health but recently experienced epigastric discomfort and reduced appetite. She has evidence of past CMV infection and immunity to Hepatitis B. Immediate life-threatening complications include hepatic encephalopathy and coagulopathy. Management priorities include stabilization, correction of coagulopathy, management of hepatic encephalopathy, and evaluation for liver transplantation. The patient's condition is critical, and close monitoring and comprehensive care are imperative.

cGPT-4

1. Chronological Events:

Date	Event
29 Nov 2023	Last seen well by father.
1 Dec 2023	Found collapsed, admitted to Hospital R for acute liver failure. Received coagulopathy correction and CVC insertion.
2 Dec 2023	Transferred to Hospital Q. Diagnosed with past CMV infection, immune to Hep B, positive for HBc Ab.

2. Clinical Problem List:

Status	Clinical Issues
Active	Acute liver failure
Active	Coagulopathy
Active	Epigastric and abdominal discomfort
Chronic	Past CMV infection
Chronic	Immunity to Hepatitis B

3. Potential Life-Threatening Complications:
- Hepatic encephalopathy (indicated by elevated ammonia levels and non-responsiveness)
- Coagulopathy leading to hemorrhage
- Increased risk of infection due to compromised liver function

4. Clinical Findings:

Organ System	Findings
Neurological	Non-responsive (Ammonia 118 umol/L)
Gastrointestinal	Epigastric and abdominal discomfort
Hepatic	No stigmata of chronic liver disease

5. Working Diagnoses:
- Acute liver failure of unknown etiology
- Coagulopathy secondary to liver failure
- Hepatic encephalopathy

6. Relevant Investigations:

Investigation	Justification
Liver function tests	To assess the extent of liver damage
Abdominal ultrasound/CT scan	To visualize the liver and identify abnormalities
Toxicology screen	To rule out drug-induced liver injury

| Serologies for viral hepatitis| To exclude viral causes of liver failure |

7. Management Plan:
- Immediate management of hepatic encephalopathy with lactulose or rifaximin.
- Correction of coagulopathy with fresh frozen plasma and vitamin K as already initiated.
- Monitoring for signs of infection and administering prophylactic antibiotics if warranted.
- Assessment for liver transplantation eligibility and preparation for possible transplant.
- Supportive care including nutritional support and management of other organ dysfunctions.

8. Executive Summary:
The patient, a 60-year-old female with no known drug allergies and non-smoker, was admitted with acute liver failure and coagulopathy. She has a history of good past health and immunity to Hepatitis B with a past CMV infection. Immediate life-threatening complications include hepatic encephalopathy and risk of hemorrhage which are being managed with appropriate interventions. A comprehensive management plan has been developed to address her acute condition, with a liver transplant team consulted and no contraindications for transplantation found.

Gemini Pro

1. Chronological Events
Date	Event
29 November 2023	Patient last seen well by her father.
1 December 2023	Patient found collapsed in her room.
2 December 2023	Patient transferred to Hospital Q.

2. Clinical Problem List
Active	Chronic
Acute liver failure	None

3. Potential Life-Threatening Complications
* Hepatic encephalopathy
* Cerebral edema
* Liver failure
* Multi-organ failure
* Death

4. Clinical Findings

Organ System	Findings
Respiratory	Kick of fever
Neurological	Non-responsive (Ammonia 118 umol/L)
Gastrointestinal	Epigastric and abdominal discomfort, reduced appetite
Skin	No skin rash
Lymphatic	No cervical lymphadenopathy

5. Working Diagnoses
* Acute liver failure
* Cytomegalovirus (CMV) infection
* Hepatitis B virus (HBV) infection

6. Relevant Investigations

Investigation	Justification
Liver function tests	To assess liver function and monitor for progression of liver failure.
Complete blood count	To assess for anemia, leukocytosis, and thrombocytopenia.
Coagulation studies	To assess for coagulopathy.
Viral serology	To confirm the diagnosis of CMV and HBV infection.
Liver biopsy	To determine the cause of liver failure and assess for the severity of liver damage.

7. Management Plan
* Supportive care:
* Intravenous fluids and electrolytes
* Oxygen therapy
* Nutritional support
* Prevention of infections
* Antiviral therapy:
* Ganciclovir for CMV infection
* Entecavir or tenofovir for HBV infection
* Liver transplantation:
* If the patient's condition does not improve with supportive care and antiviral therapy, liver transplantation may be necessary.

8. Executive Summary

A 60-year-old woman with acute liver failure was transferred to Hospital Q. She has a history of CMV and HBV infection. The patient is currently non-responsive and has a kick of fever. Investigations are underway to confirm the diagnosis and determine the cause of liver failure. The patient is being managed with supportive care, antiviral therapy, and is being evaluated for liver transplantation.

Claude 2

1. Chronological Events
Date	Event
2 weeks prior to 1 December 2023	Onset of epigastric and abdominal discomfort, reduced appetite
1 December 2023	Found collapsed at home
1 December 2023	Intubated, CVC inserted
1 December 2023 - 2 December 2023	Hospital R admission for acute liver failure
2 December 2023	Transferred to Hospital Q

2. Clinical Problem List
Problem	Active/Chronic
Acute liver failure	Active
Coagulopathy	Active
Altered mental status	Active

3. Potential Life-Threatening Complications
- Cerebral edema leading to increased intracranial pressure
- Sepsis from line infection
- Multiorgan failure
- Hemorrhage from coagulopathy

4. Anticipated Physical Examination Findings
System	Expected Findings
Neurological	Decreased level of consciousness, flapping tremor
Cardiovascular	Tachycardia
Respiratory	Tachypnea
Gastrointestinal	Distended abdomen
Integument	Jaundice, ecchymosis

5. Working Diagnoses
- Acute liver failure secondary to viral hepatitis
- Acetaminophen toxicity
- Autoimmune hepatitis
- Wilson's disease

6. Relevant Investigations
Investigation	Justification
Liver function tests	Evaluate liver synthetic function

Coagulation profile	Assess coagulopathy
Viral hepatitis serology	Look for hepatitis A, B, C and E
Ceruloplasmin, 24-hour urine copper	Evaluate for Wilson's disease
Serum acetaminophen level	Look for acetaminophen toxicity
Autoimmune workup	If autoimmune hepatitis suspected

7. Management Plan
- Admit to intensive care for close monitoring
- Identify and treat underlying cause
- Manage coagulopathy - FFP, vit K
- Consider N-acetylcysteine for possible acetaminophen toxicity
- Consider steroids if autoimmune hepatitis likely
- Monitor for cerebral edema, increased ICP
- Avoid nephrotoxic drugs
- Low threshold for liver transplant if no improvement

8. Executive Summary
This is a 60-year-old woman with acute liver failure likely from viral hepatitis versus acetaminophen toxicity. She requires close monitoring in the ICU for complications like cerebral edema and sepsis. After a diagnostic workup to identify the underlying etiology, appropriate treatment should be initiated, including N-acetylcysteine, steroids, or liver transplant if no improvement. Coagulopathy and multiorgan failure should be anticipated and managed aggressively.

38. Clinical scenario 38

GPT-4.0

1. Chronological Events
Date	Event
September 2023	Admission for long stricture at CHD, ERCP with stenting, biliary sepsis treated with Meropenem.
28 September 2023	CT showing dilated intrahepatic ducts, possible stent occlusion.
7 October 2023	Right internal-external PTBD performed, bile culture grew Pseudomonas aeruginosa resistant to imipenem, treated with Ceftolozane-tazobactam.
25 October 2023	Readmission with abdominal distension, pain, and fever.
26 October 2023	Ultrasound showing liver parenchymal disease, septated gallbladder, pancreatic and renal cysts.
27 October 2023	ERCP with removal of old pigtail stents and insertion of new stents into left and right anterior hepatic ducts.

2. Clinical Problem List
| Status| Clinical Issues |

Active	Metastatic carcinoma of gallbladder
Active	Recent acute cholangitis
Active	Biliary sepsis
Chronic	Metastasis to distal lymph nodes and peritoneal disease
Chronic	Liver tumor in segments 8 and 4
Chronic	Multiple regional and distal left neck lymph nodes
Chronic	Tight hilar stricture
Chronic	Previous ERCP with stenting

3. Potential Life-Threatening Complications
- Sepsis and septic shock due to recurrent infections and resistant organisms.
- Biliary obstruction leading to liver failure.
- Acute renal failure secondary to sepsis or obstructive processes.
- Rapid progression of metastatic disease.

4. Clinical Findings

Organ System	Anticipated Findings
General	Fever, potential signs of sepsis
Abdominal	Tenderness, distension
Hepatobiliary	Jaundice, RUQ tenderness, history of cholangitis
Renal	Left renal cyst observed on ultrasound
Hematological	Anemia (Hb 9.3 g/dL)
Infectious	Positive bile, sputum, urine, and ascitic fluid cultures

5. Working Diagnoses

- Biliary tract sepsis secondary to stent occlusion or infection
- Metastatic gallbladder carcinoma with liver involvement
- Cholangitis due to biliary obstruction and infection
- Complicated intra-abdominal infections due to multiple resistant organisms

6. Relevant Investigations

Investigation	Justification
Blood culture	To identify causative organisms and tailor antimicrobial therapy
Liver function tests	To assess the extent of liver damage and biliary obstruction
Repeat imaging (CT/MRI)	To evaluate the status of biliary stents, liver tumors, and abdominal pathology
Ascitic fluid analysis	To diagnose spontaneous bacterial peritonitis or other sources of infection

7. Management Plan
- Continue broad-spectrum antibiotics and tailor based on culture results.
- Monitor liver function, renal function, and complete blood count closely.
- Consider the need for further biliary intervention, such as revision of stenting or additional drainage procedures.
- Supportive care including fluid management, nutrition, and pain control.
- Palliative care consultation considering the metastatic nature of the disease.
- Discuss goals of care and potential end-of-life issues with the patient and family.

8. Executive Summary
A 54-year-old male with metastatic gallbladder carcinoma is currently facing acute complications of biliary sepsis and cholangitis. His condition is complicated by the presence of multi-drug resistant organisms and previous biliary interventions. Immediate concerns include managing sepsis, ensuring biliary drainage, and addressing the underlying malignancy. A multidisciplinary approach is paramount, involving infectious disease specialists, oncologists, and palliative care to optimize the management of his complex condition and to discuss the prognosis and goals of care with the patient and his family.

cGPT-4

1. Chronological Events:

Date	Clinical Issue
September 2023	Admission for long stricture at CHD, ERCP and stenting
September 2023	Biliary sepsis, treatment with Meropenem
28 September	CT showing dilated intrahepatic ducts, stent occlusion
7 October	Right internal-external PTBD due to bile culture findings
25 October	Readmission for increased abdominal distension, pain, fever
26 October	Ultrasound showing liver and gallbladder changes
27 October	ERCP, old pigtail stents removed, new stents inserted

2. Clinical Problem List:

Status	Clinical Issue
Active	Metastatic carcinoma of the gallbladder
Active	Biliary sepsis
Active	Acute cholangitis
Chronic	Peritoneal disease
Chronic	Liver tumor (segment 8 and 4)

3. Potential Life-Threatening Complications:
- Septic shock due to ongoing infections

- Biliary obstruction leading to liver failure
- Metastasis leading to systemic organ failure

4. Clinical Findings:

Organ System	Anticipated Findings
Abdominal	Tenderness, possible distension
Hepatic	Jaundice, RUQ pain
General	Fever, potential signs of sepsis

5. Working Diagnoses:
- Cholangitis
- Biliary obstruction
- Metastatic carcinoma of the gallbladder

6. Relevant Investigations:

Investigation	Justification
Blood culture	To identify causative organisms and guide antibiotic therapy
Liver function tests	To assess the extent of liver damage and biliary obstruction
Repeat CT or MRI	To evaluate liver tumors and assess for changes post-stenting

7. Management Plan:
- Continue with broad-spectrum antibiotics (Ceftolozane-tazobactam) and consider antibiotic adjustment based on culture sensitivity.
- Monitor for signs of sepsis and provide supportive care as needed, including fluids and vasopressors if hemodynamically unstable.
- Evaluate the need for further biliary drainage or stenting based on imaging and clinical progress.
- Address pain management and provide palliative care support given metastatic disease.
- Multidisciplinary team review, including an infection specialist, as per OPAT guidelines.

8. Executive Summary:
The patient, a 54-year-old male with metastatic gallbladder carcinoma, presents with complications of biliary obstruction and sepsis. Immediate management focuses on targeted antibiotic therapy, monitoring for sepsis, and addressing biliary drainage. Long-term care includes palliative support and multidisciplinary oversight. The patient's complex condition requires careful balancing of interventions to manage symptoms and complications.

Gemini Pro

1. Chronological Events:

Date	Clinical Issue
September 2023	Long stricture at common hepatic duct (CHD)
September 2023	Endoscopic retrograde cholangiopancreatography (ERCP) and stenting
September 2023	Biliary sepsis treated with Meropenem
October 7, 2023	Right internal-external percutaneous transhepatic biliary drainage (PTBD) performed
October 25, 2023	Readmitted for increased abdominal distension, pain, and fever
October 26, 2023	Ultrasound abdomen performed
October 27, 2023	ERCP performed, old pigtail stents removed, 2 stents inserted into left and right anterior hepatic ducts

2. Clinical Problem List:

Active	Chronic
Abdominal distension	Metastatic carcinoma of gallbladder
Pain	Metastasis to distal lymph nodes
Fever	Peritoneal disease
Jaundice	Segment 8 and 4 liver tumor
Right upper quadrant tenderness	Multiple regional and distal left neck lymph nodes
PTBD output positive	
Sputum culture (25 October 2023) oral commensals	
Urine culture (25 October 2023) no growth	
Ascitic fluid culture (25 October 2023) Enterococcus faecium, Staphylococcus haemolyticus	
Bile culture (25 October 2023) Stenotrophomonas maltophilia, Klebsiella pneumoniae, Enterococcus faecium, Escherichia coli	
Blood culture (25 October 2023) pending	

3. Potential Life-Threatening Complications:
* Sepsis
* Liver failure
* Cholangitis
* Pancreatitis
* Peritonitis

4. Clinical Findings:

Organ System	Anticipated Physical Examination Findings
General	Fever, tachycardia, hypotension
Respiratory	Tachypnea, hypoxia

Cardiovascular	Tachycardia, hypotension
Gastrointestinal	Abdominal distension, pain, tenderness, jaundice
Neurological	Confusion, lethargy
Musculoskeletal	Weakness
Integumentary	Jaundice, scleral icterus

5. Working Diagnoses:
* Metastatic carcinoma of gallbladder
* Cholangitis
* Peritonitis
* Sepsis

6. Relevant Investigations:

Investigation	Justification
Complete blood count (CBC)	To assess for anemia, leukocytosis, and thrombocytopenia
Comprehensive metabolic panel (CMP)	To assess for electrolyte imbalances, liver function, and renal function
Liver function tests (LFTs)	To assess for liver damage
Blood cultures	To identify the causative organism of sepsis
Urine cultures	To identify the causative organism of urinary tract infection
Ascitic fluid culture	To identify the causative organism of peritonitis
Bile culture	To identify the causative organism of cholangitis
Abdominal ultrasound	To assess for biliary obstruction, liver abscess, and ascites
CT scan of the abdomen and pelvis	To assess for metastatic disease, liver abscess, and peritonitis
ERCP	To visualize the biliary tree and identify any strictures or obstructions

7. Management Plan:
* Administer broad-spectrum antibiotics to cover the suspected pathogens
* Perform ERCP to relieve biliary obstruction
* Drain any abscesses
* Provide supportive care, including fluids, electrolytes, and blood products
* Monitor the patient closely for signs of clinical deterioration

8. Executive Summary:
The patient is a 54-year-old male with metastatic carcinoma of the gallbladder who presented with abdominal distension, pain, and fever. He was found to have cholangitis, peritonitis, and sepsis. The patient was started on broad-spectrum antibiotics and underwent ERCP to relieve biliary obstruction. He will require close monitoring for signs of clinical deterioration.

Claude 2

1. Chronological Events

Date	Event
September 2023	Long stricture at common hepatic duct, ERCP and stenting done, treated with Meropenem for biliary sepsis
28 September 2023	CT shows dilated intrahepatic ducts, possibility of stent occlusion
7 October 2023	Right internal-external PTBD performed
25 October 2023	Readmitted for abdominal distension, pain and fever
26 October 2023	Ultrasound abdomen shows parenchymal liver disease, gallbladder thickening, pancreatic cyst and left renal cyst
27 October 2023	ERCP done, stents replaced

2. Clinical Problem List

Problem	Active/Chronic
Metastatic gallbladder carcinoma	Chronic
Biliary stricture and cholangitis	Active
Biliary sepsis	Active
Ascites	Active

3. Potential Life-Threatening Complications
- Sepsis from cholangitis or infected ascites
- Bleeding from coagulopathy due to liver dysfunction
- Respiratory compromise from large volume ascites

4. Clinical Findings

System	Expected Findings
General	Fever, tachycardia
Abdominal	RUQ tenderness, ascites, jaundice
Respiratory	Tachypnea if large ascites
Cardiovascular	Hypotension in sepsis
Extremities	Ankle edema in hypoalbuminemia

5. Working Diagnoses
- Cholangitis
- Bacteremia
- Metastatic gallbladder carcinoma
- Ascites

6. Relevant Investigations

Investigation	Justification
Blood culture	To identify bacteremia
Ascitic fluid analysis and culture	To diagnose spontaneous bacterial peritonitis
Liver function tests	To assess synthetic dysfunction from liver metastasis
Coagulation profile	To assess for coagulopathy
Tumor markers - CA19-9	To monitor gallbladder cancer

7. Management Plan
- Manage sepsis with IV fluids and antibiotics per culture sensitivities
- Analgesia for pain control
- Paracentesis for infected ascites or tense ascites impairing respiration
- Consider ERCP for persistent biliary obstruction if stenting inadequate
- Anticoagulation if coagulopathy develops
- Palliative care given advanced malignancy

8. Executive Summary
This 54-year-old man with metastatic gallbladder cancer presented with abdominal pain and fever concerning for cholangitis. He requires management of his active biliary sepsis, possible spontaneous bacterial peritonitis, and risk of coagulopathy from his chronic liver metastasis. Broad spectrum antibiotics, supportive care, and further biliary drainage are recommended while monitoring for complications of his advanced malignancy.

39. Clinical scenario 39

GPT-4.0

1. Chronological Events
Date	Event
23 Sept 2023	Started on HRZE for pulmonary TB.
24 Sept 2023	Started on piperacillin-tazobactam.
29 Oct 2023	Developed diffuse maculopapular rash, liver function test derangement.
3 Nov 2023	Diagnosed with DRESS, started on hydrocortisone. All TB meds stopped. Started amikacin, levofloxacin, and ethambutol.
6 Nov 2023	Stopped amikacin, levofloxacin, and ethambutol.
10 Nov 2023	Transferred to Hospital Q for liver transplant workup, continued hydrocortisone.
1 Nov 2023	Urine grew ESBL-producing E. coli. No growth in last blood culture.
Unspecified	CT thorax, abdomen, and pelvis showing large lung abscess, cavitating lesions, and effusions.

2. Clinical Problem List

Status	Clinical Issue
Active	Pulmonary TB with pleural fluid
Active	Drug Reaction with Eosinophilia and Systemic Symptoms (DRESS)
Active	Liver function test derangement
Active	Hepatomegaly and splenomegaly
Active	Lung abscess with air-fluid level
Chronic	Ex-smoker (potential chronic respiratory issues)

3. Potential Life-Threatening Complications
- Acute Liver Failure: Due to DRESS and marked elevation of liver enzymes.
- Sepsis: Potential for systemic infection, considering the presence of ESBL-producing E. coli and lung abscess.
- Respiratory Failure: Secondary to pulmonary TB and lung abscess.

4. Clinical Findings

Organ System	Findings
General	Tired looking, jaundice, erythema, edema
Respiratory	Occasional crepitations
Cardiovascular	Dual heart sound, no murmur
Abdominal	Soft, non-tender, hepatosplenomegaly
Skin	Diffuse maculopapular rash, no vesicles

5. Working Diagnoses
- Pulmonary tuberculosis with pleural involvement.
- Drug Reaction with Eosinophilia and Systemic Symptoms (DRESS).
- Liver derangement, likely drug-induced hepatotoxicity.
- Lung abscess.

6. Relevant Investigations

Investigation	Justification
Liver Function Tests	Monitor the progression of liver injury due to DRESS.
Chest CT scan	Assess the extent of lung abscess and TB lesions.
Blood cultures	Rule out ongoing sepsis and identify causative organisms.
Hepatitis viral serologies	Exclude viral causes of hepatitis.
HIV test	TB can be an opportunistic infection in HIV.

7. Management Plan
- Pulmonary TB: Resume anti-tuberculosis therapy with drugs not implicated in the DRESS reaction.

- DRESS: Continue hydrocortisone; consider tapering schedule and monitor for improvement in skin and liver function.
- Liver derangement: Supportive care, monitor enzymes, and assess for liver transplantation if no improvement.
- Lung abscess: Antibiotic therapy guided by sensitivity; consider drainage if not resolving.

8. Executive Summary

The patient, a 61-year-old male with diagnosed pulmonary TB and subsequent DRESS, presents with complex medical issues, including liver derangement and a lung abscess. Immediate life-threatening complications include acute liver failure, sepsis, and respiratory failure. A comprehensive management plan focusing on the resumption of TB therapy, management of DRESS, and supportive care for liver derangement is crucial. Monitoring through relevant investigations and considering liver transplantation if no improvement in liver function is also recommended.

cGPT-4

1. Chronological Events:

Date	Clinical Issue
23 September 2023	Started on HRZE for pulmonary TB
24 September 2023	Started on piperacillin-tazobactam
29 October 2023	Developed diffuse maculopapular rash, deranged liver function
3 November 2023	Diagnosis of DRESS, started on hydrocortisone
2 to 6 November 2023	Switched to amikacin, levofloxacin, and ethambutol
10 November 2023	Transferred for liver transplantation workup

2. Clinical Problem List:

Active Issues	Chronic Issues
Pulmonary TB	-
Drug Reaction with Eosinophilia and Systemic Symptoms (DRESS)	-
Hepatobiliary system involvement	-
Lung abscess with air-fluid level	-
Bilateral pleural effusion	-
Eosinophilia with atypical lymphocytosis	-
Deranged liver function tests	-
ESBL-producing E. coli in urine	-

3. Potential Life-Threatening Complications:
- Acute liver failure potentially requiring transplantation.
- Severe hypersensitivity reaction (DRESS).

- Lung abscess could lead to sepsis or respiratory failure.
- Possible progression of tuberculosis if not adequately treated.

4. Clinical Findings:

Organ System	Anticipated Findings
Integumentary	Generalized erythema, no vesicles, no mucosal involvement
Respiratory	Chest clear, occasional crepitations
Cardiovascular	Heart sound dual no murmur
Gastrointestinal	Jaundice, hepatosplenomegaly
Musculoskeletal	No specific findings provided
Neurological	No specific findings provided

5. Working Diagnoses:
- Pulmonary tuberculosis.
- Drug Reaction with Eosinophilia and Systemic Symptoms (DRESS).
- ESBL-producing E. coli urinary tract infection.
- Hepatic involvement likely secondary to DRESS or TB.

6. Relevant Investigations:

Investigation	Justification
Liver function tests	Monitor progression of liver derangement
CT scan of the chest	Assess lung abscess and TB lesions
TB culture and sensitivity	Guide appropriate antitubercular therapy
Skin biopsy	Confirm DRESS diagnosis
Ultrasound of the hepatobiliary system	Evaluate hepatosplenomegaly and thickened gallbladder wall

7. Management Plan:
- Continue monitoring and supportive care for liver function.
- Resume antituberculosis treatment with drugs not associated with the hypersensitivity reaction.
- Manage DRESS with continued corticosteroid therapy and monitor for complications.
- Antibiotic therapy for ESBL-producing E. coli with an appropriate agent based on sensitivity.
- Evaluate for liver transplantation if indicated by liver function tests and clinical status.

8. Executive Summary:
The patient presents with pulmonary TB, DRESS, and hepatic involvement necessitating close monitoring. Immediate life-threatening complications include acute liver failure and severe hypersensitivity reactions. Management involves a multidisciplinary approach with resumed targeted TB treatment, corticosteroid therapy

for DRESS, and antibiotic therapy for ESBL E. coli UTI. A liver transplantation evaluation is ongoing due to significant liver function derangement.

Gemini Pro

1. Chronological Events:

Date	Clinical Event
September 23, 2023	Pulmonary tuberculosis (TB) diagnosed in China with pleural fluid
September 24, 2023	Mycobacterium tuberculosis (TB) complex DNA polymerase chain reaction (PCR) detected, with sputum grew M. tuberculosis (sensitive to isoniazid, rifampicin, pyrazinamide, ethambutol), transferred to Hospital N for management
September 23, 2023	Started on isoniazid, rifampicin, pyrazinamide, and ethambutol (HRZE)
September 24, 2023	Started on piperacillin-tazobactam
October 29, 2023	Started to develop diffuse maculopapular rash over face, trunk, and 4 limbs since initiation of tuberculosis medication, with derangement of liver function test
November 1, 2023	Blood test showed eosinophilia with atypical lymphocytosis. Hepatitis marker unremarkable. Ultrasound of the hepatobiliary system showed hepatosplenomegaly, with diffusely thickened gallbladder wall. Computed tomography (CT) of the thorax, abdomen and pelvis showing large lung abscess with air-fluid level at left lower lung, with small cavitating lesions and centrilobular nodules in both lungs. Bilateral pleural effusion
November 3, 2023	Seen by dermatology in Hospital TM, commented as Drug Reaction with Eosinophilia and Systemic Symptoms (DRESS), started on hydrocortisone since (100 mg IV every 8 hours until now)
November 2-6, 2023	All anti-tuberculosis medication stopped on admission, temporarily switched to amikacin, levofloxacin, and ethambutol
November 10, 2023	Transferred to Hospital Q for liver transplantation workup

2. Clinical Problem List:

Active Clinical Problems	Chronic Clinical Problems
Pulmonary tuberculosis	Ex-smoker
Drug Reaction with Eosinophilia and Systemic Symptoms (DRESS)	Non-drinker
Liver dysfunction	
Large lung abscess with air-fluid level at left lower lung	
Small cavitating lesions and centrilobular nodules in both lungs	
Bilateral pleural effusion	
Eosinophilia with atypical lymphocytosis	

| Hepatosplenomegaly | |
| Diffusely thickened gallbladder wall | |

3. Potential Life-Threatening Complications:
Clinical Problem	Potential Life-Threatening Complications
Pulmonary tuberculosis	Respiratory failure, sepsis
Drug Reaction with Eosinophilia and Systemic Symptoms (DRESS)	Multi-organ failure
Liver dysfunction	Hepatic encephalopathy, liver failure
Large lung abscess with air-fluid level at left lower lung	Sepsis, empyema
Small cavitating lesions and centrilobular nodules in both lungs	Hemoptysis, respiratory failure
Bilateral pleural effusion	Respiratory failure

4. Clinical Findings:
Organ System	Anticipated Physical Examination Findings
Respiratory	Chest clear, with occasional crepitations
Cardiovascular	Heart sound dual no murmur
Gastrointestinal	Abdomen soft, non-tender
Integumentary	Generalized erythema with no vesicles seen and no mucosal involvement
Musculoskeletal	Bilateral pitting edema

5. Working Diagnoses:
Probable Diagnoses
Pulmonary tuberculosis
Drug Reaction with Eosinophilia and Systemic Symptoms (DRESS)
Liver dysfunction
Large lung abscess with air-fluid level at left lower lung
Small cavitating lesions and centrilobular nodules in both lungs
Bilateral pleural effusion

6. Relevant Investigations:
Investigation	Justification
Complete blood count (CBC) with differential	Monitor for changes in white blood cell count and differential
Liver function tests (LFTs)	Monitor for liver dysfunction
Renal function tests (RFTs)	Monitor for renal dysfunction
Blood cultures	To rule out sepsis

Sputum culture	To identify the causative organism of the lung abscess
Chest X-ray	To assess the extent of the lung abscess and pleural effusion
Computed tomography (CT) scan of the chest	To further evaluate the lung abscess and pleural effusion

7. Management Plan:
Intervention	Goal
Continue hydrocortisone	To suppress the immune response and alleviate symptoms of DRESS
Start broad-spectrum antibiotics	To treat the lung abscess and prevent sepsis
Monitor liver function tests (LFTs) and renal function tests (RFTs)	To assess for and manage liver and renal dysfunction
Provide supportive care	To maintain hydration and electrolyte balance
Consider liver transplantation	If liver dysfunction progresses

8. Executive Summary:
A 61-year-old male with a history of pulmonary tuberculosis and drug reaction with eosinophilia and systemic symptoms (DRESS) presented with liver dysfunction, large lung abscess, small cavitating lesions, centrilobular nodules in both lungs, and bilateral pleural effusion. The patient is currently being treated with hydrocortisone and broad-spectrum antibiotics. The management plan includes monitoring liver and renal function, providing supportive care, and considering liver transplantation if liver dysfunction progresses.

Claude 2

1. Chronological Events
Date	Event
23 September 2023	Diagnosed with pulmonary TB in China, started on HRZE
24 September 2023	Started on piperacillin-tazobactam
29 October 2023	Developed derangement of LFTs
3 November 2023	Diagnosed with DRESS, started on hydrocortisone
10 November 2023	Transferred to Hospital Q for liver transplant workup

2. Clinical Problem List
Clinical Problem	Active/Chronic
Pulmonary TB	Active
DRESS secondary to anti-TB medications	Active
Liver injury/failure	Active
Lung abscess	Active

ESBL E. coli bacteriuria	Active

3. Potential Life-Threatening Complications
- Septic shock from lung abscess or UTI
- Acute liver failure leading to cerebral edema
- Respiratory failure from pulmonary TB

4. Clinical Findings

System	Expected Findings
General	Jaundice, generalized rash, pitting edema
Respiratory	Decreased breath sounds, wheezing, crackles
Cardiovascular	Tachycardia
Abdominal	Hepatomegaly, splenomegaly

5. Working Diagnoses
- Drug Reaction with Eosinophilia and Systemic Symptoms (DRESS)
- Acute liver injury secondary to anti-TB medications
- Pulmonary tuberculosis
- Lung abscess
- Urinary tract infection with ESBL-producing E. coli

6. Relevant Investigations

Investigation	Rationale
Liver function tests	Evaluate severity of liver injury
Coagulation profile	Assess for coagulopathy due to liver injury
Blood cultures	Check for bacteremia
Sputum AFB smears/cultures	Monitor response to anti-TB treatment
Chest X-ray	Assess lung lesions
Urinalysis and urine culture	Diagnose UTI

7. Management Plan
- Admit to intensive care for close monitoring
- Start meropenem for broad gram negative coverage of lung abscess and UTI
- Avoid hepatotoxic medications
- Monitor LFTs, INR closely
- Manage complications of liver failure (encephalopathy, coagulopathy, cerebral edema)
- Respiratory support if required
- Infectious diseases input for anti-TB regimen
- Dermatology input for ongoing management of DRESS
- Early liver transplant workup

8. Executive Summary
This 61 year old man with pulmonary TB developed DRESS and acute liver injury after starting anti-TB treatment. He now has multiple active issues including liver failure, lung abscess, and UTI with ESBL E. coli. He requires close monitoring in ICU for complications of liver failure, optimization of respiratory status, broad spectrum antibiotics, avoidance of hepatotoxic medications, and early liver transplantation workup.

40. Clinical scenario 40

GPT-4.0

1. Chronological Events:
Date	Event
19-24 Nov 2023	School trip to Thailand; exposure to mosquitoes.
29 Nov 2023	Onset of fever, malaise, rhinorrhoea.
30 Nov 2023	Referred from Private Hospital with Dengue fever diagnosis. NS1 Antigen Positive, IgM and IgG Negative. Coronary virus OC43 RNA detected in NPS.
5 days post-onset of symptoms	Patient presents with tired appearance, sore throat, and dysphagia but subjective improvement.

2. Clinical Problem List:
Status	Clinical Issues
Active	Dengue fever
Active	Coronary virus OC43 infection
Active	Sore throat and dysphagia
Chronic	G6PD deficiency

3. Potential Life-Threatening Complications:
- Severe dehydration and shock due to Dengue Hemorrhagic Fever (DHF).
- Acute hemolysis in the presence of G6PD deficiency if exposed to oxidative drugs or infections.
- Secondary bacterial infection superimposed on viral infections.

4. Clinical Findings:
Organ System	Anticipated Findings
Integumentary	Scattered petechiae over bilateral lower limbs and right medial forearm
Lymphatic	Left-sided cervical lymphadenopathy
Gastrointestinal	Abdomen soft, non-tender

| Musculoskeletal | Absence of joint pain |

5. Working Diagnoses:
- Primary diagnosis of Dengue fever.
- Concurrent infection with Coronary virus OC43.

6. Relevant Investigations:

Investigation	Justification
Complete blood count (CBC)	To assess for hemoconcentration or leukopenia common in Dengue.
Liver function tests (LFTs)	To evaluate hepatic involvement in Dengue fever.
Dengue serology (IgM and IgG)	To confirm Dengue infection and assess for immune status.
Repeat NPS for Coronary virus	To monitor the progression of Coronary virus OC43 infection.

7. Management Plan:
- Monitor vital signs and hydration status closely for signs of Dengue shock syndrome.
- Administer analgesics and antipyretics (avoid NSAIDs due to risk of hemorrhage in Dengue).
- Ensure safe medications are used considering G6PD deficiency to avoid hemolytic anemia.
- Rest and supportive care for the Coronary virus OC43 infection.
- Educate patient and family on the signs of complications, such as bleeding, abdominal pain, persistent vomiting, or change in consciousness.

8. Executive Summary:
The patient is a 16-year-old male with a recent history of travel to Thailand, presenting with Dengue fever and infection with Coronary virus OC43. Notable clinical findings include scattered petechiae and left-sided cervical lymphadenopathy. Immediate concerns include the potential development of Dengue Hemorrhagic Fever and complications related to G6PD deficiency. Recommended investigations include CBC, LFTs, and Dengue serology to monitor the disease course and guide treatment. A management plan focusing on monitoring and supportive care, with caution due to G6PD deficiency, is advised.

cGPT-4

1. Chronological Events:

Date	Event
19-24 Nov	School trip to Thailand, mosquito bites

29 Nov	Fever onset
30 Nov	Admission to hospital, positive Dengue NS1 Antigen
30 Nov	Coronary virus OC43 RNA detected in NPS
Day 5 post-symptoms	Tired appearance, sore throat, dysphagia, subjective improvement

2. Clinical Problem List:

Status	Clinical Issue
Active	Dengue fever
Active	Coronary virus OC43 infection
Chronic	G6PD deficiency

3. Potential Life-Threatening Complications:
- Severe dengue fever, which could lead to dengue shock syndrome or dengue hemorrhagic fever
- Severe infection with Coronary virus OC43 leading to respiratory failure

4. Clinical Findings:

Organ System	Findings
Skin	Scattered petechiae on limbs and forearm
Lymphatic	Left-sided cervical lymphadenopathy
Gastrointestinal	Soft, non-tender abdomen
Musculoskeletal	No joint pain
General	Tired appearance, no fever specified

5. Working Diagnoses:
- Dengue fever
- Respiratory infection with Coronary virus OC43
- G6PD deficiency without current complications

6. Relevant Investigations:

Investigation	Justification
Complete Blood Count (CBC)	Monitor for hematologic abnormalities associated with dengue fever
Liver Function Tests (LFTs)	Assess hepatic involvement in dengue fever
Dengue Virus Serology (IgM and IgG)	Confirm dengue fever diagnosis
Repeat Nasopharyngeal Swab for OC43 RNA	Monitor Coronary virus OC43 infection
G6PD level	Assess current G6PD enzyme activity

7. Management Plan:
- Symptomatic treatment for fever and pain
- Close monitoring of blood pressure and hematocrit for signs of severe dengue
- Ensure adequate hydration
- Avoid NSAIDs due to G6PD deficiency and potential for hemorrhage in dengue
- Supportive care for Coronary virus OC43 infection
- Avoidance of drugs and foods that can trigger hemolysis in G6PD deficiency
- Regular follow-up and monitoring of liver and renal function

8. Executive Summary:
The 16-year-old male patient with G6PD deficiency presented with dengue fever post-travel and concurrent Coronary virus OC43 infection. Immediate concerns include monitoring for severe manifestations of dengue fever. Investigation with CBC, LFTs, and repeated viral testing is warranted to guide management, which currently focuses on supportive care, hydration, and avoidance of NSAIDs. Regular follow-up is essential to monitor for potential complications.

Gemini Pro

1. Chronological Events:
Date	Event
19-24 November 2023	School trip to Thailand; hiking and mosquito bites
29 November 2023	Fever onset
30 November 2023	Refer to Private Hospital for Dengue fever; Dengue virus NS1 Antigen Positive, IgM and IgG Negative; Nasopharyngeal swabs: Coronary virus OC43 RNA detected
04 December 2023	Day 5 post-symptom onset; Tired looking; Sore throat and dysphagia; Subjective improved today; No other focal complaints

2. Clinical Problem List:
Active	Chronic
Dengue fever	G6PD deficiency
Coronavirus OC43 infection	

3. Potential Life-Threatening Complications:
- Dengue fever: Dengue hemorrhagic fever, dengue shock syndrome, multi-organ failure
- Coronavirus OC43 infection: Pneumonia, acute respiratory distress syndrome (ARDS)

4. Clinical Findings:

Organ System	Findings
Skin	Scattered petechiae over bilateral lower limbs and right medial forearm
Lymph nodes	Left sided cervical lymphadenopathy, non-tender on palpation
Respiratory	Sore throat, dysphagia
Gastrointestinal	No abdominal pain or tenderness
Musculoskeletal	No joint pain

5. Working Diagnoses:
- Dengue fever
- Coronavirus OC43 infection

6. Relevant Investigations:

Investigation	Justification
Complete blood count (CBC)	To assess for thrombocytopenia, leukopenia
Dengue virus serology (IgM, IgG)	To confirm dengue infection
Dengue NS1 antigen test	To detect active dengue infection
Nasopharyngeal swab for respiratory viruses	To confirm coronavirus OC43 infection
Chest X-ray	To assess for pneumonia or other pulmonary complications

7. Management Plan:
- Dengue fever:
  - Monitor vital signs and fluid status
  - Administer fluids and electrolytes as needed
  - Monitor platelet count and hematocrit
  - Consider blood transfusion if platelet count drops below 20,000/mm3
- Coronavirus OC43 infection:
  - Supportive care
  - Monitor respiratory status
  - Administer oxygen therapy if needed
  - Consider antiviral therapy if patient develops severe disease

8. Executive Summary:
- The patient is a 16-year-old male with dengue fever and coronavirus OC43 infection.
- He is currently stable but requires close monitoring for complications.
- Investigations are recommended to confirm the diagnoses and assess for complications.
- Management includes supportive care, monitoring, and antiviral therapy if indicated.

Claude 2

1. Chronological Events

Date	Event
19-24 Nov 2023	Hiking and mosquito bites during school trip in Thailand
29 Nov 2023	Fever onset, associated with malaise and rhinorrhea
30 Nov 2023	Positive dengue NS1 antigen, negative IgM/IgG. Coronavirus OC43 detected on nasopharyngeal swab
30 Nov 2023	Referred from private hospital for dengue fever
5 days after symptom onset (3-4 Dec)	Tired looking, sore throat, dysphagia, scattered petechiae over limbs, cervical lymphadenopathy

2. Clinical Problem List

Problem	Active/Chronic
Dengue fever	Active
Coronavirus OC43 upper respiratory infection	Active
G6PD deficiency	Chronic

3. Potential Life-Threatening Complications
- Dengue hemorrhagic fever leading to shock
- Expansion of petechiae/bleeding into a significant hemorrhage

4. Anticipated Physical Examination Findings

System	Expected Findings
Constitutional	Fatigue, malaise
HEENT	Pharyngeal injection, cervical lymphadenopathy
Cardiovascular	Tachycardia
Skin	Petechiae, no rash
Lymph nodes	Cervical lymphadenopathy
Abdomen	Soft, non-tender
Musculoskeletal	No joint pain

5. Working Diagnoses
- Dengue fever
- Coronavirus OC43 upper respiratory infection

6. Investigations

Investigation	Rationale
Complete blood count	Monitor platelet count, hemorrhage risk
Coagulation studies	Assess coagulation status
Liver function tests	Monitor for hepatic involvement

Repeat dengue serology	Confirm acute dengue infection
Blood culture	Rule out bacterial co-infection
Chest X-ray	Assess for pneumonia

7. Management Plan
- Admit for close monitoring of vital signs, fluid status, bleeding risk
- Avoid NSAIDs due to bleeding risk
- Administer IV fluids judiciously, avoid overhydration
- Transfuse platelets if counts very low or significant hemorrhage
- Daily complete blood counts, coagulation studies, LFTs
- Observe for warning signs of severe dengue (abdominal pain, persistent vomiting, bleeding, fluid overload, respiratory distress)
- Discharge after afebrile x48 hours and improving platelet count

8. Executive Summary
This 16 year old male with G6PD deficiency has clinical and serological evidence of acute dengue fever likely contracted during recent travel to Thailand. He is at risk for dengue hemorrhagic fever and significant bleeding due to low platelets. He requires hospitalization for close monitoring of fluid status, complete blood counts, and bleeding risk. Treatment is supportive.
