# Supplementary material for: Generative Artificial Intelligence Models in Clinical Infectious Disease Consultations: A Cross-Sectional Analysis Among Specialists and Resident Trainees
Source: Healthcare (Basel). 2025 Mar 27;13(7):744. doi: 10.3390/healthcare13070744 (PMC11988841; doi:10.3390/healthcare13070744)
Supplement: Supplementary file 1 [file healthcare-13-00744-s001.zip › healthcare-3500109-supplementary/Supplementary file S2.pdf]

Supplementary File S2

Supplementary Appendix

Index

1.	Supplementary tables…………………………………………………………	3 – 14
1.1.	Table S1. 5-point Likert scale and evaluation rubric for the assessment of artificial intelligence (AI)-generated responses………………………………	3
1.2.	Table S2. Artificial intelligence (AI)-generated response lengths……………	4
1.3.	Table S3. Comparisons of artificial intelligence (AI)-generated response lengths (word count) between AI chatbots……………………………………	5
1.4.	Table S4. Comparisons of artificial intelligence (AI)-generated response lengths (character count) between AI chatbots……………………………..	6
1.5.	Table S5. Descriptive statistics of artificial intelligence (AI)-generated responses according to domains and evaluator groups……………………….	7
1.6.	Table S6. Comparison of composite scores between specialist and resident trainee evaluators according to generative artificial intelligence (GenAI) chatbots…………………………………………………………………..	8
1.7.	Table S7. Domain level comparisons between specialist and resident trainee evaluators according to generative artificial intelligence (GenAI) chatbots….	9
1.8.	Table S8. Incident rates for each response type, according to domain………..	10
1.9.	Table S9. Incident rates for each response type, separated by evaluator groups, arranged according to domain……………………………………...	11 – 12
1.10.	Table S10. Cost of generative artificial intelligence (GenAI) models according to token consumption……………………………………………	13
1.11.	Table S11. Running cost of generative artificial intelligence (GenAI) chatbots according to chatbots………………………………………………...	14

Table S1. 5-point Likert scale and evaluation rubric for the assessment of artificial intelligence (AI)-generated responses

Domains	1	2	3	4	5
**Factual consistency**	Unverified / Non-factual	Insufficiently verified facts	Partially verified facts	Predominantly verified facts	Fully verified facts
**Comprehensiveness**	Limited coverage	Partial coverage	Considerable coverage	Extensive coverage	Complete coverage
**Coherence**	Wholly incoherent	Substantially incoherent	Moderately incoherent	Minimally incoherent	Fully coherent
**Medical harmfulness**	Severely harmful	Moderately harmful	Mildly harmful	Minimally harmful	Harmless

Table S2. Artificial intelligence (AI)-generated response lengths

	Response length (word count)			Response length (character count)		
	**Mean (SD)**	**Minimum**	**Maximum**	**Mean (SD)**	**Minimum**	**Maximum**
**GPT-4.0**	577.2 ± 81.2	443	779	4103.0 ± 522.8	3135	5223
**cGPT-4**	507.7 ± 80.2	334	671	3701.0 ± 510.8	2676	4835
**Gemini Pro**	537.8 ± 86.2	357	793	3384.0 ± 600.5	2100	5021
**Claude 2**	439.5 ± 62.6	347	626	2789.0 ± 449.1	2122	4135

cGPT-4 = Custom Chatbot (based on GPT-4.0); SD = Standard deviation.

Table S3. Comparisons of artificial intelligence (AI)-generated response lengths (word count) between AI chatbots

	GPT-4.0	cGPT-4	Gemini Pro	Claude 2
**GPT-4.0**		MD = 69.5 (24.1-114.8) p<0.001*	MD = 39.4 (-6.0-84.7) p=0.11	MD = 137.7 (92.4-183.0) p<0.001*
**cGPT-4**			MD = -30.1 (-75.4-15.2) p=0.31	MD = 68.2 (22.9-113.6) P<0.001*
**Gemini Pro**				MD = 98.3 (53.0-143.7) p=<0.001*
**Claude 2**				

cGPT-4 = Custom Chatbot (based on GPT-4.0); MD = mean difference.

Table S4. Comparisons of artificial intelligence (AI)-generated response lengths (character count) between AI chatbots

	GPT-4.0	cGPT-4	Gemini Pro	Claude 2
**GPT-4.0**		MD = 401.8 (97.7-705.8) p=0.004*	MD = 719.3 (415.3-1023.0) p=<0.001*	MD = 1313.0 (1009.0-1617.0) p=<0.001*
**cGPT-4**			MD = 317.6 (13.5-621.6) p=0.04*	MD = 911.6 (607.5-1216.0) p=<0.001*
**Gemini Pro**				MD = 594.0 (290.0-898.1) p=<0.001*
**Claude 2**				

cGPT-4 = Custom Chatbot (based on GPT-4.0); MD = mean difference.

Table S5. Descriptive statistics of artificial intelligence (AI)-generated responses according to domains and evaluator groups

	Median (IQR)				Mean (SD)			
	GPT-4.0	Gemini Pro	Claude 2	cGPT-4	GPT-4.0	Gemini Pro	Claude 2	cGPT-4
**Factual consistency**								
**Specialist**	5 (4-5)	4 (4-5)	4 (4-5)	4 (4-5)	4.508 ± 0.535	4.367 ± 0.593	4.425 ± 0.575	4.492 ± 0.519
**Resident trainee**	4 (4-4)	4 (3-4)	4 (3-4)	4 (3.25-4)	3.892 ± 0.562	3.608 ± 0.702	3.550 ± 0.672	3.767 ± 0.590
**Overall**	4 (4-5)	4 (4-4)	4 (4-4)	4 (4-5)	4.200 ± 0.629	3.988 ± 0.752	3.988 ± 0.763	4.129 ± 0.663
**Comprehensiveness**								
**Specialist**	4 (4-5)	4 (4-5)	4 (4-5)	4 (4-5)	4.300 ± 0.544	4.158 ± 0.580	4.225 ± 0.601	4.292 ± 0.627
**Resident trainee**	4 (3-4)	4 (3-4)	4 (3-4)	4 (3-4)	3.658 ± 0.761	3.392 ± 0.813	3.308 ± 0.828	3.425 ± 0.827
**Overall**	4 (4-4)	4 (3-4)	4 (3-4)	4 (3-4)	3.979 ± 0.734	3.775 ± 0.802	3.767 ± 0.856	3.858 ± 0.851
**Coherence**								
**Specialist**	4 (4-5)	4 (4-5)	4 (4-5)	4 (4-5)	4.417 ± 0.588	4.258 ± 0.680	4.375 ± 0.674	4.483 ± 0.580
**Resident trainee**	4 (3-4)	4 (3-4)	4 (3-4)	4 (3-4)	3.808 ± 0.612	3.500 ± 0.799	3.542 ± 0.697	3.775 ± 0.667
**Overall**	4 (4-5)	4 (3-4)	4 (3-5)	4 (4-5)	4.113 ± 0.672	3.879 ± 0.832	3.958 ± 0.802	4.129 ± 0.717
**Medical harmfulness**								
**Specialist**	5 (4-5)	4 (4-5)	4 (4-5)	4 (4-5)	4.467 ± 0.673	4.367 ± 0.788	4.383 ± 0.758	4.458 ± 0.721
**Resident trainee**	4 (4-4)	4 (3-4)	4 (3-4)	4 (3-4)	3.917 ± 0.693	3.467 ± 0.898	3.542 ± 0.766	3.792 ± 0.788
**Overall**	4 (4-5)	4 (3-5)	4 (3-5)	4 (4-5)	4.192 ± 0.735	3.917 ± 0.956	3.963 ± 0.869	4.125 ± 0.833
**Composite score**								
**Specialist**	4.5 (4-5)	4.25 (4-4.75)	4.25 (4-5)	4.5 (4-5)	4.423 ± 0.481	4.288 ± 0.527	4.352 ± 0.564	4.431 ± 0.518
**Resident trainee**	3.75 (3.5-4)	3.5 (3-4)	3.5 (3-3.75)	3.75 (3.5-4)	3.819 ± 0.498	3.492 ± 0.653	3.485 ± 0.581	3.690 ± 0.590
**Overall**	4 (3.75-4.688)	4 (3.5-4.5)	4 (3.5-4.5)	4 (3.75-4.5)	4.121 ± 0.575	3.890 ± 0.714	3.919 ± 0.718	4.060 ± 0.667

cGPT-4 = Custom Chatbot (based on GPT-4.0); IQR = interquartile range; SD = standard deviation.

Table S6. Comparison of composite scores between specialist and resident trainee evaluators according to generative artificial intelligence (GenAI) chatbots

	Specialist	Resident trainee	MD (95% CI)	t	p
**GPT-4.0**	4.423	3.819	0.604 (0.499-0.709)	11.62	< 0.001
**cGPT-4**	4.431	3.690	0.742 (0.615-0.868)	11.85	< 0.001
**Gemini Pro**	4.288	3.492	0.796 (0.655-0.937)	11.41	< 0.001
**Claude 2**	4.352	3.485	0.867 (0.722-1.012)	12.09	< 0.001

Table S7. Domain level comparisons between specialist and resident trainee evaluators according to generative artificial intelligence (GenAI) chatbots

	Specialist	Resident trainee	MD (95% CI)	t	p
	**Factual consistency**				
**GPT-4.0**	4.508	3.892	0.617 (0.492-0.741)	10.03	< 0.001
**cGPT-4**	4.492	3.767	0.750 (0.612-0.888)	10.98	< 0.001
**Gemini Pro**	4.367	3.608	-0.758 (-0.929--0.588)	8.99	< 0.001
**Claude 2**	4.425	3.550	-0.850 (-1.029--0.671)	9.61	< 0.001
	**Comprehensiveness**				
**GPT-4.0**	4.300	3.658	0.642 (0.496-0.787)	8.91	< 0.001
**cGPT-4**	4.292	3.425	0.892 (0.720-1.063)	10.52	< 0.001
**Gemini Pro**	4.158	3.392	0.767 (0.572-0.962)	7.96	< 0.001
**Claude 2**	4.225	3.308	0.892 (0.692-1.091)	9.03	< 0.001
	**Coherence**				
**GPT-4.0**	4.417	3.808	0.608 (0.479-0.738)	9.48	< 0.001
**cGPT-4**	4.483	3.775	0.733 (0.575-0.892)	9.35	< 0.001
**Gemini Pro**	4.258	3.500	0.758 (0.589-0.927)	9.08	< 0.001
**Claude 2**	4.375	3.542	0.808 (0.648-0.968)	1.02	< 0.001
	**Medical harmfulness**				
**GPT-4.0**	4.467	3.917	0.550 (0.389-0.711)	6.90	< 0.001
**cGPT-4**	4.458	3.792	0.708 (0.534-0.883)	8.22	< 0.001
**Gemini Pro**	4.367	3.467	0.900 (0.704-1.096)	9.27	< 0.001
**Claude 2**	4.383	3.542	0.800 (0.630-0.970)	9.50	< 0.001

cGPT-4 = Custom Chatbot (based on GPT-4.0); MD = mean difference.

Table S8. Incident rates for each response type, according to domain

	Incidence rate (95% CI)			
	**GPT-4.0**	**cGPT-4**	**Gemini Pro**	**Claude 2**
**Factual consistency**				
**Fully verified facts**	31.25% (25.42%-37.08%)	27.50% (22.08%-33.32%)	23.33% (17.92%-28.75%)	24.58% (19.17%-29.58%)
**Predominantly verified facts**	57.92% (51.67%-63.75%)	59.98% (52.92%-65.83%)	56.25% (50.42%-62.50%)	53.33% (47.50%-59.58%)
**Partially verified facts**	10.42% (6.67-14.17%)	11.25% (7.50%-15.42%)	16.25% (11.67%-21.25%)	18.33% (13.34%-24.17%)
**Insufficiently verified facts**	0.42% (0.00%-1.25%)	1.67% (0.42%-3.33%)	4.17% (1.67%-7.08%)	3.75% (1.67%-6.25%)
**Unverified / Non-factual**	0.00% (0.00%-1.25%)	0.00% (0.00%-1.25%)	0.00% (0.00%-1.25%)	0.00% (0.00%-1.25%)
**Comprehensiveness**				
**Complete coverage**	22.08% (16.67%-27.08%)	23.33% (17.92%-29.16%)	16.25% (11.67%-20.83%)	19.17% (14.17%-24.58%)
**Extensive coverage**	57.5% (51.25%-63.33%)	45.83% (38.76%-52.08%)	52.08% (45.42%-58.33%)	45.83% (39.58%-52.08%)
**Considerable coverage**	16.67% (12.08%-21.67%)	24.17% (18.75%-30.42%)	24.58% (18.75%-30.42%)	28.33% (22.5%-34.99%)
**Partial coverage**	3.75% (1.67%-6.25%)	6.67% (3.75%-10.00%)	7.08% (3.75%-10.42%)	5.83% (2.92%-8.75%)
**Limited coverage**	0.00% (0.00%-1.25%)	0.00% (0.00%-1.25%)	0.00% (0.00%-1.25%)	0.83% (0.00%-2.08%)
**Coherence**				
**Fully coherent**	27.92% (22.50%-33.33%)	30.42% (24.59%-36.66%)	23.75% (18.33%-29.58%)	26.25% (21.25%-32.49%)
**Minimally incoherence**	56.25% (50.42%-62.08%)	54.58% (48.33%-60.83%)	45.83% (39.58%-52.5%)	47.08% (40.42%-53.33%)
**Moderately incoherent**	15.00% (10.83%-19.58%)	12.50% (8.33%-16.67%)	25.00% (19.58%-30.42%)	22.92% (17.50%-28.75%)
**Substantially incoherence**	0.83% (0%-2.08%)	2.50% (0.83%-4.58%)	5.42% (2.50%-8.74%)	3.75% (1.67%-6.25%)
**Wholly incoherence**	0.00% (0%-1.25%)	0.00% (0.00%-1.25%)	0.00% (0.00%-1.25%)	0.00% (0.00%-1.25%)
**Medical harmfulness**				
**Harmless**	36.67% (30.42%-42.92%)	36.25% (29.58%-42.08%)	30.83% (24.58%-36.25%)	29.58% (23.75%-35.83%)
**Minimally harmful**	47.50% (41.25%-54.17%)	44.58% (38.75%-50.83%)	39.58% (33.33%-45.83%)	42.92% (36.25%-49.58%)
**Mildly harmful**	14.17% (10.42%-19.17%)	14.58% (10.01%-19.17%)	21.25% (16.25%-26.66%)	22.08% (17.08%-27.50%)
**Moderately harmful**	1.67% (0.42%-3.75%)	4.58% (2.08%-7.50%)	7.08% (4.17%-10.42%)	5.00% (2.50%-7.92%)
**Severely harmful**	0.00% (0.00%-1.25%)	0.00% (0.00%-1.25%)	1.25% (0.00%-2.91%)	0.42% (0.00-1.25%)

cGPT-4 = Custom Chatbot (based on GPT-4.0); 95% CI = 95% confidence interval.

Table S9. Incident rates for each response type, separated by evaluator groups, arranged according to domain

	Incident rates (95% CI)							
	GPT-4.0		cGPT-4		Gemini Pro		Claude 2	
	Specialist	Resident trainee	Specialist	Resident trainee	Specialist	Resident trainee	Specialist	Resident trainee
**Factual consistency**								
**Fully verified facts**	26.25% (20.83%-31.67%)	5.00% (2.50%-7.92%)	25% (19.58%-30.42%)	2.5% (0.83%-4.99%)	21.25% (16.26%-26.25%)	2.08% (0.42%-4.17%)	23.33% (17.93%-28.74%)	1.25% (0.00%-2.92%)
**Predominantly verified facts**	22.92% (17.50%-28.33%)	35.00% (28.75%-41.25%)	24.58% (19.17%-30.00%)	35.00% (28.75%-41.25%)	25.83% (20.42%-31.67%)	30.42% (24.58%-36.25%)	24.58% (19.17%-30%)	28.75% (23.33%-34.58%)
**Partially verified facts**	0.83% (0.00%-2.08%)	9.58% (5.83-13.33%)	0.42% (0.00%-1.25%)	10.83% (7.08%-15.00%)	2.92% (0.83%-5.00%)	13.33% (9.18%-17.50%)	24.58% (19.17%-30%)	16.25% (11.67-21.25%)
**Insufficiently verified facts**	0.00% (0.00%-1.25%)	0.42% (0.00%-1.25%)	0.00% (0.00%-1.25%)	1.67% (0.42%-3.75%)	0.00% (0.00%-1.25%)	4.17% (1.26%-7.08%)	2.08% (0.42%-4.17%)	3.75% (1.67%-6.25%)
**Unverified / Non-factual**	0.00% (0.00%-1.25%)	0.00% (0.00%-1.25%)	0.00% (0.00%-1.25%)	0.00% (0.00%-1.25%)	0.00% (0.00%-1.25%)	0.00% (0.00%-1.25%)	0.00% (0.00%-1.25%)	0.00% (0.00%-1.25%)
**Comprehensiveness**								
**Complete coverage**	17.08% (12.5%-21.67%)	5.00% (2.50%-7.92%)	19.17% (14.58%-24.17%)	4.17% (1.67%-7.08%)	12.92% (8.75%-17.08%)	3.33% (1.25%-5.83%)	15.83% (11.25%-20.83%)	3.33% (1.25%-5.42%)
**Extensive coverage**	30.83% (25.00%-36.67%)	26.67% (20.83%-32.5%)	26.25% (20.83%-31.67%)	19.58% (14.58%-24.58%)	32.08% (26.67%-37.50%)	20.00% (15.00%-25.42%)	29.58% (23.75%-35.42%)	16.25% (11.67%-21.25%)
**Considerable coverage**	2.08% (0.42%-4.17%)	14.58% (10.00%-19.58%)	4.58% (2.08%-7.08%)	19.58% (15.00%-25.00%)	5.00% (2.50%-7.92%)	19.58% (14.58%-24.58%)	4.58% (2.08%-7.50%)	23.75% (18.33%-29.58%)
**Partial coverage**	0.00% (0.00%-1.25%)	3.75% (1.67%-6.25%)	0.00% (0.00%-1.25%)	6.67% (3.75%-10.00%)	0.00% (0.00%-1.25%)	7.08% (4.17%-10.42%)	0.00% (0.00%-1.25%)	5.83% (3.33%-8.75%)

**Limited coverage**	0.00% (0.00%-1.25%)	0.00% (0.00%-1.25%)	0.00% (0.00%-1.25%)	0.00% (0.00%-1.25%)	0.00% (0.00%-1.25%)	0.00% (0.00%-1.25%)	0.00% (0.00%-1.25%)	0.83% (0.00%-2.08%)
**Coherence**								
**Fully coherent**	23.33% (17.92%-29.17%)	4.58% (2.08%-7.50%)	26.25% (20.43%-32.49%)	4.17% (1.67%-6.67%)	19.58% (14.58%-25.00%)	4.17% (1.67%-7.08%	24.17% (18.75-30.00%)	2.08% (0.42%-4.17%)
**Minimally incoherence**	24.17% (18.76%-30.00%)	32.08% (26.25%-37.5%)	21.67% (16.67%-27.08%)	32.92% (27.08%-39.17%)	23.75% (18.75%-29.17%)	22.08% (17.08%-27.50%)	20.42% (15.83%-25.42%)	26.67% (21.25%-32.50%)
**Moderately incoherent**	2.50% (0.83%-4.58%)	12.50% (8.75%-16.67%)	2.08% (0.42%-4.17%)	10.42% (6.67%-14.17%)	6.67% (3.75%-10.41%)	18.33% (13.75%-23.33%)	5.42% (2.92%-8.33%)	17.50% (12.50%-22.08%)
**Substantially incoherence**	0.00% (0.00%-1.25%)	0.83% (0.00%-2.08%)	0.00% (0.00%-1.25%)	2.50% (0.83%-4.58%)	0.00% (0.00%-1.25%)	5.42% (2.50%-8.33%)	0.00% (0.00%-1.25%)	3.75% (1.26%-6.25%)
**Wholly incoherence**	0.00% (0.00%-1.25%)	0.00% (0.00%-1.25%)	0.00% (0.00%-1.25%)	0.00% (0.00%-1.25%)	0.00% (0.00%-1.25%)	0.00% (0.00%-1.25%)	0.00% (0.00%-1.25%)	0.00% (0.00%-1.25%)
**Medical harmfulness**								
**Harmless**	27.92% (22.08%-32.92%)	8.75% (5.42%-12.49%)	29.17% (23.75%-34.58%)	7.08% (4.17%-10.42%)	27.08% (21.67%-32.50%)	3.75% (1.67%-6.25%)	26.67% (20.84%-32.08%)	2.92% (0.83%-5.42%)
**Minimally harmful**	17.92% (12.92%-23.33%)	29.58% (23.75%-35.00%)	15.00% (10.42%-19.99%)	29.58% (23.75%-35.42%)	15.00% (10.42%-19.58%)	24.58% (19.17%-30.00%)	16.67% (12.08%-21.66%)	26.25% (20.83%-31.67%)
**Mildly harmful**	3.75% (1.67%-6.25%)	10.42% (7.08%-14.58%)	5.42% (2.50%-8.75%)	9.17% (5.83%-12.92%	7.08% (4.17%-10.42%)	14.17% (9.59%-18.75%)	5.83% (2.93%-8.75%)	16.25% (11.67%-20.42%)
**Moderately harmful**	0.42% (0.00%-1.25%)	1.25% (0.00%-2.92%)	0.42% (0.00%-1.25%)	4.17% (2.08%-6.67%)	0.83% (0.00%-2.08%)	6.25% (3.33%-9.57%)	0.83% (0.00%-2.08%)	4.17% (2.08%-6.67%)
**Severely harmful**	0.00% (0.00%-1.25%)	0.00% (0.00%-1.25%)	0.00% (0.00%-1.25%)	0.00% (0.00%-1.25%)	0.00% (0.00%-1.25%)	1.25% (0.00%-2.92%)	0.00% (0.00%-1.25%)	0.42% (0.00%-1.25%)

cGPT-4 = Custom Chatbot (based on GPT-4.0); 95% CI = 95% confidence interval.

Table S10. Cost of generative artificial intelligence (GenAI) models according to token consumption

	Cost per 1,000 tokens (£)	
	**Input**	**Output**
**GPT-4.0**	0.0474	0.0948
**Gemini Pro**	0.000079	0.000158
**Claude 2**	0.00632	0.01896

Tokens are the fragments of data processed by large language models (LLMs). Tokens may be part of a word or text that the model processes or generates. Before data input can be processed or output generated, a process known as tokenization takes place, where text data are broken down into tokens. The tokenization process may be different depending on LLMs. For the same amount of text data, different number of tokens may be required according to the model specifications. At the time of writing, publicly available token calculators for Gemini Pro and Claude 2 were not available. Therefore, we used the OpenAI (California, U.S.) tokenizer for GPT-4.0 as a point of reference, when calculating the cost of each prompt. All prices have been converted to pound sterling (GBP, £; exchange rate: US$1=£0.79, as recorded on 18 May 2024).

Table S11. Running cost of generative artificial intelligence (GenAI) chatbots according to chatbots

	Average composite score (SD)	Mean cost (£; SD)	Correlation coefficient (95% CI)	p
GPT-4.0	4.121 ± 0.575	£0.0912 ± £0.0132	0.110 (0.047-0.172)	<0.001
cGPT-4	4.060 ± 0.667	£0.0806 ± £0.0165		
Gemini Pro	3.890 ± 0.714	£0.0036 ± £0.0135		
Claude 2	3.919 ± 0.718	£0.0136 ± £0.0020		

All prices have been converted to pound sterling (GBP, £; exchange rate: US$1=£0.79, as recorded on 18 May 2024). P-value reflects the statistical significance of the correlation between the running cost of GenAI chatbot and model composite score. cGPT-4 = Custom Chatbot (based on GPT-4.0); IQR = interquartile range; SD = standard deviatio
